# Multi-response Mendelian randomization: Identification of shared and distinct exposures for multimorbidity and multiple related disease outcomes

**DOI:** 10.1016/j.ajhg.2023.06.005

**Published:** 2023-07-06

**Authors:** Verena Zuber, Alex Lewin, Michael G. Levin, Alexander Haglund, Soumaya Ben-Aicha, Costanza Emanueli, Scott Damrauer, Stephen Burgess, Dipender Gill, Leonardo Bottolo

**Affiliations:** 1 Department of Epidemiology and Biostatistics, School of Public Health, Imperial College London, London, UK; 2 MRC Centre for Environment and Health, School of Public Health, Imperial College London, London, UK; 3 UK Dementia Research Institute, Imperial College London, London, UK; 4 Department of Medical Statistics, London School of Hygiene and Tropical Medicine, London, UK; 5 Division of Cardiovascular Medicine, Perelman School of Medicine, University of Pennsylvania, Philadelphia, PA, USA; 6 Department of Medicine, Perelman School of Medicine, University of Pennsylvania, Philadelphia, PA, USA; 7 Corporal Michael J. Crescenz VA Medical Center, Philadelphia, USA; 8 Department of Brain Sciences, Faculty of Medicine, Imperial College London, London, UK; 9 National Heart and Lung Institute, Imperial College London, London, UK; 10 Department of Surgery, Perelman School of Medicine, University of Pennsylvania, Philadelphia, PA, USA; 11 Department of Genetics, Perelman School of Medicine, University of Pennsylvania, Philadelphia, PA, USA; 12 MRC Biostatistics Unit, School of Clinical Medicine, University of Cambridge, Cambridge, UK; 13 Cardiovascular Epidemiology Unit, School of Clinical Medicine, University of Cambridge, Cambridge, UK; 14 Chief Scientific Advisor Office, Research and Early Development, Novo Nordisk, Copenhagen, Denmark; 15 Department of Medical Genetics, School of Clinical Medicine, University of Cambridge, Cambridge, UK; 16 Alan Turing Institute, London, UK

**Keywords:** multimorbidity, causal inference, Mendelian randomization, instrumental variable, multi-trait models, Gaussian copula regression

## Abstract

The existing framework of Mendelian randomization (MR) infers the causal effect of one or multiple exposures on one single outcome. It is not designed to jointly model multiple outcomes, as would be necessary to detect causes of more than one outcome and would be relevant to model multimorbidity or other related disease outcomes. Here, we introduce multi-response Mendelian randomization (${MR}^{2}$), an MR method specifically designed for multiple outcomes to identify exposures that cause more than one outcome or, conversely, exposures that exert their effect on distinct responses. ${MR}^{2}$ uses a sparse Bayesian Gaussian copula regression framework to detect causal effects while estimating the residual correlation between summary-level outcomes, i.e., the correlation that cannot be explained by the exposures, and vice versa. We show both theoretically and in a comprehensive simulation study how unmeasured shared pleiotropy induces residual correlation between outcomes irrespective of sample overlap. We also reveal how non-genetic factors that affect more than one outcome contribute to their correlation. We demonstrate that by accounting for residual correlation, ${MR}^{2}$ has higher power to detect shared exposures causing more than one outcome. It also provides more accurate causal effect estimates than existing methods that ignore the dependence between related responses. Finally, we illustrate how ${MR}^{2}$ detects shared and distinct causal exposures for five cardiovascular diseases in two applications considering cardiometabolic and lipidomic exposures and uncovers residual correlation between summary-level outcomes reflecting known relationships between cardiovascular diseases.

## Teaser

Existing Mendelian randomization (MR) models consider one outcome at a time in isolation and cannot identify causes of multimorbidity. We present multi-response MR (MR^2^), an MR method specifically designed for multiple outcomes to identify exposures that cause multiple responses or, conversely, exposures that exert their effect on distinct responses.

## Introduction

Researchers focus often on understanding, preventing, and treating specific health conditions in isolation with a disease-centric approach. Yet, as life expectancy increases, the incidence of diseases increases, and a growing proportion of the adult population is affected by more than one chronic health condition.^1^^,^^2^^,^^3^ Multimorbidity describes the simultaneous presence of two or more chronic conditions in one individual.^4^ The Academy of Medical Science considers multimorbidity as a key priority for global health research,^5^ and the World Health Organization identifies people with multimorbidities at higher risk of patient safety issues.^6^ To define effective prevention and intervention strategies, it is important to understand disease etiology. Recent research into multimorbidity suggests the presence of disease clusters systematically co-occurring in subjects with specific genetic predispositions and exposed to certain exposures.^7^^,^^8^ Yet, to date, it is unclear whether multimorbidity represents a random co-occurrence of seemingly unrelated individual health conditions without a common cause or whether shared causal exposures are underpinning multiple health conditions.^3^^,^^9^ Our motivation is to develop principled causal inference methodology to detect shared or distinct causes of multiple related health outcomes using genetic evidence in a multi-trait Mendelian randomization (MR) framework.

Observational studies may be biased by unmeasured confounding factors and cannot be used to infer causality. MR uses genetic variants as instrumental variables (IVs)^10^ to infer the direct causal effect of an exposure on an outcome irrespective of unmeasured confounders.^11^^,^^12^^,^^13^ MR has become an important analytical approach to gaining a deeper understanding of how modifiable exposures impact a single disease outcome.

Yet, while there are methods for multivariable MR (MV-MR) that can deal with multiple exposures in one joint model,^14^^,^^15^ to date there is no comprehensive MR methodology that can jointly model multiple outcomes, account for information shared between the outcomes, and simultaneously detect common and distinct causes of disease. Consequently, existing MR methodology largely neglects links between related disease outcomes. For example, we have recently performed wide-angled MR investigations to look at genetic determinants of lipids and cardiovascular disease outcomes (CVDs)^16^ and blood lipids and particle sizes as exposures for coronary and peripheral artery disease (PAD)^17^ where we have performed MR analysis for each outcome separately. While this strategy provides a first scan if similar causal exposures are significantly detected across different traits, there is no principled MR methodology available to test and define whether an exposure affects more than one outcome. Moreover, existing models ignore information shared between outcomes because each trait is considered in isolation. Thus, methods are needed to make full use of the growing knowledge regarding clusters of diseases that may share the same causes. In addition, genome-wide association studies (GWASs) are generally sparsely controlled for confounders, and only a few covariates like age, sex, and principal components to control for population stratification are included in the regression model to derive summary-level data for genetic associations. While this strategy reduces the risk of collider bias, there may be potential residual confounding in the GWASs themselves, which can be addressed by jointly modeling multiple outcomes.

Here, we propose multi-response MR (${MR}^{2}$) to model multiple related health conditions in a joint multivariate (multiple outcomes) and multivariable (multiple exposures) MR model. Our motivations are the following: first, we seek to distinguish between exposures that are shared (affecting more than one outcome at the same time) or distinct (affecting only one outcome). Second, our multi-response model aims at increasing the power to detect exposures that affect more than one outcome while effectively reducing the number of false positives. Third, ${MR}^{2}$ aims to combine information between outcomes to identify the effect of unmeasured pleiotropic pathways on the responses as well as the impact of non-genetic factors (independent of the exposures), such as social health determinants, on the correlation between disease outcomes.

As the first motivating example, we want to identify which common cardiometabolic risk factors, including diabetes, dyslipidemia, hypertension, physical inactivity, obesity, and smoking, are shared or distinct causes of five cardiovascular diseases, including atrial fibrillation (AF), cardioembolic stroke (CES), coronary artery disease (CAD), heart failure (HF), and PAD. We include these outcomes because there is *a priori* epidemiological and clinical evidence that they are strongly connected due to shared risk factors and because one outcome causes another. For example, CAD can cause HF^18^^,^^19^ and AF.^20^ In turn, AF can cause CES.^21^ Consequently, in clinal practice, these cardiovascular diseases are frequently present as multimorbidity.^22^ Patients living with one disease are more likely to be affected by a second cardiovascular illness than a healthy individual becoming sick with one cardiovascular disease.^23^ Epidemiological evidence^24^^,^^25^^,^^26^^,^^27^ also suggests that these diseases share a wide range of common exposures. Determining whether these exposures are universally causal or influenced by residual confouding/correlation is challenging to infer from traditional observational study designs. To date, no study has used genetic evidence in a joint multi-outcome model to establish which exposures are shared or distinct. Here, we illustrate the advantage of using the proposed joint multivariable and multi-response ${MR}^{2}$ model to identify which cardiovascular exposures are shared or distinct for different cardiovascular conditions.

As a second motivating example, we follow up on the findings from the first example to define in more detail which lipid characteristics and lipoprotein-related traits, as measured by high-throughput metabolomics, are likely causes of the selected cardiovascular diseases.

The manuscript is outlined as follows: after material and methods, where we introduce the Bayesian modeling framework of ${MR}^{2}$, we present in results an extensive simulation study. First, we illustrate how residual correlation is caused by unmeasured shared pleiotropy, and second, we compare ${MR}^{2}$ with existing multivariable, single-outcome MR models and with other statistical learning algorithms for multi-response regression regarding their ability to detect important causal exposures, distinguish between shared and distinct exposure, and accurately estimate causal effects. Then, we present the results from the two motivating application examples. In the real examples, we contrast the results obtained by ${MR}^{2}$ with standard MV-MR^14^ and with MR with Bayesian model averaging (MR-BMA), a recently proposed method for single-trait MV-MR^15^ to highlight the gain of power and the reduction of false positives when multiple responses are jointly analyzed. We also compare ${MR}^{2}$ with MV-MR-Egger^28^ to demonstrate different effects of the unmeasured pleiotropy when dealing with multiple outcomes. Finally, we conclude with a discussion and directions for future research.

## Material and methods

In this section, we illustrate the data input utilized in the proposed method as well as in existing MR models including univariable (one exposure and one outcome) and MV-MR (multiple exposures and one outcome). Then, we describe how multiple outcomes can be modeled jointly by considering the seemingly unrelated regression (SUR) framework and how this can be generalized by the copula regression model. We show analytically and demonstrate in the simulation study (see results) how unmeasured shared pleiotropy affecting more than one outcome can be captured in a multi-response MR model, which accounts for the residual correlation between outcomes both in overlapping and non-overlapping samples in the genetic associations with the outcomes. Finally, we conclude material and methods with an overview of ${MR}^{2}$, which implements a sparse copula regression model and focuses on the selection of shared and distinct exposures for multiple health conditions. Technical details are presented in appendix A. The Markov chain Monte Carlo (MCMC) implementation of the proposed ${MR}^{2}$ method is described in supplemental information.

### MR data input

${MR}^{2}$ is formulated on summary-level data of genetic association with the exposures and outcomes from large-scale GWASs, which are commonly available in the public domain.

According to the two-sample summary-level MR framework, we assume that the genetic associations with the exposure and the genetic associations with the outcome are taken from two distinct cohorts with non-overlapping samples^29^ and are thus *a priori* independent.^30^ However, when considering multiple exposures, some of them may be derived from cohorts with full or partially overlapping samples. In this case, because MV-MR models do account for measured pleiotropy between exposures,^14^^,^^31^ overlapping samples can be analyzed. Besides modeling multiple exposures, ${MR}^{2}$ also explicitly considers the correlation between multiple responses from cohorts with fully, partially, or no overlapping samples.

In summary, the summary-level design facilitates the inclusion of different disease outcomes, as well as exposures. Moreover, they do not necessarily need to be measured on different independent cohorts or be fully or partially overlapping.

### Overview of existing MR models

Standard MR models for summary-level data, both univariable (single exposure) and multivariable (multiple exposures), are formulated as weighted linear regression models where the genetic associations with exposure are regressed against the genetic associations with the outcome. Each genetic variant, used as IVs, contributes one data point (or observation) to the regression model, which we denote with the index i, i=1,…,n. For each IV, we take the beta coefficient $\beta_{X_{i}}$ and standard error $\text{se}\left(\beta_{X_{i}}\right)$ from a univariable regression in which the exposure X is regressed on the genetic variant $G_{i}$ in sample one and beta coefficient $\beta_{Y_{i}}$ and standard error $\text{se}\left(\beta_{Y_{i}}\right)$ from a univariable regression in which the outcome Y is regressed on the genetic variant $G_{i}$ in sample two.

Then, univariable MR can be formulated as a weighted linear regression model in which the genetic associations with the outcome $\beta_{Y_{i}}$ are regressed on the genetic associations with the exposure $\beta_{X_{i}}$^32^

(Equation 1) $$\beta_{Y_{i}}=\beta_{X_{i}}\theta +\epsilon_{i},\;\epsilon_{i}∼N{(\mu ,\delta^{2}\text{se}\left(\beta_{Y_{i}}\right)}^{2}),\;i=1,\ldots ,n\text{,}$$

where θ is the effect estimate, μ is the intercept, and $\delta^{2}$ is an overdispersion parameter, $\delta^{2}\geq 1$, that incorporates residual heterogeneity into the model.^33^^,^^34^ Standard MR models set μ=0, while μ≠0 captures unmeasured horizontal pleiotropy.^35^ Weighting each genetic variant i by the first-order weights $\text{se}{\left(\beta_{Y_{i}}\right)}^{2}$ is equivalent to fitting an inverse variance weighting (IVW) MR model, which gives genetic variants measured with higher precision larger weights.^13^ Alternatively, the genetic associations may be standardized before the analysis by the weights $\omega_{i}=\text{se}\left(\beta_{Y_{i}}\right)$, i=1,…,n that only depend on the standard errors of the genetic associations with the outcome.

MV-MR^14^^,^^31^ is an extension of univariable MR to consider not just one single exposure but multiple exposures in one joint model. This joint model accounts for measured pleiotropy^14^ by modeling explicitly pleiotropic pathways via any of the included exposures. Additionally, MV-MR can be used to select the most likely causal exposures from a set of candidate exposures.^15^^,^^36^

In analogy with the univariable MR model in Equation 1, in MV-MR the genetic associations with one outcome are regressed on the genetic associations with all the exposures^37^

$$\beta_{Y_{i}}=\beta_{X_{i1}}\theta_{1}+\beta_{X_{i2}}\theta_{2}+\cdots +\beta_{X_{ip}}\theta_{p}+\epsilon_{i},\;\epsilon_{i}∼N{(\mu ,\delta^{2}\text{se}\left(\beta_{Y_{i}}\right)}^{2})\text{,}$$

or, in vector notation,

(Equation 2) $$\beta_{Y_{i}}=\beta_{X_{i}}\theta +\epsilon_{i},\;\epsilon_{i}∼N{(\mu ,\delta^{2}\text{se}\left(\beta_{Y_{i}}\right)}^{2})\text{,}$$

for each i=1,…,n, where $\beta_{Y_{i}}$ are the associations of the genetic variant $G_{i}$ with the outcome Y, $\beta_{X_{i}}$ contains the associations of the genetic variant $G_{i}$ with the p exposures, $\theta ={\left(\theta_{1},\ldots ,\theta_{p}\right)}^{T}$ is the vector of the effect estimates, μ is the intercept that models unmeasured horizontal pleiotropy, and $\delta^{2}>1$ is the overdispersion parameter.

In MV-MR, a genetic variant is a valid IV if the following criteria hold: "IV1—relevance," the variant is associated with at least one of the exposures; "IV2—exchangeability," the variant is independent of all confounders of each of the exposure-outcome associations; and "IV3—exclusion restriction," the variant is independent of the outcome conditional on the exposures and confounders.

Given these assumptions hold, we consider the effect estimates θ as the direct causal effect^37^ of the exposure on the outcome after keeping all other exposures constant.

### Multi-response MR

We present here the extension of the MV-MR model in Equation 2 for p exposures when q outcomes are jointly considered. Consequently, the observed summary-level input data are a matrix $\beta_{Y}$ of dimension n×q, which contains the genetic associations with n genetic variants with the q outcomes and $\beta_{X}$ of dimension n×p, which includes the genetic associations with the same n genetic variants with the p exposures.

In the following, we assume that the input data $\beta_{Y}$ and $\beta_{X}$ have been standardized according to IVW before the analysis, and, for ease of exposition, we use the same notation after standardization. The IVW is based on first-order weights that are proportional to the inverse of the standard error of the outcome for single-response MR models. The IVW MR model is taking into account the precision of a genetic variant and gives IVs with higher precision stronger weights in the MR model. Details on how to define IVW in a multiple-outcome framework are provided in appendix A.

The aim to model q-related outcomes in one joint model can be achieved by using the SUR framework,^38^ which is formulated as a series of q multivariable regression equations, one for each of the q outcomes.

(Equation 3) $$\begin{array}{c} \beta_{Y_{i1}}=\beta_{X_{i}}\theta_{1}+\epsilon_{i1}\\ \vdots =\vdots \\ \beta_{Y_{iq}}=\beta_{X_{i}}\theta_{q}+\epsilon_{iq} \end{array}$$

For each i=1,…,n, where $\beta_{Y_{i}}=\left(\beta_{Y_{i1}},\ldots ,\beta_{Y_{iq}}\right)$ contains the observed genetic associations with the genetic variant $G_{i}$ with the q responses, $\beta_{X_{i}}$ are the associations of the genetic variant $G_{i}$ with the p exposures, $\theta_{1}={\left(\theta_{11},\ldots ,\theta_{1p}\right)}^{T}$, …, $\theta_{q}={\left(\theta_{q1}\ldots ,\theta_{qp}\right)}^{T}$ are the outcome-specific vectors of direct causal effects for each outcome, and $\epsilon_{i1}∼N\left(\mu_{1},\delta_{1}^{2}\right),\ldots ,\epsilon_{iq}∼N\left(\mu_{q},\delta_{q}^{2}\right)$ are the residuals with $\mu_{1},\ldots ,\mu_{q}$ the response-specific intercepts and $\delta_{1}^{2},\ldots ,\delta_{q}^{2}$ the overdispersion parameters.

The SUR model connects the q multivariable regressions in Equation 3 by allowing for correlation between the q residuals of the summary-level outcomes $\epsilon_{k}$, k=1,…,q. More precisely, the SUR model estimates the (q×q)-dimensional covariance matrix between the vector of residuals $\epsilon_{i}=\left(\epsilon_{i1},\ldots ,\epsilon_{iq}\right)$.

(Equation 4) $$R=\left(\begin{array}{cccc} 1 & \rho \left(\epsilon_{i1},\epsilon_{i2}\right) & \cdots & \rho \left(\epsilon_{i1},\epsilon_{q}\right)\\ \rho \left(\epsilon_{i2},\epsilon_{i1}\right) & 1 & \cdots & \rho \left(\epsilon_{i2},\epsilon_{iq}\right)\\ \vdots & \vdots & \ddots & \vdots \\ \rho \left(\epsilon_{iq},\epsilon_{i1}\right) & \rho \left(\epsilon_{iq},\epsilon_{i2}\right) & \cdots & 1 \end{array}\right)\text{.}$$

For instance, $\rho \left(\varepsilon_{ik},\varepsilon_{ik^{\prime }}\right)$ is the correlation between the residuals of the MV-MR model for summary-level outcome k and the residuals of the MV-MR model for summary-level outcome $k^{\prime }$, $k\neq k^{\prime }=1,\ldots ,q$.

Finally, the possibility to account for response-specific unmeasured horizontal pleiotropy can be turned off by setting the vector of intercepts $\mu ={\left(\mu_{1},\ldots ,\mu_{q}\right)}^{T}$ to zero.

### Correlation between outcomes in multi-response MR

**Figure 1:**
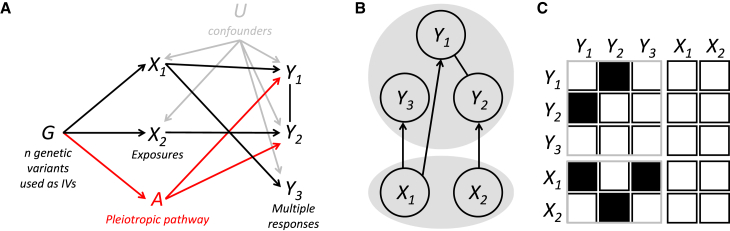
Multivariable and multi-response Mendelian randomization with multiple exposures and multiple responses (A) Directed acyclic graph (DAG) with G, genetic variant(s); $X=\left\{X_{1},X_{2}\right\}$, exposures; $Y=\left\{Y_{1},Y_{2},Y_{3}\right\}$, responses; U, unmeasured confounders; A, unobserved pleiotropic pathway. (B) Schematic representation of the ${MR}^{2}$ model, which allows the exploration of the model space consisting of all possible subsets of exposures (bottom gray circle) directly associated with the responses (top gray circle) while estimating the residual correlation between the responses (top gray circle) and vice versa. Model depicted in (A) is reported in ${MR}^{2}$ as follows: $X_{1}$ is a shared exposure for outcomes $Y_{1}$ and $Y_{3}$, while $X_{2}$ has a distinct direct causal effect on $Y_{2}$ (directed edge). Residual dependence between $Y_{1}$ and $Y_{2}$ is still present after conditioning on the associated exposures (undirected edge), and it depends on the unmeasured pleiotropic pathway A. (C) ${MR}^{2}$ model space exploration is equivalent to learning from the summary-level input data, a partitioned non-symmetrical adjacency matrix. The model depicted in (A) is represented by an adjacency matrix describing the conditional dependence structure among the responses (top left symmetrical submatrix) and the direct causal association of the exposures with the outcomes (bottom left non-symmetrical submatrix). No reverse causation is allowed in the ${MR}^{2}$ model (top right non-symmetrical submatrix), and the exposures can only have direct causal effects on the responses, i.e., no direct effects among the exposures are modeled (bottom right symmetrical submatrix).

To understand what contributes to the residuals $\varepsilon_{k}$, k=1,…,q, of the summary-level MR model in Equation 3 and generates correlation between them in Equation 4, we focus on the following generating model for the kth outcome on individual-level data considering N subjects, as illustrated in Figure 1A,

(Equation 5) $$Y_{k}=X\theta_{k}+A\theta^{A}+U\theta_{Y}^{U}+\varepsilon_{k},k=1,\ldots ,q\text{,}$$

where $\theta_{k}$ is the p-dimensional vector of effects of the exposures X on the kth outcome, $\theta^{A}$ is the effect of the unmeasured pleiotropic pathway A on the responses, $\theta_{Y}^{U}$ is the effect of the unmeasured confounder U on the outcomes, and $\varepsilon_{k}∼N_{N}\left(0,\delta_{k}^{2}I_{N}\right)$ with $\delta_{k}^{2}$ the response-specific residual variance and N the sample size. Moreover, X, A, and U are random quantities that are descendants of the same set of IVs.

Assuming that the quantitative outcomes $Y_{k}$, k=1,..,q, are measured on the same N individuals, the n-dimensional vector of summary-level genetic associations with the kth response $\beta_{Y_{k}}$ can be derived using the ordinary least squares (OLS) estimate

$$\beta_{Y_{k}}={\left(G^{T}G\right)}^{-1}G^{T}Y_{k}={\left(G^{T}G\right)}^{-1}G^{T}\left(X\theta_{k}+A\theta^{A}+U\theta_{Y}^{U}+\varepsilon_{k}\right)={\left(G^{T}G\right)}^{-1}G^{T}X\theta_{k}+{\left(G^{T}G\right)}^{-1}G^{T}\left(A\theta^{A}+U\theta_{Y}^{U}+\varepsilon_{k}\right)\text{,}$$

where the (N×n)-dimensional matrix G describes the n genetic variants associated with the exposures and measured on N individuals. Moreover, assuming that the genetic variants G selected as IVs are independent of each other (achieved by pruning) and independent of the confounder U (exchangeability assumption), the above equation simplifies to

(Equation 6) $$\beta_{Y_{k}}={\left(G^{T}G\right)}^{-1}G^{T}X\theta_{k}+{\left(G^{T}G\right)}^{-1}G^{T}A\theta^{A}+{\left(G^{T}G\right)}^{-1}G^{T}\varepsilon_{k}=\beta_{X}\theta_{k}+\beta_{A}\theta^{A}+{\tilde{\varepsilon }}_{k}\text{,}$$

where $\beta_{X}={\left(G^{T}G\right)}^{-1}G^{T}X$ is the (n×p)-dimensional matrix of the summary-level genetic association with the measured exposures X, $\beta_{A}={\left(G^{T}G\right)}^{-1}G^{T}A$ is the n-dimensional vector of genetic associations with the unmeasured pleiotropic pathway A, and ${\tilde{\varepsilon }}_{k}$ can be viewed as the OLS of the projection of $\varepsilon_{k}$ onto the linear space spanned by G. Note that $V\left({\tilde{\varepsilon }}_{k}\right)=\delta_{k}^{2}{\left(G^{T}G\right)}^{-1}$, and, given the assumption of independence of the genetic variants, it simplifies to $V\left({\tilde{\varepsilon }}_{k}\right)=\delta_{k}^{2}\text{diag}\left(v_{1}^{2},\ldots ,v_{n}^{2}\right)$, where diag(·) indicates a diagonal matrix. Thus, ${\tilde{\varepsilon }}_{k}$ is an n-dimensional vector and ${\tilde{\varepsilon }}_{k}∼N_{n}\left(0,\delta_{k}^{2}\text{diag}\left(v_{1}^{2},\ldots ,v_{n}^{2}\right)\right)$.

Consequently, the residuals in Equation 3 for the kth summary-level response, k=1,…,q, using Equation 6, can be decomposed into

(Equation 7) $$\varepsilon_{k}=\beta_{Y_{k}}-\beta_{X}\theta_{k}=\beta_{X}\theta_{k}+\beta_{A}\theta^{A}+{\tilde{\varepsilon }}_{k}-\beta_{X}\theta_{k}=\beta_{A}\theta^{A}+{\tilde{\varepsilon }}_{k}\text{,}$$

which shows that residuals in the summary-level MR model reflect unmeasured shared pleiotropy and residual variation.

Because in the designed framework the unmeasured pleiotropic pathway A is random, $\beta_{A}$ in Equation 7 is also random, and thus, $\beta_{A}\theta^{A}$ cannot be treated as the fixed-effect response-specific intercept $\mu_{k}$.^28^ Instead, it should be interpreted as a random intercept common to all summary-level responses. Similarly to random-effect models, the distribution of $\varepsilon_{k}$ follows an n-dimensional Gaussian distribution with $E\left(\varepsilon_{k}\right)=\theta^{A}\mu_{A}\text{diag}\left(v_{1}^{2},\ldots ,v_{n}^{2}\right)G^{T}1_{N}$ with $1_{N}$ an N-dimensional vector of ones and $V\left(\varepsilon_{k}\right)=\left\{{\left(\theta^{A}\right)}^{2}\sigma_{A}^{2}+\delta_{k}^{2}\right\}\text{diag}\left(v_{1}^{2},\ldots ,v_{n}^{2}\right)$, assuming A and $\varepsilon_{k}$ independent, and $E\left(A\right)=\mu_{A}1_{N}$ and $V\left(A\right)=\sigma_{A}^{2}I_{N}$ with $I_{N}$ the identity matrix of dimension N. Moreover, it reveals that the residual correlations in Equation 4 depend on the unmeasured shared pleiotropy, as illustrated below for two outcomes k and $k^{\prime }$, $k\neq k^{\prime }$,

(Equation 8) $$\rho \left(\epsilon_{ik},\epsilon_{ik^{\prime }}\right)=\frac{{\left(\theta^{A}\right)}^{2}\sigma_{A}^{2}+\sigma_{kk^{\prime }}}{{\left\{{\left(\theta^{A}\right)}^{2}\sigma_{A}^{2}+\delta_{k}^{2}\right\}}^{1/2}{\left\{{\left(\theta^{A}\right)}^{2}\sigma_{A}^{2}+\delta_{k^{\prime }}^{2}\right\}}^{1/2}}\text{,}$$

where $\sigma_{kk^{\prime }}=\text{Cov}\left(\varepsilon_{k},\varepsilon_{k^{\prime }}\right)$ is the covariance between individual-level responses’ errors in Equation 5, which is assumed constant across all N individuals.

Equation 8 shows that, independently of the sign of $\theta^{A}$ (the effect of the unmeasured pleiotropic pathway A on the responses) and the nature of the shared pleiotropy A (either “directed,” i.e., $A_{l}>0$, or “undirected,” i.e*.*, $A_{l}$ ≶ 0, l=1,…,N), the effect of unmeasured shared pleiotropy on the correlation between the residuals of the MV-MR model is always positive and constant across all combinations of responses. Moreover, the residual correlation is different from zero even when $\text{Cov}\left(\varepsilon_{k},\varepsilon_{k^{\prime }}\right)=0$, i.e., there is no correlation between individual-level responses’ errors.

Alternative scenarios can be also considered. As illustrated in Figure 1A, some outcomes may not be influenced by the unmeasured pleiotropic pathway, so their summary-level residual correlation with other responses is nil. Likewise, there may exist several unmeasured pleiotropic pathways that are shared by subgroups of responses (not necessarily distinct). For instance, pathway $A_{1}$ affects responses $\left(k_{1},k_{1}^{\prime }\right)$, $k_{1}\neq k_{1}^{\prime }$, and $A_{2}$ impacts responses $\left(k_{2},k_{2}^{\prime }\right)$, $k_{2}\neq k_{2}^{\prime }$. In these cases, the derivation of Equation 8 does not change, although $\rho \left(\epsilon_{ik},\epsilon_{ik^{\prime }}\right)$ won’t be constant across all outcomes’ pairs. Full details are presented in supplemental information.

Another important quantity in Equation 8 is $\sigma_{kk^{\prime }}$, which is the covariance between individual-level responses’ errors. In contrast with the correlation induced by unmeasured pleiotropic pathway A, which is genetically proxied by the selected IVs, $\sigma_{kk^{\prime }}$, $k\neq k^{\prime }=1,\ldots ,q$, does not depend on G. Examples of non-genetic factors that contribute to the outcomes’ correlations are, for instance, social health determinants^39^ such as personal features, socioeconomic status, culture, environment, health behaviors, access to care, and government policy. Notably, these interconnected factors that determine an individual’s health status are not associated with the exposures and, therefore, are not confounders. This echoes the instrument strength independent of direct effect (InSIDE) assumption,^35^ where the pleiotropic effect is independent of the genetic associations with the exposure, albeit here, it induces correlation between responses. Moreover, while further exposures can be included in the analysis with the hope to catch the effects of unmeasured shared pathways, the residual correlations generated by non-genetic factors cannot be “explained away” in the current MR framework. Thus, it should be considered as the baseline residual correlation between summary-level outcomes.

Finally, Equation 8 has been derived assuming that the quantitative outcomes $Y_{k}$, k=1,..,q, are measured on the same N individuals. Equation 8 still holds if non-overlapping samples of size $N_{k}$ in the genetic associations with the responses are considered with the following modification: (1) $\sigma_{kk^{\prime }}$ is equal to zero and (2) $\sigma_{A}^{2}I_{n}$ is replaced by $G_{k}^{T}\text{Cov}\left(A_{k},A_{k^{\prime }}\right)G_{k^{\prime }}$, where $G_{k}$ is the $\left(n\times N_{k}\right)$-dimensional matrix of genotypes for the group of individuals k and similarly for $G_{k^{\prime }}$. $\text{Cov}\left(A_{k},A_{k^{\prime }}\right)$ is different from zero if we assume that, since we are dealing with the same pathway A, the genetic associations with A, which are specific for responses k and $k^{\prime }$, i.e., $\beta_{A_{k}}$ and $\beta_{A_{k^{\prime }}}$, are correlated. Full details are presented in supplemental information.

### Bayesian multi-response MR

${MR}^{2}$ is based on a recently proposed Bayesian method to select important predictors in regression models with multiple responses of any type.^40^ Specifically, a sparse Gaussian copula regression (GCR) model^41^ is used to account for the multivariate dependencies between the Gaussian responses $\beta_{Y}$ once their direct causal association with a set of important exposures $\beta_{X}$ is estimated. When only Gaussian responses are considered, the GCR is similar to the SUR model^38^ (see appendix A). Figure 1B provides a schematic representation of the ${MR}^{2}$ model that allows for the estimation of important exposures (bottom gray circle) directly associated with the responses (top gray circle) while estimating the residual correlation between the responses (top gray circle) and vice versa.

Regarding ${MR}^{2}$ model specification, we use the hyper-inverse Wishart distribution as the prior density for the residual covariance matrix based on the theory of Gaussian graphical models.^42^ This prior allows some of the off-diagonal elements of the inverse covariance matrix to be identical to zero and to estimate the residual correlation between the summary-level responses. For the exposures, we use a hierarchical non-conjugate model^43^ to assign a prior distribution independently on each direct causal effect. A point mass at zero is specified on the regression coefficient of the null exposure, whereas a Gaussian distribution is assigned to the non-zero effect.

From a computational point of view, we design an efficient proposal distribution to update jointly the latent binary vectors for the selection of important exposures (selection step) and the corresponding non-zero effects (estimation step). For Gaussian responses, the designed proposal distribution allows the “implicit marginalization” of the non-zero effects in the Metropolis-Hastings (M-H) acceptance probability,^44^ which makes our MCMC algorithm more efficient than existing sparse Bayesian SUR models^45^^,^^46^^,^^47^ favoring the exploration of important combinations of exposures, i.e., the model space, in a very efficient manner (see supplemental information). It also guarantees a good acceptance rate, which, in turn, prevents slow convergence of the MCMC algorithm and reduces the autocorrelation of the posterior samples. Figure 1C shows that ${MR}^{2}$ model exploration is equivalent to learning from the input data a non-symmetrical adjacency matrix partitioned into a symmetrical submatrix (top left), which describes the conditional dependence structure among the responses and a non-symmetrical submatrix (bottom left) representing the direct causal association of the exposures with the outcomes. Note that neither reverse causation (top right submatrix) nor direct causal effects between the responses (bottom right submatrix) are allowed in the ${MR}^{2}$ model.

Two sets of parameters are deemed important in our analysis: the marginal posterior probability of inclusion (mPPI), which measures the strength of the direct causal association between each exposure-response combination and the corresponding direct causal effect, and the edge posterior probability of inclusion (ePPI), which describes the strength of the residual dependence between each pair of summary-level responses and the corresponding residual partial correlation. The posterior distribution of these quantities is usually summarized by their mean or any relevant quantile. For instance, the α/2% and (1−α/2)% quantiles are used to build the (1−α)% credible interval (CI). We summarize all quantities of interest by their posterior mean (both mPPI and ePPI can be seen as a posterior mean or frequency that an exposure-response combination or a pair of dependent responses are selected during the MCMC algorithm) and their 95% CI.

The selection of important exposures for each response and significantly correlated pairs of responses is based on mPPIs and ePPIs, respectively. Thresholding these quantities at 0.5 is usually suggested given that the optimal predictive model in linear regression is often the median probability model, which is defined as the model consisting of those predictors that have overall mPPI ≥0.5, the optimal predictive model under square loss, i.e., the optimal model that predicts not-yet-collected data.^48^ Here, we follow a different approach based on in-sample selection. A non-parametric false discovery rate (FDR) strategy based on two-component mixture models,^49^^,^^50^ which clusters low and high levels of mPPIs and low and high levels of ePPIs, is applied to select important exposures for each response and significant dependence patterns among responses at a fixed FDR level.

Importantly, for the definition of exposures causing more than one outcome, the availability of the latent binary vectors for the selection of important exposures for each response recorded during the MCMC algorithm also allows the estimation of the joint posterior probability of inclusion (jPPI), defined as the number of times an exposure is selected to be associated with two or more responses at the same time during the MCMC. Thus, jPPI can be regarded as the jPPI for any combination of responses. The detection of important direct causal effects of an exposure on a single response or multiple responses is carried out by looking at significant mPPIs selected at a nominal FDR level. If an exposure is associated with more than one response, we declare the existence of a shared direct causal effect and calculate the jPPIs.

While the residual correlation between summary-level responses captures “global” unmeasured shared pleiotropy, which is calculated across all genetic variants, we additionally screen for individual genetic variants as potential outliers due to their “local” pleiotropic effect.^51^ Building on Bayesian tools for outlier diagnostics, we propose the conditional predictive ordinate (CPO)^52^ to detect individual genetic variants as outliers or high-leverage and influential observations in the ${MR}^{2}$ model.

Full details of the ${MR}^{2}$ model, as well as post-processing of the MCMC output, are presented in appendix A.

## Results

### Simulation study: Can the effect of shared pleiotropy be detected?

Here, we conduct a simulation study to illustrate the impact of unmeasured shared pleiotropy affecting more than one outcome. We consider one of the scenarios presented in the simulation study (scenario III—undirected pleiotropy) where the residual correlation between outcomes at the summary level depends only on the unmeasured shared pleiotropy and $\sigma_{kk^{\prime }}=0$ for all responses. We look at two quantities: first, the empirical correlations between the summary-level genetic associations with the outcomes measured on the IVs. They can be computed by simply calculating the pairwise correlation between the genetic associations with the outcomes on the summary level. Second, we look at the residual correlation between the summary-level genetic associations with the outcomes measured on the IVs after accounting for the exposures and estimated by ${MR}^{2}$.

**Figure 2:**
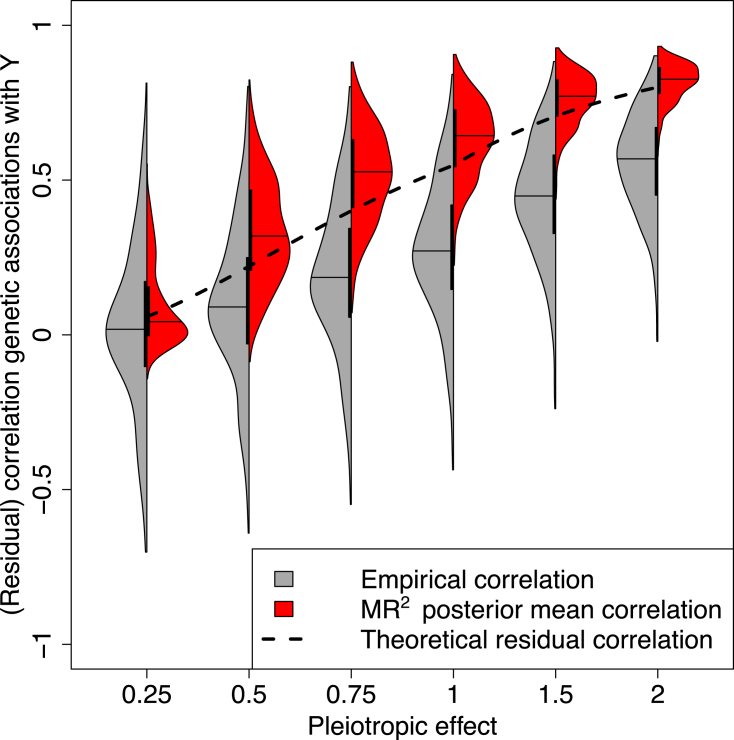
(Residual) correlation between the summary-level genetic associations with the outcomes induced by various levels of the pleiotropic effect and with sample overlap between the summary-level data of the outcomes Each violin plot depicts the empirical correlations between the summary-level genetic associations with the outcomes (dark gray) and the posterior mean of the residual correlations estimated by ${MR}^{2}$ (red) in simulation scenario III—undirected pleiotropy averaged over 50 replicates at different levels of the pleiotropy effect $\theta^{A}=\left\{0.25,0.50,0.75,1.00,1.50,2.00\right\}$. Confounding effects on the exposures and outcomes are fixed at $\theta_{X}^{U}=2$ and $\theta_{Y}^{U}=1$, respectively, without correlation between exposures and responses’ errors and assuming 100% sample overlap in the summary-level genetic associations with the outcomes. For each side of the violin plot, the vertical black thick line displays the interquartile range, while the black line indicates the median. Black dashed line depicts the theoretical value of residual correlation between summary-level outcomes as a function of $\theta^{A}$ as shown in Equation 8. For more details on the simulation setting, see Table S2.

Figure 2 shows that the larger the pleiotropic effect $\theta^{A}$ (ranging from 0.25 to 2), the bigger the empirical correlation. However, the empirical correlation (in gray) is not able to distinguish between the correlation due to the direct causal effects of the exposures on the outcomes and the unmeasured shared pleiotropy. ${MR}^{2}$ can separate the source of correlation with a good agreement between the estimated values of the residual correlation (in red) and its theoretical value derived in Equation 8 (black dashed line).

### Simulation study: Comparison of methodologies

We perform a simulation study to demonstrate the increase in power, a better estimation of direct causal effects when accounting for multiple correlated outcomes, and finally, the ability to detect shared and distinct causal exposures when using the proposed ${MR}^{2}$ method compared with existing MV-MR models that consider one outcome at a time and the recently proposed multi-response multivariable methods. In total, we simulate n=100 genetic variants used as IVs, p=15 exposures, and q=5 outcomes. Out of the p×q exposure-outcome combinations, 30% have a non-zero direct causal effect. For all scenarios, we generate N=100,000 individuals, of which half is used to generate the summary-level genetic associations for the exposure and half for the outcomes, respectively, providing data in a summary-level design. As alternative methods, we include standard MV-MR^37^ and MR-BMA,^15^ a Bayesian variable-selection approach for MV-MR. We consider two multivariable and multivariate variable-selection approaches, which have to date not been applied to MR: the multiple responses Lasso (multivariate regression with covariance estimation [MRCE])^46^ and the multiple responses Spike-and-Slab Lasso (mSSL).^47^ Both methods perform variable and covariance selection by inducing sparsity and setting the effect estimates of variables not included in the model to zero, as well as inducing sparsity in the residual covariance matrix. An overview of the alternative methods is provided in Table S1.

We simulate the following scenarios.

• Scenario I—null: there are no causal exposures for any of the outcomes and no confounder.
• Scenario II—confounding: there are 30% of exposures with a non-zero direct causal effect and there is a joint confounder for all outcomes.
• Scenario III—undirected pleiotropy: residual correlation between outcomes is induced by a shared undirected pleiotropic pathway that can increase or decrease the level of the responses.
• Scenario IV—directed pleiotropy: residual correlation between outcomes is induced by a shared directed pleiotropic pathway that only increases the level of the responses.
• Scenario V—dependence: outcomes are simulated with correlated errors, mimicking the effect of non-genetic factors that contribute to their correlation.

In the following, we consider for simplicity only the case of 100% overlapping samples in the genetic associations with the outcomes. Full details regarding the simulation study setup are presented in appendix A. An overview of the simulations setting and the open parameters ($\theta^{A}$ and $\theta_{U}^{Y}$ in Equation 5, $\theta_{U}^{X}$, the effect of the unmeasured confounder U on the exposures, and $r_{Y}$, the correlation between individual-level responses’ errors in Equation 5) that vary across the simulation scenarios are shown in Table S2. Fixed parameters, including the number of subjects in the individual-level data, the number of IVs, the range of the simulated direct causal effects, the heritability of the exposures, and the proportion of variance explained when simulating the responses, are also detailed in the same table. In supplemental information, we also provide details regarding ${MR}^{2}$ hyper-parameters setting, including the number of MCMC iterations after burn-in, as well as technical details of the alternative methods and their software implementations we used in the simulation study.

**Figure 3:**
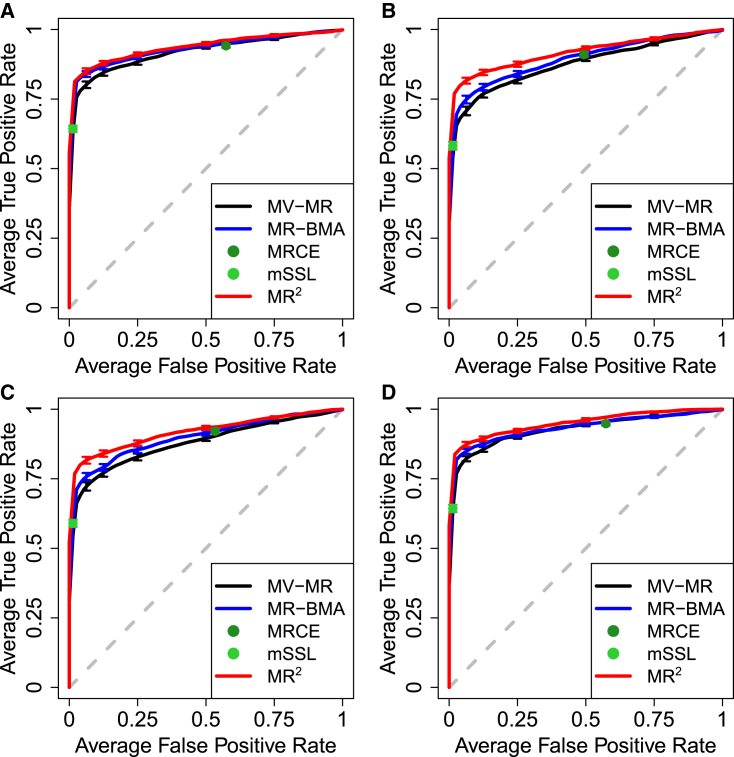
Receiver operating characteristic (ROC) curves in different simulated scenarios when the correlation between exposures is set at $r_{X}=0.6$, averaged over 50 replicates, illustrating the performance of different MR implementations and multi-response multivariable methods to distinguish between true and false causal exposures for five simulated outcomes by plotting the true positive rate (TPR) against the false positive rate (FPR) (A) Depicts baseline scenario II—confounding where only the confounding effects on the exposures and outcomes, $\theta_{X}^{U}=2$ and $\theta_{Y}^{U}=1$, respectively, are used to simulate the data. (B–D) Residual correlation induced by shared undirected pleiotropy (scenario III—undirected pleiotropy) and shared directed pleiotropy (scenario IV—directed pleiotropy) with pleiotropic effect set at $\theta^{A}=1$ are shown in (B) and (C), respectively. (D) Displays the performance of the different methods in scenario IV—dependence where the correlation between individual-level responses’ errors is fixed at $r_{Y}=0.6$. Vertical bars in each ROC curve, at specific FPR levels, indicate standard error. For more details on the simulations setting, see Table S2.

The performance in terms of exposure selection is evaluated using the receiver operating characteristic (ROC) curves where the true positive rate (TPR) is plotted against the false positive rate (FPR). As a baseline, all methods perform equally well in the case of no correlation between exposures, as seen in Figure S1. In contrast, when the exposures are correlated, all variable-selection approaches improve over the standard MV-MR, as seen in Figure 3. This improvement depends on the correlation between exposures, as shown in Figure S2.

In the following, we set the correlation between exposures at $r_{X}=0.6$, while the confounding effects on the exposures and outcomes are fixed at $\theta_{X}^{U}=2$ and $\theta_{Y}^{U}=1$, respectively. In scenario III—undirected pleiotropy and scenario IV—directed pleiotropy, the pleiotropic effect is set at $\theta^{A}=1$. Finally, in scenario V—dependence, the correlation between individual-level responses’ errors in Equation 5 is fixed at $r_{Y}=0.6$.

**Figure 4:**
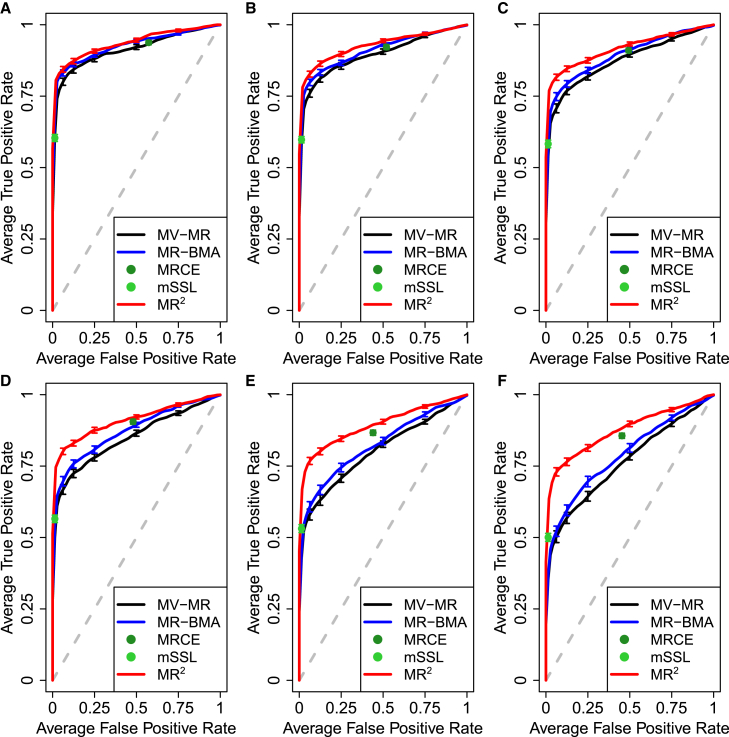
Receiver operating characteristic (ROC) curves for different levels of the pleiotropic pathway effect $\theta^{A}$ and when the correlation between exposures is set at $r_{X}=0.6$, averaged over 50 replicates, illustrating the performance of different MR implementations and multi-response statistical methods to distinguish between true and false causal exposures for five simulated outcomes by plotting the true positive rate (TPR) against the false positive rate (FPR) Pleiotropic pathway effect varies from (A)–(F) with values $\theta^{A}=\left\{0.25,0.5,0.75,1,1.5,2\right\}$. Confounding effects on the exposures and outcomes are fixed at $\theta_{X}^{U}=2$ and $\theta_{Y}^{U}=1$, respectively. Vertical bars in each ROC curve, at specific FPR levels, indicate standard error.

When residual correlation is induced by shared undirected (Figure 3B) and directed pleiotropy (Figure 3C), ${MR}^{2}$ shows a better detection of true causal exposures than MR-BMA, which in turn improves over a basic MV-MR model. As shown in Figure 4 for shared undirected pleiotropy and Figure S3 for shared directed pleiotropy, the degree of improvement depends on the strength of the pleiotropic pathway effect. In this scenario, the mSSL approach provides strong sparse solutions with many direct causal effects set to zero. In contrast, MRCE includes many more exposures in the model at the cost of a high FPR. Similar results are observed when the residual correlation between outcomes is induced directly through the correlation between individual-level responses’ errors (Figure 3D) with the degree of improvement depending on the level of correlation (Figure S6). For these scenarios, the corresponding area under the ROC curve (AUC) along with standard deviation across 50 replicates is provided in Table 1. When a pleiotropic pathway is simulated, the relative gain compared with one outcome at a time MR methods, i.e., MV-MR and MR-BMA, is around 7.15% and 4.45%, respectively, while it decreases to 2.37% and 1.71% when dependence is simulated. The relative gain is also marked with respect to mLLS and small compared with MRCE due to the opposite sparse Lasso solutions of the two methods, conservative and liberal, respectively. The AUCs of all simulated scenarios across the full range of open parameters are reported in Tables S3–S6.

**Table 1:** Area under the curve (AUC) in different simulated scenarios when the correlation between exposures is set at $r_{X}=0.6$

	Scenario II	Scenario III	Scenario IV	Scenario V
	Confounding	Undirected pleiotropy	Directed pleiotropy	Dependence
MV-MR	0.922 (0.040)	0.850 (0.059)	0.857 (0.057)	0.930 (0.037)
MR-BMA	0.932 (0.034)	0.875 (0.050)	0.876 (0.058)	0.936 (0.032)
MRCE	0.926 (0.035)	0.896 (0.046)	0.904 (0.048)	0.932 (0.037)
mSSL	0.876 (0.044)	0.834 (0.068)	0.840 (0.055)	0.869 (0.067)
${MR}^{2}$	0.939∗ (0.033)	0.912∗ (0.042)	0.917∗ (0.043)	0.952∗ (0.031)

The ability to distinguish between true and false causal exposures is evaluated by the area under the ROC curve averaged over 50 replicates with standard deviation across 50 replicates in brackets. In baseline scenario II—confounding, only the confounding effects on the exposures and outcomes, $\theta_{X}^{U}=2$ and $\theta_{Y}^{U}=1$, respectively, are used to induce residual correlation between the responses. Residual correlation induced by shared undirected pleiotropy and shared directed pleiotropy with pleiotropic effect $\theta^{A}=1$ are presented in scenario III—undirected pleiotropy and scenario IV—directed pleiotropy, respectively. In scenario IV—dependence, outcomes are simulated with the correlation between individual-level responses’ errors fixed at $r_{Y}=0.6$. Best results are denoted with an asterisk. For more details on the simulations setting, see Table S2.

Next, we compare methods according to their performance in estimating the direct causal effect strength. This is evaluated by the sum of squared errors (SSEs) presented in Table 2 and Figures S7–S11. As can be seen from Table 2, all methods show a comparable performance with negligible SSEs in scenario I—null when there are no causal exposures. In the other scenarios, when there is correlation between the exposures, the largest SSE is consistently seen for the MV-MR approach. This is in keeping with other simulation studies^15^ that have shown that MV-MR is unbiased but suffers from large variance. ${MR}^{2}$ has almost everywhere the lowest SSE compared with alternative methods as well as the lowest standard deviation across 50 replicates. The largest improvement with respect to MR-BMA is in scenario III—undirected pleiotropy and scenario IV—directed pleiotropy when the summary-level outcomes are correlated by a shared pleiotropic pathway. The multi-response implementations MRCE and mSSL perform roughly as MR-BMA in terms of SSEs but suffer either from too little (mSSL) or too much (MRCE) sparsity, which biases the direct causal effect estimates. Notably, MR-BMA performs better than both MRCE and mSSL in scenario V—dependence across all ranges of open parameters (Table S11) because the two multi-response multivariable methods are not able to identify the simulated correlation pattern between the responses’ errors, thus degrading the estimation of the direct causal effects. Instead, ${MR}^{2}$ can detect it, resulting in the lowest SSE across the full range of open parameters.

**Table 2:** Sum of squared errors (SSEs) in different simulated scenarios when the correlation between exposures is set at $r_{X}=0.6$

	Scenario I	Scenario II	Scenario III	Scenario IV	Scenario V
	Null	Confounding	Undirected pleiotropy	Directed pleiotropy	Dependence
MV-MR	0.001 (<0.001)	1.246 (0.490)	3.869 (1.502)	3.613 (1.409)	1.200 (0.464)
MR-BMA	0.000^∗^ (<0.001)	0.673 (0.302)	1.714 (1.036)	1.508 (0.663)	0.700 (0.348)
MRCE	0.001 (<0.001)	0.904 (0.516)	1.434 (0.877)	1.172 (0.618)	0.849 (0.423)
mSSL	0.000^∗^ (<0.001)	0.881 (0.374)	1.104 (0.790)	1.050 (0.700)	0.891 (1.167)
${MR}^{2}$	0.000^∗^ (<0.001)	0.567^∗^ (0.269)	0.858^∗^ (0.722)	0.821^∗^ (0.681)	0.521^∗^ (0.278)

The quality of the direct causal effect estimation is evaluated by sum of squared errors (SSEs), averaged over 50 replicates, with standard deviation across 50 replicates in brackets. Scenario I—null is simulated with the correlation between individual-level responses’ errors fixed at $r_{Y}=0.6$. In baseline scenario II—confounding, only the confounding effects on the exposures and outcomes, $\theta_{X}^{U}=2$ and $\theta_{Y}^{U}=1$, respectively, are used to induce residual correlation between the responses. Residual correlation induced by shared undirected pleiotropy and shared directed pleiotropy with pleiotropic effect $\theta^{A}=1$ are presented in scenario III—undirected pleiotropy and scenario IV—directed pleiotropy, respectively. In scenario IV—dependence, outcomes are simulated with the correlation between individual-level responses’ errors fixed at $r_{Y}=0.6$. Best results are denoted with an asterisk. For more details on the simulations setting, see Table S2.

We also assess the ability of ${MR}^{2}$ to detect shared and distinct direct causal effects. To this aim, we calculate the proportion of significant causal effects associated with either single or multiple outcomes in the same scenarios presented in Tables 1 and 2, where the correlation between exposures is fixed at $r_{X}=0.6$. For a fair comparison, we fix the type I error to be the same in all the methods, and, in particular, we set it at the level detected by mSSL given its sparse Lasso solutions with a low FPR. Specifically, we selected the threshold of Benjamini-Hochberg FDR procedure^53^ for MV-MR and mPPI for MR-BMA and ${MR}^{2}$, respectively, to match the number of false positives detected by mSSL. This was not possible for MRCE given the fixed solution of the multiple responses Lasso. Table S12 shows the power of the methods considered. ${MR}^{2}$ is the most powerful method to detect distinct direct causal effects, which is also the most likely simulated case (on average 37.3% of all exposure-response combinations), across all simulated scenarios. When the residual correlation between summary-level outcomes is simulated (scenario III—undirected pleiotropy, scenario IV—directed pleiotropy, and scenario V—dependence), ${MR}^{2}$ is also the most powerful method to detect shared direct causal effects. This is apparent when two and three shared direct causal effects are simulated. Together they account for 95% of all possible simulated shared cases. There is a clear advantage over one-response-at-a-time MR methods and less gain with respect to alternative multi-response methods as the number of the shared direct causal effects increases. When four outcomes are associated with the same risk factor, the limited number of simulated cases may not be large enough to discriminate between the power of ${MR}^{2}$ and alternative multi-response methods. Similar results are also obtained for different levels of correlation between individual-level exposures (data not shown).

Finally, results do not change much if the number of raw-level individuals is decreased to N=20,000, of which half is used to generate the summary-level genetic associations for the exposure and half for the outcomes, as well as if in the simulation study the proportion of variance explained to generate the outcomes from the exposures is decreased to $h_{X}=0.05$. For a visual comparison, interested readers can contrast Figure 4 when N=100,000 and $h_{X}=0.10$ with Figures S4 and S5 when N=20,000 and $h_{X}=0.10$, and when N=100,000 and $h_{X}=0.05$, respectively.

### Cardiometabolic exposures for cardiovascular diseases

For the first real application example, as CVDs, we consider AF, CES, CAD, HF, and PAD. Importantly, the summary-level genetic associations for PAD were derived from the Million Veteran Program, and there is no overlap in samples with the other responses, except for CES (data source: GIGASTROKE Consortium + Global Biobanks [including UK Biobank and Million Veteran Program]). Common polygenic exposures were selected according to the National Health Service guidelines on causes for CVD website (https://www.nhs.uk/conditions/coronary-heart-disease/causes/; last reviewed on the March 10, 2020). For high cholesterol, we include five major lipoprotein-related traits (apolipoprotein A1 [ApoA], apolipoprotein B [ApoB], high-density lipoprotein [HDL], low-density lipoprotein [LDL], and triglycerides [TGs]). Obesity is measured by body mass index (BMI); exercising regularly (moderate-to-vigorous intensity exercises during leisure time) is measured by physical activity (PA), defined by moderate-to-vigorous intensity exercises during leisure time; and high blood pressure is measured by systolic blood pressure (SBP). We also include a lifetime smoking index (SMOKING), a composite of smoking initiation, heaviness, duration, and cessation, and type 2 diabetes (T2D) as exposures. Given the strong epidemiological, genetical, and causal relationships between these exposures, an MV-MR design is necessary to account for potential horizontal pleiotropy and to facilitate selection of likely causal exposures. Genetic associations with exposures and outcomes are derived from publicly available summary-level data; see Table S13 for an overview of the data sources.

IVW is performed before the analysis for all outcomes and exposures using weights derived jointly from all responses, as described in appendix A. Moreover, the summary-level genetic associations with the exposures are standardized before the analysis. This allows us to interpret and compare the estimated exposures effect size for each outcome and, more importantly, across outcomes. For a complete description of the pre-processing and IV selection steps, we refer to supplemental information. Briefly, we select n=1,540 independent genetic variants associated with any of the ten exposures as IVs after clumping. Results are obtained after removing outliers or high-leverage and influential observations using scaled CPO and fitting the proposed model on the remaining n=1,533 IVs (see Figure S10).

**Figure 5:**
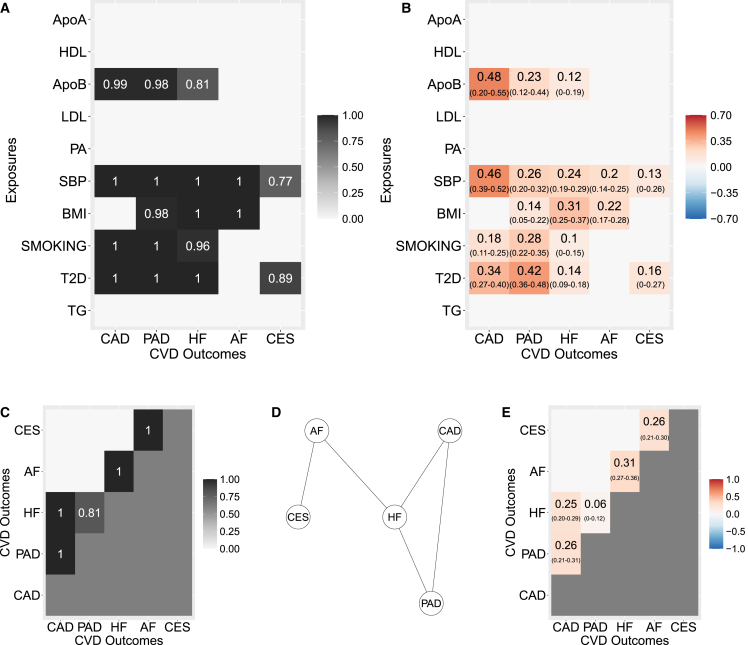
Results of the multivariable multi-response ${MR}^{2}$ model in application example 1 on common exposures for cardiovascular disease outcomes (CVDs) (A) Marginal posterior probability of inclusion (mPPI) of each exposure (y axis) against each outcome (x axis). Selected mPPIs for each exposure indicate whether an exposure is shared or distinct among multiple CVDs. (B) Posterior mean (95% credible interval) of the direct causal effect of each exposure (y axis) against each outcome (x axis). For clarity of presentation, mPPIs and estimated direct effect sizes for non-selected outcome-exposure pairs (mPPI <0.77 at 5% FDR) are not plotted. (C) Edge posterior probability of inclusion (ePPI) among outcomes. Only the upper triangular matrix is depicted. (D) Undirected graph estimated by using the selected ePPIs showing the residual dependence between outcomes not explained by the exposures. (E) Posterior mean (95% credible interval) of the partial correlations between outcomes. For clarity of presentation, ePPIs and partial correlations for non-selected outcomes pairs (ePPI <0.78 at 5% FDR) are not plotted. Only the upper triangular matrix is depicted.

${MR}^{2}$ identifies several exposures shared among CVDs, as highlighted in Figures 5A and 5B, which show the mPPI and the posterior mean direct effect sizes (95% CI) for each exposure-outcome combination, respectively. Significant mPPIs and corresponding direct effect estimates are selected, controlling FDR at 5% (see appendix A), which corresponds to mPPIs ≥0.77 (Figure S11A). For clarity of presentation, mPPIs and direct effect sizes for non-selected outcome-exposure pairs are not plotted.

Results provided by ${MR}^{2}$ can be read in two different ways: a traditional “vertical” way where, for each outcome, the significant exposures are highlighted. For instance, CAD has four significant exposures, i.e., ApoB, SBP, T2D, and SMOKING in order of importance by looking at the posterior mean direct effect estimates. PAD and HF have the same ones, although in a different order. On the other hand, genetically predicted levels of SBP and BMI are associated with AF, and genetically predicted levels of SBP and risk of T2D are associated with CES, respectively.

The main feature of the proposed methodology is that it allows reading, interpreting, and comparing the results also “horizontally” across outcomes. For instance, ApoB shows the strongest risk-increasing effect on CAD among all exposure-outcome combinations with a posterior mean direct causal effect of 0.48. Moreover, it is also selected for PAD with halved effect estimate (0.23) and around four times lower risk increase for HF (0.11). Similarly, the jPPI can be interpreted as the relevance of an exposure for a group of outcomes. For instance, ApoB has a strong jPPI of 0.78 to be jointly causal for CAD, PAD, and HF (Figure S11C). Taken together, these findings extend the results of a previous study, which found ApoB as a shared exposure for CAD and PAD^17^ and for the first time implicates a likely causal role of ApoB also for HF.

${MR}^{2}$ also provides a more rigorous statistical control of the null hypothesis (no causal effects) because it takes into account both the conditional independence among exposures (multivariable) and across responses (multi-trait). For example, conditional on ApoB, there is no evidence for any other major lipoprotein-related trait to have a likely causal role for any outcome, and conditional on CAD, PAD, and HF, there is no evidence for ApoB on any other outcome. It is an important positive control that ${MR}^{2}$ selects ApoB as the only lipid trait for CAD. Previous studies using genetic evidence and MV-MR models^17^^,^^36^^,^^54^^,^^55^^,^^56^ have shown that ApoB, representing the total number of hepatic-derived atherogenic lipoprotein particles, is the most likely causal lipid determinant of CAD, and the evidence for LDL is attenuated toward the null when accounting for ApoB.

A second example regarding the ability of ${MR}^{2}$ to disentangle complex causal relationships and advance cardiovascular domain knowledge is related to SBP. SBP is shown to increase the risk for all five CVDs considered, with a substantial jPPI of 0.77 for all five outcomes. However, SBP has the strongest posterior mean direct effect on CAD (0.46) and then it decreases steadily across the other responses (PAD [0.26], HF [0.24], AF [0.2], and CES [0.13]), suggesting that SBP lowering may not be a broad therapeutic target (as already shown in randomized clinical trials, [RCTs]) or it would require potent SBP lowering to meaningfully impact (in increasing order) HF, AF, and CES at a population level. Similar considerations can be extended to other exposures selected.

Almost all significant exposures have a large causal effect on CAD and PAD but much lower on HF, AF, and CES, except BMI on HF and AF. Therefore, it seems plausible that these traditional cardiovascular exposures are not able to fully describe the disease etiology of HF and, in particular, AF and CES. In turn, these results suggest that there may be other exposures not considered here as the main causes of these diseases. In contrast, other exposures included in this analysis, such as PA, that although considered an important exposure in medical practice (NHS guidelines), conditionally on all the others, are not significant for any CVD. This suggests that beneficial effects of PA on these outcomes is likely mediated by the other traditional cardiometabolic risk factors.

${MR}^{2}$ also acknowledges residual correlation among summary-level responses, which is not accounted for by the selected exposures as shown in Figures 5C–5E, which depict the ePPI, the indirect graph estimated by using the selected ePPIs, and the posterior mean (95% CI) of the partial correlations between outcomes, respectively. Significant ePPIs and corresponding partial correlation are selected controlling FDR at 5%, which corresponds to ePPIs ≥0.78 (see Figure S11B).

Significant residual dependence between the outcomes not explained by the exposures is identified between CAD and HF and between CAD and PAD with summary-level residual partial correlation of 0.26 and 0.25, respectively, reflecting known vertical and horizontal pleiotropy of CAD being a likely cause of HF^18^^,^^19^ and horizontal pleiotropy between CAD and PAD. In contrast, PAD and HF show significant but four times lower-level residual partial correlation (0.06). Other important residual partial correlations highlight the disease pathway HF-AF-CES, as illustrated in Figure 5C. Compared with the empirical partial correlations between summary-level genetic associations with the responses without conditioning on the exposures (Figure S8B), the genetically predicted levels of exposures are able to explain around 32% and 21% of the summary-level partial correlation between CAD and PAD and between CAD and HF, respectively, and almost 62% of the residual correlation between PAD and HF. However, not all exposures contribute in the same way to this remarkable decrease. ApoB seems not as important (jPPI =0.8) as the other associated exposures (jPPI >0.96) to explain the dependence between PAD and HF (see Figure S11C). Finally, a little reduction is observed for the disease pathway HF-AF-CES, supporting the earlier hypothesis that other important shared exposures may be missing from the proposed MR model. However, as mentioned earlier and shown in Equation 8, we cannot rule out that, besides shared pleiotropy, some non-genetic factors may be responsible for the observed residual correlation.

We conclude this section comparing the results obtained by ${MR}^{2}$ with existing MV-MR methods (see Table S1), including MV-MR-Egger^28^ to confirm that, when dealing with multiple outcomes, there are different assumptions regarding the effect of the unmeasured pleiotropy and MV-MR-Egger may not able to detect it.

MV-MR is not able to identify any lipid exposure for any outcome considered except for TGs for AF at 5% Benjamini-Hochberg FDR (Table S14). MV-MR is not designed for the analysis of many exposures that are highly correlated.^15^ Indeed, multi-collinearity reduces the precision of the estimated direct causal effects, which weakens the statistical power of the MV-MR model. Due to the strong correlation between genetic associations with lipid exposures (Figure S8C), MV-MR misses ApoB as likely causal exposure for CAD, PAD, and HF. Consequently, an intersection-union test^57^ will miss ApoB as an important risk factor jointly for CAD, PAD, and HF in contrast with ${MR}^{2}$, which assigns to ApoB a jPPI =0.78 for this group of responses. Similar results in terms of exposures selected and effect sizes are seen when MV-MR-Egger is used, with a significant unmeasured horizontal pleiotropy identified only in CES (Table S15). Note that neither MV-MR nor MV-MR-Egger can provide a clear picture of the effect size of SBP across disease outcomes, with much larger effect estimates and a narrow difference between the largest (CAD) and the smallest (CES).

MR-BMA is not able to draw a clear distinction between the exposures selected and excluded, as shown in Table S16. This is exemplified in CAD where the exposures included depend on the multiple testing correction applied. When using a strict Bonferroni threshold, MR-BMA identifies, in decreasing order, SBP, SMOKING, T2D, and ApoB with 0.66 as the smallest mPPI, while with a more lenient FDR threshold HDL, LDL, and BMI are also included with the smallest mPPI of 0.29. Regarding the other outcomes, the order of importance of the exposures is different from ${MR}^{2}$ with remarkably larger model-averaged causal effect estimates (MACEs) for PAD and HF. MR-BMA suffers the same problems as MV-MR and MV-MR-Egger regarding the magnitude of the direct causal effect estimates of SBP on the outcomes, demonstrating that this problem affects all single-trait methods regardless of their implementation. Interestingly, MR-BMA does not identify T2D as an exposure for CES, which is instead detected by ${MR}^{2}$, MV-MR, and MV-MR-Egger, favoring in contrast BMI.

### Lipidomic risk factors for cardiovascular diseases

The first application example prioritizes ApoB as a shared exposure for three out of five CVDs. Moreover, conditional on ApoB, no other major lipoprotein-related trait has a likely causal role for any outcome. Our next step is to better understand molecular determinants of ApoB by considering ten ApoB-containing lipoprotein subfractions of different sizes, ranging from small-large to extra-extra-large, very-large-density lipoproteins, measured using nuclear magnetic resonance (NMR) spectroscopy.^58^ In particular, we are considering S.LDL.Ps, small large-density lipoprotein particles; M.LDL.Ps, medium large-density lipoprotein particles; L.LDL.Ps, large large-density lipoprotein particles; IDL.Ps, intermediate-density lipoprotein particles; XS.VLDL.Ps, extra-small very-large-density lipoprotein particles; S.VLDL.Ps, small very-large-density lipoprotein particles; M.VLDL.Ps, medium very-large-density lipoprotein particles; L.VLDL.Ps: large very-large-density lipoprotein particles; XL.VLDL.Ps: extra-large very-large-density lipoprotein particles; and XXL.VLDL.Ps, extra-extra-large very-large-density lipoprotein particles. Identification of specific subfractions to different CVD manifestations can help our understanding of the pathophysiology of the disease and provide insights into pathophysiology, molecular mechanisms, risk stratification, and treatment.^17^ However, performing MR using metabolites as exposures is a difficult task given the strong correlation and intricate dependence that exist between them (see Figures S13B and S13C).

For a description of the pre-processing, including IVW and instrument selection steps, we refer to supplemental information. Briefly, given the prior hypothesis of ApoB as the leading exposure for CVDs, we select genetic variants associated with ApoB in UK Biobank at genome-wide significance, resulting in n=148 IVs after clumping. This three-sample MR design is known to reduce bias due to winner’s curse bias.^59^ Results are obtained after removing outliers or high-leverage and influential observations using scaled CPO and fitting the proposed model on n=141 IVs (see Figure S15).

**Figure 6:**
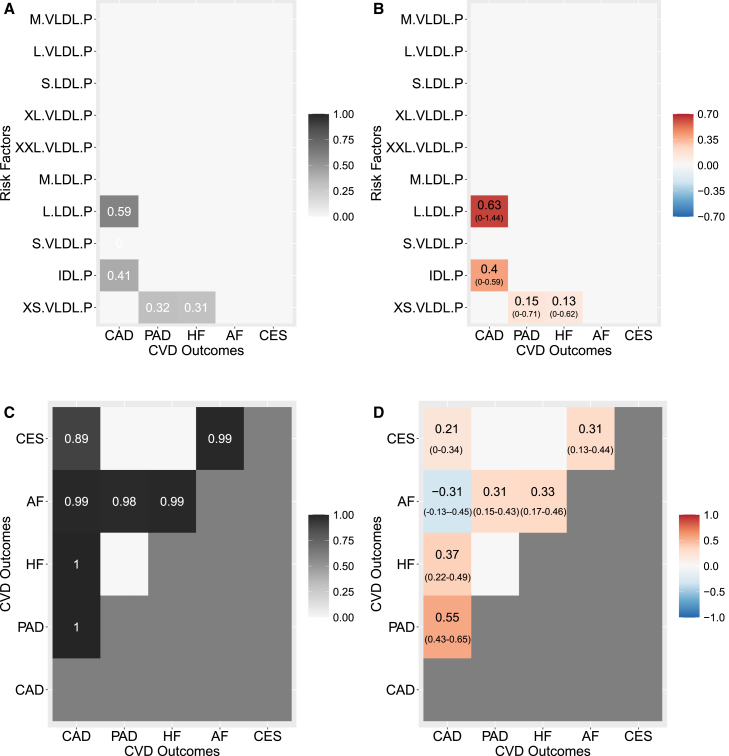
Results of the multi-response MR (${MR}^{2}$) model in application example 2 on molecular exposures for cardiovascular disease outcomes (CVDs) (A) Marginal posterior probability of inclusion (mPPI) of each exposure (y axis) against each outcome (x axis). Selected mPPIs for each exposure indicate whether an exposure is shared or distinct among multiple CVDs. (B) Posterior mean (95% credible interval) of the direct causal effect of each exposure (y axis) against each outcome (x axis). For clarity of presentation, mPPIs and estimated effect sizes for non-selected outcome-exposure pairs (mPPI <0.29 at 5% FDR) are not plotted. (C) Edge posterior probability of inclusion (ePPI) among outcomes. Only the upper triangular matrix is depicted. (D) Posterior mean (95% credible interval) of the partial correlations between outcomes. For clarity of presentation, ePPIs and partial correlations for non-selected outcomes pairs (ePPI <0.85 at 5% FDR) are not plotted. Only the upper triangular matrix is depicted.

When lipidomic risk factors are used for CVDs, results obtained by ${MR}^{2}$ are very sparse with few significant direct causal effects at 5% FDR (mPPI <0.29). Moreover, the separation between significant and non-significant causal associations is difficult (see Figure S16A). The proposed model identifies XS.VLDL.Ps as a shared exposure for both PAD and HF with mPPIs of 0.32 and 0.31, respectively, and a jPPI of 0.14 (Figure S16C) with direct causal effects of 0.15 and 0.13 (Figures 6A and 6B). Distinct exposures are detected for CAD, where smaller particle sizes, IDL.Ps and L.LDL.Ps, are prioritized with mPPIs of 0.41 and 0.59 and direct causal effects of 0.4 and 0.63, respectively. No subfractions are identified for the other disease outcomes.

In contrast with the previous application example, when dealing with molecular exposures, CIs are large, confirming the complexity of the analysis. For the strongest signal, L.LDL.P-CAD combination, the 95% CI ranges between 0 and 1.41. This is due to a combined effect of multi-collinearity (although the designed independent prior in Equation 13 protects against it; see appendix A) and the small number of genetic variants associated with ApoB in UK Biobank. Thus, while L.LDL.Ps (or IDL.Ps) could be a likely cause for CAD, the large CI suggests caution.

As expected, there is a substantial residual partial correlation in almost all combinations of summary-level outcomes, as depicted in Figures 6C and 6D, highlighting the existence of non-lipoprotein pleiotropic pathways between these traits not intercepted by the selected exposures (SBP, BMI, SMOKING, T2D pathways are missing by design) and, possibly, the effects of non-genetic factors on the responses.

When high levels of residual correlation are present and cannot be “explained away” or be “accounted for” by the exposures, the advantage of an MR multi-response model to reduce false positives is evident. For instance, MR-BMA (Table S19) identifies genetically predicted levels of L.VLDL.Ps to be associated also with AF, possibly because AF and CAD are correlated at the summary level (Figure S13). Similarly, the causal effect of S.VLDL.Ps on AF is likely a false positive by looking at its small MACE. MV-MR is not able to identify any exposure for any outcome that is significant after multiple testing correction (Table S17), even with a less conservative Benjamini-Hochberg FDR procedure, demonstrating that standard methodology is not adapted to handle highly correlated exposure data. Similar conclusions can be extended to MV-MR-Egger (Table S18) with no significant intercept identified for any outcome.

## Discussion

Here, we present ${MR}^{2}$, an MR design to analyze multiple related outcomes in a joint model and to define shared and distinct causes of related health outcomes. Based on a Bayesian copula regression model, ${MR}^{2}$ detects causal effects while estimating the residual correlation between MV-MR models for each outcome and vice versa. Thus, the proposed model makes the estimated causal effects robust to the residual correlation induced by shared pleiotropy. ${MR}^{2}$ is formulated on the summary level where the genetic variants used as IVs are considered observations. Residual correlation in the proposed model is consequently interpreted as the correlation between summary-level outcomes measured on the IVs not accounted for by the genetic associations with the exposures. We show, both theoretically and in a simulation example, how unmeasured shared pleiotropy induces residual correlation between summary-level genetic associations with the outcomes.

While residual diagnostics is an important strategy in summary-level univariable MR models to detect horizontal pleiotropy^51^^,^^60^ affecting one outcome, only multi-response MR models, like ${MR}^{2}$, can detect how much cross-variation between outcomes is unexplained after accounting for the exposures of interest. We show in an extensive simulation study that ${MR}^{2}$ has more power to detect true causal exposures, yields a better separation between causal and non-causal exposures, and improves the accuracy of the effect estimation over existing MV-MR methods that consider only one outcome at a time, such as MV-MR and MR-BMA. Moreover, ${MR}^{2}$ demonstrates more power when an exposure is causal for more than one outcome.

Thanks to the formulation as a joint multi-response model, ${MR}^{2}$ can distinguish between shared and distinct exposures for the disease, which is essential to define interventions that reduce the risk of more than one disease. We illustrate this in our application examples considering five CVDs. Multi-response models like ${MR}^{2}$ are a necessary contribution to better understanding the causes of multimorbidity. In particular, the discovery of shared and distinct causes of diseases may help define interventions with co-benefits, i.e., interventions that reduce the risk of more than one outcome. For instance, in our first application example, we have identified ApoB as likely causal exposure for CAD, PAD, and HF even when accounting for other lipoprotein measures including LDL cholesterol. These results of the applied analyses, considering effects of cardiometabolic risk factors on cardiovascular disease subtypes, are consistent with the existing epidemiological literature.^61^ Cholesterol is integral to the development of atherosclerosis and penetrates the arterial wall within those ApoB-containing lipoprotein particles that are small enough to pass to the *tunica intima* from the circulation; these particles include small VLDL, IDL, and LDL particles as well as lipoprotein(a). Taken together, this body of evidence suggests that the lipid content of the particles is secondary to ApoB.^55^ However, these results do not invalidate LDL cholesterol as a causal risk factor for the cardiovascular outcomes, as LDL particles also contain an ApoB molecule.^55^

While ApoB is an established exposure for CAD and PAD based on genetic evidence,^17^^,^^36^^,^^56^ we demonstrate that there is an independent effect of ApoB also on HF. This has the following two implications: for one, existing lipid-lowering therapies should be evaluated in terms of their impact on reducing ApoB, and second, future lipid-lowering therapies may be better tailored to reduce ApoB concentration. Moreover, this highlights the importance of including various disease endpoints in RCTs because the intervention may have benefits not just for the main disease of interest but also for other related diseases.

In our application examples, we discover residual correlation between CAD and PAD and between CAD and HF, as well as between the disease pathway HF-AF-CES, which was not accounted for by either common cardiometabolic disease exposure, including lipid traits, blood pressure, and obesity, or by lipid characteristics measured using NMR spectroscopy. Important contributors to the residual correlation when considering common cardiovascular disease exposures are molecular pathways, which are not accounted for when considering traits like ApoB or obesity. These pathways, such as inflammation or stress response, are highly polygenic with hundreds of independent regions in the genome associated with them. Another source of residual correlation between outcomes is the consequence of non-genetic factors that act exclusively on the responses. Another possible source of the observed residual correlation can be mediation, where genetic predisposition for one outcome may cause another, as may be the case for CAD and HF, as suggested, for example, by the Framingham Heart Study.^19^ Finally, sample overlap between the summary-level data of the outcomes may contribute to residual correlation, but it is not a necessary condition. As we show in extension of the simulation study (Figure S7) and we observed in the application examples, a residual correlation exists even if responses’ samples are non-overlapping. For example, we detect a substantial residual correlation between CAD, which was derived from the CARDIOGRAM Consortium and UK Biobank,^62^ and PAD, which was derived from the Million Veteran Program,^63^ where there is no overlap in the samples between these two outcomes. Sample overlap is a very common feature when working with summary-level data that are commonly built by integrating all available public data resources and, in particular, from large-scale biobanks.^30^

There are also limitations to our work. First of all, weak instrument bias is toward the null in univariable MR^64^ but can go toward any direction for MV-MR depending on the correlation between exposures.^15^ A necessary future extension of the approach is to make ${MR}^{2}$ more robust concerning weak instruments.^65^ Second, special care needs to be taken when selecting genetic variants as IVs. Importantly, the interpretation of any MR model is conditional on the IVs selected. For example, in our second data example, we have selected genetic variants based on their association with ApoB, which we identified in our primary analysis. Therefore, results need to be interpreted considering this choice and may differ when re-selecting, for example, based on LDL cholesterol. In practice, we recommend following the guidelines for reporting MR studies^66^^,^^67^ for further details. Similarly, dedicated domain knowledge is necessary when deciding which exposures to include and whether a trait is more suitable as exposure or as outcomes. For example, in the first application example, we include genetic liability to T2D as an exposure because diabetes is considered a risk factor for CAD in the NHS guidelines. Yet, being a case-control phenotype, it may be considered an outcome as well. Regarding the required number of IVs, ${MR}^{2}$ can only be used when there are enough IVs that allow the estimation of the main parameters of interest. As a rule of thumb, for each response, the number of IVs n should be greater than the number of exposures p. Moreover, n should be also large enough to permit the estimation of $\delta_{k}|Y$, k=1,…,q, and the lower triangular matrix of R|Y. A conservative choice would be n>q×(p+1)+q×(q−1)/2 with q the number of responses. However, given the sparseness assumption, for each response, the number of important direct causal effects is much smaller than p, and the number of non-zero cells in R|Y is smaller than q(q−1)/2. In addition, in real applications, the required number of IVs should not pose any problems when considering polygenic exposures because it is usually much larger than the whole number of exposures and responses.

The development of multi-response MR models on the individual level is another important future direction given the availability of large-scale biobanks with sufficient follow-up time allowing for the development of multiple disease outcomes. Such an analysis would let us study the presence of more than one disease in the same individual instead of analyzing the shared genetic basis of the outcomes as in our current work.

While our study has been motivated to detect common causal exposures for multi-morbid health conditions, ${MR}^{2}$ can be applied to any type of related outcomes. Potential application examples may include molecular biomarkers as outcomes; for example, a recent study has investigated the effect of sleep deprivation on the metabolome^68^ or of morning cortisol levels on inflammatory cytokines.^69^ Similarly, ${MR}^{2}$ can be used to define exposures for heritable imaging phenotypes measured on the brain,^70^ heart,^71^ or body composition.^72^

In conclusion, we present here ${MR}^{2}$, the first summary-level MR method that can model multiple outcomes jointly and account for residual correlation between the outcomes. Moreover, ${MR}^{2}$ can distinguish between shared or distinct causes of diseases, enhancing our understanding regarding which interventions can target more than one disease outcome.

## Data Availability

MR^2^ is freely available on https://github.com/lb664/MR2/. It includes examples that explain how to generate the simulated data and run the algorithm. Post-processing routines are also included.

## Appendix A

### IVW for multiple responses

When considering one outcome at a time, the univariable MR model in Equation 1 and MV-MR model in Equation 2 weight a genetic variant i=1,…,n, used as IV, by the standard error of the genetic association with the outcome $\text{se}\left(\beta_{Y_{i}}\right)$. These weights are also known as the “first-order weights.”^73^

In order to generalize the IVW for multiple outcomes, we note that the standard error of the genetic association with a binary trait Y of a genetic variant i, with minor allele frequency (MAF) ${\text{MAF}}_{i}$, is^74^

(Equation A1) $$\text{se}\left(\beta_{Y_{i}}\right)={\left\{N\times \mathrm{pr}\left(1-\mathrm{pr}\right)\times 2{\text{MAF}}_{i}\left(1-{\text{MAF}}_{i}\right)\right\}}^{-1/2}\text{,}$$

where pr is the percentage of cases and N is the total sample size. In Equation A1, the only quantity that depends on the genetic variant i is the MAF ${\text{MAF}}_{i}$, which may be considered as comparable when taking summary-level data from cohorts with similar ethnicity. All other parameters are specific for trait Y and do not depend on the genetic variant i and, consequently, are not relevant when weighting individually the genetic variants.

When considering q outcomes, we propose to use as weight $\omega_{i}$, i=1,…,n, the mean of the q standard errors of the genetic associations with each outcome, i.e., $\omega_{i}=\sum_{k=1}^{q}\text{se}\left(\beta_{Y_{ik}}\right)/q$, which essentially, under the hypothesis of cohorts with similar ethnicity, will result in weighting by the average of the inverse of the genetic variant standard errors defined by ${\left\{2{\text{MAF}}_{i}\left(1-{\text{MAF}}_{i}\right)\right\}}^{1/2}$.

### Bayesian multi-response MR

An alternative way to the SUR model in Equation 3 to model jointly the q-related outcomes is to describe their dependence through a copula function. A q-variate function $C\left(u_{1},\ldots ,u_{q}\right)$, where $C:{\left[0,1\right]}^{q}\to \left[0,1\right]$, is called a copula if it is a continuous distribution function and each marginal is a uniform distribution function on [0,1]. If $F_{Y_{1}}\left(\cdot \right),\ldots ,F_{Y_{q}}\left(\cdot \right)$ are the marginal cumulative density functions (cdfs) of the random variables $Y_{1},\ldots ,Y_{q}$, their joint cdf can be described through a specific copula function C as

$$F_{Y_{1},\ldots ,Y_{q}}\left(y_{1},\ldots ,y_{q}\right)=C\left\{F_{Y_{1}}\left(y_{1}\right),\ldots ,F_{Y_{q}}\left(y_{q}\right)\right\}\text{.}$$

The Gaussian copula C is described through the function

$$C\left(u_{1},\ldots ,u_{q};R\right)=\Phi_{q}\left\{\Phi^{-1}\left(u_{1}\right),\ldots ,\Phi^{-1}\left(u_{q}\right);R\right\}\text{,}$$

where $\Phi_{q}\left(\cdot ;R\right)$ is the cdf of a q-variate Gaussian distribution with zero mean vector and correlation matrix R and $\Phi^{-1}\left(\cdot \right)$ is the inverse of the univariate standard Gaussian cdf. If all the marginal distributions are continuous, the matrix R can be interpreted as the correlation matrix of the elements of Y and zeros in its inverse imply the conditional independence between the corresponding elements of the responses.

The GCR model is described by the transformation

(Equation A2) $$y_{ik}=h_{ik}^{-1}\left(z_{ik}\right),\;Z_{i}\overset{\text{iid}}{∼}N_{q}\left(0,R\right)\text{,}$$

where $z_{i1},\ldots ,z_{iq}$ are realizations from $Z_{i}$ and $h_{ik}\left(\cdot \right)=\Phi^{-1}\left\{F_{k}\left(\cdot ;x_{ik},\theta_{k},\sigma_{k}^{2}\right)\right\}$ for each i=1,…,n and k=1,…,q, $x_{ik}$ is the p-dimensional vector of predictors for the ith sample and the kth response, and $\theta_{k}={\left(\theta_{k1},\ldots ,\theta_{kp}\right)}^{T}$ is a p-dimensional vector of regression coefficients.

A detailed discussion of the Bayesian formulation of the GCR model in Equation A2 with multiple responses of any type is presented in ref. ^40^. In the following, we summarize the main aspects of the proposed multi-response MV-MR model when all margins are univariate Gaussian. Details of the MCMC algorithm are presented in supplemental information.

The SUR model is a special case of Equation A2 when all margins are univariate Gaussian with mean $x_{ik}\theta_{k}$ and variance $\sigma_{k}^{2}$ and R is the correlation matrix. Thus, Equations 3 and 4 can be written in terms of a GCR model as

(Equation A3) $$\beta_{Y_{ik}}=h_{ik}^{-1}\left(z_{ik}\right),\;Z_{i}\overset{\text{iid}}{∼}N_{q}\left(0,R\right)\text{,}$$

$with\;h_{ik}\left(\cdot \right)=\Phi^{-1}\left\{\Phi (\cdot ;\beta_{X_{i}},\theta_{k},\delta_{k}^{2}\right\}\;or,equivalently\text{,}$

(Equation A4) $$\beta_{Y_{ik}}=\beta_{X_{i}}\theta_{k}+\delta_{k}z_{ik}$$

for each i=1,…,n and k=1,…,q and with R as in Equation 4.

### Priors specification

The selection of important exposures is achieved by utilizing a binary latent vector $\gamma_{k}=\left(\gamma_{k1},\ldots ,\gamma_{kp}\right)$, k=1,…,q, where $\gamma_{kj}$ is 1 if, for the jth exposure and the kth response, the effect estimate is different from zero and 0 otherwise. Specifically, we assume that for each k, the effects in Equation A4 are distributed as

(Equation A5) $$\theta_{kj}|\gamma_{kj}∼\left(1-\gamma_{kj}\right)\delta_{0}+\gamma_{kj}N\left(0,v\right),\;j=1,\ldots ,p\text{,}$$

where $\delta_{0}$ denotes a point mass at zero and v is a fixed value. Sparsity for the selection of important exposures for the kth response is enforced by specifying the hierarchical structure

(Equation A6) $$\gamma_{kj}|\pi_{k}\overset{\text{iid}}{∼}\text{Ber}\left(\pi_{k}\right),\;j=1,\ldots ,p$$

$$\pi_{k}∼\text{Beta}\left(a_{k},b_{k}\right)\text{.}$$

Integrating out $\pi_{k}$ in Equation A6, it is readily shown that marginally

(Equation A7) $$p\left(\gamma_{k}\right)=\frac{B\left(a_{k}+\left|\gamma_{k}\right|,b_{k}+p-\left|\gamma_{k}\right|\right)}{B\left(a_{k},b_{k}\right)}\text{,}$$

where B(·,·) is the beta function and $\left|\gamma_{k}\right|=\sum_{j=1}^{p}\gamma_{kj}$. The hyperparameters $a_{k}$ and $b_{k}$ can be chosen using prior information about the average number of important exposures associated with the kth response and its variance.

Because the GCR model in Equation A3 is defined through the correlation matrix R, we need first to expand R into a covariance matrix. We define the transformation W=ZΔ, where Z is the (n×q)-dimensional matrix of the Gaussian latent variables obtained by inverting Equation A4

$$z_{ik}=\frac{\beta_{Y_{ik}}-\beta_{X_{i}}\theta_{k}}{\delta_{k}}$$

and Δ is a q×q diagonal matrix with elements $\delta_{k}$, k=1,…,q. Then,

(Equation A8) $$\text{vec}\left(W\right)∼N_{nq}\left(0,I_{n}⊗\Sigma \right)\text{,}$$

where Σ=ΔRΔ and $I_{n}$ is a diagonal matrix of dimension n that encodes the independence assumption among the genetic variants G (achieved by pruning). In this framework, the correlation matrix can be obtained by the inverse transformation $R=\Delta^{-1}\Sigma \Delta^{-1}$. We utilize the theory of decomposable graphical models^42^ to perform a conjugate analysis of the covariance structure of the model because the hyper-inverse Wishart distribution is a conjugate prior distribution for the covariance matrix Σ with respect to the symmetric adjacency matrix G of a decomposable graph G.

Finally, we assign the following prior structure on the symmetric adjacency matrix G and Δ. Let $g_{\ell }$, ℓ=1,…,q(q−1)/2, be the binary indicator for the presence of the ℓth off-diagonal edge in the lower triangular part of the symmetric adjacency matrix G of the decomposable graph G. To select important elements of the inverse covariance matrix, we assume the sparsity prior

(Equation A9) $$g_{\ell }\overset{\text{iid}}{∼}\text{Ber}\left(\pi_{G}\right),\;\pi_{G}∼\text{Unif}\left(0,1\right),\;\ell =1,\ldots ,q\left(q-1\right)/2\text{.}$$

We denote by p(G) the induced marginal prior on the symmetric adjacency matrix and define the joint distribution of Δ, R, and G as p(Δ,R,G)=p(Δ|R)p(R|G)p(G), where

(Equation A10) $$\delta_{k}|r^{kk}∼\text{IGam}\left\{\left(q+1\right)/2,{\left(R^{-1}\right)}_{kk}/2\right\},\;k=1,\ldots ,q\text{,}$$

with ${\left(R^{-1}\right)}_{kk}$ the kth diagonal element of $R^{-1}$.^75^

### Outliers, high-leverage and influential observations

Detection of outliers is performed by using the CPO^52^ to detect genetic variants $G_{i}$, i=1,…,n, as outliers in the multi-response MR model. The CPO is also known as the leave-one-out cross-validation predictive density,^76^ which, for the multi-response MR model in Equation A3, is defined as

$$p\left(\beta_{Y_{i}}|\beta_{Y_{-i}},\beta_{X}\right)=\int p\left(\beta_{Y_{i}}|\beta_{X_{i}},\Psi \right)p\left(\Psi |\beta_{Y_{-i}},\beta_{X_{-i}}\right)d\Psi =E_{\Psi |\beta_{Y_{-i}},\beta_{X_{-i}}}{\left\{p{\left(\beta_{Y_{i}}|\beta_{X_{i}},\Psi \right)}^{-1}\right\}}^{-1}\text{,}$$

where ($\beta_{Y_{i}}$, $\beta_{X_{i}}$) and ($\beta_{Y_{-i}}$, $\beta_{X_{-i}}$) are the IVW-standardized summary-level associations with the genetic variant $G_{i}$ and all the remaining genetic variants, respectively, and Ψ is the whole parameter space Ψ={Γ,Θ,G,Δ,R} with $\Gamma ={\left(\gamma_{1},\ldots ,\gamma_{q}\right)}^{T}$ and $\Theta ={\left(\theta_{1},\ldots ,\theta_{q}\right)}^{T}$.

The CPO describes the posterior probability of observing the q-dimensional vector of values of $\beta_{Y_{i}}$ when the model is fitted to all data except $\beta_{Y_{i}}$, with a larger value implying a better fit of the model for $\beta_{Y_{i}}$ and a very low CPO value suggesting that the summary-level associations with the genetic variant $G_{i}$ are either an outlier or a high-leverage and influential observation.

The CPO can be calculated by using the output of the MCMC algorithm. Let T be the number of recorded MCMC iterations. By considering the inverse likelihood across T iterations, the estimated CPO for each genetic variant $G_{i}$, i=1,…,n, is

(Equation A11) $${\text{CPO}}_{i}=\frac{T}{\sum_{t=1}^{T}p{\left(\beta_{Y_{i}}|\beta_{X_{i}}\Psi^{\left(t\right)}\right)}^{-1}}\text{,}$$

where $\Psi^{\left(t\right)}$ is tth posterior sample of Ψ obtained from the MCMC algorithm. Thus, the Monte Carlo estimate of the CPO is obtained without actually omitting $\left(\beta_{Y_{i}},\beta_{X_{i}}\right)$ from the estimation of the posterior distribution of Ψ and is provided by the harmonic mean of the likelihood.^77^

Regarding the threshold for the detection of outliers or high-leverage and influential observations, log-inverse CPOs larger than 40 can be considered as possible outliers and higher than 70 as extreme values.^78^ Congdon^79^ recommends scaling CPOs by dividing each one by its individual maximum (likelihood) recorded across the MCMC iterations and considering observations with scaled CPOs under 0.01 to be outliers or high-leverage and influential observations. If few CPOs are less than 0.01, the model is considered to fit adequately.

### FDR

The mPPI, which measures the strength of direct causal association between each exposure-response combination, and the ePPI, which describes the strength of the residual conditional correlation between each responses’ pair, are utilized to select significant exposure-response combinations and important pairs of dependent responses.

Let

(Equation A12) $${\text{mPPI}}_{h}∼\pi_{0}\text{Beta}\left(a_{0},b_{0}\right)+\pi_{1}\text{Beta}\left(a_{1},b_{1}\right),\;h=1,\ldots ,pq\text{,}$$

be the two-component mixture model that classifies the mPPI for each exposure-response combination into the null $\left(H_{0}\right)$ or the alternative distribution $\left(H_{1}\right)$ parameterized as beta densities with parameters $\left(a_{0},b_{0}\right)$ and $\left(a_{1},b_{1}\right)$, respectively, with weights $\pi_{0},\pi_{1}\geq 0$ and $\pi_{0}+\pi_{1}=1$.

In the real application examples, the parameters of the mixture model in Equation A12 are estimated using the expectation-maximization algorithm.^80^ The posterior probability of allocation to the alternative hypothesis of each mPPI is

$$P\left({\text{mPPI}}_{h}|H_{1}\right)=\frac{\pi_{1}\text{Beta}\left({\text{mPPI}}_{h};a_{1},b_{1}\right)}{\pi_{0}\text{Beta}\left({\text{mPPI}}_{h};a_{0},b_{0}\right)+\pi_{1}\text{Beta}\left({\text{mPPI}}_{h};a_{1},b_{1}\right)}=\pi_{h}^{H_{1}}\text{.}$$

Finally, let $\left\{\pi_{\left(h\right)}^{H_{1}}\right\}$, h=1,…,pq, the sequence in decreasing order of the posterior probabilities of allocation to the alternative hypothesis of the mPPIs for each exposure-response combination. Significant mPPIs are chosen such that

$${\text{arg max}}_{h}\sum_{h}\pi_{\left(h\right)}^{H_{1}}\leq \text{FDR,}$$

where FDR is the designed level.

### Simulation study

Our simulation study is formulated in a summary-level MR design, where N=100,000 independent individuals are simulated, of which half are used to compute the genetic associations with the exposures and half to compute the genetic associations with the outcomes. In the following, we indicate with the subscripts “X” and “Y” the relevant quantities that are associated with the exposures and the responses, respectively. For instance, $N_{X}$ and $N_{Y}$ indicate the sample size used in the genetic associations with the exposures and the outcomes, respectively. Moreover, we assume that the quantitative outcomes $Y_{k}$, k=1,…,q are measured on the same individuals $N_{Y}$.

In all simulation scenarios, we consider p=15 exposures, q=5 outcomes, and n=100 independent genetic variants as IVs. Genotypes for the ith genetic variant and each individual l are simulated independently according to a binomial distribution with MAF equal to 0.05, i.e., $G_{li}\overset{\text{iid}}{∼}\text{Bin}\left(2,0.05\right)$, l=1,…,N, and i=1,…,n. Without loss of generality, the resulting matrix of genotypes G is split into two equally sized groups, $G_{X}$ and $G_{Y}$, of dimension $N_{X}\times n$ and $N_{Y}\times n$ with $N_{X}=N_{Y}=N/2$, respectively. All genetic variants are considered with equal weights, and thus no IVW is needed given that the same MAF at 5% is used to simulate the genotypes.

Overall, the data-generation process consists of two steps. In the first step, the raw data for the exposures X and the responses Y are simulated. Then, in the second step, summary-level data are obtained as the regression coefficients $\beta_{X_{ij}}$, i=1,…,n, j=1,…,p, from a univariable regression in which the exposure $X_{j}$ is regressed on the genetic variant $G_{i}$ in sample one and the regression coefficients $\beta_{Y_{ik}}$, i=1,…,n, k=1,…,q from a univariable regression in which the outcome $Y_{k}$ is regressed on the genetic variant $G_{i}$ in sample two. In the following, we detail each step and how we simulate the quantities involved.

For the first stage of the simulation study, the jth exposure, j=1,…,p, is generated by

(Equation A13) $$X_{j}=G_{X}\beta_{X_{j}}+U_{X}\theta_{X}^{U}+\varepsilon_{j}\text{,}$$

where $G_{X}$ and $U_{X}$ are the matrix of genotypes of the n IVs and the confounder U measured on the same $N_{X}=50,000$ individuals, respectively, and $\varepsilon_{j}∼N_{N_{X}}\left(0,H_{X_{j}}I_{N_{X}}\right)$. $H_{X_{j}}$ is the jth diagonal element of the (p×p)-dimensional matrix $H_{X}=\frac{1-h_{X}}{h_{X}}{\left(G_{X}\beta_{X}+U_{X}\theta_{X}^{U}1_{p}^{T}\right)}^{T}\left(G_{X}\beta_{X}+U_{X}\theta_{X}^{U}1_{p}^{T}\right)$ with $1_{p}$ a p-dimensional vector of ones, $\beta_{X}=\left(\beta_{X_{1}},\ldots ,\beta_{X_{p}}\right)$ and $h_{X}$ the desired level of heritability, or how much variation G can explain in X, fixed at 10% in all simulated scenarios.

The confounder U is drawn from a multivariate standard Gaussian distribution, i.e., $U∼N_{N}\left(0,I_{N}\right)$ and then split into two equally sized vectors $U_{X}$ and $U_{Y}$ with effect $\theta_{X}^{U}$ impacting all the exposures X and $\theta_{Y}^{U}$ effecting all the outcomes Y. Their value is fixed at $\theta_{X}^{U}=2$ and $\theta_{Y}^{U}=1$ in main results. Instrument strength depends on the degree of confounding, where the average F-statistic is on average around 45 and 35 for the weakest degrees of confounding $\theta_{X}^{U}=\theta_{Y}^{U}=1$ and for the strongest confounding effects $\theta_{X}^{U}=\theta_{Y}^{U}=2$, respectively.

The effects $\beta_{X_{j}}$ of the genetic variants on the jth exposure, j=1,…,p, across n IVs are drawn independently from a multivariate Gaussian distribution, i.e., $\beta_{X_{j}}∼N_{n}\left(0,c_{X}I_{n}\right)$, where $c_{X}$ is a scaling factor related to the sought range of the simulated $\beta_{X}$ and defined as the difference between the maximum and minimum desired value of the effect size divided by four. The range of the simulated $\beta_{X}$ is chosen between −2 and 2 with 95% of the simulated values within this interval. It also implies a unit variance for the summary-level genetic associations with the exposures.

We prepare different scenarios in which the exposures are either independent or correlated by inducing a dependence between exposures by multiplying the (n×p)-dimensional matrix $\beta_{X}$ with the (p×p)-dimensional matrix $D_{X}$, where $D_{X}$ is the Cholesky decomposition of the Toeplitz matrix $R_{X}$ with $r_{X}^{\left|j-j^{\prime }\right|}$ for the $\left(j,j^{\prime }\right)$ element of $R_{X}$, $j=j^{\prime }=1,\ldots ,p$. The matrix $R_{X}$ implies a tridiagonal sparse inverse correlation matrix $R_{X}^{-1}$. We use different levels of correlation between the exposures, ranging from independence to strong correlation, i.e., $r_{X}=\left\{0,0.2,0.4,0.6,0.8\right\}$, where $r_{X}=0.6$ represents a medium correlation strength.

For the second stage of the simulation study, according to Equation 5, an outcome k, k=1,…,q, is generated on another independent set of $N_{Y}=50,000$ individuals

(Equation A14) $$Y_{k}=X\theta_{k}+A\theta^{A}+U\theta_{Y}^{U}+\varepsilon_{k}\text{,}$$

where $\theta_{k}$ is p-dimensional vector that contains the direct causal effects of the exposures on the kth outcome, X Z X is the $\left(N_{X}\times p\right)$-dimensional matrix of exposures simulated using Equation A13, $\theta^{A}$ and $\theta_{Y}^{U}$ are the effects of the unmeasured pleiotropic pathway A and the unmeasured confounder U on the same outcome, respectively, and $\varepsilon_{k}∼N_{N_{Y}}\left(0,H_{Y_{k}}I_{N_{Y}}\right)$, where $H_{Y_{k}}=\frac{1-h_{Y}}{h_{Y}}\left\{{\left(X\theta_{k}+A\theta^{A}+U\theta_{Y}^{U}\right)}^{T}\left(X\theta_{k}+A\theta^{A}+U\theta_{Y}^{U}\right)\right\}$ with $h_{Y}$ the desired level of the proportion of variance explained, fixed at 25% for all outcomes in all simulated scenarios, except for scenario I—null setting. We also simulate different second-level data with various values of the pleiotropic effect on the outcomes $\theta^{A}=\left\{0.25,0.50,0.75,1.00,1.50,2.00\right\}$.

In the simulation V—dependence scenario, we induce the correlation between the outcomes by directly controlling the responses’ error correlation. Specifically, we simulate the errors $\varepsilon =\left(\varepsilon_{1},\ldots ,\varepsilon_{q}\right)$ for all k in Equation A14 from a multivariate Gaussian distribution with mean vector 0 and correlation structure based on the Toeplitz matrix $R_{Y}$ with $r_{Y}^{\left|k-k^{\prime }\right|}$ for the $\left(k,k^{\prime }\right)$ element of $R_{Y}$, $k=k^{\prime }=1,\ldots ,q$ and the level of $r_{Y}$ chosen in the set $r_{Y}=\left\{0,0.2,0.4,0.6,0.8\right\}$ such that it induces correlation between the responses’ errors

$$\text{vec}\left(\varepsilon \right)∼N_{N_{Y}q}\left(0,H_{N_{Y}}⊗R_{Y}\right)\text{,}$$

where $H_{N_{Y}}=\frac{h_{Y}}{1-h_{Y}}diag\left\{\left(X\theta +U\theta_{Y}^{U}1_{p}^{T}\right){\left(X\theta_{k}+U\theta_{Y}^{U}1_{p}^{T}\right)}^{T}\right\}$ is an $N_{Y}\times N_{Y}$ diagonal matrix with $\theta ={\left(\theta_{1},\ldots ,\theta_{p}\right)}^{T}$ the vector of the direct effect estimates.

To evaluate the impact in Equation A14 of the unmeasured shared pleiotropy on the outcomes, we generate the $N_{Y}$-dimensional vector A as follows,

(Equation A15) $$A=G_{Y}\beta_{A}+\varepsilon_{A}\text{,}$$

where $\beta_{A}$ is the n-dimensional vector of genetic associations with the unmeasured pleiotropic pathway A drawn from a uniform distribution defined on a range between −2 and 2, i.e., $\beta_{A_{i}}\overset{\text{iid}}{∼}\text{Unif}\left(-2,2\right)$, 1,…,n, mimicking shared ”undirected” pleiotropy, i.e., $A_{l}≷0$, l=1,…,N. Similarly to the simulation of the exposures, the error term in Equation A15 is simulated controlling the level of variance explained $\varepsilon_{A}∼N_{N_{Y}}\left(0,H_{A}\right)$, where $H_{A}=\frac{1-h_{X}}{h_{X}}\text{diag}\left\{\left(G_{Y}\beta_{A}\right){\left(G_{Y}\beta_{A}\right)}^{T}\right\}$. Additionally, we consider shared “directed” pleiotropy, where the effect direction on the pleiotropic pathway A is drawn from a uniform distribution defined only on a positive range between 0 and 2, i.e., $\beta_{A_{i}}\overset{\text{iid}}{∼}\text{Unif}\left(0,2\right)$. In case of non-overlapping samples in the genetic associations with the outcomes, the simulation strategy for $A_{k}$, k=1,…,q, follows the same principles we have described earlier to induce correlation between the exposures.

Finally, the direct causal effects $\theta_{k}$, k=1,…,q, are drawn independently from a multivariate Gaussian distribution, i.e., $\theta_{k}∼N_{p}\left(0,c_{Y}I_{p}\right)$, where $c_{Y}$ is a scaling factor related to the sought range of the simulated $\theta_{k}$ and defined as the difference between the maximum and minimum desired value of the direct causal effects divided by four. For the direct causal effects, the desired interval lies between −2 and 2, implying unit variance for the direct causal effects. Note that with this choice, 70% of all direct causal effects are simulated between (−1,1).

In the simulation study, we include one “null” setting with no direct causal effects. All other settings consider a (q×p)-dimensional sparse matrix of direct causal effects $\Theta ={\left(\theta_{1},\ldots ,\theta_{q}\right)}^{T}$, where 30% cells of the matrix are non-zero and where several exposures are either shared or distinct for the outcomes. Specifically, we select at random the same proportion of cells in the matrix Θ and assign them the simulated values, while the other cells are set to zero. On average, the most likely configuration is with a distinct exposure (37.3%) followed by a shared exposure between two outcomes (31.5%), no direct causal effects (16.6%), and an exposure shared by more than two outcomes (14.5%) (see Table S12 for an overview). On the other hand, the most likely number of associated exposures for each outcome is four (24.4%), five (22.5%), three (18%), six (14.5%), two (8.5%), and seven (6.6%).

After creating data on the individual level, we compute the corresponding summary-level statistics from the two independent groups of individuals. The input data for the simulation are the summary-level statistics $\beta_{X}$, an (n×p)-dimensional matrix, and $\beta_{Y}$, a (n×q)-dimensional matrix, derived from a univariable linear regression model where each genetic variant $G_{i}$, i=1,…,n, is regressed against exposure $X_{j}$, j=1,…,p, or outcome $Y_{k}$, k=1,…,q, at a time. Additionally, we monitored the average F-statistic in the first stage to control for instrument strength. All genetic variants are considered with equal weights, and thus no IVW is needed given the same MAF at 5%.

All simulation runs are repeated 50 times, each of which is initialized with a different random seed.

## Supplemental information

Document S1. Figures S1–S17 and Tables S1–S12

Document S2. Article plus supplemental information
