# Supplementary material for: Multi-response Mendelian randomization: Identification of shared and distinct exposures for multimorbidity and multiple related disease outcomes
Source: Am J Hum Genet. 2023 Jul 6;110(7):1177–99. doi: 10.1016/j.ajhg.2023.06.005 (PMC10357504; doi:10.1016/j.ajhg.2023.06.005)
Supplement: Document S1. Figures S1–S17 and Tables S1–S12 [file mmc1.pdf]

The American Journal of Human Genetics, Volume *110*

Supplemental information

Multi-response Mendelian randomization: Identification of shared and distinct exposures for multimorbidity and multiple related disease outcomes

Verena Zuber, Alex Lewin, Michael G. Levin, Alexander Haglund, Soumaya Ben-Aicha, Costanza Emanueli, Scott Damrauer, Stephen Burgess, Dipender Gill, and Leonardo Bottolo

S1 Multi-response MR residual correlation

In eq. (6) of the main paper, $\tilde{\varepsilon}_k$, $k = 1, \ldots, q$, is normally distributed with mean $\mathbb{E}(\tilde{\varepsilon}_k) = 0$ and variance

$$\begin{aligned} \mathbb{V}(\tilde{\varepsilon}_k) &= (G^T G)^{-1} G^T V(\varepsilon_k) G (G G^T)^{-1} \\ &= (G^T G)^{-1} G^T \delta_k^2 I_N G (G^T G)^{-1} \\ &= \delta_k^2 (G^T G)^{-1}. \end{aligned} \tag{S1}$$

In eq. (7), the summary-level error ϵ_k, $k = 1, \ldots, q$, is normally distributed with mean

$$\begin{aligned} \mathbb{E}(\beta_A \theta^A + \tilde{\varepsilon}_k) &= \mathbb{E}(\beta_A \theta^A) + \mathbb{E}(\tilde{\varepsilon}_k) \\ &= \theta^A (G^T G)^{-1} G^T \, \mathbb{E}(A) + (G^T G)^{-1} G^T \, \mathbb{E}(\varepsilon_k) \\ &= \theta^A \mu_A (G^T G)^{-1} G^T 1_N, \end{aligned}$$

where $\mathbb{E}(A) = \mu_A 1_N$ with 1_N a N-dimensional vector of ones.
Assuming A and ε_k independent in eq. (5) and using eq. (S1), then

$$\begin{aligned} \mathbb{V}(\beta_A \theta^A + \tilde{\varepsilon}_k) &= \mathbb{V}(\beta_A \theta^A) + \mathbb{V}(\tilde{\varepsilon}_k) \\ &= (\theta^A)^2 \, \mathbb{V}(\beta_A) + \delta_k^2 (G^T G)^{-1} \\ &= (\theta^A)^2 \sigma_A^2 (G^T G)^{-1} + \delta_k^2 (G^T G)^{-1} \\ &= \{(\theta^A)^2 \sigma_A^2 + \delta_k^2\} (G^T G)^{-1} \end{aligned} \tag{S2}$$

with $\mathbb{V}(\beta_A) = (G^T G)^{-1} G^T \sigma_A^2 I_N G (G^T G)^{-1} = \sigma_A^2 (G^T G)^{-1}$.
Assuming that the quantitative outcomes Y_k, $k = 1, .., q$, are measured on the same N individuals, the correlation between the residuals of the summary-level outcomes k and k', $k \neq k'$, is

$$\begin{aligned} \rho(\epsilon_k, \epsilon_{k'}) &= \mathbb{V}(\beta_A \theta^A + \tilde{\varepsilon}_k)^{-1/2} \, \mathrm{Cov}(\beta_A \theta^A + \tilde{\varepsilon}_k, \beta_A \theta^A + \tilde{\varepsilon}_{k'}) \times \\ &\quad \mathbb{V}(\beta_A \theta^A + \tilde{\varepsilon}_{k'})^{-1/2} \\ &= \{(\theta^A)^2 \sigma_A^2 + \delta_k^2\}^{-1/2} (G^T G)^{1/2} \times \\ &\quad \{\mathbb{V}(\beta_A \theta^A) + \mathrm{Cov}(\tilde{\varepsilon}_k, \tilde{\varepsilon}_{k'})\} \times \\ &\quad \{(\theta^A)^2 \sigma_A^2 + \delta_{k'}^2\}^{-1/2} (G^T G)^{1/2} \\ &= \{(\theta^A)^2 \sigma_A^2 + \delta_k^2\}^{-1/2} (G^T G)^{1/2} \times \\ &\quad \{(\theta^A)^2 \sigma_A^2 (G^T G)^{-1} + \sigma_{kk'} (G^T G)^{-1}\} \times \\ &\quad \{(\theta^A)^2 \sigma_A^2 + \delta_{k'}^2\}^{-1/2} (G^T G)^{1/2} \\ &= \frac{(\theta^A)^2 \sigma_A^2 + \sigma_{kk'}}{\{(\theta^A)^2 \sigma_A^2 + \delta_k^2\}^{1/2} \{(\theta^A)^2 \sigma_A^2 + \delta_{k'}^2\}^{1/2}} I_n \end{aligned} \tag{S3}$$

using (S2) and the fact that

$$\begin{aligned}\mathrm{Cov}(\tilde{\varepsilon}_k, \tilde{\varepsilon}_{k'}) &= (G^T G)^{-1} G^T \sigma_{kk'} I_N G (G G^T)^{-1} \\ &= \sigma_{kk'} (G^T G)^{-1} G^T G (G G^T)^{-1} \\ &= \sigma_{kk'} (G^T G)^{-1},\end{aligned}$$

where $\sigma_{kk'} = \mathrm{Cov}(\varepsilon_k, \varepsilon_{k'})$ is the covariance between individual-level responses' errors which is assumed constant across all individuals.

Considering all outcomes and all IVs, the summary-level covariance matrix can be written in compact form as $\rho(\epsilon) = I_n \otimes R$ with $\epsilon = (\epsilon_1, \ldots, \epsilon_q)$ and $R_{kk'}$ as in eq. (S3) for any of the n IVs.

In case of non-overlapping samples in the genetic associations with the outcomes, we assume that responses k and k', $k \neq k'$, are measured in two distinct groups of individuals k and k', respectively, and no individuals are present in both groups. Let G_{Y_k} be the $(n \times N_{Y_k})$-dimensional matrix of genotypes for the group of individuals k for outcome k and, similarly, $G_{Y_{k'}}$ the $(n \times N_{Y_{k'}})$-dimensional matrix of genotypes for group k' for outcome k'. Without loss of generality, let $N_Y = N_{Y_k} = N_{Y_{k'}}$. Then,

$$\begin{aligned}\mathrm{Cov}(\beta_{A_k}\theta^A + \tilde{\varepsilon}_k, \beta_{A_{k'}}\theta^A + \tilde{\varepsilon}_{k'}) &= (\theta^A)^2 \mathrm{Cov}(\beta_{A_k}, \beta_{A_{k'}}) + \mathrm{Cov}(\tilde{\varepsilon}_k, \tilde{\varepsilon}_{k'}) \\ &= \{(\theta^A)^2 G^T_{Y_k} \mathrm{Cov}(A_k, A_{k'}) G_{Y_{k'}} + \qquad \text{(S4)} \\ &\quad \sigma_{kk'} G^T_{Y_k} G_{Y_{k'}}\} (G^T_{Y_k} G_{Y_k})^{-1} (G^T_{Y_{k'}} G_{Y_{k'}})^{-1}.\end{aligned}$$

Moreover, $\mathbb{V}(\beta_{A_k}\theta^A + \tilde{\varepsilon}_k) = \{(\theta^A)^2 \sigma^2_{A_k} + \delta^2_k I_n\}(G^T_{Y_k} G_{Y_k})^{-1}$ and similarly for $\mathbb{V}(\beta_{A_{k'}}\theta^A + \tilde{\varepsilon}_{k'})$. Trivially, in eq. (S4), $\sigma_{kk'} = 0$ for non-overlapping samples and it is non-zero if $\mathrm{Cov}(A_k, A_{k'}) \neq 0$ for all $\ell_k = \ell_{k'} = 1, \ldots, N_Y$. It is sufficient to assume that

$$((\beta_{A_k})_i, (\beta_{A_{k'}})_i)^T \overset{\text{iid}}{\sim} \mathrm{N}_2(0, cR^A), \quad R^A = \begin{bmatrix} 1 & \rho^A \\ \rho^A & 1 \end{bmatrix} \qquad \text{(S5)}$$

to impose that $\mathrm{Cov}(A_k, A_{k'}) \neq 0$. Note that if $G_{Y_k} = G_{Y_{k'}}$, $\rho^A = 1$ and $c = \sigma^2_A$, results coincide with eq. (S3).

S2 Details of the MCMC implementation

The MCMC algorithm for the selection of important predictors in regression models with multiple responses of any type is presented in [1]. Here, we describe the main steps of the MCMC algorithm for the proposed multi-response multivariable Mendelian randomization MR^2 model as well as the main advantages of the designed proposal distribution for the non-zero direct

causal effects θ_k^* over standard implementations of Bayesian inference for the SUR model. In the following, the symbol "*" indicates the proposed new value used in the Metropolis-Hastings (M-H) acceptance ratio.

Given a candidate value of the latent binary vector γ_k^*, the proposal density for θ_k^* is

$$q(\theta_k^*|\gamma_k^*, \beta_{Y_k}, Z_{_k}, \Delta, R) \propto \mathcal{N}_{|\gamma_k^*|}(\theta_k^*; m_{\gamma_k^*}, V_{\gamma_k^*}), \tag{S6}$$

where

- $V_{\gamma_k^*}^{-1} = \delta_{k|-k}^{-2}(\beta_X^T)_{\gamma_k^*}(\beta_X)_{\gamma_k^*} + v^{-1}I_{|\gamma_k^*|}$ is the $(|\gamma_k^*| \times |\gamma_k^*|)$-dimensional proposal precision matrix,
- $m_{\gamma_k^*} = V_{\gamma_k^*}(\beta_X^T)_{\gamma_k^*}(\beta_{Y_k} - \delta_k \mu_{k|-k})/\delta_{k|-k}^2$ is the $|\gamma_k^*|$-dimensional proposal mean,
- $\delta_{k|-k}^2$ is the conditional variance of $\beta_{Y_k}|Z_{_{-k}}$, *i.e.*, $\delta_{k|-k}^2 = \delta_k^2/(R^{-1})_{kk}$,
- $\mu_{k|-k}$ is the n-dimensional vector of conditional means of $Z_k|Z_{_{-k}}$, *i.e.*, $\mu_{k|-k} = Z_{-k}R_{-k,-k}^{-1}R_{-k,k}$

and where the subscript "$-k$" implies that the corresponding matrix (or vector) consists of all the elements except those related to the kth response.

The interpretation of the above quantities sheds light on the benefits of the proposed model. $V_{\gamma_k^*}^{-1}$ resembles the standard Gibbs sampling update of the regression coefficients precision matrix in the linear model. However, in $V_{\gamma_k^*}^{-1}$, we use the conditional variance $\delta_{k|-k}^2$ of $\beta_{Y_k}|Z_{_{-k}}$ instead of the marginal variance δ_k^2. In $m_{\gamma_k^*}$, since $\mathbb{E}(\beta_{Y_k}) = (\beta_X)_{\gamma_k^*}(\theta_k)_{\gamma_k^*} + \delta_k Z_{-k}R_{-k,-k}^{-1}R_{-k,k} = (\beta_X)_{\gamma_k^*}(\theta_k)_{\gamma_k^*} + \delta_k\mu_{k|-k}$, then $\mathbb{E}(\beta_{Y_k} - \delta_k\mu_{k|-k}) = (\beta_X)_{\gamma_k^*}(\theta_k)_{\gamma_k^*}$. Thus, we recenter the genetic associations with the kth outcome such that, once conditioned on γ_k^*, they depend only on $(\beta_X)_{\gamma_k^*}(\theta_k)_{\gamma_k^*}$ as in eq. (12). The other terms in $m_{\gamma_k^*}$ mirror the mean of the full conditional for the regression coefficients of the linear model. In summary, the proposal density $q(\theta_k^*|\gamma_k^*, \beta_{Y_k}, Z_{_k}, \Delta, R)$ is inspired by the Gibbs updates of the regression coefficients in the linear model with suitable modifications to account for the conditional dependence between the kth outcome and the remaining responses at the summary level.

The designed proposal distribution allows also the "implicit marginalisation" of the direct causal effects when the joint update of (θ_k, γ_k), $k = 1, \ldots, q$, in the M-H step is performed. For the kth response, the acceptance probability α of the joint proposal is

$$\alpha = 1 \wedge \frac{\left|V_{\gamma_k^*}\right|^{1/2} v^{|\gamma_k|/2} \exp\left(\frac{1}{2}m_{\gamma_k^*}^T V_{\gamma_k^*}^{-1} m_{\gamma_k^*}\right) p(\gamma_k^*) q(\gamma_k|\gamma_k^*)}{\left|V_{\gamma_k}\right|^{1/2} v^{|\gamma_k^*|/2} \exp\left(\frac{1}{2}m_{\gamma_k}^T V_{\gamma_k}^{-1} m_{\gamma_k}\right) p(\gamma_k) q(\gamma_k^*|\gamma_k)}, \tag{S7}$$

where $p(\gamma_k)$ is defined in eq. (15) and $q(\gamma_k^*|\gamma_k)$ is the proposal distribution for the latent binary vector γ_k^* conditional to the current value γ_k. Similarly to [2], in eq. (S7), the current and proposed value of the direct causal effects (θ_k, θ_k^*) do not appear and the acceptance probability resembles the Bayes Factor of the standard linear regression model used in the update of the latent binary vector γ_k.

The advantages of the "implicit marginalisation" of the regression coefficients in eq. (S7) for the SUR model have not been recognised until recently [1]. This is a key point that distinguishes our prosed methodology from other SUR models [3, 4]. Similarly to MR^2, a sparse Bayesian SUR model [5] proposes two computational algorithms, an MCMC algorithm using Gibbs updates [6] for variable selection called "undirected" and a "directed" algorithm involving a Monte Carlo numerical approximation of the marginal likelihood to integrate out the regression coefficients combined with an M-H algorithm similar to eq. (S7) for posterior models' exploration. However, Monte Carlo numerical approximation is unstable and very time-consuming, making [5] unfeasible even for small data sets.

We conclude with the description regarding the MCMC steps required to obtain samples from $p(\Sigma|W, \mathrm{G})$ and transform them using the inverse transformation $R = \Delta^{-1}\Sigma\Delta^{-1}$, where Δ is sampled from its prior distribution in eq. (18). To sample the symmetric adjacency matrix G from $p(\mathrm{G}|\beta_Y, Z, \Delta, R)$, we target the distribution with density

$$p(\mathrm{G}|W) = \frac{p(W|\mathrm{G})p(\mathrm{G})}{\sum_{\mathrm{G}} p(W|\mathrm{G})p(\mathrm{G})}, \tag{S8}$$

where the summation in the denominator is over all the decomposable graphs G, $p(\mathrm{G})$ is defined in (17), W is defined in Appendix and $p(W|\mathrm{G})$ can be computed analytically due to the tractability of the hyper-inverse Wishart distribution [7]. Specifically, we sample from eq. (S8) by using an M-H step in which, conditionally on the current symmetric adjacency matrix G, a new graph is proposed by adding or deleting an edge between two vertices whose index has been chosen randomly between the vertices that belong to a decomposable graph. The proposed graph is then accepted or rejected using the accept/reject mechanism of the M-H step which targets the density in eq. (S8). Finally, conditionally on W and the updated symmetric adjacency matrix G, we sample Σ from its conditional hyper-inverse Wishart distribution, *i.e.*, $\mathrm{HIW}_{\mathcal{G}}(2 + n, I_q + W^T W)$.

S3 Simulation study

S3.1 Comparison of methods setup

MR^2 parameters setting

The MR^2 algorithm is run for $7,500$ MCMC iterations of which $2,500$ as burn-in and results saved every 5 iterations, resulting in $1,000$ posterior samples for all the unknowns. The hyper-parameters a_k and b_k in eq. (15) are chosen by specifying $\mathbb{E}(\gamma_k) = 2$ and $\mathbb{V}(\gamma_k) = 2$ which implies *a priori* a range between zero and seven for the number of significant direct causal effects for each outcome, see [1] for details. Another hyper-parameter to be specified is the prior variance v in eq. (13). We set $v = 1$ since the summary-level genetic associations with the risk factors in eq. (21) have been simulated with unit variance and no IVW is required given the same MAF for all simulated genotypes. To select a suitable number of MCMC iterations, and in particular the length of the burn-in phase, we ran some preliminary analyses and checked by visual inspection the trace plots of several posterior quantities including the number of exposures selected during the MCMC for all responses, the number of non-zero cells in the sparse matrix of residual correlation $R|Y$ and the square root of the error variance $\delta_k|Y$ for each response k, $k = 1, \dots, 5$. No signs of slow convergence or aberrant behaviour of the designed MCMC sampler are identified in a variety of scenarios and parameter settings, suggesting that the number of burn-in iterations are sufficient to reach convergence and sample from the equilibrium distribution of the Markov chain.

In case of different MAF across genotypes, we recommend setting $v = 1$ and standardise the summary-level genetic associations with the risk factors after IVW in order to place appropriate prior mass on reasonable values of the non-zero direct causal effects [8].

Alternative methods and parameters setting

As alternative methods, we include standard multivariable MR (MV-MR) which considers multiple exposures in one joint model but does not perform variable selection [9]. Next, we consider MR-BMA [10], a Bayesian variable selection approach for multivariable MR, with a prior probability of inclusion set at $2/15 = 0.13$ reflecting two associated causal exposures for each outcome. In this way, we match the prior specification of the expected number of risk factors associated with the kth response used in MR^2. Both MV-MR and MR-BMA only consider one outcome at-a-time and are performed on each outcome separately.

Additionally, we include two multivariate and multivariable variable selection approaches which have to date not been applied to MR, the multiple responses Lasso (Multivariate Regression With Covariance Estimation, MRCE) [3] and the multiple responses Spike and Slab Lasso (mSSL) [4].

Regarding the MRCE algorithm, we select the cross-validation procedure option for the sparse Lasso solution which performs k-fold cross-validation using candidate tuning parameters specified in the vectors λ_1 and λ_2, the two penalisation parameters for the sparse regression and sparse covariance estimation, respectively. We run the algorithm with the default recommended values including 5-fold cross-validation and 40 equally spaced candidate tuning parameters λ_1 and λ_2 between 0 and 1. Results (not shown) do not change if a finer grid of points for λ_1 and λ_2 are chosen.

We use the default parametrization for the instance of the mSSL algorithm that performs "dynamic posterior exploration" (mSSL_dpe) which is the slowest, but the most accurate version of the algorithm. As recommended, we also run the "dynamic conditional posterior exploration" (mSSL_dcpe) and "separate variable and covariance selection" (sep_sslg) versions of the algorithm, but with inferior results (data not shown).

A unit variance of the predictors is required by both MRCE and mSSL algorithms. This condition is automatically satisfied since, in all simulated scenarios, the summary-level genetic associations with the risk factors in eq. (21) have unit variance. The simulation setup also simplifies the choice of prior variance of the non-zero regression coefficients used in MR-BMA. We use the same specification ($v = 1$) employed in MR^2. The output of MRCE and mSSL algorithms consists of the sparse Lasso solution of the direct causal effects $\Theta = (\theta_1, \ldots, \theta_q)^T$ and residual covariance Σ, or its inverse Ω, including the intercepts for each outcome. Thus, both MRCE and mSSL can be seen as the multi-response versions, with exposure selection, of multivariable MR-Egger [11], see Table S1. However, in the designed simulation setup, the unmeasured pleiotropic pathway is shared across responses and it induces residual correlation at the summary-level data as shown in eq. (5). Thus, its effect is detected by the estimated residual covariance Σ with the estimated intercepts close to zero (data not shown). Similar considerations hold when the residual correlation is induced by directly modelling the correlation between the individual-level responses' errors.

Performance measures

There are two important aspects when comparing methods for multivariable MR. First, the ability of the algorithms to detect the true causal risk factors and, second, the accuracy of the direct causal effect estimates. Receiver op-

erating characteristic (ROC) curves plot the true positive rate (TPR) against the false positive rate (FPR) and illustrate how well the methods can distinguish between true and false causal exposures when the discrimination threshold is varied. As discrimination threshold, in MV-MR we rank according to p-values of the direct causal effect estimates and in MR-BMA and MR^2 according to mPPI. The two multi-response implementations of Lasso rank exposures by their causal effect estimates of which the unimportant ones are forced to zero and thus excluded from the model. Consequently, these two methods do not provide a full ranking of all exposures, but rather a single threshold. Thus, they are presented in the ROC curves as a single point instead of a continuous line.

Second, to contrast the quality of the direct causal effect estimation, we calculate the sum of squared errors (SSE), defined as the squared difference between the estimated and the true simulated direct causal effect. The SSE captures both the squared bias and the variance, thus penalizing methods with a wide spread and high variability.

S3.2 Supplemental tables

	Designed for MR	Bayesian method	Multivariate outcomes	Selection risk factors	Estimation residual correlation	Reference
MV-MR	✓	✗	✗	✗	✗	Burgess *et al.* 2015 [9]
MR-BMA	✓	✓	✗	✓	✗	Zuber *et al.* 2020 [10]
MRCE	✗	✓	✓	✓	✓	Rothman *et al.* 2010 [3]
mSSL	✗	✓	✓	✓	✓	Deshpande *et al.* 2019 [4]
MR^2	✓	✓	✓	✓	✓	

Table S1. Overview of competing multivariable MR models that consider one outcome at-a-time and multi-response multivariable methods used in the simulation study. For each method, we indicate if: It has been designed specifically for MR, it is a Bayesian method, it can analyse multiple outcomes jointly, it can provide selection of important risk factors associated with the outcomes and it can estimate residual correlation between summary-level responses.

Scenario	Direct causal effect	Correlation among residuals	Further notes	Open parameters
I-Null	$\theta_k = 0$	No	Null direct causal effect of all exposures, only the confounders have an effect	Impact of confounder: $\theta_X^U = 2$, $\theta_Y^U = 1$
II-Confounding	$\theta_k \neq 0$	No	Direct causal effect on randomly selected exposure-confounder pairs	Impact of confounder: $\theta_X^U = 2$, $\theta_Y^U = 1$
III-Undirected pleiotropy	$\theta_k \neq 0$	Yes, induced by pleiotropic pathway	Additional effect of shared pleiotropic pathway A ("undirected": $A_l \gtrless 0$) on the level of outcomes	Pleiotropic effect: $\theta^A = \{0.25, 0.5, 0.75, 1, 1.5, 2\}$
IV-Directed pleiotropy	$\theta_k \neq 0$	Yes, induced by pleiotropic pathway	Additional effect of shared pleiotropic pathway A ("directed": $A_l > 0$) on the level of outcomes	Pleiotropic effect: $\theta^A = \{0.25, 0.5, 0.75, 1, 1.5, 2\}$
V-Dependence	$\theta_k \neq 0$	Yes, modelled direclty	Correlation between individual-level responses' errors in eq. (5)	Correlation level: $r_Y = \{0, 0.2, 0.4, 0.6, 0.8\}$

Table S2. Overview of different simulations setting. Additional open parameter is the correlation between exposures $r_X = \{0, 0.2, 0.4, 0.6, 0.8\}$. Fixed parameters are the sample size of independent subjects ($N = 50,000$) for the first (risk factors) and second (responses) level of the simulations, the number of genetic variants used as instrumental variables ($n = 100$), the range of sought values for the exposure effects (β_{X_j}, $j = 1, \ldots, p$, should be in the range between -2 and 2), the percentage of simulated non-zero direct causal effects among the exposure-outcome combinations (30%), the range of sought values for the direct causal effects (θ_k, $k = 1, \ldots, q$, should be in the range between -2 and 2) and the heritability to generate $p = 15$ exposures ($h_X = 10\%$) and the proportion of variance explained for each of $q = 5$ outcomes ($h_Y = 25\%$).

	θ_X^U		θ_Y^U	
			1	2
r_X = 0	1	MV-MR	0.958 (0.026)	0.958 (0.026)
		MR-BMA	0.960 (0.025)	0.961 (0.025)
		MRCE	0.959 (0.025)	0.959 (0.026)
		mSSL	0.901 (0.072)	0.909 (0.048)
		MR^2	**0.961** (0.025)	**0.962** (0.025)
	2	MV-MR	0.951 (0.030)	0.952 (0.030)
		MR-BMA	0.956 (0.028)	0.956 (0.029)
		MRCE	0.952 (0.036)	0.953 (0.037)
		mSSL	0.879 (0.067)	0.892 (0.044)
		MR^2	**0.961** (0.025)	**0.960** (0.025)
0.2	1	MV-MR	0.958 (0.024)	0.959 (0.024)
		MR-BMA	0.962 (0.025)	0.962 (0.025)
		MRCE	0.957 (0.029)	0.958 (0.026)
		mSSL	0.906 (0.048)	0.901 (0.050)
		MR^2	**0.964** (0.023)	**0.964** (0.025)
	2	MV-MR	0.951 (0.027)	0.951 (0.027)
		MR-BMA	**0.957** (0.027)	0.956 (0.027)
		MRCE	0.952 (0.027)	0.950 (0.029)
		mSSL	0.886 (0.071)	0.888 (0.039)
		MR^2	**0.957** (0.026)	**0.958** (0.024)
0.4	1	MV-MR	0.946 (0.041)	0.946 (0.041)
		MR-BMA	0.955 (0.034)	0.956 (0.034)
		MRCE	0.948 (0.038)	0.947 (0.039)
		mSSL	0.899 (0.050)	0.897 (0.043)
		MR^2	**0.962** (0.028)	**0.960** (0.032)
	2	MV-MR	0.940 (0.042)	0.937 (0.042)
		MR-BMA	0.950 (0.038)	0.949 (0.037)
		MRCE	0.943 (0.040)	0.942 (0.039)
		mSSL	0.870 (0.060)	0.880 (0.051)
		MR^2	**0.953** (0.037)	**0.953** (0.035)
0.6	1	MV-MR	0.928 (0.038)	0.930 (0.038)
		MR-BMA	0.940 (0.031)	0.941 (0.031)
		MRCE	0.936 (0.034)	0.937 (0.032)
		mSSL	0.884 (0.047)	0.893 (0.048)
		MR^2	**0.948** (0.030)	**0.946** (0.029)
	2	MV-MR	0.922 (0.040)	0.924 (0.038)
		MR-BMA	0.932 (0.034)	0.934 (0.035)
		MRCE	0.926 (0.035)	0.927 (0.037)
		mSSL	0.876 (0.044)	0.880 (0.046)
		MR^2	**0.939** (0.033)	**0.944** (0.029)
0.8	1	MV-MR	0.909 (0.034)	0.909 (0.033)
		MR-BMA	0.924 (0.040)	0.925 (0.040)
		MRCE	0.914 (0.039)	0.916 (0.039)
		mSSL	0.867 (0.058)	0.870 (0.048)
		MR^2	**0.932** (0.035)	**0.937** (0.036)
	2	MV-MR	0.896 (0.037)	0.899 (0.038)
		MR-BMA	0.916 (0.041)	0.919 (0.041)
		MRCE	0.900 (0.048)	0.904 (0.044)
		mSSL	0.846 (0.058)	0.851 (0.052)
		MR^2	**0.927** (0.038)	**0.932** (0.037)

Table S3. Scenario II-Confounding: Area under the receiver operating characteristic (AUC) of the simulated scenario, averaged over 50 replicates, with standard deviation across 50 replicates in brackets, illustrating the performance of different MR multivariable implementations and multi-response multivariable methods. In simulated Scenario II-Confounding, 30% of all exposure-confounder combinations are generated to have a direct causal effect. Open parameters are the correlation between exposures $r_X = \{0, 0.2, 0.4, 0.6, 0.8\}$ and the confounding effects on the exposures $\theta_X^U = \{1, 2\}$ and outcomes $\theta_Y^U = \{1, 2\}$. Best results are highlighted in bold.

		θ^A					
		0.25	0.5	0.75	1	1.5	2
r_X	0						
	MV-MR	0.943 (0.033)	0.927 (0.040)	0.912 (0.045)	0.896 (0.049)	0.859 (0.052)	0.824 (0.057)
	MR-BMA	0.948 (0.032)	0.933 (0.035)	0.916 (0.040)	0.899 (0.046)	0.864 (0.056)	0.832 (0.058)
	MRCE	0.943 (0.037)	0.935 (0.038)	0.930 (0.038)	0.923 (0.039)	0.905 (0.039)	0.891 (0.042)
	mSSL	0.889 (0.051)	0.868 (0.051)	0.867 (0.057)	0.861 (0.056)	0.845 (0.064)	0.814 (0.062)
	MR^2	**0.949** (0.030)	**0.945** (0.034)	**0.933** (0.035)	**0.932** (0.035)	**0.914** (0.042)	**0.909** (0.038)
	0.2						
	MV-MR	0.946 (0.033)	0.931 (0.036)	0.910 (0.047)	0.884 (0.056)	0.845 (0.067)	0.811 (0.073)
	MR-BMA	0.948 (0.033)	0.935 (0.033)	0.918 (0.039)	0.894 (0.047)	0.851 (0.061)	0.816 (0.064)
	MRCE	0.948 (0.035)	0.941 (0.032)	0.934 (0.038)	0.924 (0.041)	0.912 (0.046)	0.894 (0.050)
	mSSL	0.880 (0.064)	0.858 (0.066)	0.852 (0.059)	0.844 (0.068)	0.832 (0.069)	0.822 (0.054)
	MR^2	**0.952** (0.032)	**0.947** (0.031)	**0.946** (0.029)	**0.933** (0.031)	**0.925** (0.033)	**0.909** (0.043)
	0.4						
	MV-MR	0.942 (0.036)	0.922 (0.043)	0.898 (0.049)	0.875 (0.055)	0.827 (0.062)	0.792 (0.067)
	MR-BMA	0.945 (0.035)	0.929 (0.039)	0.913 (0.043)	0.892 (0.051)	0.851 (0.056)	0.809 (0.059)
	MRCE	0.938 (0.037)	0.930 (0.039)	0.924 (0.044)	0.913 (0.047)	0.900 (0.055)	0.884 (0.053)
	mSSL	0.867 (0.061)	0.860 (0.061)	0.849 (0.056)	0.845 (0.062)	0.830 (0.057)	0.803 (0.067)
	MR^2	**0.948** (0.036)	**0.941** (0.042)	**0.936** (0.040)	**0.927** (0.045)	**0.908** (0.052)	**0.898** (0.050)
	0.6						
	MV-MR	0.915 (0.045)	0.898 (0.052)	0.877 (0.056)	0.850 (0.059)	0.803 (0.065)	0.765 (0.075)
	MR-BMA	0.929 (0.037)	0.913 (0.041)	0.895 (0.045)	0.875 (0.050)	0.825 (0.068)	0.791 (0.081)
	MRCE	0.921 (0.041)	0.906 (0.051)	0.904 (0.046)	0.896 (0.046)	0.870 (0.050)	0.852 (0.059)
	mSSL	0.856 (0.055)	0.849 (0.052)	0.842 (0.065)	0.834 (0.068)	0.811 (0.068)	0.792 (0.071)
	MR^2	**0.938** (0.035)	**0.929** (0.037)	**0.918** (0.041)	**0.912** (0.042)	**0.895** (0.048)	**0.877** (0.055)
	0.8						
	MV-MR	0.887 (0.058)	0.862 (0.062)	0.828 (0.067)	0.793 (0.072)	0.746 (0.076)	0.703 (0.081)
	MR-BMA	0.897 (0.045)	0.879 (0.048)	0.854 (0.049)	0.827 (0.056)	0.776 (0.072)	0.743 (0.08)
	MRCE	0.882 (0.048)	0.874 (0.053)	0.869 (0.054)	0.854 (0.065)	0.835 (0.066)	0.827 (0.067)
	mSSL	0.838 (0.063)	0.809 (0.060)	0.795 (0.065)	0.791 (0.061)	0.749 (0.074)	0.736 (0.072)
	MR^2	**0.912** (0.042)	**0.902** (0.047)	**0.896** (0.042)	**0.884** (0.043)	**0.855** (0.054)	**0.838** (0.054)

Table S4. Simulation III-Undirected pleiotropy: Area under the receiver operating characteristic (AUC) of the simulated scenario, averaged over 50 replicates, with standard deviation across 50 replicates in brackets, illustrating the performance of different MR multivariable implementations and multi-response multivariable methods. In simulated Scenario III-Undirected pleiotropy, 30% of all exposure-confounder combinations are generated to have a direct causal effect. Open parameters are the correlation between exposures $r_X = \{0, 0.2, 0.4, 0.6, 0.8\}$ and the pleiotropic effect $\theta^A = \{0.25, 0.5, 0.75, 1, 1.5, 2\}$. Confounding effects on the exposures and outcomes are fixed at $\theta_X^U = 2$ and $\theta_Y^U = 1$, respectively. Best results are highlighted in bold.

			θ^A					
			0.25	0.5	0.75	1	1.5	2
r_X	0	MV-MR	0.943 (0.035)	0.931 (0.038)	0.917 (0.040)	0.900 (0.043)	0.857 (0.051)	0.818 (0.056)
		MR-BMA	0.947 (0.033)	0.937 (0.034)	0.924 (0.039)	0.907 (0.040)	0.863 (0.050)	0.826 (0.055)
		MRCE	0.946 (0.038)	0.943 (0.034)	0.934 (0.038)	0.928 (0.040)	**0.918** (0.037)	0.903 (0.044)
		mSSL	0.895 (0.052)	0.880 (0.057)	0.876 (0.053)	0.869 (0.062)	0.846 (0.055)	0.827 (0.063)
		MR^2	**0.952** (0.035)	**0.946** (0.038)	**0.938** (0.033)	**0.933** (0.033)	0.915 (0.040)	**0.904** (0.045)
	0.2	MV-MR	0.947 (0.034)	0.931 (0.039)	0.912 (0.048)	0.889 (0.054)	0.850 (0.061)	0.804 (0.076)
		MR-BMA	0.948 (0.034)	0.933 (0.037)	0.916 (0.043)	0.893 (0.051)	0.853 (0.064)	0.813 (0.072)
		MRCE	0.949 (0.035)	0.944 (0.033)	0.942 (0.034)	0.934 (0.031)	**0.923** (0.037)	**0.913** (0.042)
		mSSL	0.889 (0.050)	0.865 (0.069)	0.865 (0.060)	0.860 (0.059)	0.843 (0.057)	0.823 (0.067)
		MR^2	**0.950** (0.033)	**0.948** (0.029)	**0.940** (0.035)	**0.940** (0.029)	0.922 (0.039)	0.911 (0.039)
	0.4	MV-MR	0.941 (0.036)	0.923 (0.042)	0.900 (0.050)	0.877 (0.054)	0.832 (0.056)	0.783 (0.065)
		MR-BMA	0.947 (0.034)	0.934 (0.036)	0.916 (0.043)	0.895 (0.052)	0.852 (0.057)	0.806 (0.063)
		MRCE	0.943 (0.036)	0.936 (0.036)	0.929 (0.039)	0.923 (0.044)	0.912 (0.048)	**0.892** (0.051)
		mSSL	0.889 (0.058)	0.874 (0.058)	0.859 (0.060)	0.851 (0.054)	0.832 (0.060)	0.811 (0.062)
		MR^2	**0.948** (0.035)	**0.946** (0.036)	**0.934** (0.042)	**0.926** (0.044)	**0.914** (0.048)	0.889 (0.056)
	0.6	MV-MR	0.919 (0.044)	0.903 (0.047)	0.881 (0.052)	0.857 (0.057)	0.804 (0.070)	0.756 (0.078)
		MR-BMA	0.933 (0.037)	0.919 (0.044)	0.901 (0.050)	0.876 (0.058)	0.828 (0.062)	0.791 (0.071)
		MRCE	0.923 (0.041)	0.917 (0.041)	0.906 (0.047)	0.904 (0.048)	0.887 (0.048)	0.874 (0.051)
		mSSL	0.868 (0.056)	0.859 (0.053)	0.850 (0.060)	0.840 (0.055)	0.820 (0.058)	0.796 (0.072)
		MR^2	**0.940** (0.033)	**0.929** (0.039)	**0.920** (0.039)	**0.917** (0.043)	**0.895** (0.043)	**0.883** (0.050)
	0.8	MV-MR	0.885 (0.053)	0.852 (0.059)	0.818 (0.069)	0.793 (0.076)	0.738 (0.081)	0.703 (0.084)
		MR-BMA	0.895 (0.042)	0.874 (0.046)	0.850 (0.050)	0.826 (0.055)	0.780 (0.067)	0.747 (0.072)
		MRCE	0.887 (0.047)	0.880 (0.050)	0.874 (0.052)	0.867 (0.052)	0.852 (0.060)	0.834 (0.066)
		mSSL	0.842 (0.061)	0.827 (0.071)	0.807 (0.068)	0.797 (0.057)	0.766 (0.083)	0.748 (0.075)
		MR^2	**0.912** (0.043)	**0.905** (0.038)	**0.893** (0.044)	**0.881** (0.048)	**0.855** (0.054)	**0.843** (0.048)

Table S5. Simulation IV-Directed pleiotropy: Area under the receiver operating characteristic (AUC) of the simulated scenario, averaged over 50 replicates, with standard deviation across 50 replicates in brackets, illustrating the performance of different MR multivariable implementations and multi-response multivariable methods. In simulated Scenario IV-Directed pleiotropy, 30% of all exposure-confounder combinations are generated to have a direct causal effect. Open parameters are the correlation between exposures $r_X = \{0, 0.2, 0.4, 0.6, 0.8\}$ and the pleiotropic effect $\theta^A = \{0.25, 0.5, 0.75, 1, 1.5, 2\}$. Confounding effects on the exposures and outcomes are fixed at $\theta_X^U = 2$ and $\theta_Y^U = 1$, respectively. Best results are highlighted in bold.

		r_Y				
		0	0.2	0.4	0.6	0.8
r_X 0	MV-MR	0.951 (0.030)	0.951 (0.028)	0.953 (0.026)	0.950 (0.026)	0.954 (0.023)
	MR-BMA	0.956 (0.028)	0.954 (0.029)	0.956 (0.026)	0.955 (0.026)	0.956 (0.023)
	MRCE	0.951 (0.037)	0.951 (0.033)	0.951 (0.033)	0.953 (0.028)	0.955 (0.024)
	mSSL	0.886 (0.068)	0.893 (0.069)	0.886 (0.063)	0.884 (0.069)	0.896 (0.069)
	MR^2	**0.958** (0.024)	**0.962** (0.024)	**0.961** (0.027)	**0.962** (0.024)	**0.967** (0.020)
0.2	MV-MR	0.951 (0.027)	0.949 (0.028)	0.951 (0.030)	0.951 (0.032)	0.950 (0.032)
	MR-BMA	**0.957** (0.027)	**0.957** (0.028)	**0.958** (0.029)	0.957 (0.029)	0.955 (0.030)
	MRCE	0.953 (0.028)	0.953 (0.027)	0.954 (0.029)	0.952 (0.030)	0.956 (0.031)
	mSSL	0.890 (0.065)	0.900 (0.067)	0.888 (0.071)	0.894 (0.082)	0.887 (0.085)
	MR^2	**0.957** (0.026)	0.956 (0.025)	0.955 (0.029)	**0.960** (0.027)	**0.968** (0.027)
0.4	MV-MR	0.940 (0.042)	0.949 (0.030)	0.944 (0.034)	0.946 (0.032)	0.948 (0.029)
	MR-BMA	0.950 (0.038)	0.953 (0.029)	0.952 (0.033)	0.953 (0.032)	0.956 (0.031)
	MRCE	0.942 (0.041)	0.948 (0.032)	0.943 (0.038)	0.945 (0.036)	0.949 (0.035)
	mSSL	0.880 (0.050)	0.885 (0.051)	0.877 (0.064)	0.885 (0.070)	0.889 (0.075)
	MR^2	**0.953** (0.037)	**0.958** (0.026)	**0.956** (0.029)	**0.957** (0.031)	**0.961** (0.029)
0.6	MV-MR	0.922 (0.040)	0.928 (0.037)	0.925 (0.040)	0.930 (0.037)	0.930 (0.044)
	MR-BMA	0.932 (0.034)	0.935 (0.040)	0.934 (0.037)	0.936 (0.032)	0.935 (0.033)
	MRCE	0.924 (0.036)	0.933 (0.040)	0.927 (0.042)	0.932 (0.037)	0.930 (0.046)
	mSSL	0.878 (0.066)	0.870 (0.054)	0.875 (0.053)	0.869 (0.067)	0.878 (0.079)
	MR^2	**0.939** (0.033)	**0.943** (0.033)	**0.943** (0.036)	**0.952** (0.031)	**0.951** (0.029)
0.8	MV-MR	0.896 (0.037)	0.900 (0.039)	0.901 (0.041)	0.902 (0.039)	0.901 (0.037)
	MR-BMA	0.916 (0.041)	0.909 (0.039)	0.912 (0.037)	0.911 (0.039)	0.910 (0.038)
	MRCE	0.904 (0.043)	0.899 (0.049)	0.901 (0.042)	0.903 (0.041)	0.909 (0.042)
	mSSL	0.849 (0.059)	0.821 (0.082)	0.843 (0.073)	0.855 (0.062)	0.835 (0.088)
	MR^2	**0.927** (0.038)	**0.922** (0.032)	**0.926** (0.035)	**0.934** (0.030)	**0.936** (0.035)

Table S6. Simulation V-Dependence: Area under the receiver operating characteristic (AUC) of the simulated scenario, averaged over 50 replicates, with standard deviation across 50 replicates in brackets, illustrating the performance of different MR multivariable implementations and multi-response multivariable methods. In simulated Scenario V-Dependence, 30% of all exposure-confounder combinations are generated to have a direct causal effect. Open parameters are the correlation between exposures $r_X = \{0, 0.2, 0.4, 0.6, 0.8\}$ and the correlation between individual-level responses' errors $r_Y = \{0, 0.2, 0.4, 0.6, 0.8\}$. Confounding effects on the exposures and outcomes are fixed at $\theta_X^U = 2$ and $\theta_Y^U = 1$, respectively. Best results are highlighted in bold.

			r_Y				
			0	0.2	0.4	0.6	0.8
r_X	0	MV-MR	0.001 (<0.001)	0.001 (<0.001)	0.001 (<0.001)	0.001 (<0.001)	0.001 (<0.001)
		MR-BMA	**0.000** (<0.001)	**0.000** (<0.001)	**0.000** (<0.001)	**0.000** (<0.001)	**0.000** (<0.001)
		MRCE	**0.000** (<0.001)	**0.000** (<0.001)	**0.000** (<0.001)	**0.000** (<0.001)	**0.000** (<0.001)
		mSSL	**0.000** (<0.001)	**0.000** (<0.001)	**0.000** (<0.001)	**0.000** (<0.001)	**0.000** (<0.001)
		MR^2	**0.000** (<0.001)	**0.000** (<0.001)	**0.000** (<0.001)	**0.000** (<0.001)	**0.000** (<0.001)
	0.2	MV-MR	0.001 (<0.001)	0.001 (<0.001)	0.001 (<0.001)	0.001 (<0.001)	0.001 (<0.001)
		MR-BMA	**0.000** (<0.001)	**0.000** (<0.001)	**0.000** (<0.001)	**0.000** (<0.001)	**0.000** (<0.001)
		MRCE	**0.000** (<0.001)	**0.000** (<0.001)	**0.000** (<0.001)	**0.000** (<0.001)	**0.000** (<0.001)
		mSSL	**0.000** (<0.001)	**0.000** (<0.001)	**0.000** (<0.001)	**0.000** (<0.001)	**0.000** (<0.001)
		MR^2	**0.000** (<0.001)	**0.000** (<0.001)	**0.000** (<0.001)	**0.000** (<0.001)	**0.000** (<0.001)
	0.4	MV-MR	0.001 (<0.001)	0.001 (<0.001)	0.001 (<0.001)	0.001 (<0.001)	0.001 (<0.001)
		MR-BMA	**0.000** (<0.001)	**0.000** (<0.001)	**0.000** (<0.001)	**0.000** (<0.001)	**0.000** (<0.001)
		MRCE	0.001 (<0.001)	0.001 (<0.001)	0.001 (<0.001)	0.001 (<0.001)	0.001 (<0.001)
		mSSL	**0.000** (<0.001)	**0.000** (<0.001)	**0.000** (<0.001)	**0.000** (<0.001)	**0.000** (<0.001)
		MR^2	**0.000** (<0.001)	**0.000** (<0.001)	**0.000** (<0.001)	**0.000** (<0.001)	**0.000** (<0.001)
	0.6	MV-MR	0.001 (<0.001)	0.001 (<0.001)	0.001 (<0.001)	0.001 (<0.001)	0.001 (<0.001)
		MR-BMA	**0.000** (<0.001)	**0.000** (<0.001)	**0.000** (<0.001)	**0.000** (<0.001)	**0.000** (<0.001)
		MRCE	0.001 (<0.001)	0.001 (<0.001)	0.001 (<0.001)	0.001 (<0.001)	0.001 (<0.001)
		mSSL	**0.000** (<0.001)	**0.000** (<0.001)	**0.000** (<0.001)	**0.000** (<0.001)	**0.000** (<0.001)
		MR^2	**0.000** (<0.001)	**0.000** (<0.001)	**0.000** (<0.001)	**0.000** (<0.001)	**0.000** (<0.001)
	0.8	MV-MR	0.003 (<0.001)	0.003 (0.001)	0.003 (0.001)	0.003 (0.001)	0.003 (0.001)
		MR-BMA	**0.000** (<0.001)	**0.000** (<0.001)	**0.000** (<0.001)	**0.000** (<0.001)	**0.000** (<0.001)
		MRCE	0.001 (<0.001)	0.001 (<0.001)	0.001 (<0.001)	0.001 (<0.001)	0.001 (<0.001)
		mSSL	**0.000** (<0.001)	**0.000** (<0.001)	**0.000** (<0.001)	**0.000** (<0.001)	**0.000** (<0.001)
		MR^2	**0.000** (<0.001)	**0.000** (<0.001)	**0.000** (<0.001)	**0.000** (<0.001)	**0.000** (<0.001)

Table S7. Scenario I-Null: Sum of squared errors (SSE) of the simulated scenario, averaged over 50 replicates, with standard deviation across 50 replicates in brackets, illustrating the performance of different MR multivariable implementations and multi-response multivariable methods to estimate the true simulated effect size. In simulated Scenario I- Null, no direct causal effects are simulated and the outcomes are generated based only on the effect of the confounders. Open parameters are the correlation between exposures $r_X = \{0, 0.2, 0.4, 0.6, 0.8\}$ and the correlation between individual-level responses' errors $r_Y = \{0, 0.2, 0.4, 0.6, 0.8\}$. Best results are highlighted in bold.

r_X	θ_X^U		θ_Y^U = 1	θ_Y^U = 2
0	1	MV-MR	0.494 (0.200)	0.491 (0.203)
		MR-BMA	0.294 (0.141)	0.287 (0.140)
		MRCE	0.368 (0.161)	0.364 (0.163)
		mSSL	0.417 (0.644)	0.333 (0.179)
		MR^2	**0.261** (0.126)	**0.252** (0.120)
	2	MV-MR	0.611 (0.246)	0.616 (0.247)
		MR-BMA	0.347 (0.163)	0.341 (0.159)
		MRCE	0.449 (0.196)	0.449 (0.199)
		mSSL	0.577 (0.831)	0.432 (0.208)
		MR^2	**0.316** (0.143)	**0.311** (0.140)
0.2	1	MV-MR	0.577 (0.227)	0.598 (0.261)
		MR-BMA	0.338 (0.165)	0.354 (0.200)
		MRCE	0.416 (0.182)	0.432 (0.221)
		mSSL	0.377 (0.181)	0.398 (0.216)
		MR^2	**0.297** (0.148)	**0.309** (0.177)
	2	MV-MR	0.730 (0.322)	0.743 (0.322)
		MR-BMA	0.417 (0.220)	0.425 (0.221)
		MRCE	0.530 (0.268)	0.539 (0.270)
		mSSL	0.646 (0.966)	0.493 (0.223)
		MR^2	**0.378** (0.199)	**0.386** (0.200)
0.4	1	MV-MR	0.711 (0.325)	0.863 (0.325)
		MR-BMA	0.410 (0.209)	0.478 (0.211)
		MRCE	0.522 (0.257)	0.633 (0.263)
		mSSL	0.462 (0.252)	0.589 (0.251)
		MR^2	**0.355** (0.185)	**0.430** (0.189)
	2	MV-MR	0.704 (0.385)	0.875 (0.493)
		MR-BMA	0.409 (0.235)	0.478 (0.299)
		MRCE	0.523 (0.321)	0.638 (0.402)
		mSSL	0.461 (0.340)	0.552 (0.372)
		MR^2	**0.353** (0.216)	**0.432** (0.265)
0.6	1	MV-MR	0.994 (0.383)	1.005 (0.597)
		MR-BMA	0.555 (0.239)	0.553 (0.373)
		MRCE	0.730 (0.331)	0.729 (0.516)
		mSSL	0.569 (0.277)	0.564 (1.117)
		MR^2	**0.440** (0.217)	**0.444** (0.340)
	2	MV-MR	1.246 (0.490)	1.261 (0.605)
		MR-BMA	0.673 (0.302)	0.677 (0.514)
		MRCE	0.904 (0.405)	0.925 (0.367)
		mSSL	0.881 (0.374)	0.718 (0.414)
		MR^2	**0.567** (0.269)	**0.577** (0.331)
0.8	1	MV-MR	2.015 (1.013)	1.990 (0.984)
		MR-BMA	1.223 (0.724)	1.183 (0.664)
		MRCE	1.330 (0.684)	1.301 (0.643)
		mSSL	1.178 (1.093)	1.022 (0.647)
		MR^2	**0.793** (0.533)	**0.761** (0.455)
	2	MV-MR	2.496 (1.192)	2.519 (1.191)
		MR-BMA	1.441 (0.807)	1.469 (0.808)
		MRCE	1.667 (0.806)	1.689 (0.788)
		mSSL	1.475 (1.113)	1.392 (0.810)
		MR^2	**1.057** (0.629)	**1.077** (0.614)

Table S8. Scenario II-Confounding: Sum of squared errors (SSE) of the simulated scenario, averaged over 50 replicates, with standard deviation across 50 replicates in brackets, illustrating the performance of different MR multivariable implementations and multi-response multivariable methods to estimate the true effect size. In simulated Scenario II-Confounding, 30% of all exposure-confounder combinations are generated to have a direct causal effect. Open parameters are the correlation between exposures $r_X = \{0, 0.2, 0.4, 0.6, 0.8\}$ and confounding effects on the exposures $\theta_X^U = \{1, 2\}$ and outcomes $\theta_Y^U = \{1, 2\}$. Best results are highlighted in bold.

		θ^A					
		0.25	0.5	0.75	1	1.5	2
r_X	0	MV-MR 0.683 (0.254)	0.944 (0.329)	1.320 (0.417)	1.874 (0.565)	3.559 (1.094)	5.801 (1.859)
		MR-BMA 0.407 (0.179)	0.502 (0.210)	0.648 (0.266)	0.859 (0.343)	1.556 (0.596)	2.662 (1.021)
		MRCE 0.521 (0.209)	0.607 (0.239)	0.670 (0.274)	0.736 (0.297)	1.003 (0.601)	1.340 (0.887)
		mSSL 0.465 (0.249)	0.530 (0.257)	0.560 (0.277)	0.605 (0.294)	0.721 (0.393)	1.017 (0.788)
		MR^2 **0.360** (0.160)	**0.406** (0.159)	**0.431** (0.168)	**0.462** (0.162)	**0.610** (0.499)	**0.652** (0.337)
	0.2	MV-MR 0.783 (0.379)	1.054 (0.442)	1.514 (0.553)	2.179 (0.686)	4.015 (1.321)	6.445 (2.103)
		MR-BMA 0.428 (0.209)	0.563 (0.242)	0.774 (0.288)	1.065 (0.375)	1.756 (0.571)	2.731 (0.841)
		MRCE 0.567 (0.274)	0.666 (0.316)	0.786 (0.361)	0.893 (0.431)	1.141 (0.649)	1.425 (0.876)
		mSSL 0.561 (0.482)	0.630 (0.443)	0.724 (0.461)	0.747 (0.462)	0.895 (0.521)	**1.081** (0.516)
		MR^2 **0.370** (0.212)	**0.425** (0.231)	**0.498** (0.253)	**0.540** (0.267)	**0.735** (0.484)	1.092 (0.965)
	0.4	MV-MR 0.936 (0.376)	1.261 (0.450)	1.822 (0.578)	2.673 (0.903)	5.035 (1.847)	8.244 (3.092)
		MR-BMA 0.517 (0.240)	0.648 (0.273)	0.869 (0.335)	1.201 (0.450)	2.204 (0.906)	3.542 (1.403)
		MRCE 0.689 (0.302)	0.778 (0.321)	0.882 (0.353)	1.032 (0.498)	1.325 (0.917)	1.629 (1.073)
		mSSL 0.590 (0.289)	0.682 (0.302)	0.702 (0.295)	0.778 (0.354)	1.089 (0.872)	1.349 (0.952)
		MR^2 **0.436** (0.237)	**0.491** (0.241)	**0.520** (0.242)	**0.647** (0.621)	**0.844** (0.899)	**1.088** (1.137)
	0.6	MV-MR 1.332 (0.642)	1.826 (0.763)	2.699 (1.051)	3.869 (1.502)	7.017 (2.846)	11.518 (5.051)
		MR-BMA 0.774 (0.571)	0.971 (0.719)	1.330 (0.883)	1.714 (1.036)	2.965 (1.483)	4.487 (1.944)
		MRCE 0.947 (0.551)	1.094 (0.613)	1.251 (0.668)	1.434 (0.877)	1.806 (1.128)	2.191 (1.176)
		mSSL 0.877 (0.663)	0.933 (0.678)	1.013 (0.720)	1.104 (0.790)	1.410 (0.943)	1.795 (1.149)
		MR^2 **0.624** (0.523)	**0.693** (0.614)	**0.759** (0.658)	**0.858** (0.722)	**1.125** (0.987)	**1.565** (1.284)
	0.8	MV-MR 2.716 (1.263)	3.693 (1.544)	5.272 (2.085)	7.728 (2.944)	13.611 (5.260)	22.417 (8.816)
		MR-BMA 1.617 (0.722)	1.922 (0.858)	2.432 (1.045)	3.065 (1.284)	4.913 (1.875)	7.036 (2.735)
		MRCE 1.891 (0.764)	2.118 (0.858)	2.380 (1.091)	2.667 (1.275)	3.004 (1.203)	3.456 (1.478)
		mSSL 1.538 (0.795)	1.929 (1.204)	2.102 (1.180)	2.325 (1.316)	3.309 (2.217)	3.817 (2.051)
		MR^2 **1.198** (0.594)	**1.391** (0.644)	**1.560** (0.759)	**1.731** (0.815)	**2.251** (1.153)	**2.707** (1.310)

Table S9. Simulation III-Undirected pleiotropy: Sum of squared errors (SSE) of the simulated scenario, averaged over 50 replicates, with standard deviation across 50 replicates in brackets, illustrating the performance of different MR multivariable implementations and multi-response multivariable methods to estimate the true effect size. In simulated Scenario III-Undirected pleiotropy, 30% of all exposure-confounder combinations are generated to have a direct causal effect. Open parameters are the correlation between exposures $r_X = \{0, 0.2, 0.4, 0.6, 0.8\}$ and the pleiotropic effect $\theta^A = \{0.25, 0.5, 0.75, 1, 1.5, 2\}$. Confounding effects on the exposures and outcomes are fixed at $\theta^U_X = 2$ and $\theta^U_Y = 1$, respectively. Best results are highlighted in bold.

			θ^A					
			0.25	0.5	0.75	1	1.5	2
r_X	0	MV-MR	0.679 (0.255)	0.909 (0.332)	1.290 (0.456)	1.829 (0.687)	3.383 (1.331)	5.621 (2.265)
		MR-BMA	0.410 (0.183)	0.510 (0.214)	0.658 (0.274)	0.895 (0.358)	1.659 (0.603)	2.642 (0.885)
		MRCE	0.491 (0.193)	0.534 (0.210)	0.566 (0.219)	0.616 (0.23)	0.756 (0.298)	0.960 (0.422)
		mSSL	0.442 (0.244)	0.491 (0.296)	0.526 (0.271)	0.564 (0.266)	0.681 (0.367)	**0.858** (0.420)
		MR^2	**0.366** (0.165)	**0.412** (0.166)	**0.453** (0.187)	**0.507** (0.205)	**0.588** (0.252)	0.938 (1.021)
	0.2	MV-MR	0.768 (0.363)	1.021 (0.438)	1.465 (0.607)	2.080 (0.857)	3.767 (1.538)	6.349 (2.649)
		MR-BMA	0.430 (0.199)	0.560 (0.236)	0.786 (0.340)	1.088 (0.500)	1.818 (0.799)	2.849 (1.118)
		MRCE	0.540 (0.272)	0.584 (0.278)	0.659 (0.306)	0.722 (0.326)	0.893 (0.374)	1.062 (0.437)
		mSSL	0.484 (0.326)	0.591 (0.485)	0.658 (0.440)	0.696 (0.451)	0.810 (0.415)	**0.989** (0.513)
		MR^2	**0.371** (0.195)	**0.424** (0.221)	**0.496** (0.227)	**0.546** (0.249)	**0.673** (0.359)	1.033 (0.996)
	0.4	MV-MR	0.924 (0.393)	1.245 (0.500)	1.780 (0.695)	2.541 (0.989)	4.782 (1.885)	8.228 (3.153)
		MR-BMA	0.508 (0.244)	0.624 (0.260)	0.819 (0.348)	1.091 (0.495)	1.970 (0.899)	3.301 (1.250)
		MRCE	0.645 (0.296)	0.699 (0.305)	0.760 (0.317)	0.850 (0.359)	1.050 (0.500)	1.260 (0.640)
		mSSL	0.546 (0.279)	0.608 (0.286)	0.678 (0.285)	0.709 (0.296)	0.853 (0.347)	1.167 (0.695)
		MR^2	**0.430** (0.235)	**0.480** (0.243)	**0.526** (0.258)	**0.593** (0.275)	**0.827** (0.765)	**0.994** (0.696)
	0.6	MV-MR	1.294 (0.618)	1.764 (0.755)	2.548 (1.036)	3.613 (1.409)	6.802 (2.678)	11.567 (4.625)
		MR-BMA	0.732 (0.451)	0.876 (0.481)	1.156 (0.570)	1.508 (0.663)	2.581 (1.055)	4.122 (1.611)
		MRCE	0.903 (0.516)	0.975 (0.549)	1.072 (0.589)	1.172 (0.618)	1.406 (0.719)	1.683 (0.702)
		mSSL	0.835 (0.669)	0.882 (0.658)	0.946 (0.676)	1.050 (0.700)	1.261 (0.788)	1.637 (0.883)
		MR^2	**0.593** (0.432)	**0.677** (0.518)	**0.748** (0.605)	**0.821** (0.681)	**1.041** (0.855)	**1.420** (1.064)
	0.8	MV-MR	2.661 (1.249)	3.515 (1.528)	5.054 (2.103)	7.234 (2.824)	13.513 (5.142)	22.485 (8.544)
		MR-BMA	1.606 (0.691)	1.892 (0.819)	2.381 (0.975)	3.102 (1.272)	5.314 (2.453)	7.718 (3.558)
		MRCE	1.791 (0.732)	1.892 (0.770)	2.100 (0.867)	2.257 (0.940)	2.685 (1.216)	3.140 (1.419)
		mSSL	1.393 (0.757)	1.651 (0.982)	1.894 (1.155)	2.103 (1.246)	2.839 (1.726)	3.259 (1.913)
		MR^2	**1.205** (0.588)	**1.360** (0.604)	**1.514** (0.716)	**1.703** (0.766)	**2.156** (1.014)	**2.689** (1.387)

Table S10. Simulation IV-Directed pleiotropy: Sum of squared errors (SSE) of the simulated scenario, averaged over 50 replicates, with standard deviation across 50 replicates in brackets, illustrating the performance of different MR multivariable implementations and multi-response multivariable methods to estimate the true effect size. In simulated Scenario IV-Directed pleiotropy, 30% of all exposure-confounder combinations are generated to have a direct causal effect. Open parameters are the correlation between exposures $r_X = \{0, 0.2, 0.4, 0.6, 0.8\}$ and the pleiotropic effect $\theta^A = \{0.25, 0.5, 0.75, 1, 1.5, 2\}$. Confounding effects on the exposures and outcomes are fixed at $\theta_X^U = 2$ and $\theta_Y^U = 1$, respectively. Best results are highlighted in bold.

			r_Y				
			0	0.2	0.4	0.6	0.8
r_X	0	MV-MR	0.611 (0.246)	0.663 (0.315)	0.658 (0.296)	0.667 (0.305)	0.653 (0.308)
		MR-BMA	0.347 (0.163)	0.363 (0.216)	0.361 (0.207)	0.372 (0.203)	0.357 (0.193)
		MRCE	0.449 (0.196)	0.475 (0.254)	0.462 (0.227)	0.463 (0.235)	0.427 (0.228)
		mSSL	0.577 (0.831)	0.582 (0.858)	0.694 (1.053)	0.664 (1.062)	0.623 (1.036)
		MR^2	**0.315** (0.143)	**0.329** (0.207)	**0.325** (0.216)	**0.305** (0.189)	**0.268** (0.183)
	0.2	MV-MR	0.730 (0.322)	0.744 (0.298)	0.755 (0.279)	0.745 (0.276)	0.737 (0.278)
		MR-BMA	0.417 (0.220)	0.433 (0.219)	0.440 (0.218)	0.422 (0.210)	0.425 (0.217)
		MRCE	0.530 (0.268)	0.541 (0.252)	0.540 (0.234)	0.512 (0.216)	0.483 (0.230)
		mSSL	0.646 (0.966)	0.616 (0.964)	0.638 (0.964)	0.594 (0.969)	0.830 (1.773)
		MR^2	**0.378** (0.199)	**0.378** (0.197)	**0.385** (0.197)	**0.355** (0.202)	**0.321** (0.201)
	0.4	MV-MR	0.863 (0.385)	0.851 (0.348)	0.847 (0.340)	0.867 (0.346)	0.853 (0.343)
		MR-BMA	0.478 (0.235)	0.455 (0.221)	0.462 (0.215)	0.470 (0.224)	0.484 (0.234)
		MRCE	0.633 (0.321)	0.594 (0.264)	0.590 (0.236)	0.604 (0.258)	0.566 (0.227)
		mSSL	0.589 (0.340)	0.574 (0.405)	0.674 (0.868)	0.722 (1.014)	0.810 (1.406)
		MR^2	**0.430** (0.216)	**0.401** (0.201)	**0.388** (0.191)	**0.361** (0.161)	**0.334** (0.152)
	0.6	MV-MR	1.246 (0.597)	1.205 (0.519)	1.212 (0.514)	1.200 (0.464)	1.195 (0.470)
		MR-BMA	0.673 (0.373)	0.651 (0.329)	0.680 (0.369)	0.700 (0.348)	0.693 (0.337)
		MRCE	0.904 (0.516)	0.849 (0.413)	0.867 (0.431)	0.849 (0.423)	0.805 (0.355)
		mSSL	0.881 (1.117)	0.851 (1.095)	0.845 (1.168)	0.891 (1.167)	1.005 (1.762)
		MR^2	**0.567** (0.340)	**0.534** (0.289)	**0.545** (0.329)	**0.521** (0.278)	**0.438** (0.225)
	0.8	MV-MR	2.496 (1.192)	2.391 (0.968)	2.310 (0.942)	2.324 (0.939)	2.287 (0.979)
		MR-BMA	1.441 (0.807)	1.341 (0.587)	1.278 (0.525)	1.346 (0.570)	1.342 (0.635)
		MRCE	1.667 (0.806)	1.598 (0.630)	1.474 (0.545)	1.511 (0.609)	1.430 (0.642)
		mSSL	1.475 (1.113)	1.493 (1.566)	1.415 (1.533)	1.371 (1.517)	1.626 (2.440)
		MR^2	**1.057** (0.629)	**0.977** (0.475)	**0.867** (0.425)	**0.844** (0.450)	**0.754** (0.425)

Table S11. Simulation V-Dependence: Sum of squared errors (SSE) of the simulated scenario, averaged over 50 replicates, with standard deviation across 50 replicates in brackets, illustrating the performance of different MR multivariable implementations and multi-response multivariable methods to estimate the true effect size. In simulated Scenario V-Dependence, 30% of all exposure-confounder combinations are generated to have a direct causal effect. Open parameters are the correlation between exposures $r_X = \{0, 0.2, 0.4, 0.6, 0.8\}$ and the correlation between individual-level responses' errors $r_Y = \{0, 0.2, 0.4, 0.6, 0.8\}$. Confounding effects on the exposures and outcomes are fixed at $\theta_X^U = 2$ and $\theta_Y^U = 1$, respectively. Best results are highlighted in bold.

Simulated exposure effect	Average simulated proportion		Scenario II Confounding	Scenario III Undirected pleiotropy	Scenario IV Directed pleiotropy	Scenario V Dependence
Distinct	0.373	MV-MR	0.742 (0.437)	0.516 (0.500)	0.516 (0.500)	0.680 (0.467)
		MR-BMA	0.779 (0.415)	0.534 (0.499)	0.567 (0.496)	0.697 (0.460)
		mSSL	0.763 (0.426)	0.693 (0.461)	0.690 (0.463)	0.697 (0.460)
		MR^2	**0.783** (0.413)	**0.697** (0.460)	**0.722** (0.448)	**0.743** (0.437)
Shared = 2	0.315	MV-MR	**0.566** (0.496)	0.224 (0.417)	0.269 (0.444)	0.438 (0.496)
		MR-BMA	0.551 (0.498)	0.260 (0.439)	0.309 (0.462)	0.433 (0.496)
		mSSL	0.551 (0.498)	0.422 (0.494)	0.430 (0.495)	0.453 (0.498)
		MR^2	0.556 (0.496)	**0.435** (0.496)	**0.453** (0.498)	**0.463** (0.496)
Shared = 3	0.122	MV-MR	**0.443** (0.497)	0.173 (0.379)	0.173 (0.379)	0.265 (0.442)
		MR-BMA	0.381 (0.486)	0.212 (0.409)	0.240 (0.428)	0.286 (0.452)
		mSSL	0.412 (0.493)	0.298 (0.458)	0.337 (0.473)	**0.306** (0.461)
		MR^2	0.392 (0.489)	**0.308** (0.462)	**0.346** (0.476)	**0.306** (0.461)
Shared = 4	0.023	MV-MR	**0.440** (0.498)	0.133 (0.342)	0.133 (0.342)	0.231 (0.423)
		MR-BMA	0.400 (0.492)	0.200 (0.403)	0.133 (0.342)	0.269 (0.445)
		mSSL	**0.440** (0.498)	**0.267** (0.445)	**0.267** (0.445)	0.346 (0.478)
		MR^2	**0.440** (0.498)	0.233 (0.430)	**0.267** (0.445)	**0.385** (0.488)

Table S12. Power to detect distinct and shared direct causal effects at a common false positives rate in different simulated scenarios when the correlation between exposures is set at $r_X = 0.6$ over 50 replicates with standard deviation across 50 replicates in brackets. Simulated exposure effects are either distinct, where an exposure has only a causal association with one outcome or shared, where the exposure exerts its effect on multiple (2,3,4) responses (Shared = 5 was not simulated). The average simulated proportion indicates the simulated proportion (over all exposure-response combinations) that an exposure is associated with one (distinct) or multiple (shared) responses. In baseline Scenario II-Confounding, only the confounding effects on the exposures and outcomes, $\theta_X^U = 2$ and $\theta_Y^U = 1$, respectively, are used to induce residual correlation between the responses. Residual correlation induced by shared undirected pleiotropy and shared directed pleiotropy with pleiotropic effect $\theta^A = 1$ are presented in Scenario III-Undirected pleiotropy and Scenario IV-Directed pleiotropy, respectively. In Scenario IV-Dependence, outcomes are simulated with the correlation between individual-level responses' errors fixed at $r_Y = 0.6$. Best results are highlighted in bold. MRCE method is not considered since it is not possible to match its type I error with the other methods. For more details on the simulations setting, see Table S2.

S3.3 Supplemental figures

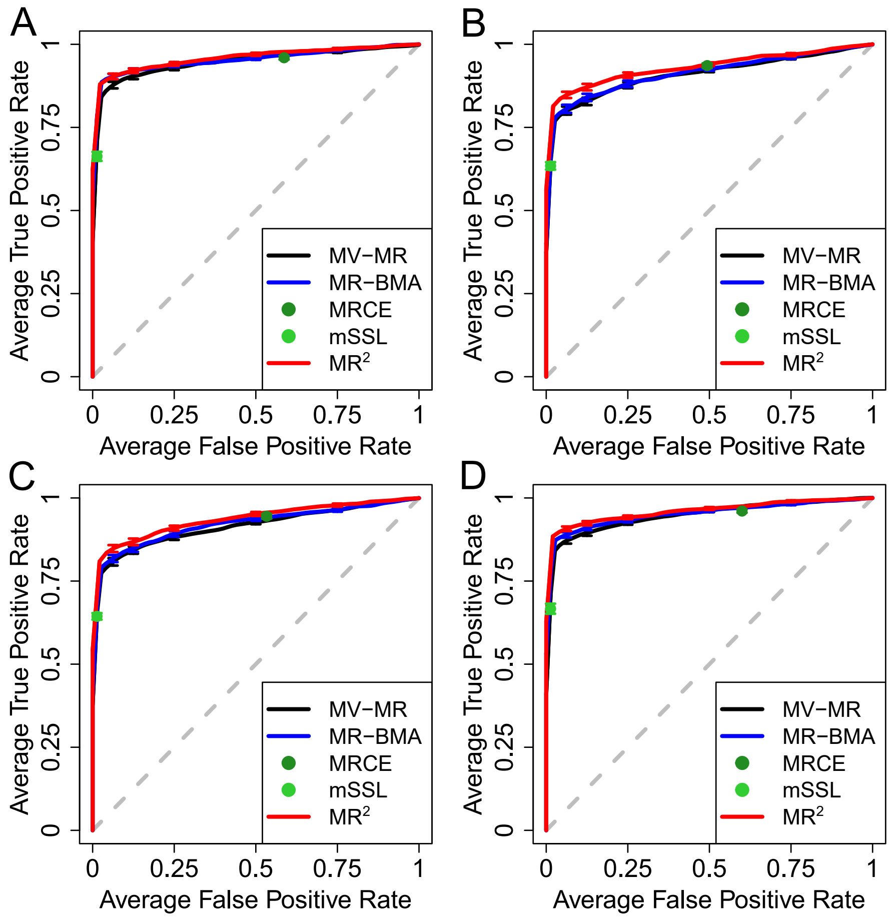

Figure S1. Receiver operating characteristic (ROC) curves in different simulated scenarios when there is no correlation between exposures, averaged over 50 replicates, illustrating the performance of different MR multivariable implementations and multi-response multivariable methods to distinguish between true and false causal exposures for the simulated outcomes by plotting the true (TPR) against the false positive rate (FPR). (**A**) depicts baseline Scenario II-Confounding where only the confounding effects on the exposures and outcomes, $\theta_X^U = 2$ and $\theta_Y^U = 1$, respectively, are used to simulate the data. Residual correlation induced by shared undirected pleiotropy (Scenario III-Undirected pleiotropy) and shared directed pleiotropy (Scenario IV-Directed pleiotropy) with pleiotropic effect set at $\theta^A = 1$ are shown in (**B**) and (**C**), respectively. (**D**) displays the performance of the different methods in Scenario IV-Dependence, where the correlation between individual-level responses' errors is fixed at $r_Y = 0.6$. Vertical bars in each ROC curve, at specific FPR levels, indicate standard error. For more details on the simulations setting, see Table S2.

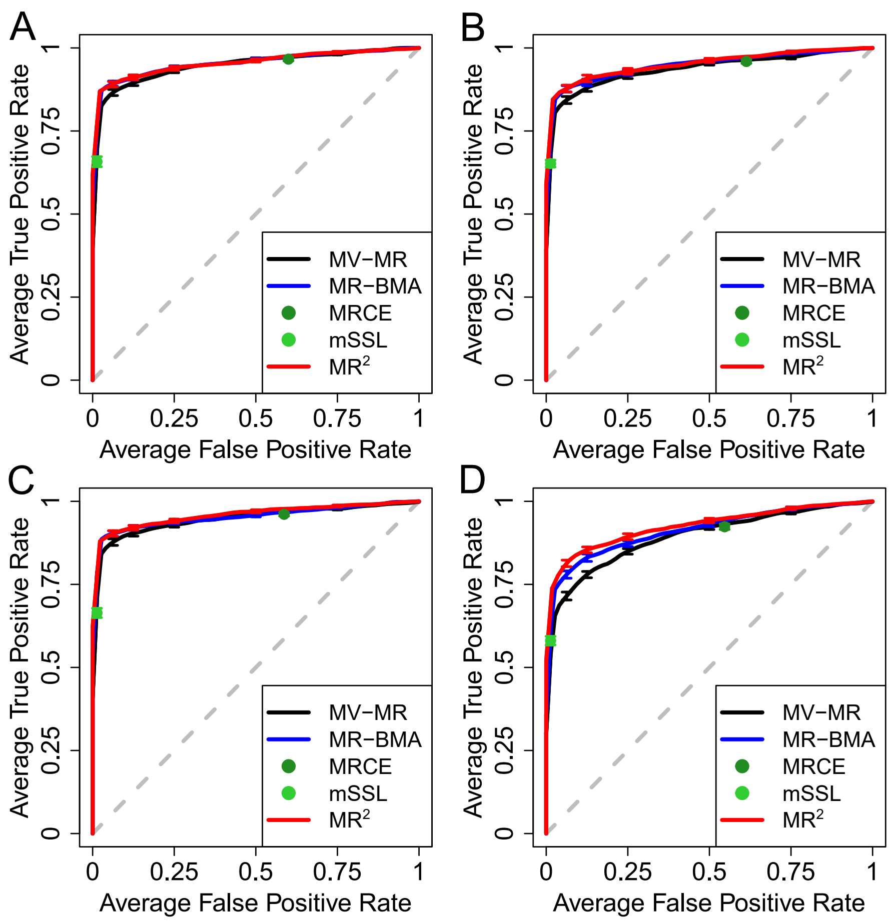

Figure S2. Simulation II-Confounding: Receiver operating characteristic (ROC) for different levels of correlation between the exposures, averaged over 50 replicates, illustrating the performance of different MR multivariable implementations and multi-response multivariable methods to distinguish between true and false causal exposures for the simulated outcomes by plotting the true (TPR) against the false positive rate (FPR). Correlation between the exposures varies from light correlation $r_X = 0.2$ in (**A**) to strong correlation $r_X = 0.8$ in (**D**) with confounding effects on the exposures and outcomes fixed at $\theta_X^U = 2$ and $\theta_Y^U = 1$, respectively. Vertical bars in each ROC curve, at specific FPR levels, indicate standard error.

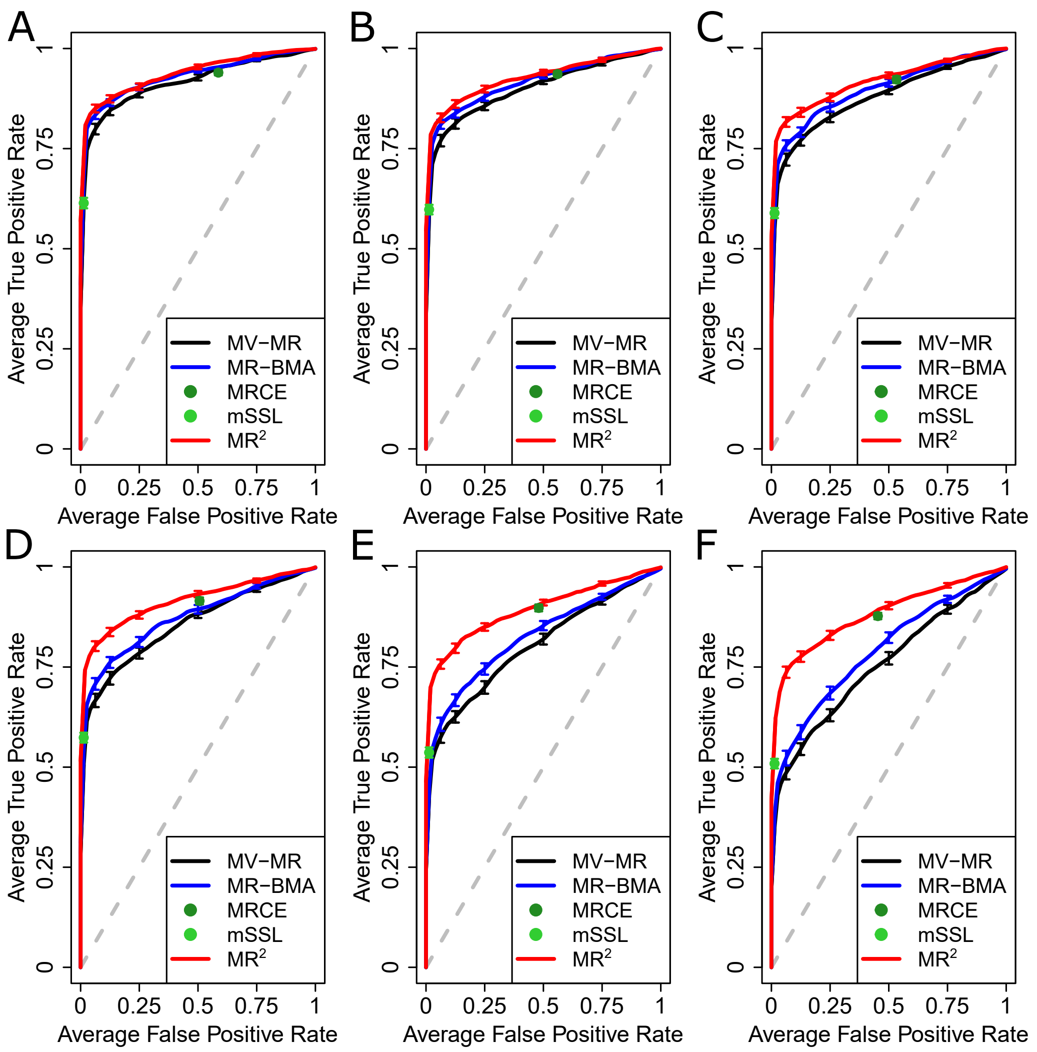

Figure S3. Simulation IV-Directed pleiotropy: Receiver operating characteristic (ROC) curves for different levels of the pleiotropic pathway effect θ^A and when the correlation between exposures is set at $r_X = 0.6$, averaged over 50 replicates, illustrating the performance of different MR implementations and multi-response statistical methods to distinguish between true and false causal exposures for the simulated outcomes by plotting the true (TPR) against the false positive rate (FPR). Pleiotropic pathway effect varies from (**A**) to (**F**) with values $\theta^A = \{0.25, 0.5, 0.75, 1, 1.5, 2\}$. Confounding effects on the exposures and outcomes are fixed at $\theta_X^U = 2$ and $\theta_Y^U = 1$, respectively. Vertical bars in each ROC curve, at specific FPR levels, indicate standard error.

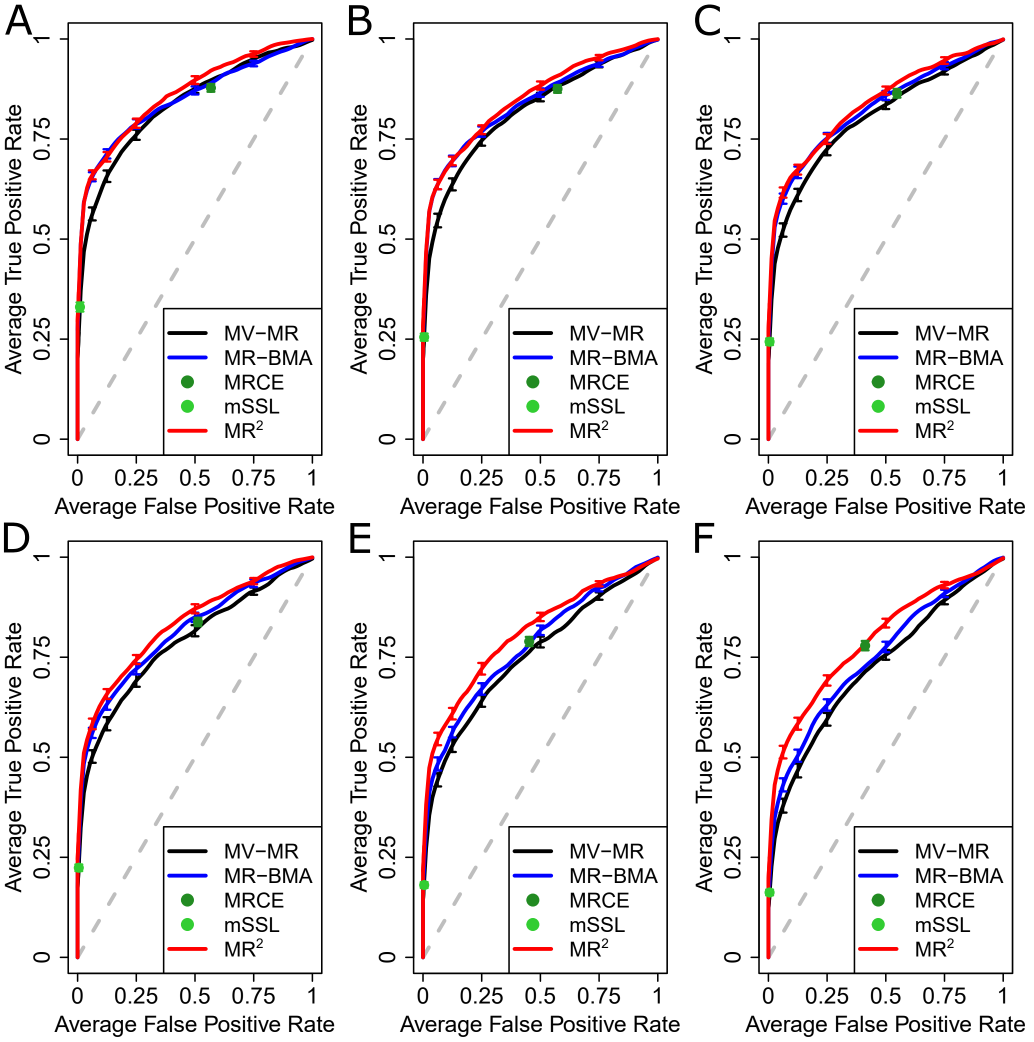

Figure S4. Simulation III-Undirected pleiotropy: Receiver operating characteristic (ROC) curves for different levels of the pleiotropic pathway effect $\boldsymbol{\theta^A}$ and when the correlation between exposures is set at $\boldsymbol{r_X = 0.6}$ and the number of individuals at the raw-level at $\boldsymbol{N = 20,000}$ of which half is used to generate the summary-level genetic associations for the exposure and half for the outcomes, averaged over 50 replicates, illustrating the performance of different MR implementations and multi-response statistical methods to distinguish between true and false causal exposures for the simulated outcomes by plotting the true (TPR) against the false positive rate (FPR). Pleiotropic pathway effect varies from (**A**) to (**F**) with values $\theta^A = \{0.25, 0.5, 0.75, 1, 1.5, 2\}$. Confounding effects on the exposures and outcomes are fixed at $\theta_X^U = 2$ and $\theta_Y^U = 1$, respectively. Vertical bars in each ROC curve, at specific FPR levels, indicate standard error.

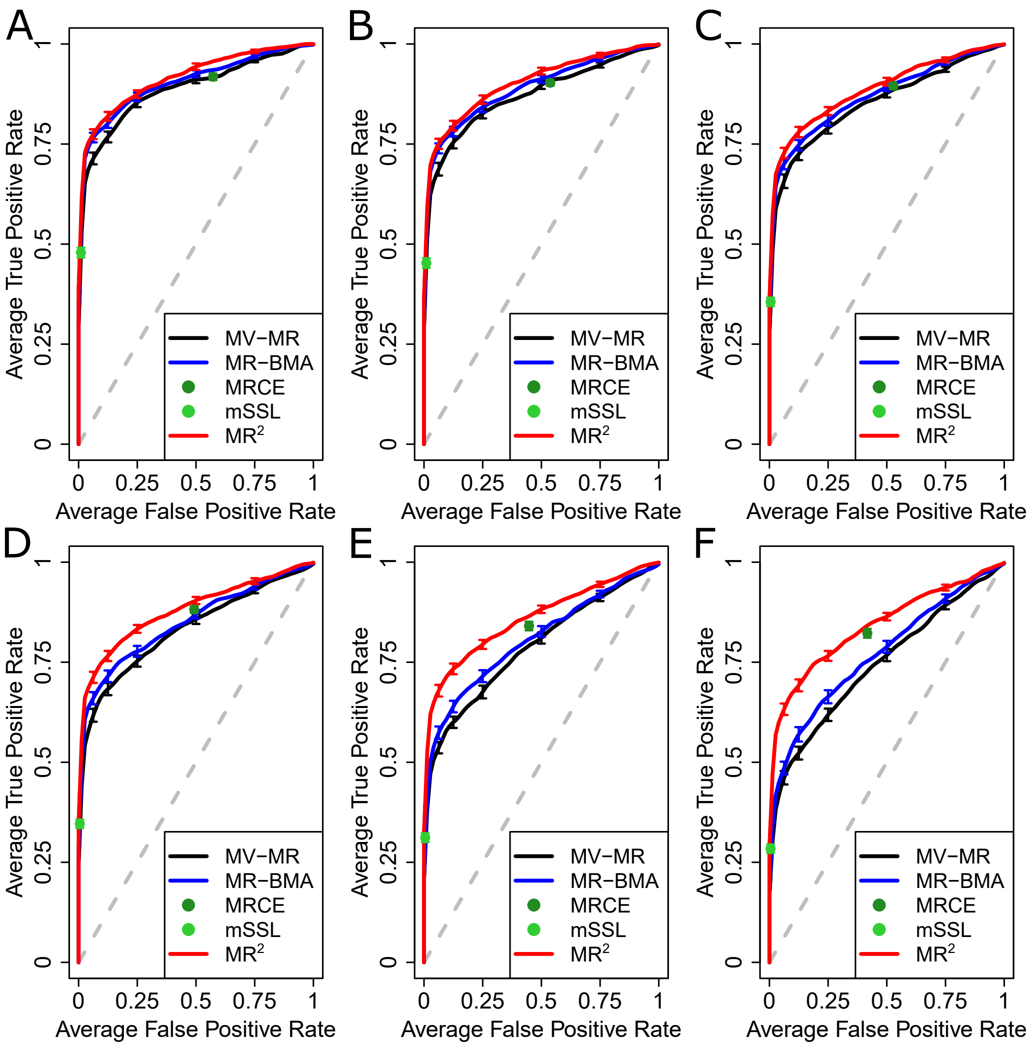

Figure S5. Simulation III-Undirected pleiotropy: Receiver operating characteristic (ROC) curves for different levels of the pleiotropic pathway effect $\boldsymbol{\theta^A}$ and when the correlation between exposures is set at $\boldsymbol{r_X = 0.6}$ and the heritability of the exposures at $\boldsymbol{h_X = 0.05}$, averaged over 50 replicates, illustrating the performance of different MR implementations and multi-response statistical methods to distinguish between true and false causal exposures for the simulated outcomes by plotting the true (TPR) against the false positive rate (FPR). Pleiotropic pathway effect varies from (**A**) to (**F**) with values $\theta^A = \{0.25, 0.5, 0.75, 1, 1.5, 2\}$. Confounding effects on the exposures and outcomes are fixed at $\theta^U_X = 2$ and $\theta^U_Y = 1$, respectively. Vertical bars in each ROC curve, at specific FPR levels, indicate standard error.

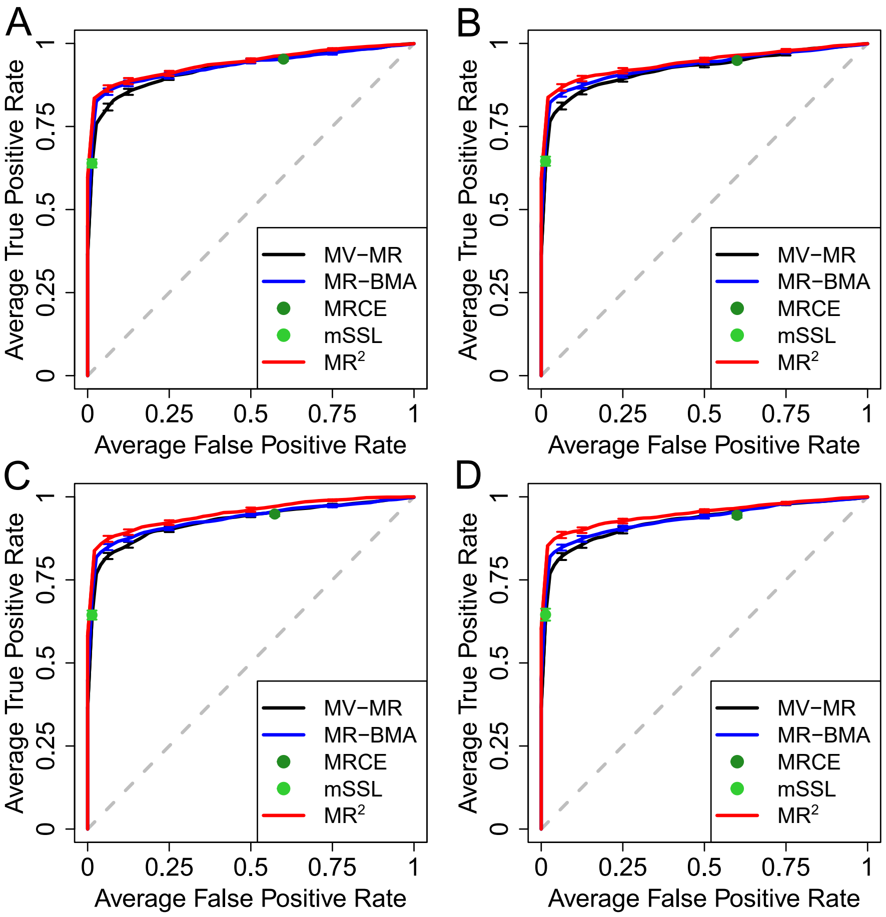

Figure S6. Simulation V-Dependence: Receiver operating characteristic (ROC) curves for different levels of the correlation between individual-level responses' errors, averaged over 50 replicates, illustrating the performance of different MR multivariable implementations and multi-response multivariable methods to distinguish between true and false causal exposures for the simulated outcomes by plotting the true (TPR) against the false positive rate (FPR). Individual-level correlation between the responses' errors varies from (**A**) to (**D**) with values $r_Y = \{0.2, 0.4, 0.6, 0.8\}$. Correlation between exposures is set at $r_X = 0.6$ and confounding effects on the exposures and outcomes are fixed at $\theta_X^U = 2$ and $\theta_Y^U = 1$, respectively. Vertical bars in each ROC curve, at specific FPR levels, indicate standard error.

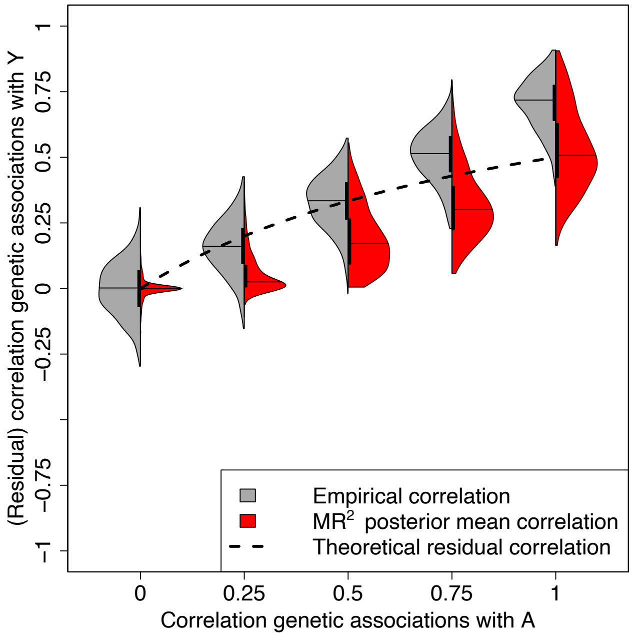

Figure S7. Residual correlation between the summary-level genetic associations with the outcomes induced by various levels of correlation of the genetic associations with the pleiotropic pathways A and with no sample overlap between the summary-level data of the outcomes. Each violin plot depicts the posterior mean of the residual correlations estimated by MR^2 in a simulation scenario similar to Scenario III-Undirected pleiotropy averaged over 50 replicates at different levels of the correlation ρ^A in eq. (S5) between the genetic associations with the pleiotropy pathways A while keeping $\theta^A = 1$ constant. The level of correlation is set at $\rho^A = \{0, 0.25, 0.50, 0.75, 0.99\}$. Confounding effects on the exposures and outcomes are fixed at $\theta^U_X = 2$ and $\theta^U_Y = 1$, respectively, without correlation between exposures and no sample overlap in the summary-level genetic associations with the outcomes. For each side of the violin plot, the vertical black thick line displays the interquartile range, while the black line indicates the median. Black dashed line depicts the theoretical value of residual correlation between summary-level outcomes as a function of ρ^A as shown in eq. (S4). For more details on the simulation setting, see Table S2.

S4 Application examples

S4.1 Data pre-processing

Application examples are based on publicly available summary-level data. An overview of the data sets, with sample sizes and references, is provided in Table S13.

The first application example considers ten common exposures for five cardiovascular disease outcomes (CVDs). The first step of the data-processing merges the summary-level data (beta regression coefficients, their standard errors and associated p-values) of all exposures by their unique “rs” identifier and aligns the effect direction of the genetic associations with each exposure according to the same effect allele. As IVs, we select any genetic variant which is associated with any of the exposures at genome-wide significance (p-value $< 5 \times 10^{-8}$). Next, we merge the genetic variants selected as IVs with the outcome data of the five CVDs by their unique “rs” identifier and aligned the effect direction of the genetic associations with each outcome according to the same effect allele. Finally, we clump the genetic variants to be independent at $r^2 < 0.001$ using a European reference panel [12]. This results in $n = 1,540$ independent genetic variants selected as IVs.

The second application example focuses on ten ApoB-containing lipoprotein subfractions derived from metabolic GWAS using the Nightingale nuclear magnetic resonance spectroscopy platform derived from fasting samples of $24,925$ European descent participants [13]. Instruments selection is based on an external data set. Specifically, given the prior hypothesis of ApoB as the leading exposure for CVDs, we select genetic variants associated with ApoB in UK Biobank at genome-wide significance (p-value $< 5 \times 10^{-8}$). Exposures and outcomes summary-level data are merged by their unique “rs” identifier and effect alleles are aligned. Finally, we clump the genetic variants to be independent at $r^2 < 0.001$ using a European reference panel [14]. This resulted in $n = 148$ IVs.

In both application examples, IVW is performed before the analysis for all outcomes and exposures using weights derived jointly from all responses as described in Appendix. Moreover, the summary-level genetic associations with the exposures are standardised before the analysis. This is usually required in (multi-response) multivariable regression models since the summary-level genetic associations with the exposures should be on the same scale in order to interpret and compare the estimated effect sizes.

S4.2 Analysis plan and comparisons with alternative methods

MR^2 parameters setting

In the first application example, the MR^2 algorithm is run for $75,000$ MCMC iterations of which $25,000$ as burn-in and results saved every 50 iterations, resulting in $1,000$ posterior samples for all the unknowns. In the second application example, we run the algorithm for $150,000$ MCMC iterations of which $50,000$ as burn-in with $1,000$ posterior samples saved. As illustrated in Figures S9 and S14, the burn-in phase and the total length of the MCMC far exceed the number of iterations required to reach convergence to the equilibrium distribution of the Markov chain and explore faithfully the posterior parameters space. No signs of slow convergence or aberrant behaviour of the designed MCMC sampler are also apparent.

In both application examples, the hyper-parameters a_k and b_k in eq. (15) are chosen by specifying $\mathbb{E}(\gamma_k) = 2$ and $\mathbb{V}(\gamma_k) = 2$ which imply *a priori* a range between zero and seven for the number of significant direct causal effects for each outcome. Sensitivity analysis shows that the *a priori* range can also be relaxed ($\mathbb{E}(\gamma_k) = 4$ and $\mathbb{V}(\gamma_k) = 4$) without noticeable differences in the posterior mean of the direct causal effects, see Figures S12 and S17. We also set $v = 1$ in eq. (13) after standardising the summary-level genetic associations with the IVW transformed exposures in order to place appropriate prior mass on reasonable values of the non-zero direct causal effects [8].

We use Congdon [15] definition of outliers or high-leverage and influential observations and report the number of standardised CPOs less than 0.01 to check model adequacy. After excluding genetic variants with standardised CPOs below this threshold, we refit the model without repeating the procedure even if new outliers or high-leverage and influential observations are detected, see Figures S10 and S15.

Finally, the FDR level is set at 5% to select important mPPIs and ePPIs, see Figures S11A and B and S16A and B.

MR alternative methods and parameters setting

MR-BMA is run for each outcome separately with a prior probability of inclusion of $2/10 = 0.2$ in application example 1, using ten common exposures for CVDs, as well as in application example 2, using ten molecular risk factors for the same disease outcomes, in order to match the sparsity prior used in MR^2. An exhaustive search evaluating all combinations of exposures in both applications is conducted and no stochastic search is necessary. Because

summary-level genetic associations with the exposures are standardised prior to the analysis, the prior variance on the direct causal effects is set at 1.

All results presented are generated after removing any influential or outlying genetic variant used as IV. Influential genetic variants are detected using the Cook's distance [12] and a cut-off is chosen according to the median of the F-distribution with d and $n - d$ degrees of freedom, where n is the number of IVs and d is the size of the best visited model in the exhaustive search.

Outlying genetic variants are identified using the q-heterogeneity statistic [12] and threshold using a χ^2 distribution with one degree of freedom and a Bonferroni-adjusted multiple testing correction. Empirical p-values for the marginal inclusion probabilities are calculated using a permutation procedure with $100,000$ permutations [14]. Multiple testing adjustment is performed using Benjamini-Hochberg [16] FDR and Bonferroni correction.

MV-MR [9] is applied on the summary-level data after removing outliers or influential variants and multiple testing adjustment is performed using Benjamini-Hochberg FDR and Bonferroni correction.

S4.3 Supplemental tables

	Response or risk factor	Acronym	Reference	Number cases & controls	Data source
Cardiovascular disease outcomes:	Atrial fibrillation	AF	Nielsen *et al.* 2018 [17]	60,620 cases & 970,216 controls	AFGen Cons., HUNT, DECODE, MGI, DiscovEHR + UK Biobank
	Cardioembolic stroke	CES	Mishra *et al.* 2022 [18]	10,804 cases & 1,234,808 controls	GIGASTROKE consortium + Global Biobanks (UK Biobank and Million Veteran Program)
	Coronary artery disease	CAD	Nelson *et al.* 2017 [19]	113,937 cases & 339,658 controls	CARDIOGRAM Cons. + UK Biobank
	Peripheral artery disease	PAD	Klarin *et al.* 2019 [20]	31,307 cases & 211,753 controls	Million Veteran Program
	Heart failure	HF	Shah *et al.* 2020 [21]	47,309 cases & 930,014 controls	HERMES Cons. + UK Biobank
Exposures in application example 1: Common exposures	Apolipoprotein A	ApoA	Nealelab 2018 [22]		UK Biobank
	Apolipoprotein B	ApoB			UK Biobank
	Body mass index	BMI			UK Biobank
	High-density lipoprotein	HDL			UK Biobank
	Low-density lipoprotein	LDL			UK Biobank
	Systolic blood pressure	SBP			UK Biobank
	Triglycerides	TG			UK Biobank
	Physical activity	PA	Wang *et al.* 2022 [23]	636,924 cases	UK Biobank
	Smoking	Smoking	Wootton *et al.* 2020 [24]	462,690 cases	UK Biobank
	Type 2 diabetes	T2D	Mahajan *et al.* 2018 [25]	74,124 cases & 824,006 controls	UK Biobank
Exposures in application example 2: Molecular risk factors	Medium large-density lipoprotein particles	M.LDL.P	Kettunen *et al.* 2016 [13]	24,925 controls	Meta-analysis of Finnish and European cohorts
	Large large-density lipoprotein particles	L.LDL.P			Meta-analysis of Finnish and European cohorts
	Intermediate-density lipoprotein particles	IDL.P			Meta-analysis of Finnish and European cohorts
	Extra small very-large density lipoprotein particles	XS.VLDL.P			Meta-analysis of Finnish and European cohorts
	Small very-large density lipoprotein particles	S.VLDL.P			Meta-analysis of Finnish and European cohorts
	Medium very-large density lipoprotein particles	M.VLDL.P			Meta-analysis of Finnish and European cohorts
	Large very-large density lipoprotein particles	L.VLDL.P			Meta-analysis of Finnish and European cohorts
	Extra large very-large density lipoprotein particles	XL.VLDL.P			Meta-analysis of Finnish and European cohorts
	Extra-extra large very-large density lipoprotein particles	XXL.VLDL.P			Meta-analysis of Finnish and European cohorts

Table S13. Overview of summary-level data in the two application examples. Common exposures (application example 1) and molecular risk factors (application example 2) for cardiovascular disease outcomes.

Outcome	Ranking	Exposure	Effect estimate	Standard error	p-value	FDR	Bonferroni
CAD	1	**SBP**	0.621	0.041	1.45×10^{-47}	1.45×10^{-46}	1.45×10^{-46}
	2	**T2D**	0.125	0.013	1.69×10^{-20}	8.43×10^{-20}	1.69×10^{-19}
	3	**SMOKING**	0.507	0.093	5.98×10^{-8}	1.99×10^{-7}	5.98×10^{-7}
	4	**BMI**	0.094	0.034	0.005	0.013	0.053
	5	TG	0.083	0.038	0.029	0.058	0.292
	6	LDL	0.251	0.136	0.065	0.105	0.650
	7	ApoA	-0.159	0.089	0.074	0.105	0.737
	8	ApoB	0.178	0.121	0.141	0.176	1.407
	9	HDL	0.084	0.094	0.372	0.414	3.723
	10	PA	0.001	0.050	0.978	0.978	9.779
PAD	1	**T2D**	0.161	0.014	7.91×10^{-30}	7.91×10^{-29}	7.91×10^{-29}
	2	**SMOKING**	0.789	0.098	1.30×10^{-15}	6.48×10^{-15}	1.30×10^{-14}
	3	**SBP**	0.326	0.043	7.81×10^{-14}	2.60×10^{-13}	7.81×10^{-13}
	4	**BMI**	0.155	0.035	1.16×10^{-5}	2.91×10^{-5}	1.16×10^{-4}
	5	TG	0.074	0.040	0.063	0.126	0.632
	6	ApoA	-0.130	0.093	0.163	0.271	1.626
	7	ApoB	0.123	0.127	0.334	0.477	3.340
	8	HDL	0.082	0.099	0.411	0.513	4.106
	9	LDL	0.035	0.143	0.807	0.818	8.067
	10	PA	0.012	0.053	0.818	0.818	8.182
HF	1	**BMI**	0.318	0.030	1.54×10^{-25}	1.54×10^{-24}	1.54×10^{-24}
	2	**SBP**	0.322	0.036	2.80×10^{-18}	1.40×10^{-17}	2.80×10^{-17}
	3	**T2D**	0.047	0.012	5.63×10^{-5}	1.88×10^{-4}	5.63×10^{-4}
	4	**SMOKING**	0.323	0.083	9.77×10^{-5}	2.44×10^{-4}	9.77×10^{-4}
	5	ApoB	0.113	0.108	0.296	0.584	2.958
	6	HDL	-0.072	0.084	0.390	0.584	3.904
	7	PA	0.037	0.045	0.409	0.584	4.088
	8	ApoA	0.048	0.079	0.540	0.674	5.396
	9	LDL	0.015	0.121	0.903	0.983	9.026
	10	TG	-0.001	0.034	0.983	0.983	9.829
AF	1	**SBP**	0.286	0.036	7.71×10^{-15}	7.71×10^{-14}	7.71×10^{-14}
	2	**BMI**	0.225	0.030	5.83×10^{-14}	2.92×10^{-13}	5.83×10^{-13}
	3	**TG**	-0.129	0.034	1.39×10^{-4}	4.62×10^{-4}	1.39×10^{-03}
	4	SMOKING	0.140	0.082	0.090	0.187	0.899
	5	ApoB	0.180	0.107	0.094	0.187	0.938
	6	HDL	-0.133	0.084	0.112	0.187	1.123
	7	ApoA	0.108	0.079	0.172	0.245	1.717
	8	LDL	-0.151	0.120	0.210	0.263	2.103
	9	T2D	-0.009	0.012	0.427	0.439	4.271
	10	PA	0.035	0.045	0.439	0.439	4.392
CES	1	**SBP**	0.266	0.063	2.76×10^{-5}	2.76×10^{-4}	2.76×10^{-4}
	2	**T2D**	0.064	0.020	0.001	0.007	0.015
	3	**SMOKING**	0.345	0.141	0.014	0.048	0.143
	4	TG	-0.117	0.058	0.044	0.111	0.444
	5	PA	0.088	0.077	0.252	0.467	2.517
	6	ApoB	0.199	0.184	0.280	0.467	2.803
	7	LDL	-0.177	0.208	0.395	0.564	3.948
	8	HDL	-0.056	0.144	0.698	0.867	6.976
	9	ApoA	0.038	0.135	0.780	0.867	7.804
	10	BMI	-0.001	0.051	0.979	0.979	9.792

Table S14. Application example 1 on common exposures for cardiovascular disease outcomes using standard multivariable MR (MV-MR) which is run on each outcome separately. Results include the causal effect estimates, the standard error and the corresponding p-value. We adjust for multiple testing using Benjamini-Hochberg False Discovery Rate (FDR) and Bonferroni correction. For each outcome, we sort the exposures by their p-value in increasing order. Exposures selected at 5% FDR highlighted in bold.

Outcome	Ranking	Exposure	Effect estimate	Standard error	p-value	FDR	Bonferroni
CAD		(Intercept)	-0.024	0.029	0.401	0.401	-
	1	**SBP**	0.621	0.041	1.53×10^{-47}	1.53×10^{-46}	1.53×10^{-46}
	2	**T2D**	0.125	0.013	1.46×10^{-20}	7.31×10^{-20}	1.46×10^{-19}
	3	**SMOKING**	0.506	0.093	6.37×10^{-8}	2.12×10^{-7}	6.37×10^{-7}
	4	**BMI**	0.094	0.034	0.005	0.013	0.051
	5	TG	0.083	0.038	0.030	0.059	0.296
	6	LDL	0.253	0.136	0.064	0.105	0.636
	7	ApoA	-0.159	0.089	0.073	0.105	0.732
	8	ApoB	0.177	0.121	0.144	0.180	1.000
	9	HDL	0.085	0.094	0.371	0.412	1.000
	10	PA	-0.001	0.050	0.985	0.985	1.000
PAD		(Intercept)	0.004	0.030	0.883	0.883	-
	1	**T2D**	0.161	0.014	8.79×10^{-30}	8.79×10^{-29}	8.79×10^{-29}
	2	**SMOKING**	0.790	0.098	1.31×10^{-15}	6.55×10^{-15}	1.31×10^{-14}
	3	**SBP**	0.326	0.043	7.92×10^{-14}	2.64×10^{-13}	7.92×10^{-13}
	4	**BMI**	0.155	0.035	1.19×10^{-5}	2.97×10^{-5}	1.19×10^{-4}
	5	TG	0.074	0.040	0.063	0.126	0.631
	6	ApoA	-0.130	0.093	0.163	0.271	1.000
	7	ApoB	0.123	0.127	0.333	0.476	1.000
	8	HDL	0.082	0.099	0.411	0.513	1.000
	9	LDL	0.035	0.143	0.808	0.812	1.000
	10	PA	0.013	0.053	0.812	0.812	1.000
HF		(Intercept)	0.000	0.025	0.990	0.990	-
	1	**BMI**	0.318	0.030	1.63×10^{-25}	1.63×10^{-24}	1.63×10^{-24}
	2	**SBP**	0.322	0.036	2.87×10^{-18}	1.44×10^{-17}	2.87×10^{-17}
	3	**T2D**	0.047	0.012	5.68×10^{-5}	1.89×10^{-4}	5.68×10^{-4}
	4	**SMOKING**	0.323	0.083	9.85×10^{-5}	2.46×10^{-4}	0.001
	5	ApoB	0.113	0.108	0.296	0.586	1.000
	6	HDL	-0.072	0.084	0.391	0.586	1.000
	7	PA	0.037	0.045	0.410	0.586	1.000
	8	ApoA	0.048	0.079	0.540	0.675	1.000
	9	LDL	0.015	0.121	0.903	0.983	1.000
	10	TG	-0.001	0.034	0.983	0.983	1.000
AF		(Intercept)	0.020	0.025	0.436	0.436	-
	1	**SBP**	0.286	0.036	7.62×10^{-15}	7.62×10^{-14}	7.62×10^{-14}
	2	**BMI**	0.225	0.030	6.65×10^{-14}	3.33×10^{-13}	6.65×10^{-13}
	3	**TG**	-0.129	0.034	1.43×10^{-4}	4.76×10^{-4}	0.001
	4	SMOKING	0.141	0.082	0.088	0.184	0.877
	5	ApoB	0.181	0.107	0.092	0.184	0.920
	6	HDL	-0.133	0.084	0.112	0.187	1.000
	7	ApoA	0.108	0.079	0.171	0.245	1.000
	8	LDL	-0.152	0.120	0.207	0.259	1.000
	9	T2D	-0.009	0.012	0.417	0.417	1.000
	10	PA	0.036	0.045	0.417	0.417	1.000
CES		**(Intercept)**	0.169	0.043	9.58×10^{-5}	9.58×10^{-5}	-
	1	**SBP**	0.265	0.063	2.56×10^{-5}	2.56×10^{-4}	2.56×10^{-4}
	2	**T2D**	0.062	0.020	0.002	0.009	0.019
	3	**SMOKING**	0.357	0.140	0.011	0.037	0.110
	4	TG	-0.116	0.058	0.044	0.109	0.438
	5	PA	0.106	0.077	0.168	0.336	1.000
	6	ApoB	0.204	0.183	0.265	0.441	1.000
	7	LDL	-0.181	0.207	0.384	0.548	1.000
	8	HDL	-0.060	0.143	0.675	0.844	1.000
	9	ApoA	0.041	0.134	0.760	0.844	1.000
	10	BMI	-0.004	0.051	0.936	0.936	1.000

Table S15. Application example 1 on common exposures for cardiovascular disease outcomes using multivariable MR-Egger which is run on each outcome separately. Results include the causal effect estimates as well as the intercept, the standard error and the corresponding p-value. We adjust for multiple testing using Benjamini-Hochberg False Discovery Rate (FDR) and Bonferroni correction. For each outcome, we sort the exposures by their p-value in increasing order. Exposures selected at 5% FDR highlighted in bold.

Outcome	Ranking	Exposure	MACE	mPPI	Empirical p-value	FDR	Bonferroni
CAD	1	**SBP**	0.626	1.000	1.00×10^{-5}	3.33×10^{-5}	1.00×10^{-4}
	2	**SMOKING**	0.560	1.000	1.00×10^{-5}	3.33×10^{-5}	1.00×10^{-4}
	3	**T2D**	0.137	1.000	1.00×10^{-5}	3.33×10^{-5}	1.00×10^{-4}
	4	**ApoB**	0.285	0.659	0.001	0.002	0.007
	5	**HDL**	-0.034	0.295	0.017	0.032	0.171
	6	**LDL**	0.161	0.344	0.022	0.032	0.219
	7	**BMI**	0.047	0.409	0.022	0.032	0.221
	8	ApoA	-0.020	0.185	0.062	0.078	0.624
	9	TG	0.002	0.033	0.902	1.000	1.000
	10	PA	0.000	0.002	1.000	1.000	1.000
PAD	1	**SMOKING**	0.787	1.000	1.00×10^{-5}	2.00×10^{-5}	1.00×10^{-4}
	2	**SBP**	0.325	1.000	1.00×10^{-5}	2.00×10^{-5}	1.00×10^{-4}
	3	**ApoB**	0.184	0.975	1.00×10^{-5}	2.00×10^{-5}	1.00×10^{-4}
	4	**BMI**	0.174	0.998	1.00×10^{-5}	2.00×10^{-5}	1.00×10^{-4}
	5	**T2D**	0.171	1.000	1.00×10^{-5}	2.00×10^{-5}	1.00×10^{-4}
	6	LDL	0.005	0.025	0.978	1.000	1.000
	7	TG	0.000	0.000	1.000	1.000	1.000
	8	PA	0.000	0.000	1.000	1.000	1.000
	9	ApoA	0.000	0.001	1.000	1.000	1.000
	10	HDL	0.000	0.001	1.000	1.000	1.000
HF	1	**SBP**	0.324	1.000	1.00×10^{-5}	5.00×10^{-5}	1.00×10^{-4}
	2	**BMI**	0.324	1.000	1.00×10^{-5}	5.00×10^{-5}	1.00×10^{-4}
	3	**ApoB**	0.117	0.897	2.00×10^{-5}	6.67×10^{-5}	2.00×10^{-4}
	4	**T2D**	0.052	0.993	4.00×10^{-5}	1.00×10^{-4}	4.00×10^{-4}
	5	**SMOKING**	0.323	0.991	2.90×10^{-4}	0.001	0.003
	6	LDL	0.014	0.104	0.267	0.445	1.000
	7	TG	0.000	0.001	1.000	1.000	1.000
	8	PA	0.000	0.000	1.000	1.000	1.000
	9	ApoA	0.000	0.000	1.000	1.000	1.000
	10	HDL	0.000	0.001	1.000	1.000	1.000
AF	1	**SBP**	0.281	1.000	1.00×10^{-5}	5.00×10^{-5}	1.00×10^{-4}
	2	**BMI**	0.233	1.000	1.00×10^{-5}	5.00×10^{-5}	1.00×10^{-4}
	3	**TG**	-0.035	0.520	0.009	0.031	0.094
	4	HDL	-0.002	0.048	0.700	0.995	1.000
	5	SMOKING	0.018	0.132	0.870	0.995	1.000
	6	ApoB	0.003	0.040	0.878	0.995	1.000
	7	ApoA	-0.001	0.030	0.923	0.995	1.000
	8	LDL	-0.002	0.027	0.968	0.995	1.000
	9	T2D	0.000	0.008	0.988	0.995	1.000
	10	PA	0.001	0.028	0.995	0.995	1.000
CES	1	**SBP**	0.295	1.000	1.00×10^{-5}	5.00×10^{-5}	1.00×10^{-4}
	2	**BMI**	0.217	1.000	1.00×10^{-5}	5.00×10^{-5}	1.00×10^{-4}
	3	TG	-0.013	0.232	0.048	0.160	0.481
	4	SMOKING	0.041	0.244	0.381	0.953	1.000
	5	HDL	-0.001	0.025	0.947	0.990	1.000
	6	ApoA	-0.001	0.024	0.960	0.990	1.000
	7	ApoB	0.002	0.024	0.970	0.990	1.000
	8	PA	0.002	0.040	0.978	0.990	1.000
	9	T2D	0.000	0.010	0.979	0.990	1.000
	10	LDL	-0.001	0.020	0.990	0.990	1.000

Table S16. Application example 1 on common exposures for cardiovascular disease outcomes using the Mendelian randomisation Bayesian model averaging (MR-BMA) algorithm which is run on each outcome separately. Results include the model-averaged causal effect estimate (MACE), the marginal posterior probability of inclusion (mPPI) and the corresponding empirical p-value [12]. We adjust for multiple testing using Benjamini-Hochberg False Discovery Rate (FDR) and Bonferroni correction. For each outcome, we sort the exposures by their empirical p-value in increasing order and the absolute value of MACE in decreasing order. Exposures selected at 5% FDR highlighted in bold.

Outcome	Ranking	Exposure	Effect estimate	Standard error	p-value	FDR	Bonferroni
CAD	1	M.VLDL.P	0.354	0.289	0.224	0.853	1.000
	2	S.LDL.P	-0.519	0.549	0.347	0.853	1.000
	3	M.LDL.P	1.550	1.727	0.371	0.853	1.000
	4	S.VLDL.P	-0.239	0.340	0.483	0.853	1.000
	5	L.LDL.P	-1.096	2.207	0.620	0.853	1.000
	6	IDL.P	0.541	1.329	0.685	0.853	1.000
	7	L.VLDL.P	-0.120	0.322	0.709	0.853	1.000
	8	XL.VLDL.P	-0.068	0.212	0.749	0.853	1.000
	9	XXL.VLDL.P	-0.048	0.184	0.795	0.853	1.000
	10	XS.VLDL.P	0.105	0.566	0.853	0.853	1.000
PAD	1	XS.VLDL.P	0.727	0.469	0.124	0.632	1.000
	2	S.LDL.P	-0.690	0.533	0.198	0.632	1.000
	3	S.VLDL.P	-0.384	0.305	0.210	0.632	1.000
	4	XL.VLDL.P	-0.240	0.209	0.253	0.632	1.000
	5	XXL.VLDL.P	0.172	0.184	0.352	0.703	1.000
	6	M.LDL.P	1.164	1.666	0.486	0.810	1.000
	7	IDL.P	-0.636	1.156	0.583	0.812	1.000
	8	M.VLDL.P	0.126	0.276	0.649	0.812	1.000
	9	L.VLDL.P	0.078	0.309	0.801	0.890	1.000
	10	L.LDL.P	-0.076	2.065	0.971	0.971	1.000
HF	1	M.LDL.P	3.620	1.478	0.016	0.156	0.156
	2	L.LDL.P	-3.783	1.856	0.044	0.158	0.435
	3	S.VLDL.P	-0.550	0.275	0.047	0.158	0.474
	4	S.LDL.P	-0.867	0.468	0.066	0.166	0.664
	5	M.VLDL.P	0.390	0.249	0.120	0.240	1.000
	6	XXL.VLDL.P	0.218	0.165	0.189	0.287	1.000
	7	IDL.P	1.344	1.046	0.201	0.287	1.000
	8	L.VLDL.P	-0.209	0.279	0.456	0.562	1.000
	9	XL.VLDL.P	-0.125	0.188	0.506	0.562	1.000
	10	XS.VLDL.P	0.238	0.423	0.574	0.574	1.000
AF	1	L.VLDL.P	-0.417	0.250	0.097	0.945	0.968
	2	XXL.VLDL.P	0.157	0.140	0.263	0.945	1.000
	3	M.VLDL.P	0.172	0.220	0.438	0.945	1.000
	4	XL.VLDL.P	-0.093	0.157	0.556	0.945	1.000
	5	XS.VLDL.P	0.209	0.374	0.578	0.945	1.000
	6	IDL.P	-0.448	0.902	0.620	0.945	1.000
	7	S.VLDL.P	0.086	0.247	0.730	0.945	1.000
	8	L.LDL.P	0.401	1.572	0.799	0.945	1.000
	9	M.LDL.P	-0.131	1.262	0.917	0.945	1.000
	10	S.LDL.P	0.029	0.414	0.945	0.945	1.000
CES	1	IDL.P	-3.923	1.654	0.019	0.142	0.191
	2	L.LDL.P	6.416	2.894	0.028	0.142	0.283
	3	M.LDL.P	-4.059	2.311	0.081	0.271	0.813
	4	XS.VLDL.P	0.928	0.673	0.170	0.426	1.000
	5	S.VLDL.P	0.541	0.440	0.220	0.440	1.000
	6	XXL.VLDL.P	0.223	0.249	0.372	0.541	1.000
	7	L.VLDL.P	-0.392	0.443	0.378	0.541	1.000
	8	M.VLDL.P	-0.264	0.391	0.500	0.625	1.000
	9	S.LDL.P	0.406	0.742	0.585	0.650	1.000
	10	XL.VLDL.P	0.046	0.286	0.874	0.874	1.000

Table S17. Application example 2 on molecular risk factors for cardiovascular disease outcomes using standard multivariable MR (MV-MR) which is run on each outcome separately. Results presented include the causal effect estimates, the standard error and the corresponding p-value. We adjusted for multiple testing using Benjamini-Hochberg False Discovery Rate (FDR) and Bonferroni correction. For each outcome, we sort the exposures by their p-value in increasing order. No exposures are selected at 5% FDR.

Outcome	Ranking	Exposure	Effect estimate	Standard error	p-value	FDR	Bonferroni
CAD		(Intercept)	-0.031	0.112	0.781	0.781	-
	1	M.VLDL.P	0.348	0.291	0.235	0.860	1.000
	2	S.LDL.P	-0.511	0.552	0.356	0.860	1.000
	3	M.LDL.P	1.558	1.733	0.371	0.860	1.000
	4	S.VLDL.P	-0.235	0.341	0.493	0.860	1.000
	5	L.LDL.P	-1.121	2.217	0.614	0.860	1.000
	6	IDL.P	0.555	1.335	0.678	0.860	1.000
	7	L.VLDL.P	-0.119	0.324	0.715	0.860	1.000
	8	XL.VLDL.P	-0.071	0.213	0.739	0.860	1.000
	9	XXL.VLDL.P	-0.047	0.185	0.799	0.860	1.000
	10	XS.VLDL.P	0.100	0.568	0.860	0.860	1.000
PAD		(Intercept)	0.085	0.107	0.426	0.426	-
	1	XS.VLDL.P	0.767	0.473	0.107	0.615	1.000
	2	S.LDL.P	-0.724	0.535	0.179	0.615	1.000
	3	S.VLDL.P	-0.409	0.307	0.185	0.615	1.000
	4	XL.VLDL.P	-0.232	0.210	0.270	0.675	1.000
	5	XXL.VLDL.P	0.174	0.185	0.349	0.698	1.000
	6	M.LDL.P	1.162	1.668	0.487	0.769	1.000
	7	IDL.P	-0.717	1.162	0.539	0.769	1.000
	8	M.VLDL.P	0.137	0.277	0.623	0.778	1.000
	9	L.VLDL.P	0.078	0.309	0.801	0.890	1.000
	10	L.LDL.P	0.015	2.071	0.994	0.994	1.000
HF		(Intercept)	-0.126	0.095	0.186	0.186	-
	1	M.LDL.P	3.673	1.474	0.014	0.139	0.139
	2	L.LDL.P	-3.961	1.855	0.035	0.173	0.346
	3	S.VLDL.P	-0.515	0.275	0.064	0.196	0.637
	4	S.LDL.P	-0.830	0.468	0.078	0.196	0.784
	5	M.VLDL.P	0.371	0.249	0.138	0.271	1.000
	6	IDL.P	1.471	1.047	0.163	0.271	1.000
	7	XXL.VLDL.P	0.215	0.165	0.195	0.279	1.000
	8	L.VLDL.P	-0.208	0.279	0.458	0.531	1.000
	9	XL.VLDL.P	-0.133	0.188	0.478	0.531	1.000
	10	XS.VLDL.P	0.179	0.424	0.674	0.674	1.000
AF		(Intercept)	0.070	0.085	0.417	0.417	-
	1	L.VLDL.P	-0.415	0.250	0.099	0.987	0.994
	2	XXL.VLDL.P	0.156	0.140	0.266	0.987	1.000
	3	M.VLDL.P	0.180	0.221	0.418	0.987	1.000
	4	XS.VLDL.P	0.232	0.376	0.538	0.987	1.000
	5	IDL.P	-0.491	0.905	0.588	0.987	1.000
	6	XL.VLDL.P	-0.085	0.157	0.592	0.987	1.000
	7	L.LDL.P	0.442	1.575	0.780	0.989	1.000
	8	S.VLDL.P	0.066	0.249	0.791	0.989	1.000
	9	M.LDL.P	-0.112	1.264	0.929	0.997	1.000
	10	S.LDL.P	-0.002	0.416	0.997	0.997	1.000
CES		(Intercept)	0.286	0.151	0.060	0.060	-
	1	IDL.P	-4.151	1.643	0.013	0.109	0.127
	2	L.LDL.P	6.657	2.870	0.022	0.109	0.219
	3	M.LDL.P	-4.028	2.289	0.081	0.269	0.807
	4	XS.VLDL.P	1.044	0.669	0.121	0.303	1.000
	5	S.VLDL.P	0.462	0.437	0.293	0.554	1.000
	6	XXL.VLDL.P	0.220	0.246	0.373	0.554	1.000
	7	L.VLDL.P	-0.381	0.439	0.388	0.554	1.000
	8	M.VLDL.P	-0.234	0.387	0.547	0.684	1.000
	9	S.LDL.P	0.292	0.737	0.692	0.769	1.000
	10	XL.VLDL.P	0.072	0.284	0.801	0.801	1.000

Table S18. Application example 2 on molecular risk factors for cardiovascular disease outcomes using multivariable MR-Egger which is run on each outcome separately. Results include the causal effect estimates as well as the intercept, the standard error and the corresponding p-value. We adjust for multiple testing using Benjamini-Hochberg False Discovery Rate (FDR) and Bonferroni correction. For each outcome, we sort the exposures by their p-value in increasing order. No molecular exposures are selected at 5% FDR.

Outcome	Ranking	Exposure	MACE	mPPI	Empirical p-value	FDR	Bonferroni
CAD	1	**L.LDL.P**	0.300	0.689	3.00×10^{-5}	3.00×10^{-4}	3.00×10^{-4}
	2	IDL.P	0.094	0.291	0.013	0.065	0.130
	3	M.LDL.P	0.063	0.212	0.055	0.184	0.553
	4	XS.VLDL.P	0.010	0.093	0.491	0.999	1.000
	5	S.LDL.P	-0.011	0.091	0.846	0.999	1.000
	6	M.VLDL.P	0.008	0.055	0.985	0.999	1.000
	7	L.VLDL.P	-0.003	0.040	0.997	0.999	1.000
	8	S.VLDL.P	0.004	0.049	0.997	0.999	1.000
	9	XL.VLDL.P	-0.004	0.041	0.997	0.999	1.000
	10	XXL.VLDL.P	-0.002	0.035	0.999	0.999	1.000
PAD	1	**XS.VLDL.P**	0.192	0.840	9.00×10^{-5}	0.001	0.001
	2	IDL.P	0.026	0.149	0.154	0.772	1.000
	3	XL.VLDL.P	-0.015	0.106	0.888	0.995	1.000
	4	L.LDL.P	0.004	0.063	0.938	0.995	1.000
	5	M.LDL.P	0.004	0.054	0.972	0.995	1.000
	6	S.VLDL.P	0.004	0.065	0.986	0.995	1.000
	7	L.VLDL.P	-0.003	0.056	0.988	0.995	1.000
	8	S.LDL.P	-0.002	0.044	0.992	0.995	1.000
	9	M.VLDL.P	0.003	0.049	0.994	0.995	1.000
	10	XXL.VLDL.P	-0.001	0.049	0.995	0.995	1.000
HF	1	**XS.VLDL.P**	0.190	0.881	1.00×10^{-5}	1.00×10^{-4}	1.00×10^{-4}
	2	IDL.P	0.018	0.118	0.336	0.997	1.000
	3	L.LDL.P	0.001	0.054	0.964	0.997	1.000
	4	M.LDL.P	0.004	0.051	0.977	0.997	1.000
	5	S.LDL.P	0.002	0.044	0.991	0.997	1.000
	6	M.VLDL.P	0.005	0.047	0.993	0.997	1.000
	7	XXL.VLDL.P	0.005	0.049	0.993	0.997	1.000
	8	XL.VLDL.P	-0.005	0.050	0.993	0.997	1.000
	9	L.VLDL.P	-0.003	0.041	0.997	0.997	1.000
	10	S.VLDL.P	0.001	0.050	0.997	0.997	1.000
AF	1	**S.VLDL.P**	0.071	0.433	0.002	0.016	0.023
	2	**L.VLDL.P**	-0.122	0.473	0.003	0.016	0.031
	3	XL.VLDL.P	-0.041	0.274	0.044	0.135	0.442
	4	M.VLDL.P	0.045	0.220	0.054	0.135	0.539
	5	XS.VLDL.P	0.009	0.132	0.147	0.293	1.000
	6	IDL.P	0.005	0.108	0.368	0.614	1.000
	7	L.LDL.P	0.007	0.108	0.476	0.681	1.000
	8	M.LDL.P	0.008	0.103	0.646	0.807	1.000
	9	S.LDL.P	-0.005	0.093	0.785	0.872	1.000
	10	XXL.VLDL.P	0.001	0.089	0.918	0.918	1.000
CES	1	XXL.VLDL.P	-0.010	0.170	0.273	0.639	1.000
	2	L.VLDL.P	-0.011	0.149	0.336	0.639	1.000
	3	S.VLDL.P	0.006	0.109	0.369	0.639	1.000
	4	XS.VLDL.P	-0.003	0.095	0.419	0.639	1.000
	5	IDL.P	-0.005	0.100	0.461	0.639	1.000
	6	M.VLDL.P	0.001	0.124	0.478	0.639	1.000
	7	XL.VLDL.P	-0.003	0.139	0.509	0.639	1.000
	8	M.LDL.P	0.006	0.104	0.615	0.639	1.000
	9	L.LDL.P	0.000	0.099	0.618	0.639	1.000
	10	S.LDL.P	-0.004	0.102	0.639	0.639	1.000

Table S19. Application example 2 on molecular risk factors for cardiovascular disease outcomes using the Mendelian randomisation Bayesian model averaging (MR-BMA) algorithm wich is run on each outcome separately. Results include the model-averaged causal effect estimate (MACE), the marginal posterior probability of inclusion (mPPI) and the corresponding empirical p-value [12]. We adjust for multiple testing using Benjamini-Hochberg False Discovery Rate (FDR) and Bonferroni correction. For each outcome, we sort the exposures by their empirical p-value in increasing order and the absolute value of MACE in decreasing order. Exposures selected at 5% FDR highlighted in bold.

S4.4 Supplemental figures

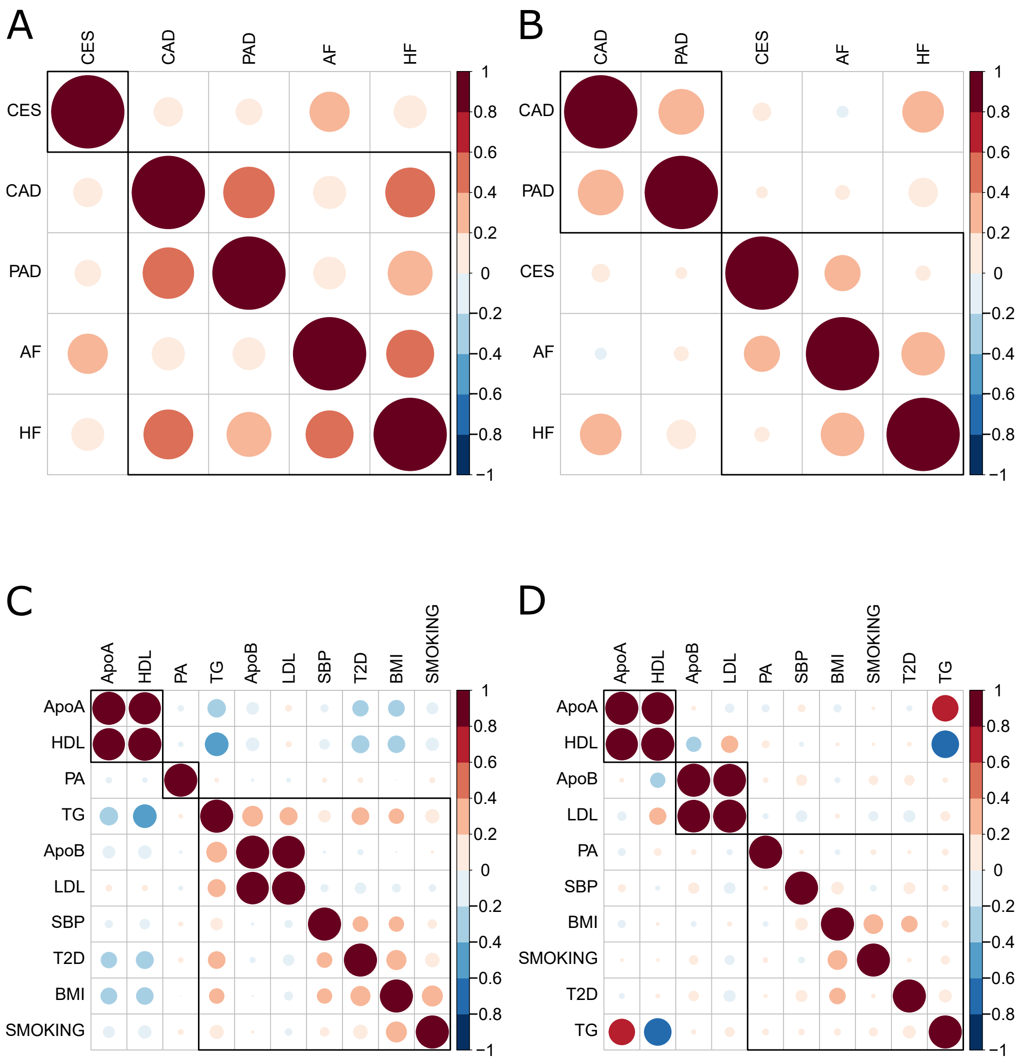

Figure S8. Correlation and partial correlation between summary-level outcomes and between summary-level exposures in application example 1 on common exposures for cardiovascular disease outcomes. Black boxes indicate clusters obtained by using hierarchical clustering with average agglomeration method, where the optimal number of clusters is estimated using `NbClust` R package. (**A**) Correlation between responses based on 1,533 independent genetic variants associated with any of the ten exposures after removing seven IVs identified as outliers or high-leverage and influential observations (see Figure S10A). (**B**) Partial correlation between responses based on the same IVs. (**C**) Correlation between summary-level exposures based on 1,533 independent genetic variants associated with any of the ten exposures. (**D**) Partial correlation between summary-level exposures based on the same IVs.

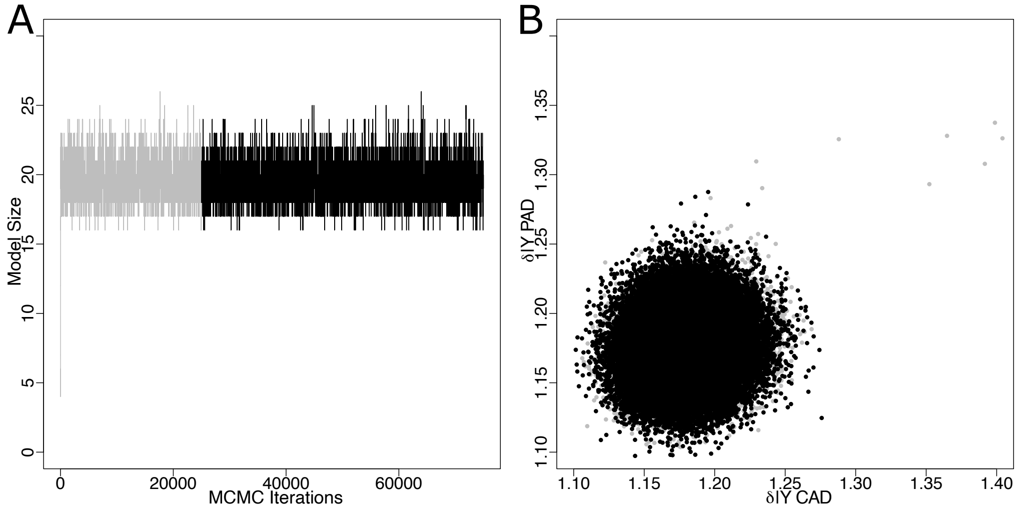

Figure S9. Convergence diagnostics of the multi-response MR^2 model in application example 1 on common exposures for cardiovascular disease outcomes (CVDs)(A) Trace plot of the number of non-zeros direct casual effects estimated during 75K MCMC iterations. Grey colour indicates burn-in phase up to 25K iterations, while black colour shows samples drawn after the burn-in. The designed MCMC algorithm reaches convergence after few iterations. (**B**) Scatterplot of the posterior samples of the square root of the error variance $\delta_k|Y$ for CAD and PAD. Grey colour indicates burn-in phase up to 25K iterations, while black colour shows samples drawn after the burn-in. After a few iterations (top right corner) the algorithm samples from the equilibrium distribution of the Markov chain.

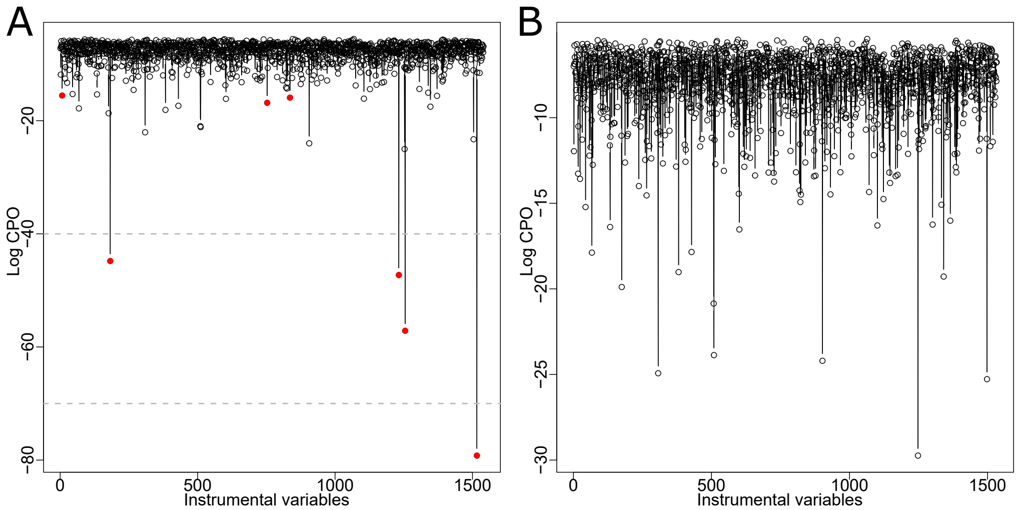

Figure S10. Outliers, high-leverage and influential observations detection using the multi-response MR^2 model in application example 1 on common exposures for cardiovascular disease outcomes (CVDs), plotting the log-conditional predictive ordinate (CPO) (y-axis) against 1.540 instrumental variables (x-axis). Red dots indicate observations with scaled CPOs below 0.01 to be regarded as outliers, high-leverage and influential observations [15]. We also show log-inverse-CPOs larger than 40 (possible outliers) and higher than 70 (extreme values) [26]. (**A**) Log-CPO for each instrumental variable and outliers, high-leverage and influential observations detected in the original summary-level data set of common exposures for CVDs. Seven IVs (0.5%) have scaled CPOs below 0.01, so the MR^2 model is considered to fit adequately. (**B**) Log-CPOs after removing outliers, high-leverage and influential observations from the original data set. No outliers or high-leverage and influential observations are detected.

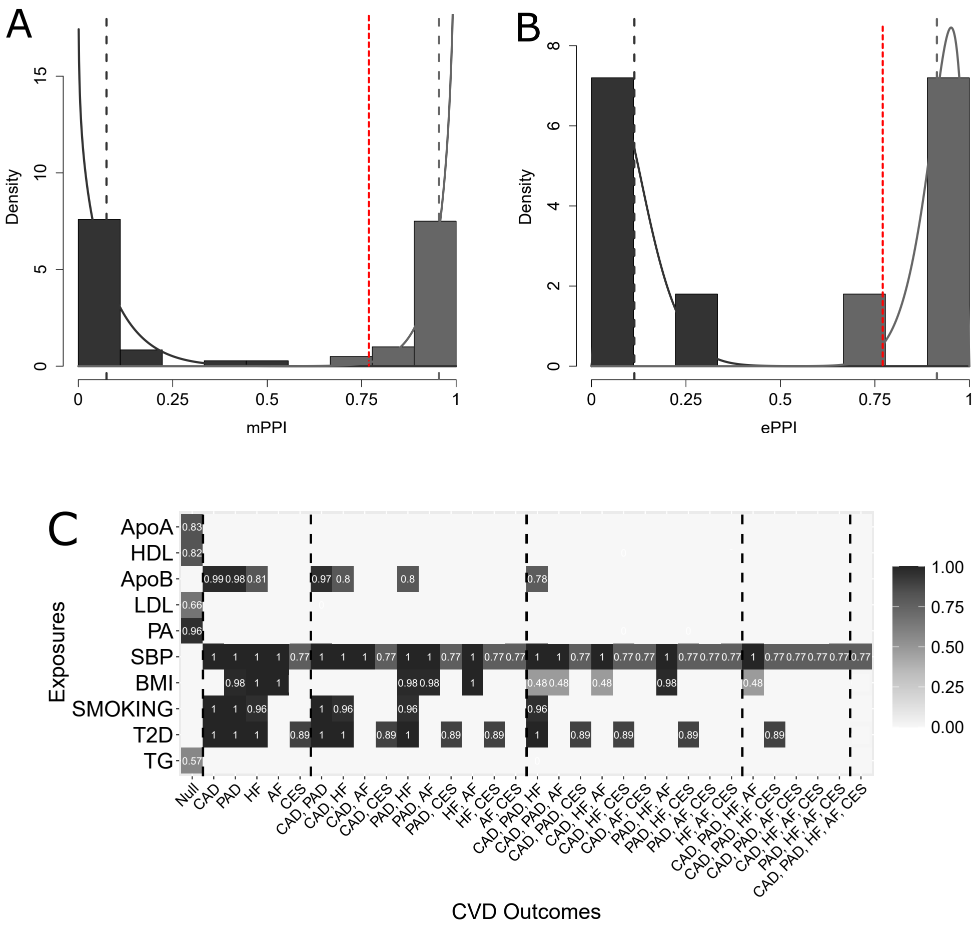

Figure S11. Post-processing results of the multi-response MR^2 model in application example 1 on common exposures for cardiovascular disease outcomes. (**A**) Non-parametric False Discovery Rate (FDR) strategy based on two-component mixture model which clusters low and high levels of the marginal posterior probabilities of inclusion (mPPIs). Dark grey colour code indicates mPPIs and the estimated density of the null (H_0) component, while light grey is used for the alternative (H_1) component. Dark grey and light grey dotted vertical lines shows the means of the beta components with a good separation between them, while red dotted vertical line specifies the threshold on the mPPI scale (mPPI ≥ 0.77) that corresponds to 5% FDR nominal level. (**B**) Non-parametric FDR strategy based on two-component mixture model which clusters low and high levels of the edge posterior probabilities of inclusion (ePPIs). Red dotted vertical line specifies the threshold on the ePPI scale (ePPI ≥ 0.78) that corresponds to 5% FDR nominal level. (**C**) Marginal and joint posterior probability of inclusion (jPPI) of each exposure (y-axis) against a single response or combinations of them (x-axis). Black dotted vertical lines separate combinations of outcomes with the same group size, *i.e.*, singletons, all possible pairs, all possible triplets, etc. For clarity of presentation, mPPIs for non-selected outcome-exposure pairs (mPPI < 0.77 at 5% FDR) are not plotted as well as jPPIs for combination of exposures marginally not selected. The "Null" vertical bar on the left indicates the posterior model probability of the null model.

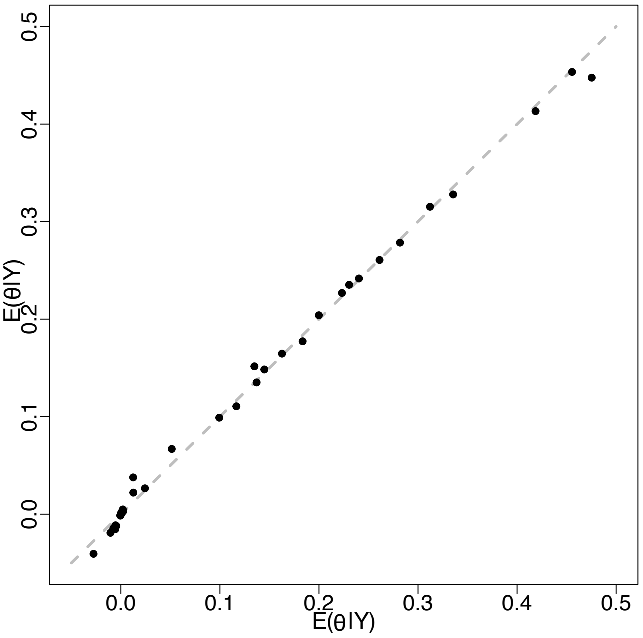

Figure S12. Sensitivity analysis of MR^2 with respected the *a priori* range of important exposures in application example 1 on common exposures for cardiovascular disease outcomes (CVDs). Robustness of the proposed method with respect to the level of sparsity controlled by $\mathbb{E}(\gamma_k)$ and $\mathbb{V}(\gamma_k)$, the *a priori* average number of important exposures associated with each response and its variance. In the original analysis, we fixed these values at $\mathbb{E}(\gamma_k) = 2$ and $\mathbb{V}(\gamma_k) = 2$. This prior choice implies *a priori* a range between zero and seven for the number of significant direct causal effects for each outcome. We changed them to $\mathbb{E}(\gamma_k) = 4$ and $\mathbb{V}(\gamma_k) = 4$ with *a priori* range between zero and 10. Scatterplot of the posterior mean of the direct causal effects in the original prior specification (x-axis) and the new specification (y-axis) shows no appreciable differences.

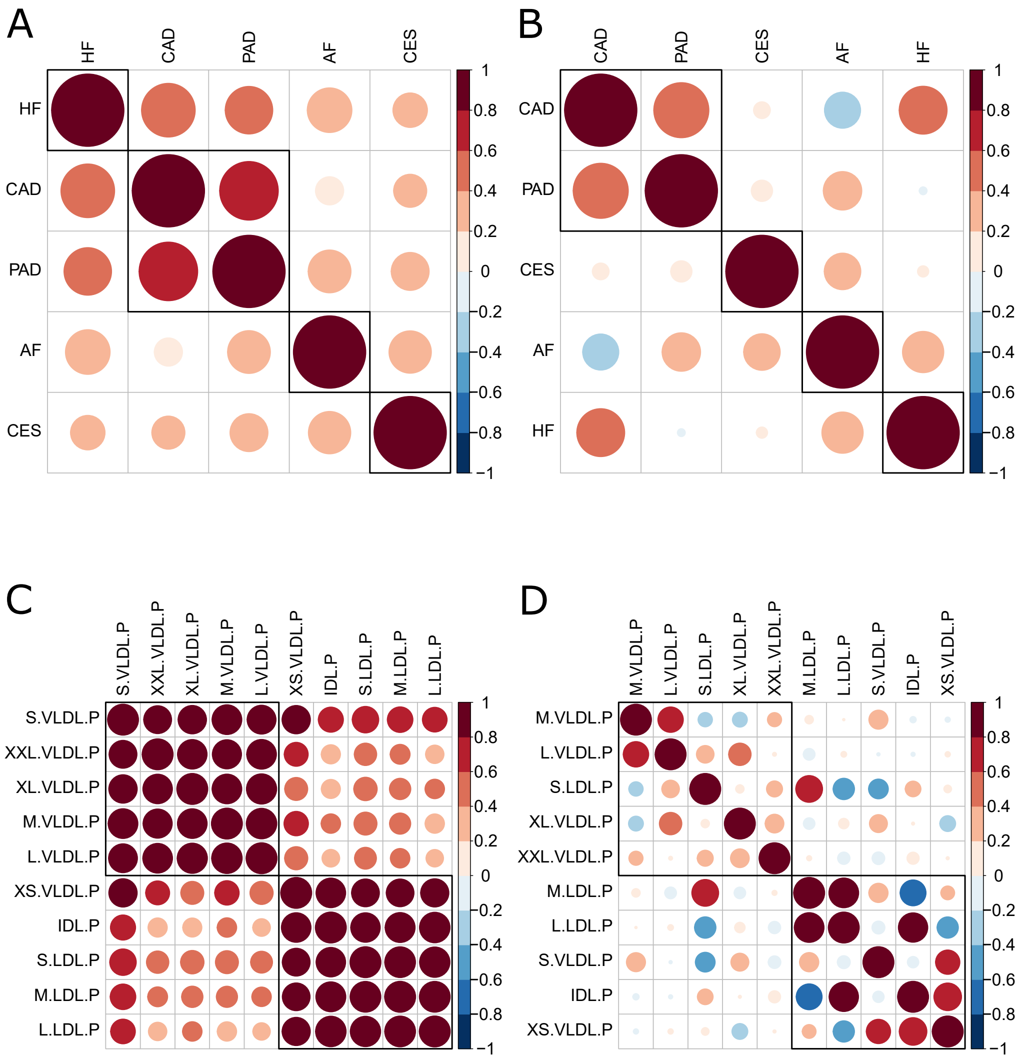

Figure S13. Correlation and partial correlation between summary-level outcomes and between summary-level exposures in application example 2 on molecular exposures for cardiovascular disease outcomes. Black boxes indicate clusters obtained by using hierarchical clustering with average agglomeration method where the optimal number of clusters was estimated using `NbClust` R package. (**A**) Correlation between responses based on 140 independent genetic variants associated with any of the ten molecular exposures after removing eight IVS identified as outliers or high-leverage and influential observations (see Figure S15A). (**B**) Partial correlation between summary-level responses based on the same IVs. (**C**) Correlation between risk factors based on 140 independent genetic variants associated with any of the ten molecular exposures. (**D**) Partial correlation between summary-level risk factors based on the same IVs.

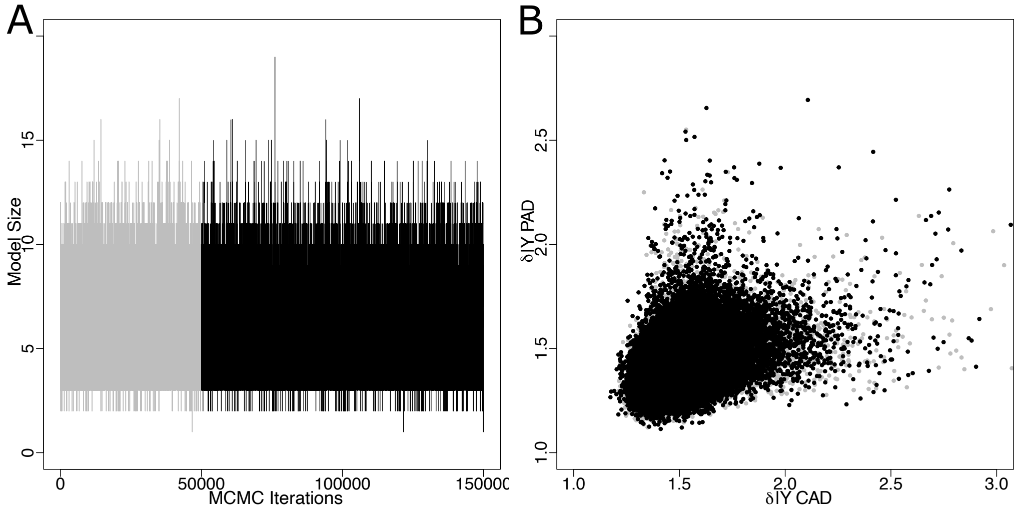

Figure S14. Convergence diagnostics of the multi-response MR^2 model in application example 2 on molecular risk factors for cardiovascular disease outcomes (CVDs)(**A**) Trace plot of the number of non-zeros direct casual effects estimated during 150K MCMC iterations. Grey colour indicates burn-in phase up to 50K iterations, while black colour shows samples drawn after the burn-in. The designed MCMC algorithm reaches convergence after few iterations. (**B**) Scatterplot of the posterior samples of the square root of the error variance $\delta_k|Y$ for CAD and PAD. Grey colour indicates burn-in phase up to 50K iterations, while black colour shows samples drawn after the burn-in. After a few iterations, the algorithm samples from the equilibrium distribution of the Markov chain. Since fewer exposures are selected, MR^2 samples larger error variances than in application example 1.

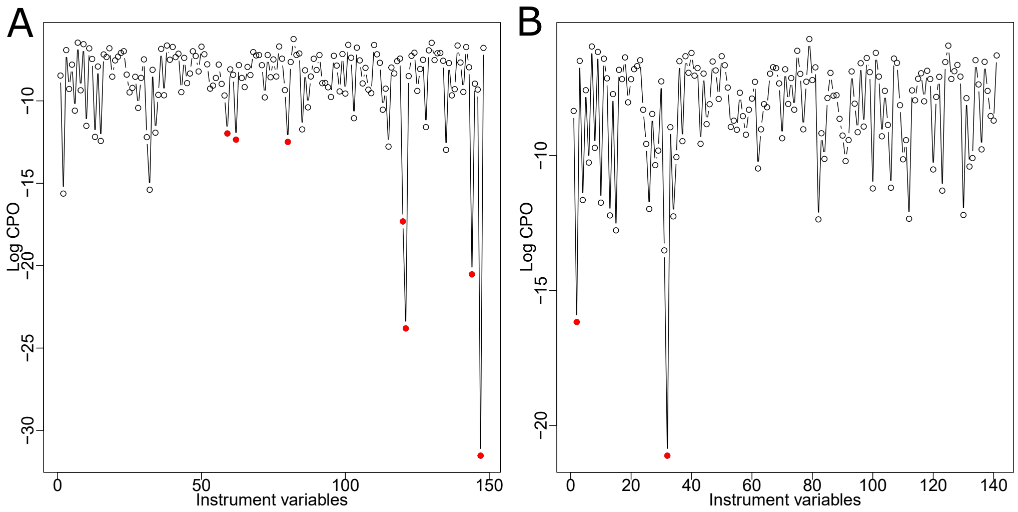

Figure S15. Outliers, high-leverage and influential observations detection using the multi-response MR^2 model in application example 2 on molecular risk factors for cardiovascular disease outcomes (CVDs), plotting the log-conditional predictive ordinate (CPO) (y-axis) against 148 instrumental variables (x-axis). Red dots indicate observations with scaled CPOs below 0.01 to be regarded as outliers, high-leverage and influential observations [15]. (**A**) Log-CPO for each instrumental variable and outliers, high-leverage and influential observations detected in the original summary-level data set of molecular risk factors for CVDs. Seven IVs (5%) have scaled CPOs below 0.01. (**B**) Log-CPOs after removing outliers, high-leverage and influential observations from the original data set. The MR^2 model is now considered to fit adequately since only two outliers or high-leverage and influential observations (2%) are still detected.

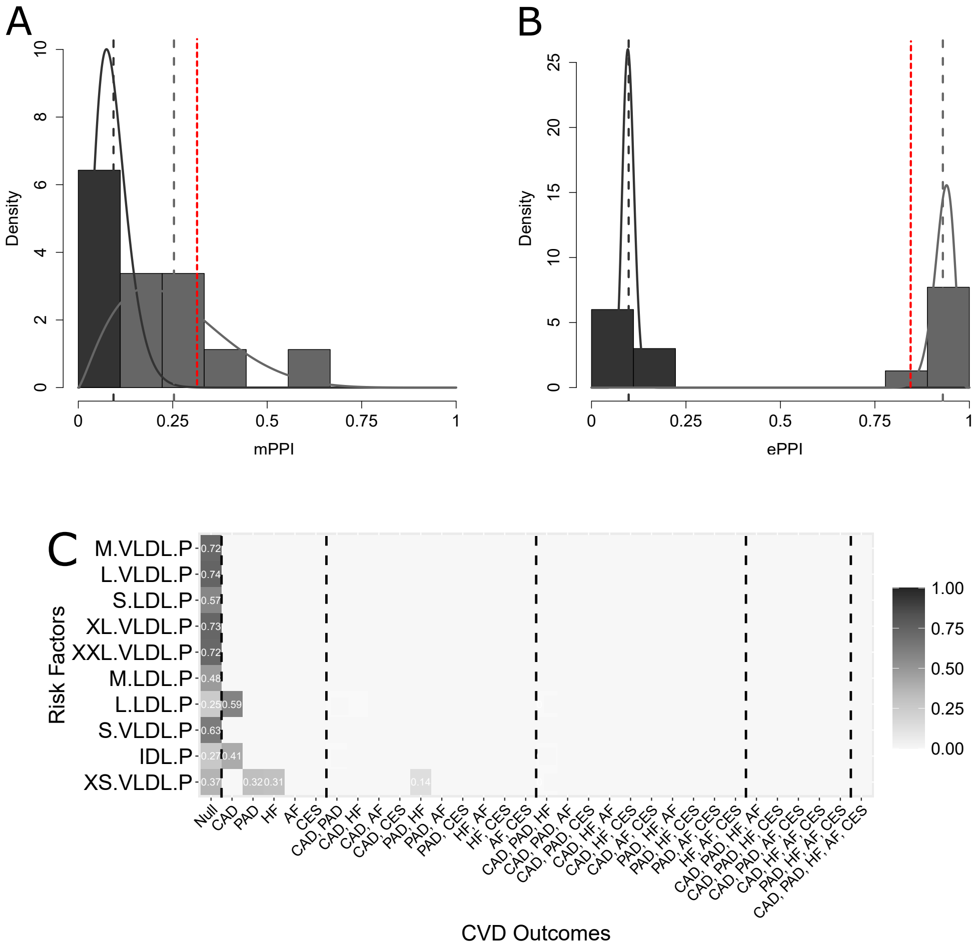

Figure S16. Post-processing results of the multi-response MR^2 model in application example 2 on molecular risk factors for cardiovascular disease outcomes. (**A**) Non-parametric False Discovery Rate (FDR) strategy based on two-component mixture model which clusters low and high levels of the marginal posterior probabilities of inclusion (mPPIs). Dark grey colour code indicates mPPIs and the estimated density of the null (H_0) component, while light grey is used for the alternative (H_1) component. Dark grey and light grey dotted vertical lines shows the means of the beta components, while red dotted vertical line specifies the threshold on the mPPI scale (mPPI ≥ 0.29) that corresponds to 5% FDR nominal level. (**B**) Non-parametric FDR strategy based on two-component mixture model which clusters low and high levels of the edge posterior probabilities of inclusion (ePPIs). Red dotted vertical line specifies the threshold on the ePPI scale (ePPI ≥ 0.85) that corresponds to 5% FDR nominal level.(**C**) Marginal and joint posterior probability of inclusion (jPPI) of each exposure (y-axis) against a single response or combinations of them (x-axis). Black dotted vertical lines separate combinations of outcomes with the same group size, *i.e.*, singletons, all possible pairs, all possible triplets, etc. For clarity of presentation, mPPIs for non-selected outcome-exposure pairs (mPPI < 0.29 at 5% FDR) are not plotted as well as jPPIs for combination of risk factors marginally not selected. The "Null" vertical bar on the left indicates the posterior model probability of the null model.

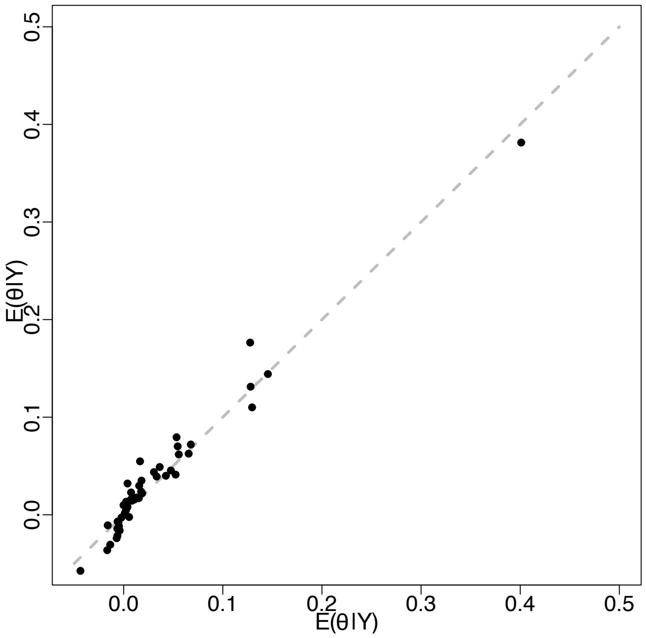

Figure S17. Sensitivity analysis of MR^2 with respected the *a priori* range of important exposures in application example 2 on molecular risk factors for cardiovascular disease outcomes. Robustness of the proposed method with respect to the level of sparsity controlled by $\mathbb{E}(\gamma_k)$ and $\mathbb{V}(\gamma_k)$, the *a priori* average number of important exposures associated with each response and its variance. In the original analysis, we fixed these values at $\mathbb{E}(\gamma_k) = 2$ and $\mathbb{V}(\gamma_k) = 2$. This prior choice implies *a priori* a range between zero and seven for the number of significant direct causal effects for each outcome. We changed them to $\mathbb{E}(\gamma_k) = 4$ and $\mathbb{V}(\gamma_k) = 4$ with *a priori* range between zero and 10. Scatterplot of the posterior mean of the direct causal effects in the original prior specification (x-axis) and the new specification (y-axis) shows no appreciable differences.

References

[1] Alexopoulos, A. & Bottolo, L. Bayesian Variable Selection for Gaussian copula regression models. *Journal of Computational and Graphical Statistics* **30**, 578–593 (2021). URL `https://doi.org/10.1080/10618600.2020.1840997`.

[2] Holmes, C. C. & Held, L. Bayesian auxiliary variable models for binary and multinomial regression. *Bayesian Analysis* **1**, 145–168 (2006). URL `https://doi.org/10.1214/06-BA105`.

[3] Rothman, A. J., Levina, E. & Zhu, J. Sparse multivariate regression with covariance estimation. *Journal of Computational and Graphical Statistics* **19**, 947–962 (2010). URL `https://doi.org/10.1198/jcgs.2010.09188`.

[4] Deshpande, S. K., Ročková, V. & George, E. I. Simultaneous variable and covariance selection with the multivariate spike-and-slab lasso. *Journal of Computational and Graphical Statistics* **28**, 921–931 (2019). URL `https://doi.org/10.1080/10618600.2019.1593179`.

[5] Wang, H. Sparse seemingly unrelated regression modelling: Applications in finance and econometrics. *Computational Statistics and Data Analysis* **54**, 2866–2877 (2010). URL `https://doi.org/10.1016/j.csda.2010.03.028`.

[6] George, E. & McCulloch, R. E. Variable selection via Gibbs sampling. *Journal of the American Statistical Association* **88**, 881–889 (1993). URL `https://www.jstor.org/stable/2290777`.

[7] Lauritzen, S. L. *Graphical Models* (Clarendon Press, Oxford, New York, 2004). Repr. with corrections.

[8] Hans, C., Dobra, A. & West, M. Shotgun stochastic search for "large p" regression. *Journal of the American Statistical Association* **102**, 507–516 (2007). URL `https://doi.org/10.1198/016214507000000121`.

[9] Burgess, S., Daniel, R. M., Butterworth, A. S. & Thompson, S. G. Network Mendelian randomization: Using genetic variants as instrumental variables to investigate mediation in causal pathways. *International Journal of Epidemiology* **44**, 484–495 (2015). URL `https://doi.org/10.1093/ije/dyu176`.

[10] Zuber, V., Colijn, J. M., Klaver, C. & Burgess, S. Selecting likely causal risk factors from high-throughput experiments using multivariable Mendelian randomization. *Nature Communications* **11**, 29 (2020). URL `https://doi.org/10.1038/s41467-019-13870-3`.

[11] Rees, J. M. B., Wood, A. M. & Burgess, S. Extending the MR-Egger method for multivariable Mendelian randomization to correct for both measured and unmeasured pleiotropy. *Statistics in Medicine* **36**, 4705–4718 (2017). URL `https://doi.org/10.1002/sim.7492`.

[12] Zuber, V. *et al.* High-throughput multivariable Mendelian randomization analysis prioritizes apolipoprotein B as key lipid risk factor for coronary artery disease. *International Journal of Epidemiology* **50**, 893–901 (2021). URL `https://doi.org/10.1093/ije/dyaa216`.

[13] Kettunen, J. *et al.* Genome-wide study for circulating metabolites identifies 62 loci and reveals novel systemic effects of LPA. *Nature Communications* **7** (2016). URL `https://doi.org/10.1038/ncomms11122`.

[14] Levin, M. G. *et al.* Prioritizing the role of major lipoproteins and subfractions as risk factors for peripheral artery disease. *Circulation* **144**, 353–364 (2021). URL `https://doi.org/10.1161/circulationaha.121.053797`.

[15] Congdon, P. *Bayesian Models for Categorical Data* (John Wiley & Sons, Chichester, 2005).

[16] Benjamini, Y. & Hochberg, Y. Controlling the false discovery rate: a practical and powerful approach to multiple testing. *Journal of the Royal Statistical Society: Series B (Methodological)* **57**, 289–300 (1995). URL `https://doi.org/10.1111/j.2517-6161.1995.tb02031.x`.

[17] Nielsen, J. B. *et al.* Biobank-driven genomic discovery yields new insight into atrial fibrillation biology. *Nature Genetics* **50**, 1234–1239 (2018). URL `https://doi.org/10.1038/s41588-018-0171-3`.

[18] Mishra, A. *et al.* Stroke genetics informs drug discovery and risk prediction across ancestries. *Nature* **611**, 115–123 (2022). URL `https://doi.org/10.1038/s41586-022-05165-3`.

[19] Nelson, C. P. *et al.* Association analyses based on false discovery rate implicate new loci for coronary artery disease. *Nature Genetics* **49**, 1385–1391 (2017). URL `https://doi.org/10.1038/ng.3913`.

[20] Klarin, D. *et al.* Genome-wide association study of peripheral artery disease in the Million Veteran Program. *Nature Medicine* **25**, 1274–1279 (2019). URL https://doi.org/10.1038/s41591-019-0492-5.

[21] Shah, S. *et al.* Genome-wide association and Mendelian randomisation analysis provide insights into the pathogenesis of heart failure. *Nature Communications* **11**, 163 (2020). URL https://doi.org/10.1038/s41467-019-13690-5.

[22] Liam, A. *et al.* UkBiobank GWAS results (2018). URL http://www.nealelab.is/uk-biobank.

[23] Wang, Z. *et al.* Genome-wide association analyses of physical activity and sedentary behavior provide insights into underlying mechanisms and roles in disease prevention. *Nature Genetics* **54**, 1332–1344 (2022). URL https://doi.org/10.1038/s41588-022-01165-1.

[24] Wootton, R. E. *et al.* Evidence for causal effects of lifetime smoking on risk for depression and schizophrenia: A Mendelian randomisation study. *Psychological Medicine* **50**, 2435–2443 (2020). URL https://doi.org/10.1017/S0033291719002678.

[25] Mahajan, A. *et al.* Fine-mapping type 2 diabetes loci to single-variant resolution using high-density imputation and islet-specific epigenome maps. *Nature Genetics* **50**, 1505–1513 (2018). URL https://doi.org/10.1038/s41588-018-0241-6.

[26] Ntzoufras, I. *Bayesian Modeling Using WinBUGS* (Wiley, Hoboken, N.J., 2009). URL https://doi.org/10.1002/9780470434567.
