# Supplementary material for: Multi-response Mendelian randomization: Identification of shared and distinct exposures for multimorbidity and multiple related disease outcomes
Source: Am J Hum Genet. 2023 Jul 6;110(7):1177–99. doi: 10.1016/j.ajhg.2023.06.005 (PMC10357504; doi:10.1016/j.ajhg.2023.06.005)
Supplement: Document S2. Article plus supplemental information [file mmc2.pdf]

Multi-response Mendelian randomization: Identification of shared and distinct exposures for multimorbidity and multiple related disease outcomes

Authors

Verena Zuber, Alex Lewin, Michael G. Levin, ...,
Stephen Burgess, Dipender Gill,
Leonardo Bottolo

Correspondence

v.zuber@imperial.ac.uk (V.Z.),
lb664@cam.ac.uk (L.B.)

Existing Mendelian randomization (MR) models consider one outcome at a time in isolation and cannot identify causes of multimorbidity. We present multi-response MR (MR^2), an MR method specifically designed for multiple outcomes to identify exposures that cause multiple responses or, conversely, exposures that exert their effect on distinct responses.

Zuber et al., 2023, The American Journal of Human Genetics *110*, 1177–1199
July 6, 2023 Crown Copyright © 2023
https://doi.org/10.1016/j.ajhg.2023.06.005

Multi-response Mendelian randomization: Identification of shared and distinct exposures for multimorbidity and multiple related disease outcomes

Verena Zuber,[1,2,3,*] Alex Lewin,[4] Michael G. Levin,[5,6,7] Alexander Haglund,[8] Soumaya Ben-Aicha,[9] Costanza Emanueli,[9] Scott Damrauer,[10,11,7] Stephen Burgess,[12,13] Dipender Gill,[1,14] and Leonardo Bottolo[15,16,12,*]

Summary

The existing framework of Mendelian randomization (MR) infers the causal effect of one or multiple exposures on one single outcome. It is not designed to jointly model multiple outcomes, as would be necessary to detect causes of more than one outcome and would be relevant to model multimorbidity or other related disease outcomes. Here, we introduce multi-response Mendelian randomization (MR^2), an MR method specifically designed for multiple outcomes to identify exposures that cause more than one outcome or, conversely, exposures that exert their effect on distinct responses. MR^2 uses a sparse Bayesian Gaussian copula regression framework to detect causal effects while estimating the residual correlation between summary-level outcomes, i.e., the correlation that cannot be explained by the exposures, and vice versa. We show both theoretically and in a comprehensive simulation study how unmeasured shared pleiotropy induces residual correlation between outcomes irrespective of sample overlap. We also reveal how non-genetic factors that affect more than one outcome contribute to their correlation. We demonstrate that by accounting for residual correlation, MR^2 has higher power to detect shared exposures causing more than one outcome. It also provides more accurate causal effect estimates than existing methods that ignore the dependence between related responses. Finally, we illustrate how MR^2 detects shared and distinct causal exposures for five cardiovascular diseases in two applications considering cardiometabolic and lipidomic exposures and uncovers residual correlation between summary-level outcomes reflecting known relationships between cardiovascular diseases.

Introduction

Researchers focus often on understanding, preventing, and treating specific health conditions in isolation with a disease-centric approach. Yet, as life expectancy increases, the incidence of diseases increases, and a growing proportion of the adult population is affected by more than one chronic health condition.[1–3] Multimorbidity describes the simultaneous presence of two or more chronic conditions in one individual.[4] The Academy of Medical Science considers multimorbidity as a key priority for global health research,[5] and the World Health Organization identifies people with multimorbidities at higher risk of patient safety issues.[6] To define effective prevention and intervention strategies, it is important to understand disease etiology. Recent research into multimorbidity suggests the presence of disease clusters systematically co-occurring in subjects with specific genetic predispositions and exposed to certain exposures.[7,8] Yet, to date, it is unclear whether multimorbidity represents a random co-occurrence of seemingly unrelated individual health conditions without a common cause or whether shared causal exposures are underpinning multiple health conditions.[3,9] Our motivation is to develop principled causal inference methodology to detect shared or distinct causes of multiple related health outcomes using genetic evidence in a multi-trait Mendelian randomization (MR) framework.

Observational studies may be biased by unmeasured confounding factors and cannot be used to infer causality. MR uses genetic variants as instrumental variables (IVs)[10] to infer the direct causal effect of an exposure on an outcome irrespective of unmeasured confounders.[11–13] MR has become an important analytical approach to gaining a deeper understanding of how modifiable exposures impact a single disease outcome.

Yet, while there are methods for multivariable MR (MV-MR) that can deal with multiple exposures in one joint model,[14,15] to date there is no comprehensive MR methodology that can jointly model multiple outcomes, account for information shared between the outcomes,

[1]Department of Epidemiology and Biostatistics, School of Public Health, Imperial College London, London, UK; [2]MRC Centre for Environment and Health, School of Public Health, Imperial College London, London, UK; [3]UK Dementia Research Institute, Imperial College London, London, UK; [4]Department of Medical Statistics, London School of Hygiene and Tropical Medicine, London, UK; [5]Division of Cardiovascular Medicine, Perelman School of Medicine, University of Pennsylvania, Philadelphia, PA, USA; [6]Department of Medicine, Perelman School of Medicine, University of Pennsylvania, Philadelphia, PA, USA; [7]Corporal Michael J. Crescenz VA Medical Center, Philadelphia, USA; [8]Department of Brain Sciences, Faculty of Medicine, Imperial College London, London, UK; [9]National Heart and Lung Institute, Imperial College London, London, UK; [10]Department of Surgery, Perelman School of Medicine, University of Pennsylvania, Philadelphia, PA, USA; [11]Department of Genetics, Perelman School of Medicine, University of Pennsylvania, Philadelphia, PA, USA; [12]MRC Biostatistics Unit, School of Clinical Medicine, University of Cambridge, Cambridge, UK; [13]Cardiovascular Epidemiology Unit, School of Clinical Medicine, University of Cambridge, Cambridge, UK; [14]Chief Scientific Advisor Office, Research and Early Development, Novo Nordisk, Copenhagen, Denmark; [15]Department of Medical Genetics, School of Clinical Medicine, University of Cambridge, Cambridge, UK; [16]Alan Turing Institute, London, UK
*Correspondence: v.zuber@imperial.ac.uk (V.Z.), lb664@cam.ac.uk (L.B.)
https://doi.org/10.1016/j.ajhg.2023.06.005.
Crown Copyright © 2023 This is an open access article under the CC BY license (http://creativecommons.org/licenses/by/4.0/).

and simultaneously detect common and distinct causes of disease. Consequently, existing MR methodology largely neglects links between related disease outcomes. For example, we have recently performed wide-angled MR investigations to look at genetic determinants of lipids and cardiovascular disease outcomes (CVDs)[16] and blood lipids and particle sizes as exposures for coronary and peripheral artery disease (PAD)[17] where we have performed MR analysis for each outcome separately. While this strategy provides a first scan if similar causal exposures are significantly detected across different traits, there is no principled MR methodology available to test and define whether an exposure affects more than one outcome. Moreover, existing models ignore information shared between outcomes because each trait is considered in isolation. Thus, methods are needed to make full use of the growing knowledge regarding clusters of diseases that may share the same causes. In addition, genome-wide association studies (GWASs) are generally sparsely controlled for confounders, and only a few covariates like age, sex, and principal components to control for population stratification are included in the regression model to derive summary-level data for genetic associations. While this strategy reduces the risk of collider bias, there may be potential residual confounding in the GWASs themselves, which can be addressed by jointly modeling multiple outcomes.

Here, we propose multi-response MR (MR^2) to model multiple related health conditions in a joint multivariate (multiple outcomes) and multivariable (multiple exposures) MR model. Our motivations are the following: first, we seek to distinguish between exposures that are shared (affecting more than one outcome at the same time) or distinct (affecting only one outcome). Second, our multi-response model aims at increasing the power to detect exposures that affect more than one outcome while effectively reducing the number of false positives. Third, MR^2 aims to combine information between outcomes to identify the effect of unmeasured pleiotropic pathways on the responses as well as the impact of non-genetic factors (independent of the exposures), such as social health determinants, on the correlation between disease outcomes.

As the first motivating example, we want to identify which common cardiometabolic risk factors, including diabetes, dyslipidemia, hypertension, physical inactivity, obesity, and smoking, are shared or distinct causes of five cardiovascular diseases, including atrial fibrillation (AF), cardioembolic stroke (CES), coronary artery disease (CAD), heart failure (HF), and PAD. We include these outcomes because there is *a priori* epidemiological and clinical evidence that they are strongly connected due to shared risk factors and because one outcome causes another. For example, CAD can cause HF[18,19] and AF.[20] In turn, AF can cause CES.[21] Consequently, in clinal practice, these cardiovascular diseases are frequently present as multimorbidity.[22] Patients living with one disease are more likely to be affected by a second cardiovascular illness than a healthy individual becoming sick with one cardiovascular disease.[23] Epidemiological evidence[24–27] also suggests that these diseases share a wide range of common exposures. Determining whether these exposures are universally causal or influenced by residual confouding/correlation is challenging to infer from traditional observational study designs. To date, no study has used genetic evidence in a joint multi-outcome model to establish which exposures are shared or distinct. Here, we illustrate the advantage of using the proposed joint multivariable and multi-response MR^2 model to identify which cardiovascular exposures are shared or distinct for different cardiovascular conditions.

As a second motivating example, we follow up on the findings from the first example to define in more detail which lipid characteristics and lipoprotein-related traits, as measured by high-throughput metabolomics, are likely causes of the selected cardiovascular diseases.

The manuscript is outlined as follows: after material and methods, where we introduce the Bayesian modeling framework of MR^2, we present in results an extensive simulation study. First, we illustrate how residual correlation is caused by unmeasured shared pleiotropy, and second, we compare MR^2 with existing multivariable, single-outcome MR models and with other statistical learning algorithms for multi-response regression regarding their ability to detect important causal exposures, distinguish between shared and distinct exposure, and accurately estimate causal effects. Then, we present the results from the two motivating application examples. In the real examples, we contrast the results obtained by MR^2 with standard MV-MR[14] and with MR with Bayesian model averaging (MR-BMA), a recently proposed method for single-trait MV-MR[15] to highlight the gain of power and the reduction of false positives when multiple responses are jointly analyzed. We also compare MR^2 with MV-MR-Egger[28] to demonstrate different effects of the unmeasured pleiotropy when dealing with multiple outcomes. Finally, we conclude with a discussion and directions for future research.

Material and methods

In this section, we illustrate the data input utilized in the proposed method as well as in existing MR models including univariable (one exposure and one outcome) and MV-MR (multiple exposures and one outcome). Then, we describe how multiple outcomes can be modeled jointly by considering the seemingly unrelated regression (SUR) framework and how this can be generalized by the copula regression model. We show analytically and demonstrate in the simulation study (see results) how unmeasured shared pleiotropy affecting more than one outcome can be captured in a multi-response MR model, which accounts for the residual correlation between outcomes both in overlapping and non-overlapping samples in the genetic associations with the outcomes. Finally, we conclude material and methods with an overview of MR^2, which implements a sparse copula regression model and focuses on the selection of shared and distinct exposures for multiple health conditions. Technical details are presented in appendix A. The Markov chain Monte Carlo (MCMC) implementation of the proposed MR^2 method is described in supplemental information.

MR data input

MR^2 is formulated on summary-level data of genetic association with the exposures and outcomes from large-scale GWASs, which are commonly available in the public domain.

According to the two-sample summary-level MR framework, we assume that the genetic associations with the exposure and the genetic associations with the outcome are taken from two distinct cohorts with non-overlapping samples[29] and are thus *a priori* independent.[30] However, when considering multiple exposures, some of them may be derived from cohorts with full or partially overlapping samples. In this case, because MV-MR models do account for measured pleiotropy between exposures,[14,31] overlapping samples can be analyzed. Besides modeling multiple exposures, MR^2 also explicitly considers the correlation between multiple responses from cohorts with fully, partially, or no overlapping samples.

In summary, the summary-level design facilitates the inclusion of different disease outcomes, as well as exposures. Moreover, they do not necessarily need to be measured on different independent cohorts or be fully or partially overlapping.

Overview of existing MR models

Standard MR models for summary-level data, both univariable (single exposure) and multivariable (multiple exposures), are formulated as weighted linear regression models where the genetic associations with exposure are regressed against the genetic associations with the outcome. Each genetic variant, used as IVs, contributes one data point (or observation) to the regression model, which we denote with the index i, $i = 1, \ldots, n$. For each IV, we take the beta coefficient β_{X_i} and standard error $\mathrm{se}(\beta_{X_i})$ from a univariable regression in which the exposure X is regressed on the genetic variant G_i in sample one and beta coefficient β_{Y_i} and standard error $\mathrm{se}(\beta_{Y_i})$ from a univariable regression in which the outcome Y is regressed on the genetic variant G_i in sample two.

Then, univariable MR can be formulated as a weighted linear regression model in which the genetic associations with the outcome β_{Y_i} are regressed on the genetic associations with the exposure β_{X_i}[32]

$$\beta_{Y_i} = \beta_{X_i}\theta + \epsilon_i,\ \epsilon_i \sim \mathrm{N}\left(\mu, \delta^2 \mathrm{se}\left(\beta_{Y_i}\right)^2\right),\ i = 1, \ldots, n, \quad \text{(Equation 1)}$$

where θ is the effect estimate, μ is the intercept, and δ^2 is an overdispersion parameter, $\delta^2 \geq 1$, that incorporates residual heterogeneity into the model.[33,34] Standard MR models set $\mu = 0$, while $\mu \neq 0$ captures unmeasured horizontal pleiotropy.[35] Weighting each genetic variant i by the first-order weights $\mathrm{se}(\beta_{Y_i})^2$ is equivalent to fitting an inverse variance weighting (IVW) MR model, which gives genetic variants measured with higher precision larger weights.[13] Alternatively, the genetic associations may be standardized before the analysis by the weights $\omega_i = \mathrm{se}(\beta_{Y_i}), i = 1, \ldots, n$ that only depend on the standard errors of the genetic associations with the outcome.

MV-MR[14,31] is an extension of univariable MR to consider not just one single exposure but multiple exposures in one joint model. This joint model accounts for measured pleiotropy[14] by modeling explicitly pleiotropic pathways via any of the included exposures. Additionally, MV-MR can be used to select the most likely causal exposures from a set of candidate exposures.[15,36]

In analogy with the univariable MR model in Equation 1, in MV-MR the genetic associations with one outcome are regressed on the genetic associations with all the exposures[37]

$$\beta_{Y_i} = \beta_{X_{i1}}\theta_1 + \beta_{X_{i2}}\theta_2 + \cdots + \beta_{X_{ip}}\theta_p + \epsilon_i,\ \epsilon_i \sim \mathrm{N}\left(\mu, \delta^2 \mathrm{se}\left(\beta_{Y_i}\right)^2\right),$$

or, in vector notation,

$$\beta_{Y_i} = \beta_{X_i}\theta + \epsilon_i,\ \epsilon_i \sim \mathrm{N}\left(\mu, \delta^2 \mathrm{se}\left(\beta_{Y_i}\right)^2\right), \quad \text{(Equation 2)}$$

for each $i = 1, \ldots, n$, where β_{Y_i} are the associations of the genetic variant G_i with the outcome Y, β_{X_i} contains the associations of the genetic variant G_i with the p exposures, $\theta = (\theta_1, \ldots, \theta_p)^T$ is the vector of the effect estimates, μ is the intercept that models unmeasured horizontal pleiotropy, and $\delta^2 > 1$ is the overdispersion parameter.

In MV-MR, a genetic variant is a valid IV if the following criteria hold: "IV1—relevance," the variant is associated with at least one of the exposures; "IV2—exchangeability," the variant is independent of all confounders of each of the exposure-outcome associations; and "IV3—exclusion restriction," the variant is independent of the outcome conditional on the exposures and confounders.

Given these assumptions hold, we consider the effect estimates θ as the direct causal effect[37] of the exposure on the outcome after keeping all other exposures constant.

Multi-response MR

We present here the extension of the MV-MR model in Equation 2 for p exposures when q outcomes are jointly considered. Consequently, the observed summary-level input data are a matrix β_Y of dimension $n \times q$, which contains the genetic associations with n genetic variants with the q outcomes and β_X of dimension $n \times p$, which includes the genetic associations with the same n genetic variants with the p exposures.

In the following, we assume that the input data β_Y and β_X have been standardized according to IVW before the analysis, and, for ease of exposition, we use the same notation after standardization. The IVW is based on first-order weights that are proportional to the inverse of the standard error of the outcome for single-response MR models. The IVW MR model is taking into account the precision of a genetic variant and gives IVs with higher precision stronger weights in the MR model. Details on how to define IVW in a multiple-outcome framework are provided in appendix A.

The aim to model q-related outcomes in one joint model can be achieved by using the SUR framework,[38] which is formulated as a series of q multivariable regression equations, one for each of the q outcomes.

$$\begin{aligned} \beta_{Y_{i1}} &= \beta_{X_i}\theta_1 + \epsilon_{i1} \\ \vdots &= \vdots \\ \beta_{Y_{iq}} &= \beta_{X_i}\theta_q + \epsilon_{iq} \end{aligned} \quad \text{(Equation 3)}$$

For each $i = 1, \ldots, n$, where $\beta_{Y_i} = (\beta_{Y_{i1}}, \ldots, \beta_{Y_{iq}})$ contains the observed genetic associations with the genetic variant G_i with the q responses, β_{X_i} are the associations of the genetic variant G_i with the p exposures, $\theta_1 = (\theta_{11}, \ldots, \theta_{1p})^T$, ..., $\theta_q = (\theta_{q1} \ldots, \theta_{qp})^T$ are the outcome-specific vectors of direct causal effects for each outcome, and $\epsilon_{i1} \sim \mathrm{N}(\mu_1, \delta_1^2), \ldots, \epsilon_{iq} \sim \mathrm{N}(\mu_q, \delta_q^2)$ are the residuals with $\mu_1, \ldots, \mu_q$ the response-specific intercepts and $\delta_1^2, \ldots, \delta_q^2$ the overdispersion parameters.

The SUR model connects the q multivariable regressions in Equation 3 by allowing for correlation between the q residuals of the summary-level outcomes ϵ_k, $k = 1, \ldots, q$. More precisely, the SUR model estimates the $(q \times q)$-dimensional covariance matrix between the vector of residuals $\epsilon_i = (\epsilon_{i1}, \ldots, \epsilon_{iq})$.

$$R = \begin{pmatrix} 1 & \rho(\epsilon_{i1}, \epsilon_{i2}) & \cdots & \rho(\epsilon_{i1}, \epsilon_q) \\ \rho(\epsilon_{i2}, \epsilon_{i1}) & 1 & \cdots & \rho(\epsilon_{i2}, \epsilon_{iq}) \\ \vdots & \vdots & \ddots & \vdots \\ \rho(\epsilon_{iq}, \epsilon_{i1}) & \rho(\epsilon_{iq}, \epsilon_{i2}) & \cdots & 1 \end{pmatrix}. \quad \text{(Equation 4)}$$

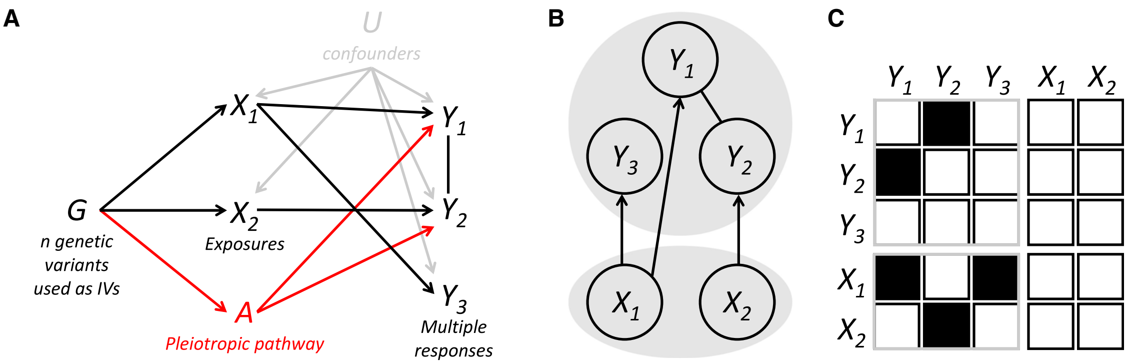

Figure 1. Multivariable and multi-response Mendelian randomization with multiple exposures and multiple responses
(A) Directed acyclic graph (DAG) with G, genetic variant(s); $X = \{X_1, X_2\}$, exposures; $Y = \{Y_1, Y_2, Y_3\}$, responses; U, unmeasured confounders; A, unobserved pleiotropic pathway.
(B) Schematic representation of the MR^2 model, which allows the exploration of the model space consisting of all possible subsets of exposures (bottom gray circle) directly associated with the responses (top gray circle) while estimating the residual correlation between the responses (top gray circle) and vice versa. Model depicted in (A) is reported in MR^2 as follows: X_1 is a shared exposure for outcomes Y_1 and Y_3, while X_2 has a distinct direct causal effect on Y_2 (directed edge). Residual dependence between Y_1 and Y_2 is still present after conditioning on the associated exposures (undirected edge), and it depends on the unmeasured pleiotropic pathway A.
(C) MR^2 model space exploration is equivalent to learning from the summary-level input data, a partitioned non-symmetrical adjacency matrix. The model depicted in (A) is represented by an adjacency matrix describing the conditional dependence structure among the responses (top left symmetrical submatrix) and the direct causal association of the exposures with the outcomes (bottom left non-symmetrical submatrix). No reverse causation is allowed in the MR^2 model (top right non-symmetrical submatrix), and the exposures can only have direct causal effects on the responses, i.e., no direct effects among the exposures are modeled (bottom right symmetrical submatrix).

For instance, $\rho(\varepsilon_{ik}, \varepsilon_{ik'})$ is the correlation between the residuals of the MV-MR model for summary-level outcome k and the residuals of the MV-MR model for summary-level outcome k', $k \neq k' = 1, \ldots, q$.

Finally, the possibility to account for response-specific unmeasured horizontal pleiotropy can be turned off by setting the vector of intercepts $\mu = (\mu_1, \ldots, \mu_q)^T$ to zero.

Correlation between outcomes in multi-response MR

To understand what contributes to the residuals ε_k, $k = 1, \ldots, q$, of the summary-level MR model in Equation 3 and generates correlation between them in Equation 4, we focus on the following generating model for the kth outcome on individual-level data considering N subjects, as illustrated in Figure 1A,

$$Y_k = X\theta_k + A\theta^A + U\theta_Y^U + \varepsilon_k, k = 1, \ldots, q, \quad \text{(Equation 5)}$$

where θ_k is the p-dimensional vector of effects of the exposures X on the kth outcome, θ^A is the effect of the unmeasured pleiotropic pathway A on the responses, θ_Y^U is the effect of the unmeasured confounder U on the outcomes, and $\varepsilon_k \sim \mathrm{N}_N(0, \delta_k^2 I_N)$ with δ_k^2 the response-specific residual variance and N the sample size. Moreover, X, A, and U are random quantities that are descendants of the same set of IVs.

Assuming that the quantitative outcomes Y_k, $k = 1, .., q$, are measured on the same N individuals, the n-dimensional vector of summary-level genetic associations with the kth response β_{Y_k} can be derived using the ordinary least squares (OLS) estimate

$$\begin{aligned} \beta_{Y_k} &= \left(G^T G\right)^{-1} G^T Y_k \\ &= \left(G^T G\right)^{-1} G^T \left(X\theta_k + A\theta^A + U\theta_Y^U + \varepsilon_k\right) \\ &= \left(G^T G\right)^{-1} G^T X\theta_k + \left(G^T G\right)^{-1} G^T \left(A\theta^A + U\theta_Y^U + \varepsilon_k\right), \end{aligned}$$

where the $(N \times n)$-dimensional matrix G describes the n genetic variants associated with the exposures and measured on N individuals. Moreover, assuming that the genetic variants G selected as IVs are independent of each other (achieved by pruning) and independent of the confounder U (exchangeability assumption), the above equation simplifies to

$$\begin{aligned} \beta_{Y_k} &= \left(G^T G\right)^{-1} G^T X\theta_k + \left(G^T G\right)^{-1} G^T A\theta^A + \left(G^T G\right)^{-1} G^T \varepsilon_k \\ &= \beta_X \theta_k + \beta_A \theta^A + \tilde{\varepsilon}_k, \end{aligned} \quad \text{(Equation 6)}$$

where $\beta_X = (G^T G)^{-1} G^T X$ is the $(n \times p)$-dimensional matrix of the summary-level genetic association with the measured exposures X, $\beta_A = (G^T G)^{-1} G^T A$ is the n-dimensional vector of genetic associations with the unmeasured pleiotropic pathway A, and $\tilde{\varepsilon}_k$ can be viewed as the OLS of the projection of ε_k onto the linear space spanned by G. Note that $\mathbb{V}(\tilde{\varepsilon}_k) = \delta_k^2 (G^T G)^{-1}$, and, given the assumption of independence of the genetic variants, it simplifies to $\mathbb{V}(\tilde{\varepsilon}_k) = \delta_k^2 \mathrm{diag}(v_1^2, \ldots, v_n^2)$, where diag$(\cdot)$ indicates a diagonal matrix. Thus, $\tilde{\varepsilon}_k$ is an n-dimensional vector and $\tilde{\varepsilon}_k \sim \mathrm{N}_n(0, \delta_k^2 \mathrm{diag}(v_1^2, \ldots, v_n^2))$.

Consequently, the residuals in Equation 3 for the kth summary-level response, $k = 1, \ldots, q$, using Equation 6, can be decomposed into

$$\begin{aligned} \varepsilon_k &= \beta_{Y_k} - \beta_X \theta_k \\ &= \beta_X \theta_k + \beta_A \theta^A + \tilde{\varepsilon}_k - \beta_X \theta_k \\ &= \beta_A \theta^A + \tilde{\varepsilon}_k, \end{aligned} \quad \text{(Equation 7)}$$

which shows that residuals in the summary-level MR model reflect unmeasured shared pleiotropy and residual variation.

Because in the designed framework the unmeasured pleiotropic pathway A is random, β_A in Equation 7 is also random, and thus, $\beta_A \theta^A$ cannot be treated as the fixed-effect response-specific intercept μ_k.[28] Instead, it should be interpreted as a random intercept common to all summary-level responses. Similarly to random-effect models, the distribution of ε_k follows an n-dimensional Gaussian distribution with $\mathbb{E}(\varepsilon_k) = \theta^A \mu_A \mathrm{diag}(v_1^2, \ldots, v_n^2) G^T 1_N$ with 1_N an N-dimensional vector of ones and $\mathbb{V}(\varepsilon_k) = \{(\theta^A)^2 \sigma_A^2 + \delta_k^2\} \mathrm{diag}(v_1^2, \ldots, v_n^2)$, assuming A and ε_k independent,

and $\mathbb{E}(A) = \mu_A 1_N$ and $\mathbb{V}(A) = \sigma_A^2 I_N$ with I_N the identity matrix of dimension N. Moreover, it reveals that the residual correlations in Equation 4 depend on the unmeasured shared pleiotropy, as illustrated below for two outcomes k and k', $k \neq k'$,

$$\rho(\epsilon_{ik}, \epsilon_{ik'}) = \frac{(\theta^A)^2 \sigma_A^2 + \sigma_{kk'}}{\left\{(\theta^A)^2 \sigma_A^2 + \delta_k^2\right\}^{1/2} \left\{(\theta^A)^2 \sigma_A^2 + \delta_{k'}^2\right\}^{1/2}}, \quad \text{(Equation 8)}$$

where $\sigma_{kk'} = \text{Cov}(\varepsilon_k, \varepsilon_{k'})$ is the covariance between individual-level responses' errors in Equation 5, which is assumed constant across all N individuals.

Equation 8 shows that, independently of the sign of θ^A (the effect of the unmeasured pleiotropic pathway A on the responses) and the nature of the shared pleiotropy A (either "directed," i.e., $A_l > 0$, or "undirected," i.e., $A_l \lessgtr 0$, $l = 1, \dots, N$), the effect of unmeasured shared pleiotropy on the correlation between the residuals of the MV-MR model is always positive and constant across all combinations of responses. Moreover, the residual correlation is different from zero even when $\text{Cov}(\varepsilon_k, \varepsilon_{k'}) = 0$, i.e., there is no correlation between individual-level responses' errors.

Alternative scenarios can be also considered. As illustrated in Figure 1A, some outcomes may not be influenced by the unmeasured pleiotropic pathway, so their summary-level residual correlation with other responses is nil. Likewise, there may exist several unmeasured pleiotropic pathways that are shared by subgroups of responses (not necessarily distinct). For instance, pathway A_1 affects responses (k_1, k_1'), $k_1 \neq k_1'$, and A_2 impacts responses (k_2, k_2'), $k_2 \neq k_2'$. In these cases, the derivation of Equation 8 does not change, although $\rho(\epsilon_{ik}, \epsilon_{ik'})$ won't be constant across all outcomes' pairs. Full details are presented in supplemental information.

Another important quantity in Equation 8 is $\sigma_{kk'}$, which is the covariance between individual-level responses' errors. In contrast with the correlation induced by unmeasured pleiotropic pathway A, which is genetically proxied by the selected IVs, $\sigma_{kk'}$, $k \neq k' = 1, \dots, q$, does not depend on G. Examples of non-genetic factors that contribute to the outcomes' correlations are, for instance, social health determinants[39] such as personal features, socioeconomic status, culture, environment, health behaviors, access to care, and government policy. Notably, these interconnected factors that determine an individual's health status are not associated with the exposures and, therefore, are not confounders. This echoes the instrument strength independent of direct effect (InSIDE) assumption,[35] where the pleiotropic effect is independent of the genetic associations with the exposure, albeit here, it induces correlation between responses. Moreover, while further exposures can be included in the analysis with the hope to catch the effects of unmeasured shared pathways, the residual correlations generated by non-genetic factors cannot be "explained away" in the current MR framework. Thus, it should be considered as the baseline residual correlation between summary-level outcomes.

Finally, Equation 8 has been derived assuming that the quantitative outcomes Y_k, $k = 1, .., q$, are measured on the same N individuals. Equation 8 still holds if non-overlapping samples of size N_k in the genetic associations with the responses are considered with the following modification: (1) $\sigma_{kk'}$ is equal to zero and (2) $\sigma_A^2 I_n$ is replaced by $G_k^T \text{Cov}(A_k, A_{k'}) G_{k'}$, where G_k is the $(n \times N_k)$-dimensional matrix of genotypes for the group of individuals k and similarly for $G_{k'}$. $\text{Cov}(A_k, A_{k'})$ is different from zero if we assume that, since we are dealing with the same pathway A, the genetic associations with A, which are specific for responses k and k', i.e., β_{A_k} and $\beta_{A_{k'}}$, are correlated. Full details are presented in supplemental information.

Bayesian multi-response MR

MR^2 is based on a recently proposed Bayesian method to select important predictors in regression models with multiple responses of any type.[40] Specifically, a sparse Gaussian copula regression (GCR) model[41] is used to account for the multivariate dependencies between the Gaussian responses β_Y once their direct causal association with a set of important exposures β_X is estimated. When only Gaussian responses are considered, the GCR is similar to the SUR model[38] (see appendix A). Figure 1B provides a schematic representation of the MR^2 model that allows for the estimation of important exposures (bottom gray circle) directly associated with the responses (top gray circle) while estimating the residual correlation between the responses (top gray circle) and vice versa.

Regarding MR^2 model specification, we use the hyper-inverse Wishart distribution as the prior density for the residual covariance matrix based on the theory of Gaussian graphical models.[42] This prior allows some of the off-diagonal elements of the inverse covariance matrix to be identical to zero and to estimate the residual correlation between the summary-level responses. For the exposures, we use a hierarchical non-conjugate model[43] to assign a prior distribution independently on each direct causal effect. A point mass at zero is specified on the regression coefficient of the null exposure, whereas a Gaussian distribution is assigned to the non-zero effect.

From a computational point of view, we design an efficient proposal distribution to update jointly the latent binary vectors for the selection of important exposures (selection step) and the corresponding non-zero effects (estimation step). For Gaussian responses, the designed proposal distribution allows the "implicit marginalization" of the non-zero effects in the Metropolis-Hastings (M-H) acceptance probability,[44] which makes our MCMC algorithm more efficient than existing sparse Bayesian SUR models[45–47] favoring the exploration of important combinations of exposures, i.e., the model space, in a very efficient manner (see supplemental information). It also guarantees a good acceptance rate, which, in turn, prevents slow convergence of the MCMC algorithm and reduces the autocorrelation of the posterior samples. Figure 1C shows that MR^2 model exploration is equivalent to learning from the input data a non-symmetrical adjacency matrix partitioned into a symmetrical submatrix (top left), which describes the conditional dependence structure among the responses and a non-symmetrical submatrix (bottom left) representing the direct causal association of the exposures with the outcomes. Note that neither reverse causation (top right submatrix) nor direct causal effects between the responses (bottom right submatrix) are allowed in the MR^2 model.

Two sets of parameters are deemed important in our analysis: the marginal posterior probability of inclusion (mPPI), which measures the strength of the direct causal association between each exposure-response combination and the corresponding direct causal effect, and the edge posterior probability of inclusion (ePPI), which describes the strength of the residual dependence between each pair of summary-level responses and the corresponding residual partial correlation. The posterior distribution of these quantities is usually summarized by their mean or any relevant quantile. For instance, the $\alpha/2$% and $(1 - \alpha/2)$% quantiles are used to build the $(1 - \alpha)$% credible interval (CI). We summarize

all quantities of interest by their posterior mean (both mPPI and ePPI can be seen as a posterior mean or frequency that an exposure-response combination or a pair of dependent responses are selected during the MCMC algorithm) and their 95% CI.

The selection of important exposures for each response and significantly correlated pairs of responses is based on mPPIs and ePPIs, respectively. Thresholding these quantities at 0.5 is usually suggested given that the optimal predictive model in linear regression is often the median probability model, which is defined as the model consisting of those predictors that have overall mPPI $\geq$ 0.5, the optimal predictive model under square loss, i.e., the optimal model that predicts not-yet-collected data.[48] Here, we follow a different approach based on in-sample selection. A non-parametric false discovery rate (FDR) strategy based on two-component mixture models,[49,50] which clusters low and high levels of mPPIs and low and high levels of ePPIs, is applied to select important exposures for each response and significant dependence patterns among responses at a fixed FDR level.

Importantly, for the definition of exposures causing more than one outcome, the availability of the latent binary vectors for the selection of important exposures for each response recorded during the MCMC algorithm also allows the estimation of the joint posterior probability of inclusion (jPPI), defined as the number of times an exposure is selected to be associated with two or more responses at the same time during the MCMC. Thus, jPPI can be regarded as the jPPI for any combination of responses. The detection of important direct causal effects of an exposure on a single response or multiple responses is carried out by looking at significant mPPIs selected at a nominal FDR level. If an exposure is associated with more than one response, we declare the existence of a shared direct causal effect and calculate the jPPIs.

While the residual correlation between summary-level responses captures "global" unmeasured shared pleiotropy, which is calculated across all genetic variants, we additionally screen for individual genetic variants as potential outliers due to their "local" pleiotropic effect.[51] Building on Bayesian tools for outlier diagnostics, we propose the conditional predictive ordinate (CPO)[52] to detect individual genetic variants as outliers or high-leverage and influential observations in the MR^2 model.

Full details of the MR^2 model, as well as post-processing of the MCMC output, are presented in appendix A.

Results

Simulation study: Can the effect of shared pleiotropy be detected?

Here, we conduct a simulation study to illustrate the impact of unmeasured shared pleiotropy affecting more than one outcome. We consider one of the scenarios presented in the simulation study (scenario III—undirected pleiotropy) where the residual correlation between outcomes at the summary level depends only on the unmeasured shared pleiotropy and $\sigma_{kk'} = 0$ for all responses. We look at two quantities: first, the empirical correlations between the summary-level genetic associations with the outcomes measured on the IVs. They can be computed by simply calculating the pairwise correlation between the genetic associations with the outcomes on the summary level. Second, we look at the residual correlation between the summary-level genetic associations with the outcomes measured on the IVs after accounting for the exposures and estimated by MR^2.

Figure 2 shows that the larger the pleiotropic effect θ^A (ranging from 0.25 to 2), the bigger the empirical correlation. However, the empirical correlation (in gray) is not able to distinguish between the correlation due to the direct causal effects of the exposures on the outcomes and the unmeasured shared pleiotropy. MR^2 can separate the source of correlation with a good agreement between the estimated values of the residual correlation (in red) and its theoretical value derived in Equation 8 (black dashed line).

Simulation study: Comparison of methodologies

We perform a simulation study to demonstrate the increase in power, a better estimation of direct causal effects when accounting for multiple correlated outcomes, and finally, the ability to detect shared and distinct causal exposures when using the proposed MR^2 method compared with existing MV-MR models that consider one outcome at a time and the recently proposed multi-response multivariable methods. In total, we simulate $n = 100$ genetic variants used as IVs, $p = 15$ exposures, and $q = 5$ outcomes. Out of the $p \times q$ exposure-outcome combinations, 30% have a non-zero direct causal effect. For all scenarios, we generate $N = 100{,}000$ individuals, of which half is used to generate the summary-level genetic associations for the exposure and half for the outcomes, respectively, providing data in a summary-level design. As alternative methods, we include standard MV-MR[37] and MR-BMA,[15] a Bayesian variable-selection approach for MV-MR. We consider two multivariable and multivariate variable-selection approaches, which have to date not been applied to MR: the multiple responses Lasso (multivariate regression with covariance estimation [MRCE])[46] and the multiple responses Spike-and-Slab Lasso (mSSL).[47] Both methods perform variable and covariance selection by inducing sparsity and setting the effect estimates of variables not included in the model to zero, as well as inducing sparsity in the residual covariance matrix. An overview of the alternative methods is provided in Table S1.

We simulate the following scenarios.

- Scenario I—null: there are no causal exposures for any of the outcomes and no confounder.
- Scenario II—confounding: there are 30% of exposures with a non-zero direct causal effect and there is a joint confounder for all outcomes.
- Scenario III—undirected pleiotropy: residual correlation between outcomes is induced by a shared undirected pleiotropic pathway that can increase or decrease the level of the responses.
- Scenario IV—directed pleiotropy: residual correlation between outcomes is induced by a shared directed pleiotropic pathway that only increases the level of the responses.

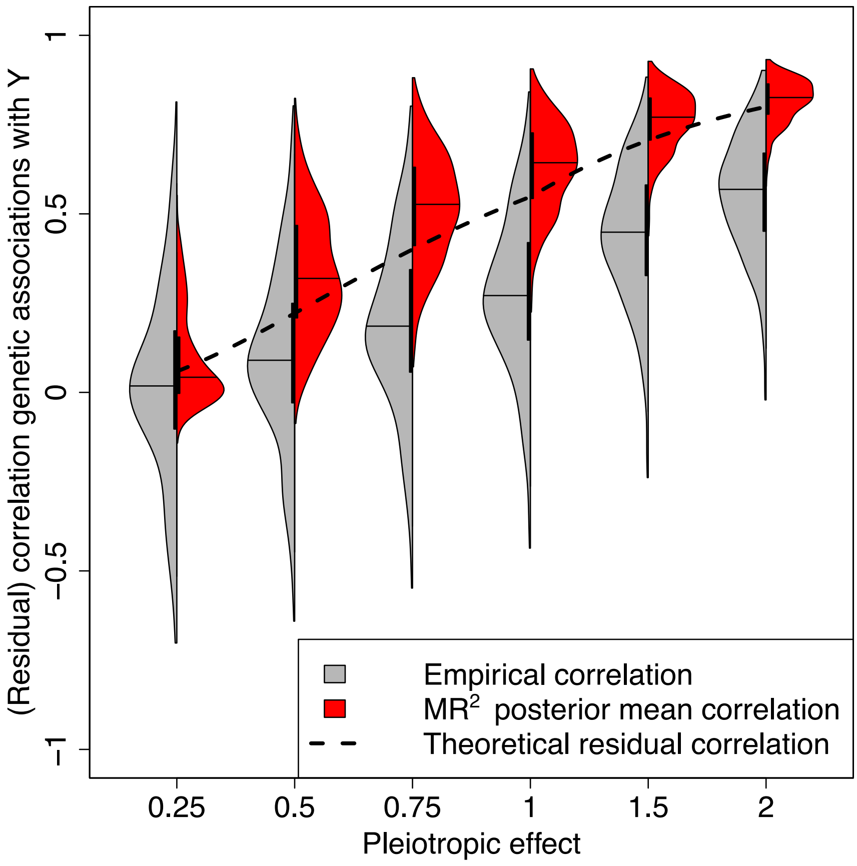

Figure 2. (Residual) correlation between the summary-level genetic associations with the outcomes induced by various levels of the pleiotropic effect and with sample overlap between the summary-level data of the outcomes

Each violin plot depicts the empirical correlations between the summary-level genetic associations with the outcomes (dark gray) and the posterior mean of the residual correlations estimated by MR^2 (red) in simulation scenario III—undirected pleiotropy averaged over 50 replicates at different levels of the pleiotropy effect $\theta^A = \{0.25, 0.50, 0.75, 1.00, 1.50, 2.00\}$. Confounding effects on the exposures and outcomes are fixed at $\theta_X^U = 2$ and $\theta_Y^U = 1$, respectively, without correlation between exposures and responses' errors and assuming 100% sample overlap in the summary-level genetic associations with the outcomes. For each side of the violin plot, the vertical black thick line displays the interquartile range, while the black line indicates the median. Black dashed line depicts the theoretical value of residual correlation between summary-level outcomes as a function of θ^A as shown in Equation 8. For more details on the simulation setting, see Table S2.

- Scenario V—dependence: outcomes are simulated with correlated errors, mimicking the effect of non-genetic factors that contribute to their correlation.

In the following, we consider for simplicity only the case of 100% overlapping samples in the genetic associations with the outcomes. Full details regarding the simulation study setup are presented in appendix A. An overview of the simulations setting and the open parameters (θ^A and θ_U^Y in Equation 5, θ_U^X, the effect of the unmeasured confounder U on the exposures, and r_Y, the correlation between individual-level responses' errors in Equation 5) that vary across the simulation scenarios are shown in Table S2. Fixed parameters, including the number of subjects in the individual-level data, the number of IVs, the range of the simulated direct causal effects, the heritability of the exposures, and the proportion of variance explained when simulating the responses, are also detailed in the same table. In supplemental information, we also provide details regarding MR^2 hyper-parameters setting, including the number of MCMC iterations after burn-in, as well as technical details of the alternative methods and their software implementations we used in the simulation study.

The performance in terms of exposure selection is evaluated using the receiver operating characteristic (ROC) curves where the true positive rate (TPR) is plotted against the false positive rate (FPR). As a baseline, all methods perform equally well in the case of no correlation between exposures, as seen in Figure S1. In contrast, when the exposures are correlated, all variable-selection approaches improve over the standard MV-MR, as seen in Figure 3. This improvement depends on the correlation between exposures, as shown in Figure S2.

In the following, we set the correlation between exposures at $r_X = 0.6$, while the confounding effects on the exposures and outcomes are fixed at $\theta_X^U = 2$ and $\theta_Y^U = 1$, respectively. In scenario III—undirected pleiotropy and scenario IV—directed pleiotropy, the pleiotropic effect is set at $\theta^A = 1$. Finally, in scenario V—dependence, the correlation between individual-level responses' errors in Equation 5 is fixed at $r_Y = 0.6$.

When residual correlation is induced by shared undirected (Figure 3B) and directed pleiotropy (Figure 3C), MR^2 shows a better detection of true causal exposures than MR-BMA, which in turn improves over a basic MV-MR model. As shown in Figure 4 for shared undirected pleiotropy and Figure S3 for shared directed pleiotropy, the degree of improvement depends on the strength of the pleiotropic pathway effect. In this scenario, the mSSL approach provides strong sparse solutions with many direct causal effects set to zero. In contrast, MRCE includes many more exposures in the model at the cost of a high FPR. Similar results are observed when the residual correlation between outcomes is induced directly through the

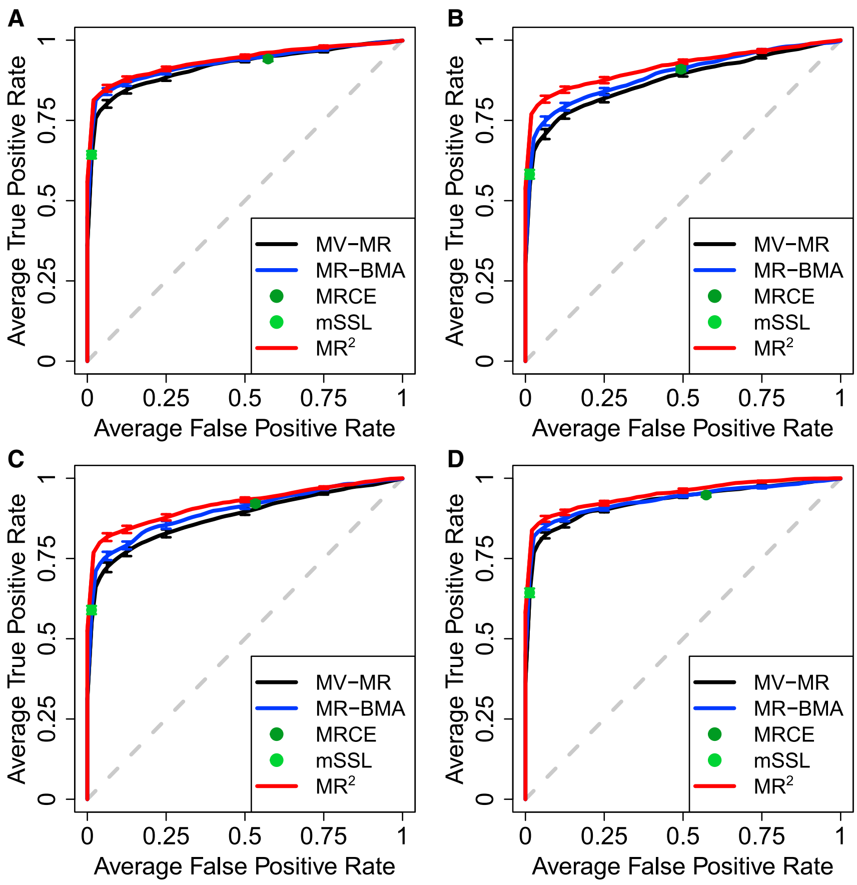

Figure 3. Receiver operating characteristic (ROC) curves in different simulated scenarios when the correlation between exposures is set at $\mathbf{r_X = 0.6}$, averaged over 50 replicates, illustrating the performance of different MR implementations and multi-response multivariable methods to distinguish between true and false causal exposures for five simulated outcomes by plotting the true positive rate (TPR) against the false positive rate (FPR)

(A) Depicts baseline scenario II—confounding where only the confounding effects on the exposures and outcomes, $\theta_X^U = 2$ and $\theta_Y^U = 1$, respectively, are used to simulate the data.

(B–D) Residual correlation induced by shared undirected pleiotropy (scenario III—undirected pleiotropy) and shared directed pleiotropy (scenario IV—directed pleiotropy) with pleiotropic effect set at $\theta^A = 1$ are shown in (B) and (C), respectively. (D) Displays the performance of the different methods in scenario IV—dependence where the correlation between individual-level responses' errors is fixed at $r_Y = 0.6$. Vertical bars in each ROC curve, at specific FPR levels, indicate standard error. For more details on the simulations setting, see Table S2.

correlation between individual-level responses' errors (Figure 3D) with the degree of improvement depending on the level of correlation (Figure S6). For these scenarios, the corresponding area under the ROC curve (AUC) along with standard deviation across 50 replicates is provided in Table 1. When a pleiotropic pathway is simulated, the relative gain compared with one outcome at a time MR methods, i.e., MV-MR and MR-BMA, is around 7.15% and 4.45%, respectively, while it decreases to 2.37% and 1.71% when dependence is simulated. The relative gain is also marked with respect to mLLS and small compared with MRCE due to the opposite sparse Lasso solutions of the two methods, conservative and liberal, respectively. The AUCs of all simulated scenarios across the full range of open parameters are reported in Tables S3–S6.

Next, we compare methods according to their performance in estimating the direct causal effect strength. This is evaluated by the sum of squared errors (SSEs) presented in Table 2 and Figures S7–S11. As can be seen from Table 2, all methods show a comparable performance with negligible SSEs in scenario I—null when there are no causal exposures. In the other scenarios, when there is correlation between the exposures, the largest SSE is consistently seen for the MV-MR approach. This is in keeping with other simulation studies[15] that have shown that MV-MR is unbiased but suffers from large variance. MR^2 has almost everywhere the lowest SSE compared with alternative methods as well as the lowest standard deviation across 50 replicates. The largest improvement with respect to MR-BMA is in scenario III—undirected pleiotropy and scenario IV—directed pleiotropy when the summary-level outcomes are correlated by a shared pleiotropic pathway. The multi-response implementations MRCE and mSSL perform roughly as MR-BMA in terms of SSEs but suffer either from too little (mSSL) or too much (MRCE) sparsity, which biases the direct causal effect estimates. Notably, MR-BMA performs better than both MRCE and mSSL in scenario V—dependence across all ranges of open parameters (Table S11) because the two multi-response multivariable methods are not able to identify the simulated correlation pattern between the responses' errors, thus degrading the estimation of the direct causal effects. Instead, MR^2 can detect it, resulting in the lowest SSE across the full range of open parameters.

We also assess the ability of MR^2 to detect shared and distinct direct causal effects. To this aim, we calculate the proportion of significant causal effects associated with either single or multiple outcomes in the same scenarios presented in Tables 1 and 2, where the correlation between exposures is fixed at $r_X = 0.6$. For a fair comparison, we fix the type I error to be the same in all the methods, and, in particular, we set it at the level detected by mSSL given its sparse Lasso solutions with a low FPR. Specifically, we selected the threshold of Benjamini-Hochberg FDR procedure[53] for MV-MR and mPPI for MR-BMA and MR^2, respectively, to match the number of false positives detected by

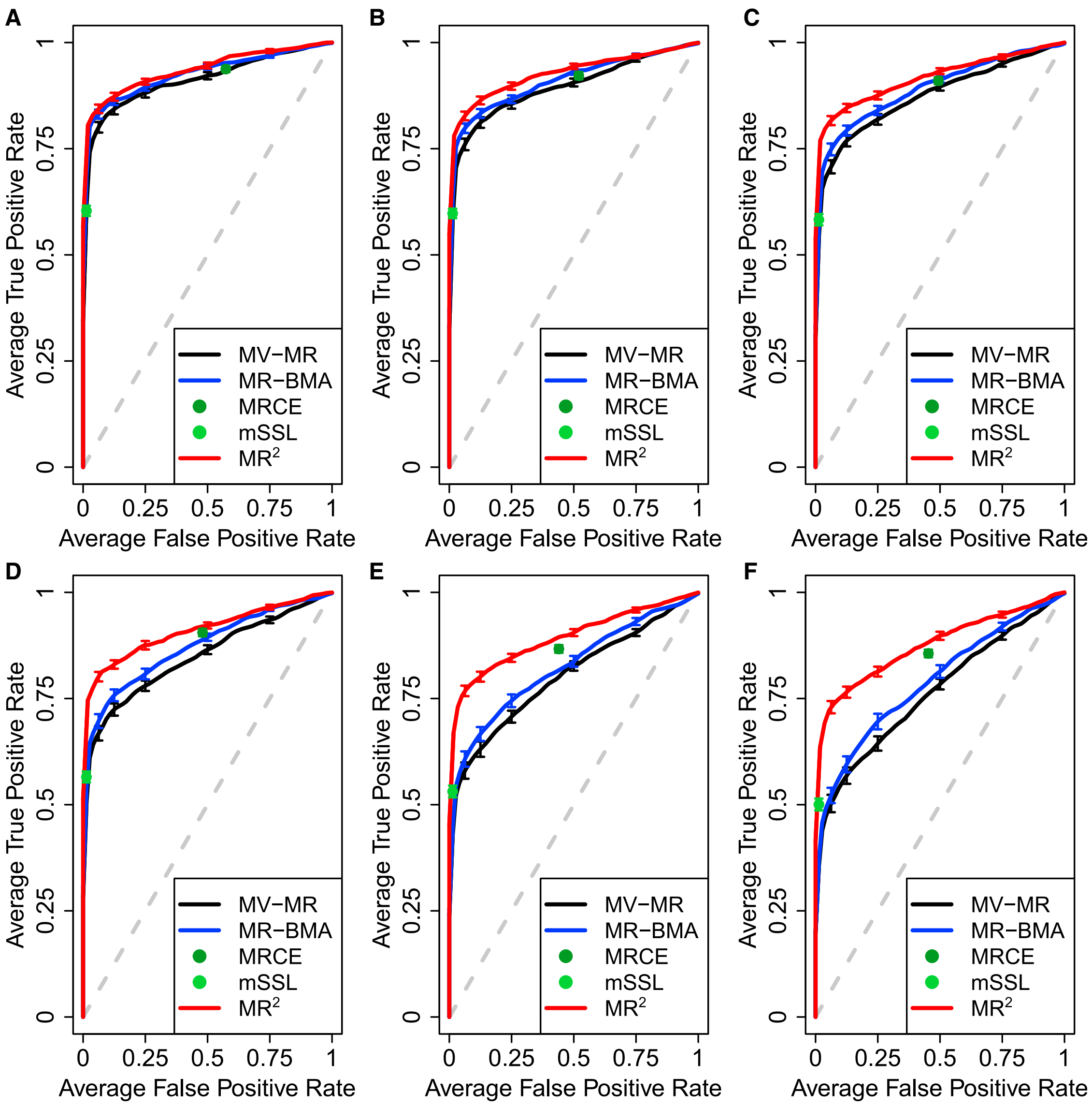

Figure 4. Receiver operating characteristic (ROC) curves for different levels of the pleiotropic pathway effect θ^A and when the correlation between exposures is set at $\mathbf{r_X} = \mathbf{0.6}$, averaged over 50 replicates, illustrating the performance of different MR implementations and multi-response statistical methods to distinguish between true and false causal exposures for five simulated outcomes by plotting the true positive rate (TPR) against the false positive rate (FPR)

Pleiotropic pathway effect varies from (A)–(F) with values $\theta^A = \{0.25, 0.5, 0.75, 1, 1.5, 2\}$. Confounding effects on the exposures and outcomes are fixed at $\theta_X^U = 2$ and $\theta_Y^U = 1$, respectively. Vertical bars in each ROC curve, at specific FPR levels, indicate standard error.

mSSL. This was not possible for MRCE given the fixed solution of the multiple responses Lasso. Table S12 shows the power of the methods considered. MR^2 is the most powerful method to detect distinct direct causal effects, which is also the most likely simulated case (on average 37.3% of all exposure-response combinations), across all simulated scenarios. When the residual correlation between summary-level outcomes is simulated (scenario III—undirected pleiotropy, scenario IV—directed pleiotropy, and scenario V—dependence), MR^2 is also the most powerful method to detect shared direct causal effects. This is apparent when two and three shared direct causal effects are simulated. Together they account for 95% of all possible simulated shared cases. There is a clear advantage over one-response-at-a-time MR methods and less gain with respect to alternative multi-response methods as the number of the shared direct causal effects increases. When four outcomes are associated with the same risk factor, the limited number of simulated cases may not be large enough to discriminate between the power of MR^2 and alternative multi-response methods. Similar results are also obtained for different levels of correlation between individual-level exposures (data not shown).

Finally, results do not change much if the number of raw-level individuals is decreased to $N = 20,000$, of which half is used to generate the summary-level genetic associations for the exposure and half for the outcomes, as well as if in the simulation study the proportion of variance

Table 1. Area under the curve (AUC) in different simulated scenarios when the correlation between exposures is set at $r_X = 0.6$

	Scenario II	Scenario III	Scenario IV	Scenario V
	Confounding	Undirected pleiotropy	Directed pleiotropy	Dependence
MV-MR	0.922 (0.040)	0.850 (0.059)	0.857 (0.057)	0.930 (0.037)
MR-BMA	0.932 (0.034)	0.875 (0.050)	0.876 (0.058)	0.936 (0.032)
MRCE	0.926 (0.035)	0.896 (0.046)	0.904 (0.048)	0.932 (0.037)
mSSL	0.876 (0.044)	0.834 (0.068)	0.840 (0.055)	0.869 (0.067)
MR^2	0.939* (0.033)	0.912* (0.042)	0.917* (0.043)	0.952* (0.031)

The ability to distinguish between true and false causal exposures is evaluated by the area under the ROC curve averaged over 50 replicates with standard deviation across 50 replicates in brackets. In baseline scenario II—confounding, only the confounding effects on the exposures and outcomes, $\theta_X^U = 2$ and $\theta_Y^U = 1$, respectively, are used to induce residual correlation between the responses. Residual correlation induced by shared undirected pleiotropy and shared directed pleiotropy with pleiotropic effect $\theta^A = 1$ are presented in scenario III—undirected pleiotropy and scenario IV—directed pleiotropy, respectively. In scenario IV—dependence, outcomes are simulated with the correlation between individual-level responses' errors fixed at $r_Y = 0.6$. Best results are denoted with an asterisk. For more details on the simulations setting, see Table S2.

explained to generate the outcomes from the exposures is decreased to $h_X = 0.05$. For a visual comparison, interested readers can contrast Figure 4 when $N = 100,000$ and $h_X = 0.10$ with Figures S4 and S5 when $N = 20,000$ and $h_X = 0.10$, and when $N = 100,000$ and $h_X = 0.05$, respectively.

Cardiometabolic exposures for cardiovascular diseases

For the first real application example, as CVDs, we consider AF, CES, CAD, HF, and PAD. Importantly, the summary-level genetic associations for PAD were derived from the Million Veteran Program, and there is no overlap in samples with the other responses, except for CES (data source: GIGASTROKE Consortium + Global Biobanks [including UK Biobank and Million Veteran Program]). Common polygenic exposures were selected according to the National Health Service guidelines on causes for CVD website (https://www.nhs.uk/conditions/coronary-heart-disease/causes/; last reviewed on the March 10, 2020). For high cholesterol, we include five major lipoprotein-related traits (apolipoprotein A1 [ApoA], apolipoprotein B [ApoB], high-density lipoprotein [HDL], low-density lipoprotein [LDL], and triglycerides [TGs]). Obesity is measured by body mass index (BMI); exercising regularly (moderate-to-vigorous intensity exercises during leisure time) is measured by physical activity (PA), defined by moderate-to-vigorous intensity exercises during leisure time; and high blood pressure is measured by systolic blood pressure (SBP). We also include a lifetime smoking index (SMOKING), a composite of smoking initiation, heaviness, duration, and cessation, and type 2 diabetes (T2D) as exposures. Given the strong epidemiological, genetical, and causal relationships between these exposures, an MV-MR design is necessary to account for potential horizontal pleiotropy and to facilitate selection of likely causal exposures. Genetic associations with exposures and outcomes are derived from publicly available summary-level data; see Table S13 for an overview of the data sources.

IVW is performed before the analysis for all outcomes and exposures using weights derived jointly from all responses, as described in appendix A. Moreover, the summary-level genetic associations with the exposures are standardized before the analysis. This allows us to interpret and compare the estimated exposures effect size for each outcome and, more importantly, across outcomes. For a complete description of the pre-processing and IV selection steps, we refer to supplemental information. Briefly, we select $n = 1,540$ independent genetic variants associated with any of the ten exposures as IVs after clumping. Results are obtained after removing outliers or high-leverage and influential observations using scaled CPO and fitting the proposed model on the remaining $n = 1,533$ IVs (see Figure S10).

MR^2 identifies several exposures shared among CVDs, as highlighted in Figures 5A and 5B, which show the mPPI and the posterior mean direct effect sizes (95% CI) for each exposure-outcome combination, respectively. Significant mPPIs and corresponding direct effect estimates are selected, controlling FDR at 5% (see appendix A), which corresponds to mPPIs ≥ 0.77 (Figure S11A). For clarity of presentation, mPPIs and direct effect sizes for non-selected outcome-exposure pairs are not plotted.

Results provided by MR^2 can be read in two different ways: a traditional "vertical" way where, for each outcome, the significant exposures are highlighted. For instance, CAD has four significant exposures, i.e., ApoB, SBP, T2D, and SMOKING in order of importance by looking at the posterior mean direct effect estimates. PAD and HF have the same ones, although in a different order. On the other hand, genetically predicted levels of SBP and BMI are associated with AF, and genetically predicted levels of SBP and risk of T2D are associated with CES, respectively.

The main feature of the proposed methodology is that it allows reading, interpreting, and comparing the results also "horizontally" across outcomes. For instance, ApoB shows the strongest risk-increasing effect on CAD among all exposure-outcome combinations with a posterior mean direct causal effect of 0.48. Moreover, it is also selected for PAD with halved effect estimate (0.23) and around four times lower risk increase for HF (0.11). Similarly, the jPPI can be interpreted as the relevance of an exposure for a group of outcomes. For instance, ApoB has a strong jPPI of 0.78 to be jointly causal for CAD, PAD, and HF (Figure S11C). Taken together, these findings extend the results of a previous study, which found ApoB as a shared exposure for CAD and PAD[17] and for the first time implicates a likely causal role of ApoB also for HF.

MR^2 also provides a more rigorous statistical control of the null hypothesis (no causal effects) because it takes

Table 2. Sum of squared errors (SSEs) in different simulated scenarios when the correlation between exposures is set at $r_X = 0.6$

	Scenario I	Scenario II	Scenario III	Scenario IV	Scenario V
	Null	Confounding	Undirected pleiotropy	Directed pleiotropy	Dependence
MV-MR	0.001 (< 0.001)	1.246 (0.490)	3.869 (1.502)	3.613 (1.409)	1.200 (0.464)
MR-BMA	0.000* (< 0.001)	0.673 (0.302)	1.714 (1.036)	1.508 (0.663)	0.700 (0.348)
MRCE	0.001 (< 0.001)	0.904 (0.516)	1.434 (0.877)	1.172 (0.618)	0.849 (0.423)
mSSL	0.000* (< 0.001)	0.881 (0.374)	1.104 (0.790)	1.050 (0.700)	0.891 (1.167)
MR^2	0.000* (< 0.001)	0.567* (0.269)	0.858* (0.722)	0.821* (0.681)	0.521* (0.278)

The quality of the direct causal effect estimation is evaluated by sum of squared errors (SSEs), averaged over 50 replicates, with standard deviation across 50 replicates in brackets. Scenario I—null is simulated with the correlation between individual-level responses' errors fixed at $r_Y = 0.6$. In baseline scenario II—confounding, only the confounding effects on the exposures and outcomes, $\theta_X^U = 2$ and $\theta_Y^U = 1$, respectively, are used to induce residual correlation between the responses. Residual correlation induced by shared undirected pleiotropy and shared directed pleiotropy with pleiotropic effect $\theta^A = 1$ are presented in scenario III—undirected pleiotropy and scenario IV—directed pleiotropy, respectively. In scenario IV—dependence, outcomes are simulated with the correlation between individual-level responses' errors fixed at $r_Y = 0.6$. Best results are denoted with an asterisk. For more details on the simulations setting, see Table S2.

into account both the conditional independence among exposures (multivariable) and across responses (multi-trait). For example, conditional on ApoB, there is no evidence for any other major lipoprotein-related trait to have a likely causal role for any outcome, and conditional on CAD, PAD, and HF, there is no evidence for ApoB on any other outcome. It is an important positive control that MR^2 selects ApoB as the only lipid trait for CAD. Previous studies using genetic evidence and MV-MR models[17,36,54–56] have shown that ApoB, representing the total number of hepatic-derived atherogenic lipoprotein particles, is the most likely causal lipid determinant of CAD, and the evidence for LDL is attenuated toward the null when accounting for ApoB.

A second example regarding the ability of MR^2 to disentangle complex causal relationships and advance cardiovascular domain knowledge is related to SBP. SBP is shown to increase the risk for all five CVDs considered, with a substantial jPPI of 0.77 for all five outcomes. However, SBP has the strongest posterior mean direct effect on CAD (0.46) and then it decreases steadily across the other responses (PAD [0.26], HF [0.24], AF [0.2], and CES [0.13]), suggesting that SBP lowering may not be a broad therapeutic target (as already shown in randomized clinical trials, [RCTs]) or it would require potent SBP lowering to meaningfully impact (in increasing order) HF, AF, and CES at a population level. Similar considerations can be extended to other exposures selected.

Almost all significant exposures have a large causal effect on CAD and PAD but much lower on HF, AF, and CES, except BMI on HF and AF. Therefore, it seems plausible that these traditional cardiovascular exposures are not able to fully describe the disease etiology of HF and, in particular, AF and CES. In turn, these results suggest that there may be other exposures not considered here as the main causes of these diseases. In contrast, other exposures included in this analysis, such as PA, that although considered an important exposure in medical practice (NHS guidelines), conditionally on all the others, are not significant for any CVD. This suggests that beneficial effects of PA on these outcomes is likely mediated by the other traditional cardiometabolic risk factors.

MR^2 also acknowledges residual correlation among summary-level responses, which is not accounted for by the selected exposures as shown in Figures 5C–5E, which depict the ePPI, the indirect graph estimated by using the selected ePPIs, and the posterior mean (95% CI) of the partial correlations between outcomes, respectively. Significant ePPIs and corresponding partial correlation are selected controlling FDR at 5%, which corresponds to ePPIs ≥ 0.78 (see Figure S11B).

Significant residual dependence between the outcomes not explained by the exposures is identified between CAD and HF and between CAD and PAD with summary-level residual partial correlation of 0.26 and 0.25, respectively, reflecting known vertical and horizontal pleiotropy of CAD being a likely cause of HF[18,19] and horizontal pleiotropy between CAD and PAD. In contrast, PAD and HF show significant but four times lower-level residual partial correlation (0.06). Other important residual partial correlations highlight the disease pathway HF-AF-CES, as illustrated in Figure 5C. Compared with the empirical partial correlations between summary-level genetic associations with the responses without conditioning on the exposures (Figure S8B), the genetically predicted levels of exposures are able to explain around 32% and 21% of the summary-level partial correlation between CAD and PAD and between CAD and HF, respectively, and almost 62% of the residual correlation between PAD and HF. However, not all exposures contribute in the same way to this remarkable decrease. ApoB seems not as important (jPPI = 0.8) as the other associated exposures (jPPI > 0.96) to explain the dependence between PAD and HF (see Figure S11C). Finally, a little reduction is observed for the disease pathway HF-AF-CES, supporting the earlier hypothesis that other important shared exposures may be missing from the proposed MR model. However, as mentioned earlier and shown in Equation 8, we cannot rule out that, besides shared pleiotropy, some non-genetic factors may be responsible for the observed residual correlation.

We conclude this section comparing the results obtained by MR^2 with existing MV-MR methods (see Table S1), including MV-MR-Egger[28] to confirm that, when dealing with multiple outcomes, there are different assumptions regarding the effect of the unmeasured pleiotropy and MV-MR-Egger may not able to detect it.

MV-MR is not able to identify any lipid exposure for any outcome considered except for TGs for AF at 5%

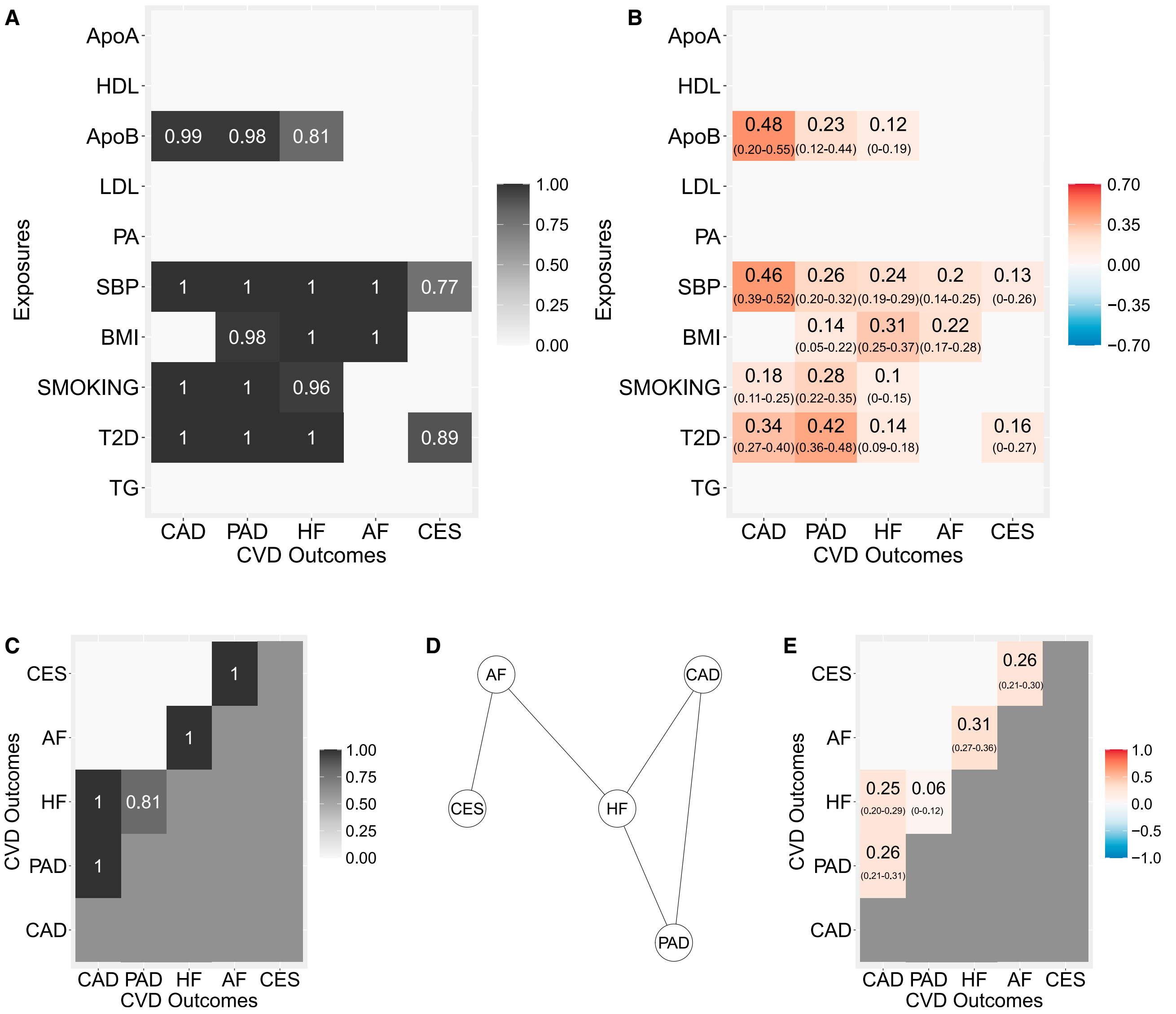

Figure 5. Results of the multivariable multi-response MR^2 model in application example 1 on common exposures for cardiovascular disease outcomes (CVDs)

(A) Marginal posterior probability of inclusion (mPPI) of each exposure (y axis) against each outcome (x axis). Selected mPPIs for each exposure indicate whether an exposure is shared or distinct among multiple CVDs.

(B) Posterior mean (95% credible interval) of the direct causal effect of each exposure (y axis) against each outcome (x axis). For clarity of presentation, mPPIs and estimated direct effect sizes for non-selected outcome-exposure pairs (mPPI $<$ 0.77 at 5% FDR) are not plotted.

(C) Edge posterior probability of inclusion (ePPI) among outcomes. Only the upper triangular matrix is depicted.

(D) Undirected graph estimated by using the selected ePPIs showing the residual dependence between outcomes not explained by the exposures.

(E) Posterior mean (95% credible interval) of the partial correlations between outcomes. For clarity of presentation, ePPIs and partial correlations for non-selected outcomes pairs (ePPI $<$ 0.78 at 5% FDR) are not plotted. Only the upper triangular matrix is depicted.

Benjamini-Hochberg FDR (Table S14). MV-MR is not designed for the analysis of many exposures that are highly correlated.[15] Indeed, multi-collinearity reduces the precision of the estimated direct causal effects, which weakens the statistical power of the MV-MR model. Due to the strong correlation between genetic associations with lipid exposures (Figure S8C), MV-MR misses ApoB as likely causal exposure for CAD, PAD, and HF. Consequently, an intersection-union test[57] will miss ApoB as an important risk factor jointly for CAD, PAD, and HF in contrast with MR^2, which assigns to ApoB a jPPI $=$ 0.78 for this group of responses. Similar results in terms of exposures selected and effect sizes are seen when MV-MR-Egger is used, with a significant unmeasured horizontal pleiotropy identified only in CES (Table S15). Note that neither MV-MR nor MV-MR-Egger can provide a clear picture of the effect size of SBP across disease outcomes, with much larger effect estimates and a narrow difference between the largest (CAD) and the smallest (CES).

MR-BMA is not able to draw a clear distinction between the exposures selected and excluded, as shown in

Table S16. This is exemplified in CAD where the exposures included depend on the multiple testing correction applied. When using a strict Bonferroni threshold, MR-BMA identifies, in decreasing order, SBP, SMOKING, T2D, and ApoB with 0.66 as the smallest mPPI, while with a more lenient FDR threshold HDL, LDL, and BMI are also included with the smallest mPPI of 0.29. Regarding the other outcomes, the order of importance of the exposures is different from MR^2 with remarkably larger model-averaged causal effect estimates (MACEs) for PAD and HF. MR-BMA suffers the same problems as MV-MR and MV-MR-Egger regarding the magnitude of the direct causal effect estimates of SBP on the outcomes, demonstrating that this problem affects all single-trait methods regardless of their implementation. Interestingly, MR-BMA does not identify T2D as an exposure for CES, which is instead detected by MR^2, MV-MR, and MV-MR-Egger, favoring in contrast BMI.

Lipidomic risk factors for cardiovascular diseases

The first application example prioritizes ApoB as a shared exposure for three out of five CVDs. Moreover, conditional on ApoB, no other major lipoprotein-related trait has a likely causal role for any outcome. Our next step is to better understand molecular determinants of ApoB by considering ten ApoB-containing lipoprotein subfractions of different sizes, ranging from small-large to extra-extra-large, very-large-density lipoproteins, measured using nuclear magnetic resonance (NMR) spectroscopy.[58] In particular, we are considering S.LDL.Ps, small large-density lipoprotein particles; M.LDL.Ps, medium large-density lipoprotein particles; L.LDL.Ps, large large-density lipoprotein particles; IDL.Ps, intermediate-density lipoprotein particles; XS.VLDL.Ps, extra-small very-large-density lipoprotein particles; S.VLDL.Ps, small very-large-density lipoprotein particles; M.VLDL.Ps, medium very-large-density lipoprotein particles; L.VLDL.Ps: large very-large-density lipoprotein particles; XL.VLDL.Ps: extra-large very-large-density lipoprotein particles; and XXL.VLDL.Ps, extra-extra-large very-large-density lipoprotein particles. Identification of specific subfractions to different CVD manifestations can help our understanding of the pathophysiology of the disease and provide insights into pathophysiology, molecular mechanisms, risk stratification, and treatment.[17] However, performing MR using metabolites as exposures is a difficult task given the strong correlation and intricate dependence that exist between them (see Figures S13B and S13C).

For a description of the pre-processing, including IVW and instrument selection steps, we refer to supplemental information. Briefly, given the prior hypothesis of ApoB as the leading exposure for CVDs, we select genetic variants associated with ApoB in UK Biobank at genome-wide significance, resulting in $n = 148$ IVs after clumping. This three-sample MR design is known to reduce bias due to winner's curse bias.[59] Results are obtained after removing outliers or high-leverage and influential observations using scaled CPO and fitting the proposed model on $n = 141$ IVs (see Figure S15).

When lipidomic risk factors are used for CVDs, results obtained by MR^2 are very sparse with few significant direct causal effects at 5% FDR (mPPI < 0.29). Moreover, the separation between significant and non-significant causal associations is difficult (see Figure S16A). The proposed model identifies XS.VLDL.Ps as a shared exposure for both PAD and HF with mPPIs of 0.32 and 0.31, respectively, and a jPPI of 0.14 (Figure S16C) with direct causal effects of 0.15 and 0.13 (Figures 6A and 6B). Distinct exposures are detected for CAD, where smaller particle sizes, IDL.Ps and L.LDL.Ps, are prioritized with mPPIs of 0.41 and 0.59 and direct causal effects of 0.4 and 0.63, respectively. No subfractions are identified for the other disease outcomes.

In contrast with the previous application example, when dealing with molecular exposures, CIs are large, confirming the complexity of the analysis. For the strongest signal, L.LDL.P-CAD combination, the 95% CI ranges between 0 and 1.41. This is due to a combined effect of multi-collinearity (although the designed independent prior in Equation 13 protects against it; see appendix A) and the small number of genetic variants associated with ApoB in UK Biobank. Thus, while L.LDL.Ps (or IDL.Ps) could be a likely cause for CAD, the large CI suggests caution.

As expected, there is a substantial residual partial correlation in almost all combinations of summary-level outcomes, as depicted in Figures 6C and 6D, highlighting the existence of non-lipoprotein pleiotropic pathways between these traits not intercepted by the selected exposures (SBP, BMI, SMOKING, T2D pathways are missing by design) and, possibly, the effects of non-genetic factors on the responses.

When high levels of residual correlation are present and cannot be "explained away" or be "accounted for" by the exposures, the advantage of an MR multi-response model to reduce false positives is evident. For instance, MR-BMA (Table S19) identifies genetically predicted levels of L.VLDL.Ps to be associated also with AF, possibly because AF and CAD are correlated at the summary level (Figure S13). Similarly, the causal effect of S.VLDL.Ps on AF is likely a false positive by looking at its small MACE. MV-MR is not able to identify any exposure for any outcome that is significant after multiple testing correction (Table S17), even with a less conservative Benjamini-Hochberg FDR procedure, demonstrating that standard methodology is not adapted to handle highly correlated exposure data. Similar conclusions can be extended to MV-MR-Egger (Table S18) with no significant intercept identified for any outcome.

Discussion

Here, we present MR^2, an MR design to analyze multiple related outcomes in a joint model and to define shared

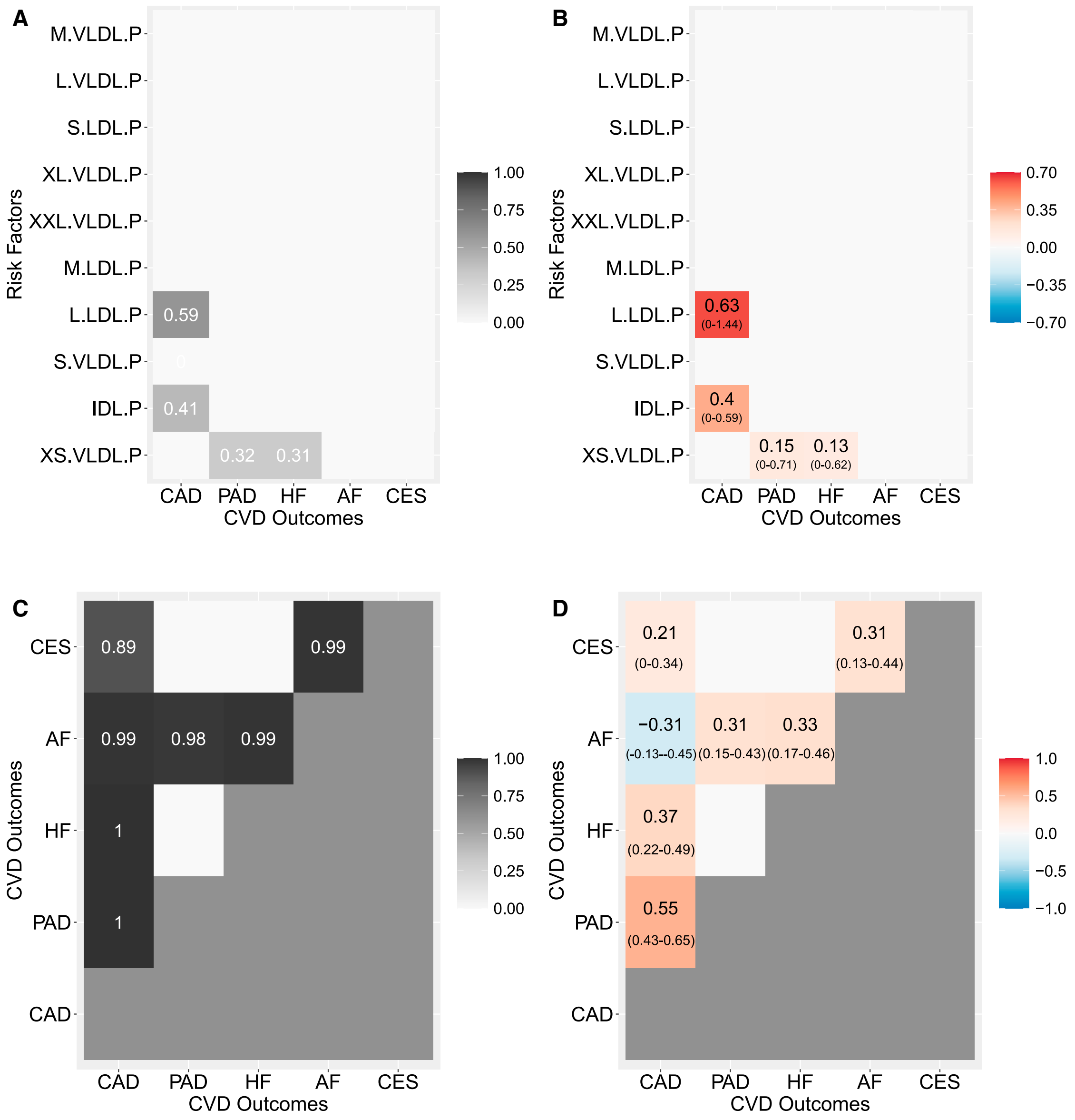

Figure 6. Results of the multi-response MR (MR^2) model in application example 2 on molecular exposures for cardiovascular disease outcomes (CVDs)

(A) Marginal posterior probability of inclusion (mPPI) of each exposure (y axis) against each outcome (x axis). Selected mPPIs for each exposure indicate whether an exposure is shared or distinct among multiple CVDs.
(B) Posterior mean (95% credible interval) of the direct causal effect of each exposure (y axis) against each outcome (x axis). For clarity of presentation, mPPIs and estimated effect sizes for non-selected outcome-exposure pairs (mPPI < 0.29 at 5% FDR) are not plotted.
(C) Edge posterior probability of inclusion (ePPI) among outcomes. Only the upper triangular matrix is depicted.
(D) Posterior mean (95% credible interval) of the partial correlations between outcomes. For clarity of presentation, ePPIs and partial correlations for non-selected outcomes pairs (ePPI < 0.85 at 5% FDR) are not plotted. Only the upper triangular matrix is depicted.

and distinct causes of related health outcomes. Based on a Bayesian copula regression model, MR^2 detects causal effects while estimating the residual correlation between MV-MR models for each outcome and vice versa. Thus, the proposed model makes the estimated causal effects robust to the residual correlation induced by shared pleiotropy. MR^2 is formulated on the summary level where the genetic variants used as IVs are considered observations. Residual correlation in the proposed model is consequently interpreted as the correlation between summary-level outcomes measured on the IVs not accounted for by the genetic associations with the exposures. We show, both theoretically and in a simulation example, how unmeasured shared pleiotropy induces residual correlation between summary-level genetic associations with the outcomes.

While residual diagnostics is an important strategy in summary-level univariable MR models to detect horizontal pleiotropy[51,60] affecting one outcome, only multi-response MR models, like MR^2, can detect how much cross-variation between outcomes is unexplained after accounting for the exposures of interest. We show in an

extensive simulation study that MR^2 has more power to detect true causal exposures, yields a better separation between causal and non-causal exposures, and improves the accuracy of the effect estimation over existing MV-MR methods that consider only one outcome at a time, such as MV-MR and MR-BMA. Moreover, MR^2 demonstrates more power when an exposure is causal for more than one outcome.

Thanks to the formulation as a joint multi-response model, MR^2 can distinguish between shared and distinct exposures for the disease, which is essential to define interventions that reduce the risk of more than one disease. We illustrate this in our application examples considering five CVDs. Multi-response models like MR^2 are a necessary contribution to better understanding the causes of multimorbidity. In particular, the discovery of shared and distinct causes of diseases may help define interventions with co-benefits, i.e., interventions that reduce the risk of more than one outcome. For instance, in our first application example, we have identified ApoB as likely causal exposure for CAD, PAD, and HF even when accounting for other lipoprotein measures including LDL cholesterol. These results of the applied analyses, considering effects of cardiometabolic risk factors on cardiovascular disease subtypes, are consistent with the existing epidemiological literature.[61] Cholesterol is integral to the development of atherosclerosis and penetrates the arterial wall within those ApoB-containing lipoprotein particles that are small enough to pass to the *tunica intima* from the circulation; these particles include small VLDL, IDL, and LDL particles as well as lipoprotein(a). Taken together, this body of evidence suggests that the lipid content of the particles is secondary to ApoB.[55] However, these results do not invalidate LDL cholesterol as a causal risk factor for the cardiovascular outcomes, as LDL particles also contain an ApoB molecule.[55]

While ApoB is an established exposure for CAD and PAD based on genetic evidence,[17,36,56] we demonstrate that there is an independent effect of ApoB also on HF. This has the following two implications: for one, existing lipid-lowering therapies should be evaluated in terms of their impact on reducing ApoB, and second, future lipid-lowering therapies may be better tailored to reduce ApoB concentration. Moreover, this highlights the importance of including various disease endpoints in RCTs because the intervention may have benefits not just for the main disease of interest but also for other related diseases.

In our application examples, we discover residual correlation between CAD and PAD and between CAD and HF, as well as between the disease pathway HF-AF-CES, which was not accounted for by either common cardiometabolic disease exposure, including lipid traits, blood pressure, and obesity, or by lipid characteristics measured using NMR spectroscopy. Important contributors to the residual correlation when considering common cardiovascular disease exposures are molecular pathways, which are not accounted for when considering traits like ApoB or obesity. These pathways, such as inflammation or stress response, are highly polygenic with hundreds of independent regions in the genome associated with them. Another source of residual correlation between outcomes is the consequence of non-genetic factors that act exclusively on the responses. Another possible source of the observed residual correlation can be mediation, where genetic predisposition for one outcome may cause another, as may be the case for CAD and HF, as suggested, for example, by the Framingham Heart Study.[19] Finally, sample overlap between the summary-level data of the outcomes may contribute to residual correlation, but it is not a necessary condition. As we show in extension of the simulation study (Figure S7) and we observed in the application examples, a residual correlation exists even if responses' samples are non-overlapping. For example, we detect a substantial residual correlation between CAD, which was derived from the CARDIOGRAM Consortium and UK Biobank,[62] and PAD, which was derived from the Million Veteran Program,[63] where there is no overlap in the samples between these two outcomes. Sample overlap is a very common feature when working with summary-level data that are commonly built by integrating all available public data resources and, in particular, from large-scale biobanks.[30]

There are also limitations to our work. First of all, weak instrument bias is toward the null in univariable MR[64] but can go toward any direction for MV-MR depending on the correlation between exposures.[15] A necessary future extension of the approach is to make MR^2 more robust concerning weak instruments.[65] Second, special care needs to be taken when selecting genetic variants as IVs. Importantly, the interpretation of any MR model is conditional on the IVs selected. For example, in our second data example, we have selected genetic variants based on their association with ApoB, which we identified in our primary analysis. Therefore, results need to be interpreted considering this choice and may differ when re-selecting, for example, based on LDL cholesterol. In practice, we recommend following the guidelines for reporting MR studies[66,67] for further details. Similarly, dedicated domain knowledge is necessary when deciding which exposures to include and whether a trait is more suitable as exposure or as outcomes. For example, in the first application example, we include genetic liability to T2D as an exposure because diabetes is considered a risk factor for CAD in the NHS guidelines. Yet, being a case-control phenotype, it may be considered an outcome as well. Regarding the required number of IVs, MR^2 can only be used when there are enough IVs that allow the estimation of the main parameters of interest. As a rule of thumb, for each response, the number of IVs n should be greater than the number of exposures p. Moreover, n should be also large enough to permit the estimation of $\delta_k|Y$, $k = 1, \ldots, q$, and the lower triangular matrix of $R|Y$. A conservative choice would be

$n > q\times(p+1) + q\times(q-1)/2$ with q the number of responses. However, given the sparseness assumption, for each response, the number of important direct causal effects is much smaller than p, and the number of non-zero cells in $R|Y$ is smaller than $q(q-1)/2$. In addition, in real applications, the required number of IVs should not pose any problems when considering polygenic exposures because it is usually much larger than the whole number of exposures and responses.

The development of multi-response MR models on the individual level is another important future direction given the availability of large-scale biobanks with sufficient follow-up time allowing for the development of multiple disease outcomes. Such an analysis would let us study the presence of more than one disease in the same individual instead of analyzing the shared genetic basis of the outcomes as in our current work.

While our study has been motivated to detect common causal exposures for multi-morbid health conditions, MR^2 can be applied to any type of related outcomes. Potential application examples may include molecular biomarkers as outcomes; for example, a recent study has investigated the effect of sleep deprivation on the metabolome[68] or of morning cortisol levels on inflammatory cytokines.[69] Similarly, MR^2 can be used to define exposures for heritable imaging phenotypes measured on the brain,[70] heart,[71] or body composition.[72]

In conclusion, we present here MR^2, the first summary-level MR method that can model multiple outcomes jointly and account for residual correlation between the outcomes. Moreover, MR^2 can distinguish between shared or distinct causes of diseases, enhancing our understanding regarding which interventions can target more than one disease outcome.

Appendix A

IVW for multiple responses

When considering one outcome at a time, the univariable MR model in Equation 1 and MV-MR model in Equation 2 weight a genetic variant $i = 1,\dots,n$, used as IV, by the standard error of the genetic association with the outcome $\mathrm{se}(\beta_{Y_i})$. These weights are also known as the "first-order weights."[73]

In order to generalize the IVW for multiple outcomes, we note that the standard error of the genetic association with a binary trait Y of a genetic variant i, with minor allele frequency (MAF) MAF_i, is[74]

$$\mathrm{se}\left(\beta_{Y_i}\right) = \{N \times pr(1-pr) \times 2\mathrm{MAF}_i(1-\mathrm{MAF}_i)\}^{-1/2}, \quad \text{(Equation A1)}$$

where pr is the percentage of cases and N is the total sample size. In Equation A1, the only quantity that depends on the genetic variant i is the MAF MAF_i, which may be considered as comparable when taking summary-level data from cohorts with similar ethnicity. All other parameters are specific for trait Y and do not depend on the genetic variant i and, consequently, are not relevant when weighting individually the genetic variants.

When considering q outcomes, we propose to use as weight ω_i, $i = 1,\dots,n$, the mean of the q standard errors of the genetic associations with each outcome, i.e., $\omega_i = \sum_{k=1}^{q}\mathrm{se}(\beta_{Y_{ik}})/q$, which essentially, under the hypothesis of cohorts with similar ethnicity, will result in weighting by the average of the inverse of the genetic variant standard errors defined by $\{2\mathrm{MAF}_i(1-\mathrm{MAF}_i)\}^{1/2}$.

Bayesian multi-response MR

An alternative way to the SUR model in Equation 3 to model jointly the q-related outcomes is to describe their dependence through a copula function. A q-variate function $C(u_1,\dots,u_q)$, where $C:[0,1]^q \to [0,1]$, is called a copula if it is a continuous distribution function and each marginal is a uniform distribution function on [0,1]. If $F_{Y_1}(\cdot),\dots,F_{Y_q}(\cdot)$ are the marginal cumulative density functions (cdfs) of the random variables $Y_1,\dots,Y_q$, their joint cdf can be described through a specific copula function C as

$$F_{Y_1,\dots,Y_q}\left(y_1,\dots,y_q\right) = C\left\{F_{Y_1}(y_1),\dots,F_{Y_q}\left(y_q\right)\right\}.$$

The Gaussian copula C is described through the function

$$C(u_1,\dots,u_q;R) = \Phi_q\{\Phi^{-1}(u_1),\dots,\Phi^{-1}(u_q);R\},$$

where $\Phi_q(\cdot;R)$ is the cdf of a q-variate Gaussian distribution with zero mean vector and correlation matrix R and $\Phi^{-1}(\cdot)$ is the inverse of the univariate standard Gaussian cdf. If all the marginal distributions are continuous, the matrix R can be interpreted as the correlation matrix of the elements of Y and zeros in its inverse imply the conditional independence between the corresponding elements of the responses.

The GCR model is described by the transformation

$$y_{ik} = h_{ik}^{-1}(z_{ik}),\ Z_i \overset{\text{iid}}{\sim} \mathrm{N}_q(0,R), \quad \text{(Equation A2)}$$

where $z_{i1},\dots,z_{iq}$ are realizations from Z_i and $h_{ik}(\cdot) = \Phi^{-1}\{F_k(\cdot;x_{ik},\theta_k,\sigma_k^2)\}$ for each $i = 1,\dots,n$ and $k = 1,\dots,q$, x_{ik} is the p-dimensional vector of predictors for the ith sample and the kth response, and $\theta_k = (\theta_{k1},\dots,\theta_{kp})^T$ is a p-dimensional vector of regression coefficients.

A detailed discussion of the Bayesian formulation of the GCR model in Equation A2 with multiple responses of any type is presented in ref. [40]. In the following, we summarize the main aspects of the proposed multi-response MV-MR model when all margins are univariate Gaussian. Details of the MCMC algorithm are presented in supplemental information.

The SUR model is a special case of Equation A2 when all margins are univariate Gaussian with mean $x_{ik}\theta_k$ and variance σ_k^2 and R is the correlation matrix. Thus, Equations 3 and 4 can be written in terms of a GCR model as

$$\beta_{Y_{ik}} = h_{ik}^{-1}(z_{ik}),\ Z_i \overset{\text{iid}}{\sim} \mathrm{N}_q(0, R), \qquad \text{(Equation A3)}$$

with $h_{ik}(\cdot) = \Phi^{-1}\{\Phi(\cdot; \beta_{X_i}, \theta_k, \delta_k^2\}$ or, equivalently,

$$\beta_{Y_{ik}} = \beta_{X_i}\theta_k + \delta_k z_{ik} \qquad \text{(Equation A4)}$$

for each $i = 1, \ldots, n$ and $k = 1, \ldots, q$ and with R as in Equation 4.

Priors specification

The selection of important exposures is achieved by utilizing a binary latent vector $\gamma_k = (\gamma_{k1}, \ldots, \gamma_{kp})$, $k = 1, \ldots, q$, where γ_{kj} is 1 if, for the jth exposure and the kth response, the effect estimate is different from zero and 0 otherwise. Specifically, we assume that for each k, the effects in Equation A4 are distributed as

$$\theta_{kj}\Big|\gamma_{kj} \sim \Big(1 - \gamma_{kj}\Big)\delta_0 + \gamma_{kj}\mathrm{N}(0, v),\ j = 1, \ldots, p, \qquad \text{(Equation A5)}$$

where δ_0 denotes a point mass at zero and v is a fixed value. Sparsity for the selection of important exposures for the kth response is enforced by specifying the hierarchical structure

$$\gamma_{kj}\big|\pi_k \overset{\text{iid}}{\sim} \mathrm{Ber}(\pi_k),\ j = 1, \ldots, p \qquad \text{(Equation A6)}$$

$$\pi_k \sim \mathrm{Beta}(a_k, b_k).$$

Integrating out π_k in Equation A6, it is readily shown that marginally

$$p(\gamma_k) = \frac{\mathrm{B}(a_k + |\gamma_k|, b_k + p - |\gamma_k|)}{\mathrm{B}(a_k, b_k)}, \qquad \text{(Equation A7)}$$

where $\mathrm{B}(\cdot,\cdot)$ is the beta function and $|\gamma_k| = \sum_{j=1}^{p}\gamma_{kj}$. The hyperparameters a_k and b_k can be chosen using prior information about the average number of important exposures associated with the kth response and its variance.

Because the GCR model in Equation A3 is defined through the correlation matrix R, we need first to expand R into a covariance matrix. We define the transformation $W = Z\Delta$, where Z is the $(n \times q)$-dimensional matrix of the Gaussian latent variables obtained by inverting Equation A4

$$z_{ik} = \frac{\beta_{Y_{ik}} - \beta_{X_i}\theta_k}{\delta_k}$$

and Δ is a $q \times q$ diagonal matrix with elements δ_k, $k = 1, \ldots, q$. Then,

$$\mathrm{vec}(W) \sim \mathrm{N}_{nq}(0, I_n \otimes \Sigma), \qquad \text{(Equation A8)}$$

where $\Sigma = \Delta R \Delta$ and I_n is a diagonal matrix of dimension n that encodes the independence assumption among the genetic variants G (achieved by pruning). In this framework, the correlation matrix can be obtained by the inverse transformation $R = \Delta^{-1}\Sigma\Delta^{-1}$. We utilize the theory of decomposable graphical models[42] to perform a conjugate analysis of the covariance structure of the model because the hyper-inverse Wishart distribution is a conjugate prior distribution for the covariance matrix Σ with respect to the symmetric adjacency matrix G of a decomposable graph $\mathcal{G}$.

Finally, we assign the following prior structure on the symmetric adjacency matrix G and Δ. Let g_ℓ, $\ell = 1, \ldots, q(q-1)/2$, be the binary indicator for the presence of the ℓth off-diagonal edge in the lower triangular part of the symmetric adjacency matrix G of the decomposable graph $\mathcal{G}$. To select important elements of the inverse covariance matrix, we assume the sparsity prior

$$g_\ell \overset{\text{iid}}{\sim} \mathrm{Ber}(\pi_{\mathrm{G}}),\ \pi_{\mathrm{G}} \sim \mathrm{Unif}(0, 1),\ \ell = 1, \ldots, q(q-1)/2. \qquad \text{(Equation A9)}$$

We denote by $p(\mathrm{G})$ the induced marginal prior on the symmetric adjacency matrix and define the joint distribution of Δ, R, and G as $p(\Delta, R, \mathrm{G}) = p(\Delta|R)p(R|\mathrm{G})p(\mathrm{G})$, where

$$\delta_k\big|r^{kk} \sim \mathrm{IGam}\{(q+1)/2, (R^{-1})_{kk}/2\},\ k = 1, \ldots, q, \qquad \text{(Equation A10)}$$

with $(R^{-1})_{kk}$ the kth diagonal element of R^{-1}.[75]

Outliers, high-leverage and influential observations

Detection of outliers is performed by using the CPO[52] to detect genetic variants G_i, $i = 1, \ldots, n$, as outliers in the multi-response MR model. The CPO is also known as the leave-one-out cross-validation predictive density,[76] which, for the multi-response MR model in Equation A3, is defined as

$$\begin{aligned} p(\beta_{Y_i}|\beta_{Y_{-i}}, \beta_X) &= \int p(\beta_{Y_i}|\beta_{X_i}, \Psi)p(\Psi|\beta_{Y_{-i}}, \beta_{X_{-i}})d\Psi \\ &= \mathbb{E}_{\Psi|\beta_{Y_{-i}}, \beta_{X_{-i}}}\left\{p(\beta_{Y_i}|\beta_{X_i}, \Psi)^{-1}\right\}^{-1}, \end{aligned}$$

where $(\beta_{Y_i}, \beta_{X_i})$ and $(\beta_{Y_{-i}}, \beta_{X_{-i}})$ are the IVW-standardized summary-level associations with the genetic variant G_i and all the remaining genetic variants, respectively, and Ψ is the whole parameter space $\Psi = \{\Gamma, \Theta, \mathrm{G}, \Delta, R\}$ with $\Gamma = (\gamma_1, \ldots, \gamma_q)^T$ and $\Theta = (\theta_1, \ldots, \theta_q)^T$.

The CPO describes the posterior probability of observing the q-dimensional vector of values of β_{Y_i} when the model is fitted to all data except β_{Y_i}, with a larger value implying a better fit of the model for β_{Y_i} and a very low CPO value suggesting that the summary-level associations with the genetic variant G_i are either an outlier or a high-leverage and influential observation.

The CPO can be calculated by using the output of the MCMC algorithm. Let T be the number of recorded MCMC iterations. By considering the inverse likelihood across T iterations, the estimated CPO for each genetic variant G_i, $i = 1, \ldots, n$, is

$$\mathrm{CPO}_i = \frac{T}{\sum_{t=1}^{T} p\left(\beta_{Y_i}|\beta_{X_i}\Psi^{(t)}\right)^{-1}}, \qquad \text{(Equation A11)}$$

where $\Psi^{(t)}$ is tth posterior sample of Ψ obtained from the MCMC algorithm. Thus, the Monte Carlo estimate of the

CPO is obtained without actually omitting $(\beta_{Y_i}, \beta_{X_i})$ from the estimation of the posterior distribution of Ψ and is provided by the harmonic mean of the likelihood.[77]

Regarding the threshold for the detection of outliers or high-leverage and influential observations, log-inverse CPOs larger than 40 can be considered as possible outliers and higher than 70 as extreme values.[78] Congdon[79] recommends scaling CPOs by dividing each one by its individual maximum (likelihood) recorded across the MCMC iterations and considering observations with scaled CPOs under 0.01 to be outliers or high-leverage and influential observations. If few CPOs are less than 0.01, the model is considered to fit adequately.

FDR

The mPPI, which measures the strength of direct causal association between each exposure-response combination, and the ePPI, which describes the strength of the residual conditional correlation between each responses' pair, are utilized to select significant exposure-response combinations and important pairs of dependent responses.

Let

$$\text{mPPI}_h \sim \pi_0 \text{Beta}(a_0, b_0) + \pi_1 \text{Beta}(a_1, b_1),\ h = 1, \dots, pq, \quad \text{(Equation A12)}$$

be the two-component mixture model that classifies the mPPI for each exposure-response combination into the null (H_0) or the alternative distribution (H_1) parameterized as beta densities with parameters (a_0, b_0) and (a_1, b_1), respectively, with weights $\pi_0, \pi_1 \geq 0$ and $\pi_0 + \pi_1 = 1$.

In the real application examples, the parameters of the mixture model in Equation A12 are estimated using the expectation-maximization algorithm.[80] The posterior probability of allocation to the alternative hypothesis of each mPPI is

$$\mathbb{P}(\text{mPPI}_h | H_1) = \frac{\pi_1 \text{Beta}(\text{mPPI}_h; a_1, b_1)}{\pi_0 \text{Beta}(\text{mPPI}_h; a_0, b_0) + \pi_1 \text{Beta}(\text{mPPI}_h; a_1, b_1)}$$

$$= \pi_h^{H_1}.$$

Finally, let $\{\pi_{(h)}^{H_1}\}$, $h = 1, \dots, pq$, the sequence in decreasing order of the posterior probabilities of allocation to the alternative hypothesis of the mPPIs for each exposure-response combination. Significant mPPIs are chosen such that

$$\arg\max_h \sum_h \pi_{(h)}^{H_1} \leq \text{FDR},$$

where FDR is the designed level.

Simulation study

Our simulation study is formulated in a summary-level MR design, where $N = 100,000$ independent individuals are simulated, of which half are used to compute the genetic associations with the exposures and half to compute the genetic associations with the outcomes. In the following, we indicate with the subscripts "X" and "Y" the relevant quantities that are associated with the exposures and the responses, respectively. For instance, N_X and N_Y indicate the sample size used in the genetic associations with the exposures and the outcomes, respectively. Moreover, we assume that the quantitative outcomes Y_k, $k = 1, \dots, q$ are measured on the same individuals N_Y.

In all simulation scenarios, we consider $p = 15$ exposures, $q = 5$ outcomes, and $n = 100$ independent genetic variants as IVs. Genotypes for the ith genetic variant and each individual l are simulated independently according to a binomial distribution with MAF equal to 0.05, i.e., $G_{li} \overset{\text{iid}}{\sim} \text{Bin}(2, 0.05)$, $l = 1, \dots, N$, and $i = 1, \dots, n$. Without loss of generality, the resulting matrix of genotypes G is split into two equally sized groups, G_X and G_Y, of dimension $N_X \times n$ and $N_Y \times n$ with $N_X = N_Y = N/2$, respectively. All genetic variants are considered with equal weights, and thus no IVW is needed given that the same MAF at 5% is used to simulate the genotypes.

Overall, the data-generation process consists of two steps. In the first step, the raw data for the exposures X and the responses Y are simulated. Then, in the second step, summary-level data are obtained as the regression coefficients $\beta_{X_{ij}}$, $i = 1, \dots, n$, $j = 1, \dots, p$, from a univariable regression in which the exposure X_j is regressed on the genetic variant G_i in sample one and the regression coefficients $\beta_{Y_{ik}}$, $i = 1, \dots, n$, $k = 1, \dots, q$ from a univariable regression in which the outcome Y_k is regressed on the genetic variant G_i in sample two. In the following, we detail each step and how we simulate the quantities involved.

For the first stage of the simulation study, the jth exposure, $j = 1, \dots, p$, is generated by

$$X_j = G_X \beta_{X_j} + U_X \theta_X^U + \varepsilon_j, \quad \text{(Equation A13)}$$

where G_X and U_X are the matrix of genotypes of the n IVs and the confounder U measured on the same $N_X = 50,000$ individuals, respectively, and $\varepsilon_j \sim \text{N}_{N_X}(0, H_{X_j} I_{N_X})$. H_{X_j} is the jth diagonal element of the $(p \times p)$-dimensional matrix $H_X = \frac{1-h_X}{h_X} \left(G_X \beta_X + U_X \theta_X^U 1_p^T\right)^T \left(G_X \beta_X + U_X \theta_X^U 1_p^T\right)$ with 1_p a p-dimensional vector of ones, $\beta_X = (\beta_{X_1}, \dots, \beta_{X_p})$ and h_X the desired level of heritability, or how much variation G can explain in X, fixed at 10% in all simulated scenarios.

The confounder U is drawn from a multivariate standard Gaussian distribution, i.e., $U \sim \text{N}_N(0, I_N)$ and then split into two equally sized vectors U_X and U_Y with effect θ_X^U impacting all the exposures X and θ_Y^U effecting all the outcomes Y. Their value is fixed at $\theta_X^U = 2$ and $\theta_Y^U = 1$ in main results. Instrument strength depends on the degree of confounding, where the average F-statistic is on average around 45 and 35 for the weakest degrees of confounding $\theta_X^U = \theta_Y^U = 1$ and for the strongest confounding effects $\theta_X^U = \theta_Y^U = 2$, respectively.

The effects β_{X_j} of the genetic variants on the jth exposure, $j = 1, \dots, p$, across n IVs are drawn independently

from a multivariate Gaussian distribution, i.e., $\beta_{X_j} \sim \mathrm{N}_n(0, c_X I_n)$, where c_X is a scaling factor related to the sought range of the simulated β_X and defined as the difference between the maximum and minimum desired value of the effect size divided by four. The range of the simulated β_X is chosen between -2 and 2 with 95% of the simulated values within this interval. It also implies a unit variance for the summary-level genetic associations with the exposures.

We prepare different scenarios in which the exposures are either independent or correlated by inducing a dependence between exposures by multiplying the $(n \times p)$-dimensional matrix β_X with the $(p \times p)$-dimensional matrix D_X, where D_X is the Cholesky decomposition of the Toeplitz matrix R_X with $r_X^{|j-j'|}$ for the (j,j') element of R_X, $j = j' = 1,\ldots,p$. The matrix R_X implies a tridiagonal sparse inverse correlation matrix R_X^{-1}. We use different levels of correlation between the exposures, ranging from independence to strong correlation, i.e., $r_X = \{0, 0.2, 0.4, 0.6, 0.8\}$, where $r_X = 0.6$ represents a medium correlation strength.

For the second stage of the simulation study, according to Equation 5, an outcome k, $k = 1,\ldots,q$, is generated on another independent set of $N_Y = 50,000$ individuals

$$Y_k = X\theta_k + A\theta^A + U\theta_Y^U + \varepsilon_k, \qquad \text{(Equation A14)}$$

where θ_k is p-dimensional vector that contains the direct causal effects of the exposures on the kth outcome, $X Z X$ is the $(N_X \times p)$-dimensional matrix of exposures simulated using Equation A13, θ^A and θ_Y^U are the effects of the unmeasured pleiotropic pathway A and the unmeasured confounder U on the same outcome, respectively, and $\varepsilon_k \sim \mathrm{N}_{N_Y}(0, H_{Y_k} I_{N_Y})$, where $H_{Y_k} = \frac{1-h_Y}{h_Y}\{(X\theta_k + A\theta^A + U\theta_Y^U)^T(X\theta_k + A\theta^A + U\theta_Y^U)\}$ with h_Y the desired level of the proportion of variance explained, fixed at 25% for all outcomes in all simulated scenarios, except for scenario I—null setting. We also simulate different second-level data with various values of the pleiotropic effect on the outcomes $\theta^A = \{0.25, 0.50, 0.75, 1.00, 1.50, 2.00\}$.

In the simulation V—dependence scenario, we induce the correlation between the outcomes by directly controlling the responses' error correlation. Specifically, we simulate the errors $\varepsilon = (\varepsilon_1, \ldots, \varepsilon_q)$ for all k in Equation A14 from a multivariate Gaussian distribution with mean vector 0 and correlation structure based on the Toeplitz matrix R_Y with $r_Y^{|k-k'|}$ for the (k,k') element of R_Y, $k = k' = 1,\ldots,q$ and the level of r_Y chosen in the set $r_Y = \{0, 0.2, 0.4, 0.6, 0.8\}$ such that it induces correlation between the responses' errors

$$\mathrm{vec}(\varepsilon) \sim \mathrm{N}_{N_Y q}(0, H_{N_Y} \otimes R_Y),$$

where $H_{N_Y} = \frac{h_Y}{1-h_Y}\mathrm{diag}\{(X\theta + U\theta_Y^U 1_p^T)(X\theta_k + U\theta_Y^U 1_p^T)^T\}$ is an $N_Y \times N_Y$ diagonal matrix with $\theta = (\theta_1, \ldots, \theta_p)^T$ the vector of the direct effect estimates.

To evaluate the impact in Equation A14 of the unmeasured shared pleiotropy on the outcomes, we generate the N_Y-dimensional vector A as follows,

$$A = G_Y\beta_A + \varepsilon_A, \qquad \text{(Equation A15)}$$

where β_A is the n-dimensional vector of genetic associations with the unmeasured pleiotropic pathway A drawn from a uniform distribution defined on a range between -2 and 2, i.e., $\beta_{A_i} \overset{\text{iid}}{\sim} \mathrm{Unif}(-2, 2)$, $1, \ldots, n$, mimicking shared "undirected" pleiotropy, i.e., $A_l \gtrless 0$, $l = 1,\ldots,N$. Similarly to the simulation of the exposures, the error term in Equation A15 is simulated controlling the level of variance explained $\varepsilon_A \sim \mathrm{N}_{N_Y}(0, H_A)$, where $H_A = \frac{1-h_X}{h_X}\mathrm{diag}\{(G_Y\beta_A)(G_Y\beta_A)^T\}$. Additionally, we consider shared "directed" pleiotropy, where the effect direction on the pleiotropic pathway A is drawn from a uniform distribution defined only on a positive range between 0 and 2, i.e., $\beta_{A_i} \overset{\text{iid}}{\sim} \mathrm{Unif}(0, 2)$. In case of non-overlapping samples in the genetic associations with the outcomes, the simulation strategy for A_k, $k = 1,\ldots,q$, follows the same principles we have described earlier to induce correlation between the exposures.

Finally, the direct causal effects θ_k, $k = 1,\ldots,q$, are drawn independently from a multivariate Gaussian distribution, i.e., $\theta_k \sim \mathrm{N}_p(0, c_Y I_p)$, where c_Y is a scaling factor related to the sought range of the simulated θ_k and defined as the difference between the maximum and minimum desired value of the direct causal effects divided by four. For the direct causal effects, the desired interval lies between -2 and 2, implying unit variance for the direct causal effects. Note that with this choice, 70% of all direct causal effects are simulated between $(-1, 1)$.

In the simulation study, we include one "null" setting with no direct causal effects. All other settings consider a $(q \times p)$-dimensional sparse matrix of direct causal effects $\Theta = (\theta_1, \ldots, \theta_q)^T$, where 30% cells of the matrix are non-zero and where several exposures are either shared or distinct for the outcomes. Specifically, we select at random the same proportion of cells in the matrix Θ and assign them the simulated values, while the other cells are set to zero. On average, the most likely configuration is with a distinct exposure (37.3%) followed by a shared exposure between two outcomes (31.5%), no direct causal effects (16.6%), and an exposure shared by more than two outcomes (14.5%) (see Table S12 for an overview). On the other hand, the most likely number of associated exposures for each outcome is four (24.4%), five (22.5%), three (18%), six (14.5%), two (8.5%), and seven (6.6%).

After creating data on the individual level, we compute the corresponding summary-level statistics from the two independent groups of individuals. The input data for the simulation are the summary-level statistics β_X, an $(n \times p)$-dimensional matrix, and β_Y, a $(n \times q)$-dimensional matrix, derived from a univariable linear regression model where each genetic variant G_i, $i = 1, \ldots, n$, is

regressed against exposure X_j, $j = 1, \ldots, p$, or outcome Y_k, $k = 1, \ldots, q$, at a time. Additionally, we monitored the average F-statistic in the first stage to control for instrument strength. All genetic variants are considered with equal weights, and thus no IVW is needed given the same MAF at 5%.

All simulation runs are repeated 50 times, each of which is initialized with a different random seed.

Data and code availability

MR^2 is freely available on https://github.com/lb664/MR2/. It includes examples that explain how to generate the simulated data and run the algorithm. Post-processing routines are also included.

Supplemental information

Supplemental information can be found online at https://doi.org/10.1016/j.ajhg.2023.06.005.

Acknowledgments

The authors are thankful to Zhi Zhao and Manuela Zucknick for their insightful comments on an early draft of the manuscript. The authors are also grateful to the editor and two anonymous referees for their valuable comments that improved the presentation of the article and for pointing out the problem of non-overlapping samples in the genetic associations with the responses.

The authors gratefully acknowledge the United Kingdom Research and Innovation Medical Research Council grants MR/W029790/1 (V.Z., L.B.) and MC_UU_00002/7 (S.B.), The Alan Turing Institute under the Engineering and Physical Sciences Research Council grant EP/N510129/1 (L.B.), Marmaduke Sheild Fund (L.B.), the British Heart Foundation Centre of Research Excellence at Imperial College London grant RE/18/4/34215 (D.G.), Wellcome Trust award 225790/Z/22/Z (S.B.), the Institute for Translational Medicine and Therapeutics of the Perelman School of Medicine at the University of Pennsylvania (M.G.L.), the NIH/NHLBI National Research Service award postdoctoral fellowship (T32HL007843) (M.G.L.), the Methuselah Foundation (M.G.L.), and US Department of Veterans Affairs award IK2-CX001780 (S.M.D.). This publication does not represent the views of the Department of Veterans Affairs or the United States Government.

Author contributions

Conceptualization: V.Z., L.B., D.G.; methodology: V.Z., L.B.; formal analysis: L.B., V.Z.; resources: all collaborators; data curation: V.Z., D.G.; writing – original draft: V.Z., L.B.; writing – review & editing: all collaborators; visualization: L.B., V.Z.; supervision: L.B.

Declaration of interests

The authors declare no competing interests.

Received: January 30, 2023
Accepted: June 11, 2023
Published: July 6, 2023

References

1. Marengoni, A., Angleman, S., Melis, R., Mangialasche, F., Karp, A., Garmen, A., Meinow, B., and Fratiglioni, L. (2011). Aging with multimorbidity: A systematic review of the literature. Ageing Res. Rev. *10*, 430–439. https://doi.org/10.1016/j.arr.2011.03.003.
2. Pearson-Stuttard, J., Ezzati, M., and Gregg, E.W. (2019). Multimorbidity–a defining challenge for health systems. Lancet Public Health *4*, e599–e600. https://doi.org/10.1016/S2468-2667(19)30222-1.
3. Whitty, C.J.M., and Watt, F.M. (2020). Map clusters of diseases to tackle multimorbidity. Nature *579*, 494–496. https://doi.org/10.1038/d41586-020-00837-4.
4. Skou, S.T., Mair, F.S., Fortin, M., Guthrie, B., Nunes, B.P., Miranda, J.J., Boyd, C.M., Pati, S., Mtenga, S., and Smith, S.M. (2022). Multimorbidity. *Nature Reviews Disease Primers* *8*, 48. https://doi.org/10.1038/s41572-022-00376-4.
5. MacMahon, S.; and The Academy of Medical Sciences (2018). Multimorbidity: A priority for Global Health Research. https://acmedsci.ac.uk/file-download/82222577.
6. World Health Organization (2016). Patient Engagement: Technical Series on Safer Primary Care. https://apps.who.int/iris/bitstream/handle/10665/252269/9789241511629-eng.pdf.
7. Dong, G., Feng, J., Sun, F., Chen, J., and Zhao, X.M. (2021). A global overview of genetically interpretable multimorbidities among common diseases in the UK Biobank. Genome Med. *13*, 110. https://doi.org/10.1186/s13073-021-00927-6.
8. Pietzner, M., Stewart, I.D., Raffler, J., Khaw, K.T., Michelotti, G.A., Kastenmüller, G., Wareham, N.J., and Langenberg, C. (2021). Plasma metabolites to profile pathways in noncommunicable disease multimorbidity. Nat. Med. *27*, 471–479. https://doi.org/10.1038/s41591-021-01266-0.
9. Whitty, C.J.M., MacEwen, C., Goddard, A., Alderson, D., Marshall, M., Calderwood, C., Atherton, F., McBride, M., Atherton, J., Stokes-Lampard, H., et al. (2020). Rising to the challenge of multimorbidity. Br. Med. J. *368*, l6964. https://doi.org/10.1136/bmj.l6964.
10. Didelez, V., and Sheehan, N. (2007). Mendelian randomization as an instrumental variable approach to causal inference. Stat. Methods Med. Res. *16*, 309–330. https://doi.org/10.1177/0962280206077743.
11. Smith, G.D., Ebrahim, S., and Ebrahim, S. (2003). 'M. randomization': Can genetic epidemiology contribute to understanding environmental determinants of disease? Int. J. Epidemiol. *32*, 1–22. https://doi.org/10.1093/ije/dyg070.
12. Davies, N.M., Holmes, M.V., and Davey Smith, G. (2018). Reading Mendelian randomisation studies: A guide, glossary, and checklist for clinicians. Br. Med. J. *362*, k601. https://doi.org/10.1136/bmj.k601.
13. Burgess, S., Foley, C.N., and Zuber, V. (2018). Inferring causal relationships between risk factors and outcomes from genome-wide association study data. Annu. Rev. Genomics Hum. Genet. *19*, 303–327. https://doi.org/10.1146/annurev-genom-083117-021731.
14. Burgess, S., and Thompson, S.G. (2015). Multivariable Mendelian randomization: The use of pleiotropic genetic variants to estimate causal effects. Am. J. Epidemiol. *181*, 251–260. https://doi.org/10.1093/aje/kwu283.
15. Zuber, V., Colijn, J.M., Klaver, C., and Burgess, S. (2020). Selecting likely causal risk factors from high-throughput experiments

using multivariable Mendelian randomization. Nat. Commun. *11*, 29. https://doi.org/10.1038/s41467-019-13870-3.

16. Allara, E., Morani, G., Carter, P., Gkatzionis, A., Zuber, V., Foley, C.N., Rees, J.M.B., Mason, A.M., Bell, S., Gill, D., et al. (2019). Genetic determinants of lipids and cardiovascular disease outcomes: A wide-angled Mendelian randomization investigation. Circ. Genom. Precis. Med. *12*, 543–551. https://doi.org/10.1161/CIRCGEN.119.002711.
17. Levin, M.G., Zuber, V., Walker, V.M., Klarin, D., Lynch, J., Malik, R., Aday, A.W., Bottolo, L., Pradhan, A.D., Dichgans, M., et al. (2021). Prioritizing the role of major lipoproteins and subfractions as risk factors for peripheral artery disease. Circulation *144*, 353–364. https://doi.org/10.1161/circulationaha.121.053797.
18. Fox, K.F., Cowie, M.R., Wood, D.A., Coats, A.J., Gibbs, J.S., Underwood, S.R., Turner, R.M., Poole-Wilson, P.A., Davies, S.W., and Sutton, G.C. (2001). Coronary artery disease as the cause of incident heart failure in the population. Eur. Heart J. *22*, 228–236. https://doi.org/10.1053/euhj.2000.2289.
19. Gheorghiade, M., Sopko, G., De Luca, L., Velazquez, E.J., Parker, J.D., Binkley, P.F., Sadowski, Z., Golba, K.S., Prior, D.L., Rouleau, J.L., and Bonow, R.O. (2006). Navigating the crossroads of coronary artery disease and heart failure. Circulation *114*, 1202–1213. https://doi.org/10.1161/CIRCULATIONAHA.106.623199.
20. Benjamin, E.J., Levy, D., Vaziri, S.M., D'Agostino, R.B., Belanger, A.J., and Wolf, P.A. (1994). Independent risk factors for atrial fibrillation in a population-based cohort. The Framingham Heart Study. JAMA *271*, 840–844. https://doi.org/10.1001/jama.1994.03510350050036.
21. Stewart, S., Hart, C.L., Hole, D.J., and McMurray, J.J.V. (2002). A population-based study of the long-term risks associated with atrial fibrillation: 20-year follow-up of the Renfrew/Paisley study. Am. J. Med. *113*, 359–364. https://doi.org/10.1016/s0002-9343(02)01236-6.
22. Rahimi, K., Lam, C.S.P., and Steinhubl, S. (2018). Cardiovascular disease and multimorbidity: A call for interdisciplinary research and personalized cardiovascular care. PLoS Med. *15*, 1–3. https://doi.org/10.1371/journal.pmed.1002545.
23. Xu, X., Mishra, G.D., Dobson, A.J., and Jones, M. (2018). Progression of diabetes, heart disease, and stroke multimorbidity in middle-aged women: A 20-year cohort study. PLoS Med. *15*, e1002516–e1002518. https://doi.org/10.1371/journal.pmed.1002516.
24. Wilson, P.W. (1994). Established risk factors and coronary artery disease: The framingham study. Am. J. Hypertens. *7*, 7S–12S. https://doi.org/10.1093/ajh/7.7.7S.
25. Fowkes, F.G.R., Rudan, D., Rudan, I., Aboyans, V., Denenberg, J.O., McDermott, M.M., Norman, P.E., Sampson, U.K.A., Williams, L.J., Mensah, G.A., and Criqui, M.H. (2013). Comparison of global estimates of prevalence and risk factors for peripheral artery disease in 2000 and 2010: A systematic review and analysis. Lancet *382*, 1329–1340. https://doi.org/10.1016/S0140-6736(13)61249-0.
26. Khatibzadeh, S., Farzadfar, F., Oliver, J., Ezzati, M., and Moran, A. (2013). Worldwide risk factors for heart failure: A systematic review and pooled analysis. Int. J. Cardiol. *168*, 1186–1194. https://doi.org/10.1016/j.ijcard.2012.11.065.
27. Shaper, A.G., Phillips, A.N., Pocock, S.J., Walker, M., and Macfarlane, P.W. (1991). Risk factors for stroke in middle aged British men. Br. Med. J. *302*, 1111–1115. https://doi.org/10.1136/bmj.302.6785.1111.
28. Rees, J.M.B., Wood, A.M., and Burgess, S. (2017). Extending the MR-Egger method for multivariable Mendelian randomization to correct for both measured and unmeasured pleiotropy. Stat. Med. *36*, 4705–4718. https://doi.org/10.1002/sim.7492.
29. Burgess, S., Davies, N.M., and Thompson, S.G. (2016). Bias due to participant overlap in two-sample Mendelian randomization. Genet. Epidemiol. *40*, 597–608. https://doi.org/10.1002/gepi.21998.
30. LeBlanc, M., Zuber, V., Thompson, W.K., Andreassen, O.A., Schizophrenia and Bipolar Disorder Working Groups of the Psychiatric Genomics Consortium, Frigessi, A., and Andreassen, B.K. (2018). A correction for sample overlap in genome-wide association studies in a polygenic pleiotropy-informed framework. BMC Genom. *19*, 494. https://doi.org/10.1186/s12864-018-4859-7.
31. Sanderson, E., Davey Smith, G., Windmeijer, F., and Bowden, J. (2019). An examination of multivariable Mendelian randomization in the single-sample and two-sample summary data settings. Int. J. Epidemiol. *48*, 713–727. https://doi.org/10.1093/ije/dyy262.
32. Burgess, S., Dudbridge, F., and Thompson, S.G. (2016). Combining information on multiple instrumental variables in Mendelian randomization: Comparison of allele score and summarized data methods. Stat. Med. *35*, 1880–1906. https://doi.org/10.1002/sim.6835.
33. Thompson, S.G., and Sharp, S.J. (1999). Explaining heterogeneity in meta-analysis: A comparison of methods. Stat. Med. *18*, 2693–2708. https://doi.org/10.1002/(SICI)1097-0258(19991030)18:20¸2693::AID-SIM235¿3.0.CO;2-V.
34. Mawdsley, D., Higgins, J.P.T., Sutton, A.J., and Abrams, K.R. (2017). Accounting for heterogeneity in meta-analysis using a multiplicative model–an empirical study. Res. Synth. Methods *8*, 43–52. https://doi.org/10.1002/jrsm.1216.
35. Bowden, J., Davey Smith, G., and Burgess, S. (2015). Mendelian randomization with invalid instruments: Effect estimation and bias detection through Egger regression. Int. J. Epidemiol. *44*, 512–525. https://doi.org/10.1093/ije/dyv080.
36. Zuber, V., Gill, D., Ala-Korpela, M., Langenberg, C., Butterworth, A., Bottolo, L., and Burgess, S. (2021). High-throughput multivariable Mendelian randomization analysis prioritizes apolipoprotein B as key lipid risk factor for coronary artery disease. Int. J. Epidemiol. *50*, 893–901. https://doi.org/10.1093/ije/dyaa216.
37. Burgess, S., Daniel, R.M., Butterworth, A.S., Thompson, S.G.; and EPIC-InterAct Consortium (2015). Network Mendelian randomization: Using genetic variants as instrumental variables to investigate mediation in causal pathways. Int. J. Epidemiol. *44*, 484–495. https://doi.org/10.1093/ije/dyu176.
38. Zellner, A. (1962). An efficient method of estimating seemingly unrelated regressions and tests for aggregation bias. J. Am. Stat. Assoc. *57*, 348–368. https://doi.org/10.1080/01621459.1962.10480664.
39. World Health Organization. (2022). Social Determinants of Health. https://www.who.int/health-topics/social-determinants-of-health.
40. Alexopoulos, A., and Bottolo, L. (2020). Bayesian Variable Selection for Gaussian copula regression models. J. Comput. Graph Stat. *30*, 578–593. https://doi.org/10.1080/10618600.2020.1840997.
41. Smith, M.S. (2013). Bayesian approaches to copula modelling. In Bayesian Theory and Applications, P. Damien, P.

Dellaportas, N.G. Polson, and D.A. Stephens, eds. (Oxford University Press), pp. 336–358. https://doi.org/10.1093/acprof:oso/9780199695607.003.0017.
42. Lauritzen, S.L. (2004). Graphical Models (Clarendon Press).
43. George, E.I., and McCulloch, R.E. (1997). Approaches for Bayesian variable selection. Stat. Sin. *7*, 339–373. http://www.jstor.org/stable/24306083.
44. Held, L., and Holmes, C.C. (2006). Bayesian auxiliary variable models for binary and multinomial regression. Bayesian Anal. *1*, 145–168. https://doi.org/10.1214/06-BA105.
45. Wang, H. (2010). Sparse seemingly unrelated regression modelling: Applications in finance and econometrics. Comput. Stat. Data Anal. *54*, 2866–2877. https://doi.org/10.1016/j.csda.2010.03.028.
46. Rothman, A.J., Levina, E., and Zhu, J. (2010). Sparse multivariate regression with covariance estimation. J. Comput. Graph Stat. *19*, 947–962. https://doi.org/10.1198/jcgs.2010.09188.
47. Deshpande, S.K., Ročková, V., and George, E.I. (2019). Simultaneous variable and covariance selection with the multivariate spike-and-slab lasso. J. Comput. Graph Stat. *28*, 921–931. https://doi.org/10.1080/10618600.2019.1593179.
48. Barbieri, M.M., and Berger, J.O. (2004). Optimal predictive model selection. Ann. Statist. *32*, 870–897. https://doi.org/10.1214/009053604000000238.
49. Broët, P., Lewin, A., Richardson, S., Dalmasso, C., and Magdelenat, H. (2004). A mixture model-based strategy for selecting sets of genes in multiclass response microarray experiments. Bioinformatics *20*, 2562–2571. https://doi.org/10.1093/bioinformatics/bth285.
50. Müller, P., Parmigiani, G., and Rice, K. (2007). FDR and Bayesian multiple comparison rules. In Bayesian Statistics, *8*, J.M. Bernardo, et al., eds. *(Oxford University Press), pp. 349–370*.
51. Bowden, J., Hemani, G., and Davey Smith, G. (2018). Invited commentary: Detecting individual and global horizontal pleiotropy in Mendelian randomization-A job for the humble heterogeneity statistic? Am. J. Epidemiol. *187*, 2681–2685. https://doi.org/10.1093/aje/kwy185.
52. Geisser, S., and Eddy, W.F. (1979). A predictive approach to model selection. J. Am. Stat. Assoc. *74*, 153–160. https://doi.org/10.1080/01621459.1979.10481632.
53. Benjamini, Y., and Hochberg, Y. (1995). Controlling the false discovery rate: a practical and powerful approach to multiple testing. J. Roy. Stat. Soc. B *57*, 289–300. https://doi.org/10.1111/j.2517-6161.1995.tb02031.x.
54. Ference, B.A., Kastelein, J.J.P., Ray, K.K., Ginsberg, H.N., Chapman, M.J., Packard, C.J., Laufs, U., Oliver-Williams, C., Wood, A.M., Butterworth, A.S., et al. (2019). Association of triglyceride-lowering LPL variants and LDL-C–lowering LDLR variants with risk of coronary heart disease. JAMA *321*, 364–373. https://doi.org/10.1001/jama.2018.20045.
55. Sniderman, A.D., Thanassoulis, G., Glavinovic, T., Navar, A.M., Pencina, M., Catapano, A., and Ference, B.A. (2019). Apolipoprotein B particles and cardiovascular disease. JAMA Cardiol. *4*, 1287–1295. https://doi.org/10.1001/jamacardio.2019.3780.
56. Richardson, T.G., Sanderson, E., Palmer, T.M., Ala-Korpela, M., Ference, B.A., Davey Smith, G., and Holmes, M.V. (2020). Evaluating the relationship between circulating lipoprotein lipids and apolipoproteins with risk of coronary heart disease: A multivariable Mendelian randomisation analysis. PLoS Med. *17*, e1003062. https://doi.org/10.1371/journal.pmed.1003062.
57. Berger, R.L. (1997). Likelihood ratio tests and intersection-union tests. In Advances in Statistical Decision Theory and Applications, S. Panchapakesan and N. Balakrishnan, eds. (Birkhäuser), pp. 225–237. chap. 15. https://doi.org/10.1007/978-1-4612-2308-5_15.
58. Kettunen, J., Demirkan, A., Würtz, P., Draisma, H.H.M., Haller, T., Rawal, R., Vaarhorst, A., Kangas, A.J., Lyytikäinen, L.P., Pirinen, M., et al. (2016). Genome-wide study for circulating metabolites identifies 62 loci and reveals novel systemic effects of LPA. Nat. Commun. *7*, 11122. https://doi.org/10.1038/ncomms11122.
59. Jiang, T., Gill, D., Butterworth, A.S., and Burgess, S. (2022). An empirical investigation into the impact of winner's curse on estimates from Mendelian randomization. Int. J. Epidemiol., dyac233. https://doi.org/10.1093/ije/dyac233. dyac233.
60. Verbanck, M., Chen, C.Y., Neale, B., and Do, R. (2018). Detection of widespread horizontal pleiotropy in causal relationships inferred from Mendelian randomization between complex traits and diseases. Nat. Genet. *50*, 693–698. https://doi.org/10.1038/s41588-018-0099-7.
61. Vaduganathan, M., Mensah, G.A., Turco, J.V., Fuster, V., and Roth, G.A. (2022). The global burden of cardiovascular diseases and risk: A compass for future health. J. Am. Coll. Cardiol. *80*, 2361–2371. https://doi.org/10.1016/j.jacc.2022.11.005.
62. Nelson, C.P., Goel, A., Butterworth, A.S., Kanoni, S., Webb, T.R., Marouli, E., Zeng, L., Ntalla, I., Lai, F.Y., Hopewell, J.C., et al. (2017). Association analyses based on false discovery rate implicate new loci for coronary artery disease. Nat. Genet. *49*, 1385–1391. https://doi.org/10.1038/ng.3913.
63. Klarin, D., Lynch, J., Aragam, K., Chaffin, M., Assimes, T.L., Huang, J., Lee, K.M., Shao, Q., Huffman, J.E., Natarajan, P., et al. (2019). Genome-wide association study of peripheral artery disease in the Million Veteran Program. Nat. Med. *25*, 1274–1279. https://doi.org/10.1038/s41591-019-0492-5.
64. Pierce, B.L., and Burgess, S. (2013). Efficient design for mendelian randomization studies: Subsample and 2-sample instrumental variable estimators. Am. J. Epidemiol. *178*, 1177–1184. https://doi.org/10.1093/aje/kwt084.
65. Sanderson, E., Spiller, W., and Bowden, J. (2021). Testing and correcting for weak and pleiotropic instruments in two-sample multivariable Mendelian randomization. Stat. Med. *40*, 5434–5452. https://doi.org/10.1002/sim.9133.
66. Davey Smith, G., Davies, N.M., Dimou, N., Egger, M., Gallo, V., Golub, R., Higgins, J.P.T., Langenberg, C., Loder, E.W., Richards, J.B., et al. (2019). STROBE-MR: Guidelines for strengthening the reporting of Mendelian randomization studies. PeerJ Preprints *7*, e27857v1. https://doi.org/10.7287/peerj.preprints.27857v1.
67. Burgess, S., Davey Smith, G., Davies, N.M., Dudbridge, F., Gill, D., Glymour, M.M., Hartwig, F.P., Holmes, M.V., Minelli, C., Relton, C.L., and Theodoratou, E. (2019). Guidelines for performing Mendelian randomization investigations. Wellcome Open Res. *4*, 186. https://doi.org/10.12688/wellcomeopenres.15555.2.
68. Bos, M.M., Goulding, N.J., Lee, M.A., Hofman, A., Bot, M., Pool, R., Vijfhuizen, L.S., Zhang, X., Li, C., Mustafa, R., et al. (2021). Investigating the relationships between unfavourable habitual sleep and metabolomic traits: evidence from multicohort multivariable regression and Mendelian randomization analyses. BMC Med. *19*, 69. https://doi.org/10.1186/s12916-021-01939-0.

69. Rajasundaram, S., Rahman, R.P., Woolf, B., Zhao, S.S., and Gill, D. (2022). Morning cortisol and circulating inflammatory cytokine levels: A Mendelian randomisation study. Genes *13*, 116. https://doi.org/10.3390/genes13010116.

70. Elliott, L.T., Sharp, K., Alfaro-Almagro, F., Shi, S., Miller, K.L., Douaud, G., Marchini, J., and Smith, S.M. (2018). Genome-wide association studies of brain imaging phenotypes in UK Biobank. Nature *562*, 210–216. https://doi.org/10.1038/s41586-018-0571-7.

71. Meyer, H.V., Dawes, T.J.W., Serrani, M., Bai, W., Tokarczuk, P., Cai, J., de Marvao, A., Henry, A., Lumbers, R.T., Gierten, J., et al. (2020). Genetic and functional insights into the fractal structure of the heart. Nature *584*, 589–594. https://doi.org/10.1038/s41586-020-2635-8.

72. Lotta, L.A., Wittemans, L.B.L., Zuber, V., Stewart, I.D., Sharp, S.J., Luan, J., Day, F.R., Li, C., Bowker, N., Cai, L., et al. (2018). Association of genetic variants related to gluteofemoral vs abdominal fat distribution with type 2 diabetes, coronary disease, and cardiovascular risk factors. Journal of the American Medical Association *320*, 2553–2563. https://doi.org/10.1001/jama.2018.19329.

73. Bowden, J., Del Greco M, F., Minelli, C., Zhao, Q., Lawlor, D.A., Sheehan, N.A., Thompson, J., and Davey Smith, G. (2019). Improving the accuracy of two-sample summary-data Mendelian randomization: Moving beyond the NOME assumption. Int. J. Epidemiol. *48*, 728–742. https://doi.org/10.1093/ije/dyy258.

74. Giambartolomei, C., Vukcevic, D., Schadt, E.E., Franke, L., Hingorani, A.D., Wallace, C., and Plagnol, V. (2014). Bayesian test for colocalisation between pairs of genetic association studies using summary statistics. PLoS Genet. *10*, e1004383. https://doi.org/10.1371/journal.pgen.1004383.

75. Talhouk, A., Doucet, A., and Murphy, K. (2012). Efficient Bayesian inference for multivariate Probit models with sparse inverse correlation matrices. J. Comput. Graph Stat. *21*, 739–757. https://doi.org/10.1080/10618600.2012.679239.

76. Gelfand, A.E. (1996). Model determination using sampling-based methods. In Markov Chain Monte Carlo in Practice, W. Gilks, S. Richardson, and D. Spiegelhalter, eds. (Chapman & Hall), pp. 145–161.

77. Carlin, B.P., and Louis, T.A. (1996). Bayes and Empirical Bayes Methods for Data Analysis (Chapman and Hall).

78. Ntzoufras, I. (2009). Bayesian Modeling Using WinBUGS (Wiley). https://doi.org/10.1002/9780470434567.

79. Congdon, P. (2005). Bayesian Models for Categorical Data (John Wiley & Sons).

80. Dempster, A.P., Laird, N.M., and Rubin, D.B. (1977). Maximum likelihood from incomplete data via the EM algorithm. J. Roy. Stat. Soc. B *39*, 1–22. https://www.jstor.org/stable/2984875.

The American Journal of Human Genetics, Volume *110*

Supplemental information

Multi-response Mendelian randomization: Identification of shared and distinct exposures for multimorbidity and multiple related disease outcomes

Verena Zuber, Alex Lewin, Michael G. Levin, Alexander Haglund, Soumaya Ben-Aicha, Costanza Emanueli, Scott Damrauer, Stephen Burgess, Dipender Gill, and Leonardo Bottolo

S1 Multi-response MR residual correlation

In eq. (6) of the main paper, $\tilde{\varepsilon}_k$, $k = 1, \ldots, q$, is normally distributed with mean $\mathbb{E}(\tilde{\varepsilon}_k) = 0$ and variance

$$\begin{aligned} \mathbb{V}(\tilde{\varepsilon}_k) &= (G^T G)^{-1} G^T V(\varepsilon_k) G (G G^T)^{-1} \\ &= (G^T G)^{-1} G^T \delta_k^2 I_N G (G^T G)^{-1} \\ &= \delta_k^2 (G^T G)^{-1}. \end{aligned} \tag{S1}$$

In eq. (7), the summary-level error ϵ_k, $k = 1, \ldots, q$, is normally distributed with mean

$$\begin{aligned} \mathbb{E}(\beta_A \theta^A + \tilde{\varepsilon}_k) &= \mathbb{E}(\beta_A \theta^A) + \mathbb{E}(\tilde{\varepsilon}_k) \\ &= \theta^A (G^T G)^{-1} G^T \, \mathbb{E}(A) + (G^T G)^{-1} G^T \, \mathbb{E}(\varepsilon_k) \\ &= \theta^A \mu_A (G^T G)^{-1} G^T 1_N, \end{aligned}$$

where $\mathbb{E}(A) = \mu_A 1_N$ with 1_N a N-dimensional vector of ones.
Assuming A and ε_k independent in eq. (5) and using eq. (S1), then

$$\begin{aligned} \mathbb{V}(\beta_A \theta^A + \tilde{\varepsilon}_k) &= \mathbb{V}(\beta_A \theta^A) + \mathbb{V}(\tilde{\varepsilon}_k) \\ &= (\theta^A)^2 \, \mathbb{V}(\beta_A) + \delta_k^2 (G^T G)^{-1} \\ &= (\theta^A)^2 \sigma_A^2 (G^T G)^{-1} + \delta_k^2 (G^T G)^{-1} \\ &= \{(\theta^A)^2 \sigma_A^2 + \delta_k^2\} (G^T G)^{-1} \end{aligned} \tag{S2}$$

with $\mathbb{V}(\beta_A) = (G^T G)^{-1} G^T \sigma_A^2 I_N G (G^T G)^{-1} = \sigma_A^2 (G^T G)^{-1}$.
Assuming that the quantitative outcomes Y_k, $k = 1, .., q$, are measured on the same N individuals, the correlation between the residuals of the summary-level outcomes k and k', $k \neq k'$, is

$$\begin{aligned} \rho(\epsilon_k, \epsilon_{k'}) &= \mathbb{V}(\beta_A \theta^A + \tilde{\varepsilon}_k)^{-1/2} \, \mathrm{Cov}(\beta_A \theta^A + \tilde{\varepsilon}_k, \beta_A \theta^A + \tilde{\varepsilon}_{k'}) \times \\ &\quad \mathbb{V}(\beta_A \theta^A + \tilde{\varepsilon}_{k'})^{-1/2} \\ &= \{(\theta^A)^2 \sigma_A^2 + \delta_k^2\}^{-1/2} (G^T G)^{1/2} \times \\ &\quad \{\mathbb{V}(\beta_A \theta^A) + \mathrm{Cov}(\tilde{\varepsilon}_k, \tilde{\varepsilon}_{k'})\} \times \\ &\quad \{(\theta^A)^2 \sigma_A^2 + \delta_{k'}^2\}^{-1/2} (G^T G)^{1/2} \\ &= \{(\theta^A)^2 \sigma_A^2 + \delta_k^2\}^{-1/2} (G^T G)^{1/2} \times \\ &\quad \{(\theta^A)^2 \sigma_A^2 (G^T G)^{-1} + \sigma_{kk'} (G^T G)^{-1}\} \times \\ &\quad \{(\theta^A)^2 \sigma_A^2 + \delta_{k'}^2\}^{-1/2} (G^T G)^{1/2} \\ &= \frac{(\theta^A)^2 \sigma_A^2 + \sigma_{kk'}}{\{(\theta^A)^2 \sigma_A^2 + \delta_k^2\}^{1/2} \{(\theta^A)^2 \sigma_A^2 + \delta_{k'}^2\}^{1/2}} I_n \end{aligned} \tag{S3}$$

using (S2) and the fact that

$$\begin{aligned}\mathrm{Cov}(\tilde{\varepsilon}_k, \tilde{\varepsilon}_{k'}) &= (G^T G)^{-1} G^T \sigma_{kk'} I_N G (GG^T)^{-1} \\ &= \sigma_{kk'} (G^T G)^{-1} G^T G (GG^T)^{-1} \\ &= \sigma_{kk'} (G^T G)^{-1},\end{aligned}$$

where $\sigma_{kk'} = \mathrm{Cov}(\varepsilon_k, \varepsilon_{k'})$ is the covariance between individual-level responses' errors which is assumed constant across all individuals.

Considering all outcomes and all IVs, the summary-level covariance matrix can be written in compact form as $\rho(\epsilon) = I_n \otimes R$ with $\epsilon = (\epsilon_1, \ldots, \epsilon_q)$ and $R_{kk'}$ as in eq. (S3) for any of the n IVs.

In case of non-overlapping samples in the genetic associations with the outcomes, we assume that responses k and k', $k \neq k'$, are measured in two distinct groups of individuals k and k', respectively, and no individuals are present in both groups. Let G_{Y_k} be the $(n \times N_{Y_k})$-dimensional matrix of genotypes for the group of individuals k for outcome k and, similarly, $G_{Y_{k'}}$ the $(n \times N_{Y_{k'}})$-dimensional matrix of genotypes for group k' for outcome k'. Without loss of generality, let $N_Y = N_{Y_k} = N_{Y_{k'}}$. Then,

$$\begin{aligned}\mathrm{Cov}(\beta_{A_k}\theta^A + \tilde{\varepsilon}_k, \beta_{A_{k'}}\theta^A + \tilde{\varepsilon}_{k'}) &= (\theta^A)^2 \,\mathrm{Cov}(\beta_{A_k}, \beta_{A_{k'}}) + \mathrm{Cov}(\tilde{\varepsilon}_k, \tilde{\varepsilon}_{k'}) \\ &= \{(\theta^A)^2 G_{Y_k}^T \,\mathrm{Cov}(A_k, A_{k'}) G_{Y_{k'}} + \qquad \text{(S4)} \\ &\quad \sigma_{kk'} G_{Y_k}^T G_{Y_{k'}}\} (G_{Y_k}^T G_{Y_k})^{-1} (G_{Y_{k'}}^T G_{Y_{k'}})^{-1}.\end{aligned}$$

Moreover, $\mathbb{V}(\beta_{A_k}\theta^A + \tilde{\varepsilon}_k) = \{(\theta^A)^2 \sigma_{A_k}^2 + \delta_k^2 I_n\}(G_{Y_k}^T G_{Y_k})^{-1}$ and similarly for $\mathbb{V}(\beta_{A_{k'}}\theta^A + \tilde{\varepsilon}_{k'})$. Trivially, in eq. (S4), $\sigma_{kk'} = 0$ for non-overlapping samples and it is non-zero if $\mathrm{Cov}(A_k, A_{k'}) \neq 0$ for all $\ell_k = \ell_{k'} = 1, \ldots, N_Y$. It is sufficient to assume that

$$((\beta_{A_k})_i, (\beta_{A_{k'}})_i)^T \overset{\text{iid}}{\sim} \mathrm{N}_2(0, cR^A), \quad R^A = \begin{bmatrix} 1 & \rho^A \\ \rho^A & 1 \end{bmatrix} \qquad \text{(S5)}$$

to impose that $\mathrm{Cov}(A_k, A_{k'}) \neq 0$. Note that if $G_{Y_k} = G_{Y_{k'}}$, $\rho^A = 1$ and $c = \sigma_A^2$, results coincide with eq. (S3).

S2 Details of the MCMC implementation

The MCMC algorithm for the selection of important predictors in regression models with multiple responses of any type is presented in [1]. Here, we describe the main steps of the MCMC algorithm for the proposed multi-response multivariable Mendelian randomization MR^2 model as well as the main advantages of the designed proposal distribution for the non-zero direct

causal effects θ_k^* over standard implementations of Bayesian inference for the SUR model. In the following, the symbol "*" indicates the proposed new value used in the Metropolis-Hastings (M-H) acceptance ratio.

Given a candidate value of the latent binary vector γ_k^*, the proposal density for θ_k^* is

$$q(\theta_k^*|\gamma_k^*, \beta_{Y_k}, Z_{_k}, \Delta, R) \propto \mathcal{N}_{|\gamma_k^*|}(\theta_k^*; m_{\gamma_k^*}, V_{\gamma_k^*}), \tag{S6}$$

where

- $V_{\gamma_k^*}^{-1} = \delta_{k|-k}^{-2}(\beta_X^T)_{\gamma_k^*}(\beta_X)_{\gamma_k^*} + v^{-1}I_{|\gamma_k^*|}$ is the $(|\gamma_k^*| \times |\gamma_k^*|)$-dimensional proposal precision matrix,
- $m_{\gamma_k^*} = V_{\gamma_k^*}(\beta_X^T)_{\gamma_k^*}(\beta_{Y_k} - \delta_k\mu_{k|-k})/\delta_{k|-k}^2$ is the $|\gamma_k^*|$-dimensional proposal mean,
- $\delta_{k|-k}^2$ is the conditional variance of $\beta_{Y_k}|Z_{_{-k}}$, *i.e.*, $\delta_{k|-k}^2 = \delta_k^2/(R^{-1})_{kk}$,
- $\mu_{k|-k}$ is the n-dimensional vector of conditional means of $Z_k|Z_{_{-k}}$, *i.e.*, $\mu_{k|-k} = Z_{-k}R_{-k,-k}^{-1}R_{-k,k}$

and where the subscript "$-k$" implies that the corresponding matrix (or vector) consists of all the elements except those related to the kth response.

The interpretation of the above quantities sheds light on the benefits of the proposed model. $V_{\gamma_k^*}^{-1}$ resembles the standard Gibbs sampling update of the regression coefficients precision matrix in the linear model. However, in $V_{\gamma_k^*}^{-1}$, we use the conditional variance $\delta_{k|-k}^2$ of $\beta_{Y_k}|Z_{_{-k}}$ instead of the marginal variance δ_k^2. In $m_{\gamma_k^*}$, since $\mathbb{E}(\beta_{Y_k}) = (\beta_X)_{\gamma_k^*}(\theta_k)_{\gamma_k^*} + \delta_k Z_{-k}R_{-k,-k}^{-1}R_{-k,k} = (\beta_X)_{\gamma_k^*}(\theta_k)_{\gamma_k^*} + \delta_k\mu_{k|-k}$, then $\mathbb{E}(\beta_{Y_k} - \delta_k\mu_{k|-k}) = (\beta_X)_{\gamma_k^*}(\theta_k)_{\gamma_k^*}$. Thus, we recenter the genetic associations with the kth outcome such that, once conditioned on γ_k^*, they depend only on $(\beta_X)_{\gamma_k^*}(\theta_k)_{\gamma_k^*}$ as in eq. (12). The other terms in $m_{\gamma_k^*}$ mirror the mean of the full conditional for the regression coefficients of the linear model. In summary, the proposal density $q(\theta_k^*|\gamma_k^*, \beta_{Y_k}, Z_{_k}, \Delta, R)$ is inspired by the Gibbs updates of the regression coefficients in the linear model with suitable modifications to account for the conditional dependence between the kth outcome and the remaining responses at the summary level.

The designed proposal distribution allows also the "implicit marginalisation" of the direct causal effects when the joint update of (θ_k, γ_k), $k = 1, \ldots, q$, in the M-H step is performed. For the kth response, the acceptance probability α of the joint proposal is

$$\alpha = 1 \wedge \frac{\left|V_{\gamma_k^*}\right|^{1/2} v^{|\gamma_k|/2} \exp\left(\frac{1}{2}m_{\gamma_k^*}^T V_{\gamma_k^*}^{-1} m_{\gamma_k^*}\right) p(\gamma_k^*)q(\gamma_k|\gamma_k^*)}{\left|V_{\gamma_k}\right|^{1/2} v^{|\gamma_k^*|/2} \exp\left(\frac{1}{2}m_{\gamma_k}^T V_{\gamma_k}^{-1} m_{\gamma_k}\right) p(\gamma_k)q(\gamma_k^*|\gamma_k)}, \tag{S7}$$

where $p(\gamma_k)$ is defined in eq. (15) and $q(\gamma_k^*|\gamma_k)$ is the proposal distribution for the latent binary vector γ_k^* conditional to the current value γ_k. Similarly to [2], in eq. (S7), the current and proposed value of the direct causal effects (θ_k, θ_k^*) do not appear and the acceptance probability resembles the Bayes Factor of the standard linear regression model used in the update of the latent binary vector γ_k.

The advantages of the "implicit marginalisation" of the regression coefficients in eq. (S7) for the SUR model have not been recognised until recently [1]. This is a key point that distinguishes our prosed methodology from other SUR models [3, 4]. Similarly to MR^2, a sparse Bayesian SUR model [5] proposes two computational algorithms, an MCMC algorithm using Gibbs updates [6] for variable selection called "undirected" and a "directed" algorithm involving a Monte Carlo numerical approximation of the marginal likelihood to integrate out the regression coefficients combined with an M-H algorithm similar to eq. (S7) for posterior models' exploration. However, Monte Carlo numerical approximation is unstable and very time-consuming, making [5] unfeasible even for small data sets.

We conclude with the description regarding the MCMC steps required to obtain samples from $p(\Sigma|W, \mathrm{G})$ and transform them using the inverse transformation $R = \Delta^{-1}\Sigma\Delta^{-1}$, where Δ is sampled from its prior distribution in eq. (18). To sample the symmetric adjacency matrix G from $p(\mathrm{G}|\beta_Y, Z, \Delta, R)$, we target the distribution with density

$$p(\mathrm{G}|W) = \frac{p(W|\mathrm{G})p(\mathrm{G})}{\sum_{\mathrm{G}} p(W|\mathrm{G})p(\mathrm{G})}, \tag{S8}$$

where the summation in the denominator is over all the decomposable graphs G, $p(\mathrm{G})$ is defined in (17), W is defined in Appendix and $p(W|\mathrm{G})$ can be computed analytically due to the tractability of the hyper-inverse Wishart distribution [7]. Specifically, we sample from eq. (S8) by using an M-H step in which, conditionally on the current symmetric adjacency matrix G, a new graph is proposed by adding or deleting an edge between two vertices whose index has been chosen randomly between the vertices that belong to a decomposable graph. The proposed graph is then accepted or rejected using the accept/reject mechanism of the M-H step which targets the density in eq. (S8). Finally, conditionally on W and the updated symmetric adjacency matrix G, we sample Σ from its conditional hyper-inverse Wishart distribution, *i.e.*, $\mathrm{HIW}_{\mathcal{G}}(2 + n, I_q + W^T W)$.

S3 Simulation study

S3.1 Comparison of methods setup

MR^2 parameters setting

The MR^2 algorithm is run for $7,500$ MCMC iterations of which $2,500$ as burn-in and results saved every 5 iterations, resulting in $1,000$ posterior samples for all the unknowns. The hyper-parameters a_k and b_k in eq. (15) are chosen by specifying $\mathbb{E}(\gamma_k) = 2$ and $\mathbb{V}(\gamma_k) = 2$ which implies *a priori* a range between zero and seven for the number of significant direct causal effects for each outcome, see [1] for details. Another hyper-parameter to be specified is the prior variance v in eq. (13). We set $v = 1$ since the summary-level genetic associations with the risk factors in eq. (21) have been simulated with unit variance and no IVW is required given the same MAF for all simulated genotypes. To select a suitable number of MCMC iterations, and in particular the length of the burn-in phase, we ran some preliminary analyses and checked by visual inspection the trace plots of several posterior quantities including the number of exposures selected during the MCMC for all responses, the number of non-zero cells in the sparse matrix of residual correlation $R|Y$ and the square root of the error variance $\delta_k|Y$ for each response k, $k = 1, \dots, 5$. No signs of slow convergence or aberrant behaviour of the designed MCMC sampler are identified in a variety of scenarios and parameter settings, suggesting that the number of burn-in iterations are sufficient to reach convergence and sample from the equilibrium distribution of the Markov chain.

In case of different MAF across genotypes, we recommend setting $v = 1$ and standardise the summary-level genetic associations with the risk factors after IVW in order to place appropriate prior mass on reasonable values of the non-zero direct causal effects [8].

Alternative methods and parameters setting

As alternative methods, we include standard multivariable MR (MV-MR) which considers multiple exposures in one joint model but does not perform variable selection [9]. Next, we consider MR-BMA [10], a Bayesian variable selection approach for multivariable MR, with a prior probability of inclusion set at $2/15 = 0.13$ reflecting two associated causal exposures for each outcome. In this way, we match the prior specification of the expected number of risk factors associated with the kth response used in MR^2. Both MV-MR and MR-BMA only consider one outcome at-a-time and are performed on each outcome separately.

Additionally, we include two multivariate and multivariable variable selection approaches which have to date not been applied to MR, the multiple responses Lasso (Multivariate Regression With Covariance Estimation, MRCE) [3] and the multiple responses Spike and Slab Lasso (mSSL) [4].

Regarding the MRCE algorithm, we select the cross-validation procedure option for the sparse Lasso solution which performs k-fold cross-validation using candidate tuning parameters specified in the vectors λ_1 and λ_2, the two penalisation parameters for the sparse regression and sparse covariance estimation, respectively. We run the algorithm with the default recommended values including 5-fold cross-validation and 40 equally spaced candidate tuning parameters λ_1 and λ_2 between 0 and 1. Results (not shown) do not change if a finer grid of points for λ_1 and λ_2 are chosen.

We use the default parametrization for the instance of the mSSL algorithm that performs "dynamic posterior exploration" (mSSL_dpe) which is the slowest, but the most accurate version of the algorithm. As recommended, we also run the "dynamic conditional posterior exploration" (mSSL_dcpe) and "separate variable and covariance selection" (sep_sslg) versions of the algorithm, but with inferior results (data not shown).

A unit variance of the predictors is required by both MRCE and mSSL algorithms. This condition is automatically satisfied since, in all simulated scenarios, the summary-level genetic associations with the risk factors in eq. (21) have unit variance. The simulation setup also simplifies the choice of prior variance of the non-zero regression coefficients used in MR-BMA. We use the same specification ($v = 1$) employed in MR^2. The output of MRCE and mSSL algorithms consists of the sparse Lasso solution of the direct causal effects $\Theta = (\theta_1, \ldots, \theta_q)^T$ and residual covariance Σ, or its inverse Ω, including the intercepts for each outcome. Thus, both MRCE and mSSL can be seen as the multi-response versions, with exposure selection, of multivariable MR-Egger [11], see Table S1. However, in the designed simulation setup, the unmeasured pleiotropic pathway is shared across responses and it induces residual correlation at the summary-level data as shown in eq. (5). Thus, its effect is detected by the estimated residual covariance Σ with the estimated intercepts close to zero (data not shown). Similar considerations hold when the residual correlation is induced by directly modelling the correlation between the individual-level responses' errors.

Performance measures

There are two important aspects when comparing methods for multivariable MR. First, the ability of the algorithms to detect the true causal risk factors and, second, the accuracy of the direct causal effect estimates. Receiver op-

erating characteristic (ROC) curves plot the true positive rate (TPR) against the false positive rate (FPR) and illustrate how well the methods can distinguish between true and false causal exposures when the discrimination threshold is varied. As discrimination threshold, in MV-MR we rank according to p-values of the direct causal effect estimates and in MR-BMA and MR^2 according to mPPI. The two multi-response implementations of Lasso rank exposures by their causal effect estimates of which the unimportant ones are forced to zero and thus excluded from the model. Consequently, these two methods do not provide a full ranking of all exposures, but rather a single threshold. Thus, they are presented in the ROC curves as a single point instead of a continuous line.

Second, to contrast the quality of the direct causal effect estimation, we calculate the sum of squared errors (SSE), defined as the squared difference between the estimated and the true simulated direct causal effect. The SSE captures both the squared bias and the variance, thus penalizing methods with a wide spread and high variability.

S3.2 Supplemental tables

	Designed for MR	Bayesian method	Multivariate outcomes	Selection risk factors	Estimation residual correlation	Reference
MV-MR	✓	✗	✗	✗	✗	Burgess *et al.* 2015 [9]
MR-BMA	✓	✓	✗	✓	✗	Zuber *et al.* 2020 [10]
MRCE	✗	✓	✓	✓	✓	Rothman *et al.* 2010 [3]
mSSL	✗	✓	✓	✓	✓	Deshpande *et al.* 2019 [4]
MR^2	✓	✓	✓	✓	✓	

Table S1. Overview of competing multivariable MR models that consider one outcome at-a-time and multi-response multivariable methods used in the simulation study. For each method, we indicate if: It has been designed specifically for MR, it is a Bayesian method, it can analyse multiple outcomes jointly, it can provide selection of important risk factors associated with the outcomes and it can estimate residual correlation between summary-level responses.

Scenario	Direct causal effect	Correlation among residuals	Further notes	Open parameters
I-Null	$\theta_k = 0$	No	Null direct causal effect of all exposures, only the confounders have an effect	Impact of confounder: $\theta_X^U = 2$, $\theta_Y^U = 1$
II-Confounding	$\theta_k \neq 0$	No	Direct causal effect on randomly selected exposure-confounder pairs	Impact of confounder: $\theta_X^U = 2$, $\theta_Y^U = 1$
III-Undirected pleiotropy	$\theta_k \neq 0$	Yes, induced by pleiotropic pathway	Additional effect of shared pleiotropic pathway A ("undirected": $A_l \gtrless 0$) on the level of outcomes	Pleiotropic effect: $\theta^A = \{0.25, 0.5, 0.75, 1, 1.5, 2\}$
IV-Directed pleiotropy	$\theta_k \neq 0$	Yes, induced by pleiotropic pathway	Additional effect of shared pleiotropic pathway A ("directed": $A_l > 0$) on the level of outcomes	Pleiotropic effect: $\theta^A = \{0.25, 0.5, 0.75, 1, 1.5, 2\}$
V-Dependence	$\theta_k \neq 0$	Yes, modelled directly	Correlation between individual-level responses' errors in eq. (5)	Correlation level: $r_Y = \{0, 0.2, 0.4, 0.6, 0.8\}$

Table S2. Overview of different simulations setting. Additional open parameter is the correlation between exposures $r_X = \{0, 0.2, 0.4, 0.6, 0.8\}$. Fixed parameters are the sample size of independent subjects ($N = 50,000$) for the first (risk factors) and second (responses) level of the simulations, the number of genetic variants used as instrumental variables ($n = 100$), the range of sought values for the exposure effects (β_{X_j}, $j = 1, \ldots, p$, should be in the range between -2 and 2), the percentage of simulated non-zero direct causal effects among the exposure-outcome combinations (30%), the range of sought values for the direct causal effects (θ_k, $k = 1, \ldots, q$, should be in the range between -2 and 2) and the heritability to generate $p = 15$ exposures ($h_X = 10\%$) and the proportion of variance explained for each of $q = 5$ outcomes ($h_Y = 25\%$).

	θ_X^U		θ_Y^U	
			1	2
r_X 0	1	MV-MR	0.958 (0.026)	0.958 (0.026)
		MR-BMA	0.960 (0.025)	0.961 (0.025)
		MRCE	0.959 (0.025)	0.959 (0.026)
		mSSL	0.901 (0.072)	0.909 (0.048)
		MR^2	**0.961** (0.025)	**0.962** (0.025)
	2	MV-MR	0.951 (0.030)	0.952 (0.030)
		MR-BMA	0.956 (0.028)	0.956 (0.029)
		MRCE	0.952 (0.036)	0.953 (0.037)
		mSSL	0.879 (0.067)	0.892 (0.044)
		MR^2	**0.961** (0.025)	**0.960** (0.025)
0.2	1	MV-MR	0.958 (0.024)	0.959 (0.024)
		MR-BMA	0.962 (0.025)	0.962 (0.025)
		MRCE	0.957 (0.029)	0.958 (0.026)
		mSSL	0.906 (0.048)	0.901 (0.050)
		MR^2	**0.964** (0.023)	**0.964** (0.025)
	2	MV-MR	0.951 (0.027)	0.951 (0.027)
		MR-BMA	**0.957** (0.027)	0.956 (0.027)
		MRCE	0.952 (0.027)	0.950 (0.029)
		mSSL	0.886 (0.071)	0.888 (0.039)
		MR^2	**0.957** (0.026)	**0.958** (0.024)
0.4	1	MV-MR	0.946 (0.041)	0.946 (0.041)
		MR-BMA	0.955 (0.034)	0.956 (0.034)
		MRCE	0.948 (0.038)	0.947 (0.039)
		mSSL	0.899 (0.050)	0.897 (0.043)
		MR^2	**0.962** (0.028)	**0.960** (0.032)
	2	MV-MR	0.940 (0.042)	0.937 (0.042)
		MR-BMA	0.950 (0.038)	0.949 (0.037)
		MRCE	0.943 (0.040)	0.942 (0.039)
		mSSL	0.870 (0.060)	0.880 (0.051)
		MR^2	**0.953** (0.037)	**0.953** (0.035)
0.6	1	MV-MR	0.928 (0.038)	0.930 (0.038)
		MR-BMA	0.940 (0.031)	0.941 (0.031)
		MRCE	0.936 (0.034)	0.937 (0.032)
		mSSL	0.884 (0.047)	0.893 (0.048)
		MR^2	**0.948** (0.030)	**0.946** (0.029)
	2	MV-MR	0.922 (0.040)	0.924 (0.038)
		MR-BMA	0.932 (0.034)	0.934 (0.035)
		MRCE	0.926 (0.035)	0.927 (0.037)
		mSSL	0.876 (0.044)	0.880 (0.046)
		MR^2	**0.939** (0.033)	**0.944** (0.029)
0.8	1	MV-MR	0.909 (0.034)	0.909 (0.033)
		MR-BMA	0.924 (0.040)	0.925 (0.040)
		MRCE	0.914 (0.039)	0.916 (0.039)
		mSSL	0.867 (0.058)	0.870 (0.048)
		MR^2	**0.932** (0.035)	**0.937** (0.036)
	2	MV-MR	0.896 (0.037)	0.899 (0.038)
		MR-BMA	0.916 (0.041)	0.919 (0.041)
		MRCE	0.900 (0.048)	0.904 (0.044)
		mSSL	0.846 (0.058)	0.851 (0.052)
		MR^2	**0.927** (0.038)	**0.932** (0.037)

Table S3. Scenario II-Confounding: Area under the receiver operating characteristic (AUC) of the simulated scenario, averaged over 50 replicates, with standard deviation across 50 replicates in brackets, illustrating the performance of different MR multivariable implementations and multi-response multivariable methods. In simulated Scenario II-Confounding, 30% of all exposure-confounder combinations are generated to have a direct causal effect. Open parameters are the correlation between exposures $r_X = \{0, 0.2, 0.4, 0.6, 0.8\}$ and the confounding effects on the exposures $\theta_X^U = \{1, 2\}$ and outcomes $\theta_Y^U = \{1, 2\}$. Best results are highlighted in bold.

			θ^A					
			0.25	0.5	0.75	1	1.5	2
r_X	0	MV-MR	0.943 (0.033)	0.927 (0.040)	0.912 (0.045)	0.896 (0.049)	0.859 (0.052)	0.824 (0.057)
		MR-BMA	0.948 (0.032)	0.933 (0.035)	0.916 (0.040)	0.899 (0.046)	0.864 (0.056)	0.832 (0.058)
		MRCE	0.943 (0.037)	0.935 (0.038)	0.930 (0.038)	0.923 (0.039)	0.905 (0.039)	0.891 (0.042)
		mSSL	0.889 (0.051)	0.868 (0.051)	0.867 (0.057)	0.861 (0.056)	0.845 (0.064)	0.814 (0.062)
		MR^2	**0.949** (0.030)	**0.945** (0.034)	**0.933** (0.035)	**0.932** (0.035)	**0.914** (0.042)	**0.909** (0.038)
	0.2	MV-MR	0.946 (0.033)	0.931 (0.036)	0.910 (0.047)	0.884 (0.056)	0.845 (0.067)	0.811 (0.073)
		MR-BMA	0.948 (0.033)	0.935 (0.033)	0.918 (0.039)	0.894 (0.047)	0.851 (0.061)	0.816 (0.064)
		MRCE	0.948 (0.035)	0.941 (0.032)	0.934 (0.038)	0.924 (0.041)	0.912 (0.046)	0.894 (0.050)
		mSSL	0.880 (0.064)	0.858 (0.066)	0.852 (0.059)	0.844 (0.068)	0.832 (0.069)	0.822 (0.054)
		MR^2	**0.952** (0.032)	**0.947** (0.031)	**0.946** (0.029)	**0.933** (0.031)	**0.925** (0.033)	**0.909** (0.043)
	0.4	MV-MR	0.942 (0.036)	0.922 (0.043)	0.898 (0.049)	0.875 (0.055)	0.827 (0.062)	0.792 (0.067)
		MR-BMA	0.945 (0.035)	0.929 (0.039)	0.913 (0.043)	0.892 (0.051)	0.851 (0.056)	0.809 (0.059)
		MRCE	0.938 (0.037)	0.930 (0.039)	0.924 (0.044)	0.913 (0.047)	0.900 (0.055)	0.884 (0.053)
		mSSL	0.867 (0.061)	0.860 (0.061)	0.849 (0.056)	0.845 (0.062)	0.830 (0.057)	0.803 (0.067)
		MR^2	**0.948** (0.036)	**0.941** (0.042)	**0.936** (0.040)	**0.927** (0.045)	**0.908** (0.052)	**0.898** (0.050)
	0.6	MV-MR	0.915 (0.045)	0.898 (0.052)	0.877 (0.056)	0.850 (0.059)	0.803 (0.065)	0.765 (0.075)
		MR-BMA	0.929 (0.037)	0.913 (0.041)	0.895 (0.045)	0.875 (0.050)	0.825 (0.068)	0.791 (0.081)
		MRCE	0.921 (0.041)	0.906 (0.051)	0.904 (0.046)	0.896 (0.046)	0.870 (0.050)	0.852 (0.059)
		mSSL	0.856 (0.055)	0.849 (0.052)	0.842 (0.065)	0.834 (0.068)	0.811 (0.068)	0.792 (0.071)
		MR^2	**0.938** (0.035)	**0.929** (0.037)	**0.918** (0.041)	**0.912** (0.042)	**0.895** (0.048)	**0.877** (0.055)
	0.8	MV-MR	0.887 (0.058)	0.862 (0.062)	0.828 (0.067)	0.793 (0.072)	0.746 (0.076)	0.703 (0.081)
		MR-BMA	0.897 (0.045)	0.879 (0.048)	0.854 (0.049)	0.827 (0.056)	0.776 (0.072)	0.743 (0.08)
		MRCE	0.882 (0.048)	0.874 (0.053)	0.869 (0.054)	0.854 (0.065)	0.835 (0.066)	0.827 (0.067)
		mSSL	0.838 (0.063)	0.809 (0.060)	0.795 (0.065)	0.791 (0.061)	0.749 (0.074)	0.736 (0.072)
		MR^2	**0.912** (0.042)	**0.902** (0.047)	**0.896** (0.042)	**0.884** (0.043)	**0.855** (0.054)	**0.838** (0.054)

Table S4. Simulation III-Undirected pleiotropy: Area under the receiver operating characteristic (AUC) of the simulated scenario, averaged over 50 replicates, with standard deviation across 50 replicates in brackets, illustrating the performance of different MR multivariable implementations and multi-response multivariable methods. In simulated Scenario III-Undirected pleiotropy, 30% of all exposure-confounder combinations are generated to have a direct causal effect. Open parameters are the correlation between exposures $r_X = \{0, 0.2, 0.4, 0.6, 0.8\}$ and the pleiotropic effect $\theta^A = \{0.25, 0.5, 0.75, 1, 1.5, 2\}$. Confounding effects on the exposures and outcomes are fixed at $\theta^U_X = 2$ and $\theta^U_Y = 1$, respectively. Best results are highlighted in bold.

		θ^A					
		0.25	0.5	0.75	1	1.5	2
r_X	0						
	MV-MR	0.943 (0.035)	0.931 (0.038)	0.917 (0.040)	0.900 (0.043)	0.857 (0.051)	0.818 (0.056)
	MR-BMA	0.947 (0.033)	0.937 (0.034)	0.924 (0.039)	0.907 (0.040)	0.863 (0.050)	0.826 (0.055)
	MRCE	0.946 (0.038)	0.943 (0.034)	0.934 (0.038)	0.928 (0.040)	**0.918** (0.037)	0.903 (0.044)
	mSSL	0.895 (0.052)	0.880 (0.057)	0.876 (0.053)	0.869 (0.062)	0.846 (0.055)	0.827 (0.063)
	MR^2	**0.952** (0.035)	**0.946** (0.038)	**0.938** (0.033)	**0.933** (0.033)	0.915 (0.040)	**0.904** (0.045)
	0.2						
	MV-MR	0.947 (0.034)	0.931 (0.039)	0.912 (0.048)	0.889 (0.054)	0.850 (0.061)	0.804 (0.076)
	MR-BMA	0.948 (0.034)	0.933 (0.037)	0.916 (0.043)	0.893 (0.051)	0.853 (0.064)	0.813 (0.072)
	MRCE	0.949 (0.035)	0.944 (0.033)	0.942 (0.034)	0.934 (0.031)	**0.923** (0.037)	**0.913** (0.042)
	mSSL	0.889 (0.050)	0.865 (0.069)	0.865 (0.060)	0.860 (0.059)	0.843 (0.057)	0.823 (0.067)
	MR^2	**0.950** (0.033)	**0.948** (0.029)	**0.940** (0.035)	**0.940** (0.029)	0.922 (0.039)	0.911 (0.039)
	0.4						
	MV-MR	0.941 (0.036)	0.923 (0.042)	0.900 (0.050)	0.877 (0.054)	0.832 (0.056)	0.783 (0.065)
	MR-BMA	0.947 (0.034)	0.934 (0.036)	0.916 (0.043)	0.895 (0.052)	0.852 (0.057)	0.806 (0.063)
	MRCE	0.943 (0.036)	0.936 (0.036)	0.929 (0.039)	0.923 (0.044)	0.912 (0.048)	**0.892** (0.051)
	mSSL	0.889 (0.058)	0.874 (0.058)	0.859 (0.060)	0.851 (0.054)	0.832 (0.060)	0.811 (0.062)
	MR^2	**0.948** (0.035)	**0.946** (0.036)	**0.934** (0.042)	**0.926** (0.044)	**0.914** (0.048)	0.889 (0.056)
	0.6						
	MV-MR	0.919 (0.044)	0.903 (0.047)	0.881 (0.052)	0.857 (0.057)	0.804 (0.070)	0.756 (0.078)
	MR-BMA	0.933 (0.037)	0.919 (0.044)	0.901 (0.050)	0.876 (0.058)	0.828 (0.062)	0.791 (0.071)
	MRCE	0.923 (0.041)	0.917 (0.041)	0.906 (0.047)	0.904 (0.048)	0.887 (0.048)	0.874 (0.051)
	mSSL	0.868 (0.056)	0.859 (0.053)	0.850 (0.060)	0.840 (0.055)	0.820 (0.058)	0.796 (0.072)
	MR^2	**0.940** (0.033)	**0.929** (0.039)	**0.920** (0.039)	**0.917** (0.043)	**0.895** (0.043)	**0.883** (0.050)
	0.8						
	MV-MR	0.885 (0.053)	0.852 (0.059)	0.818 (0.069)	0.793 (0.076)	0.738 (0.081)	0.703 (0.084)
	MR-BMA	0.895 (0.042)	0.874 (0.046)	0.850 (0.050)	0.826 (0.055)	0.780 (0.067)	0.747 (0.072)
	MRCE	0.887 (0.047)	0.880 (0.050)	0.874 (0.052)	0.867 (0.052)	0.852 (0.060)	0.834 (0.066)
	mSSL	0.842 (0.061)	0.827 (0.071)	0.807 (0.068)	0.797 (0.057)	0.766 (0.083)	0.748 (0.075)
	MR^2	**0.912** (0.043)	**0.905** (0.038)	**0.893** (0.044)	**0.881** (0.048)	**0.855** (0.054)	**0.843** (0.048)

Table S5. Simulation IV-Directed pleiotropy: Area under the receiver operating characteristic (AUC) of the simulated scenario, averaged over 50 replicates, with standard deviation across 50 replicates in brackets, illustrating the performance of different MR multivariable implementations and multi-response multivariable methods. In simulated Scenario IV-Directed pleiotropy, 30% of all exposure-confounder combinations are generated to have a direct causal effect. Open parameters are the correlation between exposures $r_X = \{0, 0.2, 0.4, 0.6, 0.8\}$ and the pleiotropic effect $\theta^A = \{0.25, 0.5, 0.75, 1, 1.5, 2\}$. Confounding effects on the exposures and outcomes are fixed at $\theta_X^U = 2$ and $\theta_Y^U = 1$, respectively. Best results are highlighted in bold.

			r_Y				
			0	0.2	0.4	0.6	0.8
r_X	0	MV-MR	0.951 (0.030)	0.951 (0.028)	0.953 (0.026)	0.950 (0.026)	0.954 (0.023)
		MR-BMA	0.956 (0.028)	0.954 (0.029)	0.956 (0.026)	0.955 (0.026)	0.956 (0.023)
		MRCE	0.951 (0.037)	0.951 (0.033)	0.951 (0.033)	0.953 (0.028)	0.955 (0.024)
		mSSL	0.886 (0.068)	0.893 (0.069)	0.886 (0.063)	0.884 (0.069)	0.896 (0.069)
		MR^2	**0.958** (0.024)	**0.962** (0.024)	**0.961** (0.027)	**0.962** (0.024)	**0.967** (0.020)
	0.2	MV-MR	0.951 (0.027)	0.949 (0.028)	0.951 (0.030)	0.951 (0.032)	0.950 (0.032)
		MR-BMA	**0.957** (0.027)	**0.957** (0.028)	**0.958** (0.029)	0.957 (0.029)	0.955 (0.030)
		MRCE	0.953 (0.028)	0.953 (0.027)	0.954 (0.029)	0.952 (0.030)	0.956 (0.031)
		mSSL	0.890 (0.065)	0.900 (0.067)	0.888 (0.071)	0.894 (0.082)	0.887 (0.085)
		MR^2	**0.957** (0.026)	0.956 (0.025)	0.955 (0.029)	**0.960** (0.027)	**0.968** (0.027)
	0.4	MV-MR	0.940 (0.042)	0.949 (0.030)	0.944 (0.034)	0.946 (0.032)	0.948 (0.029)
		MR-BMA	0.950 (0.038)	0.953 (0.029)	0.952 (0.033)	0.953 (0.032)	0.956 (0.031)
		MRCE	0.942 (0.041)	0.948 (0.032)	0.943 (0.038)	0.945 (0.036)	0.949 (0.035)
		mSSL	0.880 (0.050)	0.885 (0.051)	0.877 (0.064)	0.885 (0.070)	0.889 (0.075)
		MR^2	**0.953** (0.037)	**0.958** (0.026)	**0.956** (0.029)	**0.957** (0.031)	**0.961** (0.029)
	0.6	MV-MR	0.922 (0.040)	0.928 (0.037)	0.925 (0.040)	0.930 (0.037)	0.930 (0.044)
		MR-BMA	0.932 (0.034)	0.935 (0.040)	0.934 (0.037)	0.936 (0.032)	0.935 (0.033)
		MRCE	0.924 (0.036)	0.933 (0.040)	0.927 (0.042)	0.932 (0.037)	0.930 (0.046)
		mSSL	0.878 (0.066)	0.870 (0.054)	0.875 (0.053)	0.869 (0.067)	0.878 (0.079)
		MR^2	**0.939** (0.033)	**0.943** (0.033)	**0.943** (0.036)	**0.952** (0.031)	**0.951** (0.029)
	0.8	MV-MR	0.896 (0.037)	0.900 (0.039)	0.901 (0.041)	0.902 (0.039)	0.901 (0.037)
		MR-BMA	0.916 (0.041)	0.909 (0.039)	0.912 (0.037)	0.911 (0.039)	0.910 (0.038)
		MRCE	0.904 (0.043)	0.899 (0.049)	0.901 (0.042)	0.903 (0.041)	0.909 (0.042)
		mSSL	0.849 (0.059)	0.821 (0.082)	0.843 (0.073)	0.855 (0.062)	0.835 (0.088)
		MR^2	**0.927** (0.038)	**0.922** (0.032)	**0.926** (0.035)	**0.934** (0.030)	**0.936** (0.035)

Table S6. Simulation V-Dependence: Area under the receiver operating characteristic (AUC) of the simulated scenario, averaged over 50 replicates, with standard deviation across 50 replicates in brackets, illustrating the performance of different MR multivariable implementations and multi-response multivariable methods. In simulated Scenario V-Dependence, 30% of all exposure-confounder combinations are generated to have a direct causal effect. Open parameters are the correlation between exposures $r_X = \{0, 0.2, 0.4, 0.6, 0.8\}$ and the correlation between individual-level responses' errors $r_Y = \{0, 0.2, 0.4, 0.6, 0.8\}$. Confounding effects on the exposures and outcomes are fixed at $\theta_X^U = 2$ and $\theta_Y^U = 1$, respectively. Best results are highlighted in bold.

			r_Y				
			0	0.2	0.4	0.6	0.8
r_X	0	MV-MR	0.001 (<0.001)	0.001 (<0.001)	0.001 (<0.001)	0.001 (<0.001)	0.001 (<0.001)
		MR-BMA	**0.000** (<0.001)	**0.000** (<0.001)	**0.000** (<0.001)	**0.000** (<0.001)	**0.000** (<0.001)
		MRCE	**0.000** (<0.001)	**0.000** (<0.001)	**0.000** (<0.001)	**0.000** (<0.001)	**0.000** (<0.001)
		mSSL	**0.000** (<0.001)	**0.000** (<0.001)	**0.000** (<0.001)	**0.000** (<0.001)	**0.000** (<0.001)
		MR^2	**0.000** (<0.001)	**0.000** (<0.001)	**0.000** (<0.001)	**0.000** (<0.001)	**0.000** (<0.001)
	0.2	MV-MR	0.001 (<0.001)	0.001 (<0.001)	0.001 (<0.001)	0.001 (<0.001)	0.001 (<0.001)
		MR-BMA	**0.000** (<0.001)	**0.000** (<0.001)	**0.000** (<0.001)	**0.000** (<0.001)	**0.000** (<0.001)
		MRCE	**0.000** (<0.001)	**0.000** (<0.001)	**0.000** (<0.001)	**0.000** (<0.001)	**0.000** (<0.001)
		mSSL	**0.000** (<0.001)	**0.000** (<0.001)	**0.000** (<0.001)	**0.000** (<0.001)	**0.000** (<0.001)
		MR^2	**0.000** (<0.001)	**0.000** (<0.001)	**0.000** (<0.001)	**0.000** (<0.001)	**0.000** (<0.001)
	0.4	MV-MR	0.001 (<0.001)	0.001 (<0.001)	0.001 (<0.001)	0.001 (<0.001)	0.001 (<0.001)
		MR-BMA	**0.000** (<0.001)	**0.000** (<0.001)	**0.000** (<0.001)	**0.000** (<0.001)	**0.000** (<0.001)
		MRCE	0.001 (<0.001)	0.001 (<0.001)	0.001 (<0.001)	0.001 (<0.001)	0.001 (<0.001)
		mSSL	**0.000** (<0.001)	**0.000** (<0.001)	**0.000** (<0.001)	**0.000** (<0.001)	**0.000** (<0.001)
		MR^2	**0.000** (<0.001)	**0.000** (<0.001)	**0.000** (<0.001)	**0.000** (<0.001)	**0.000** (<0.001)
	0.6	MV-MR	0.001 (<0.001)	0.001 (<0.001)	0.001 (<0.001)	0.001 (<0.001)	0.001 (<0.001)
		MR-BMA	**0.000** (<0.001)	**0.000** (<0.001)	**0.000** (<0.001)	**0.000** (<0.001)	**0.000** (<0.001)
		MRCE	0.001 (<0.001)	0.001 (<0.001)	0.001 (<0.001)	0.001 (<0.001)	0.001 (<0.001)
		mSSL	**0.000** (<0.001)	**0.000** (<0.001)	**0.000** (<0.001)	**0.000** (<0.001)	**0.000** (<0.001)
		MR^2	**0.000** (<0.001)	**0.000** (<0.001)	**0.000** (<0.001)	**0.000** (<0.001)	**0.000** (<0.001)
	0.8	MV-MR	0.003 (<0.001)	0.003 (0.001)	0.003 (0.001)	0.003 (0.001)	0.003 (0.001)
		MR-BMA	**0.000** (<0.001)	**0.000** (<0.001)	**0.000** (<0.001)	**0.000** (<0.001)	**0.000** (<0.001)
		MRCE	0.001 (<0.001)	0.001 (<0.001)	0.001 (<0.001)	0.001 (<0.001)	0.001 (<0.001)
		mSSL	**0.000** (<0.001)	**0.000** (<0.001)	**0.000** (<0.001)	**0.000** (<0.001)	**0.000** (<0.001)
		MR^2	**0.000** (<0.001)	**0.000** (<0.001)	**0.000** (<0.001)	**0.000** (<0.001)	**0.000** (<0.001)

Table S7. Scenario I-Null: Sum of squared errors (SSE) of the simulated scenario, averaged over 50 replicates, with standard deviation across 50 replicates in brackets, illustrating the performance of different MR multivariable implementations and multi-response multivariable methods to estimate the true simulated effect size. In simulated Scenario I- Null, no direct causal effects are simulated and the outcomes are generated based only on the effect of the confounders. Open parameters are the correlation between exposures $r_X = \{0, 0.2, 0.4, 0.6, 0.8\}$ and the correlation between individual-level responses' errors $r_Y = \{0, 0.2, 0.4, 0.6, 0.8\}$. Best results are highlighted in bold.

	θ_X^U		θ_Y^U 1	2
r_X 0	1	MV-MR	0.494 (0.200)	0.491 (0.203)
		MR-BMA	0.294 (0.141)	0.287 (0.140)
		MRCE	0.368 (0.161)	0.364 (0.163)
		mSSL	0.417 (0.644)	0.333 (0.179)
		MR^2	**0.261** (0.126)	**0.252** (0.120)
	2	MV-MR	0.611 (0.246)	0.616 (0.247)
		MR-BMA	0.347 (0.163)	0.341 (0.159)
		MRCE	0.449 (0.196)	0.449 (0.199)
		mSSL	0.577 (0.831)	0.432 (0.208)
		MR^2	**0.316** (0.143)	**0.311** (0.140)
0.2	1	MV-MR	0.577 (0.227)	0.598 (0.261)
		MR-BMA	0.338 (0.165)	0.354 (0.200)
		MRCE	0.416 (0.182)	0.432 (0.221)
		mSSL	0.377 (0.181)	0.398 (0.216)
		MR^2	**0.297** (0.148)	**0.309** (0.177)
	2	MV-MR	0.730 (0.322)	0.743 (0.322)
		MR-BMA	0.417 (0.220)	0.425 (0.221)
		MRCE	0.530 (0.268)	0.539 (0.270)
		mSSL	0.646 (0.966)	0.493 (0.223)
		MR^2	**0.378** (0.199)	**0.386** (0.200)
0.4	1	MV-MR	0.711 (0.325)	0.863 (0.325)
		MR-BMA	0.410 (0.209)	0.478 (0.211)
		MRCE	0.522 (0.257)	0.633 (0.263)
		mSSL	0.462 (0.252)	0.589 (0.251)
		MR^2	**0.355** (0.185)	**0.430** (0.189)
	2	MV-MR	0.704 (0.385)	0.875 (0.493)
		MR-BMA	0.409 (0.235)	0.478 (0.299)
		MRCE	0.523 (0.321)	0.638 (0.402)
		mSSL	0.461 (0.340)	0.552 (0.372)
		MR^2	**0.353** (0.216)	**0.432** (0.265)
0.6	1	MV-MR	0.994 (0.383)	1.005 (0.597)
		MR-BMA	0.555 (0.239)	0.553 (0.373)
		MRCE	0.730 (0.331)	0.729 (0.516)
		mSSL	0.569 (0.277)	0.564 (1.117)
		MR^2	**0.440** (0.217)	**0.444** (0.340)
	2	MV-MR	1.246 (0.490)	1.261 (0.605)
		MR-BMA	0.673 (0.302)	0.677 (0.514)
		MRCE	0.904 (0.405)	0.925 (0.367)
		mSSL	0.881 (0.374)	0.718 (0.414)
		MR^2	**0.567** (0.269)	**0.577** (0.331)
0.8	1	MV-MR	2.015 (1.013)	1.990 (0.984)
		MR-BMA	1.223 (0.724)	1.183 (0.664)
		MRCE	1.330 (0.684)	1.301 (0.643)
		mSSL	1.178 (1.093)	1.022 (0.647)
		MR^2	**0.793** (0.533)	**0.761** (0.455)
	2	MV-MR	2.496 (1.192)	2.519 (1.191)
		MR-BMA	1.441 (0.807)	1.469 (0.808)
		MRCE	1.667 (0.806)	1.689 (0.788)
		mSSL	1.475 (1.113)	1.392 (0.810)
		MR^2	**1.057** (0.629)	**1.077** (0.614)

Table S8. Scenario II-Confounding: Sum of squared errors (SSE) of the simulated scenario, averaged over 50 replicates, with standard deviation across 50 replicates in brackets, illustrating the performance of different MR multivariable implementations and multi-response multivariable methods to estimate the true effect size. In simulated Scenario II-Confounding, 30% of all exposure-confounder combinations are generated to have a direct causal effect. Open parameters are the correlation between exposures $r_X = \{0, 0.2, 0.4, 0.6, 0.8\}$ and confounding effects on the exposures $\theta_X^U = \{1, 2\}$ and outcomes $\theta_Y^U = \{1, 2\}$. Best results are highlighted in bold.

		θ^A					
		0.25	0.5	0.75	1	1.5	2
r_X	0						
	MV-MR	0.683 (0.254)	0.944 (0.329)	1.320 (0.417)	1.874 (0.565)	3.559 (1.094)	5.801 (1.859)
	MR-BMA	0.407 (0.179)	0.502 (0.210)	0.648 (0.266)	0.859 (0.343)	1.556 (0.596)	2.662 (1.021)
	MRCE	0.521 (0.209)	0.607 (0.239)	0.670 (0.274)	0.736 (0.297)	1.003 (0.601)	1.340 (0.887)
	mSSL	0.465 (0.249)	0.530 (0.257)	0.560 (0.277)	0.605 (0.294)	0.721 (0.393)	1.017 (0.788)
	MR^2	**0.360** (0.160)	**0.406** (0.159)	**0.431** (0.168)	**0.462** (0.162)	**0.610** (0.499)	**0.652** (0.337)
	0.2						
	MV-MR	0.783 (0.379)	1.054 (0.442)	1.514 (0.553)	2.179 (0.686)	4.015 (1.321)	6.445 (2.103)
	MR-BMA	0.428 (0.209)	0.563 (0.242)	0.774 (0.288)	1.065 (0.375)	1.756 (0.571)	2.731 (0.841)
	MRCE	0.567 (0.274)	0.666 (0.316)	0.786 (0.361)	0.893 (0.431)	1.141 (0.649)	1.425 (0.876)
	mSSL	0.561 (0.482)	0.630 (0.443)	0.724 (0.461)	0.747 (0.462)	0.895 (0.521)	**1.081** (0.516)
	MR^2	**0.370** (0.212)	**0.425** (0.231)	**0.498** (0.253)	**0.540** (0.267)	**0.735** (0.484)	1.092 (0.965)
	0.4						
	MV-MR	0.936 (0.376)	1.261 (0.450)	1.822 (0.578)	2.673 (0.903)	5.035 (1.847)	8.244 (3.092)
	MR-BMA	0.517 (0.240)	0.648 (0.273)	0.869 (0.335)	1.201 (0.450)	2.204 (0.906)	3.542 (1.403)
	MRCE	0.689 (0.302)	0.778 (0.321)	0.882 (0.353)	1.032 (0.498)	1.325 (0.917)	1.629 (1.073)
	mSSL	0.590 (0.289)	0.682 (0.302)	0.702 (0.295)	0.778 (0.354)	1.089 (0.872)	1.349 (0.952)
	MR^2	**0.436** (0.237)	**0.491** (0.241)	**0.520** (0.242)	**0.647** (0.621)	**0.844** (0.899)	**1.088** (1.137)
	0.6						
	MV-MR	1.332 (0.642)	1.826 (0.763)	2.699 (1.051)	3.869 (1.502)	7.017 (2.846)	11.518 (5.051)
	MR-BMA	0.774 (0.571)	0.971 (0.719)	1.330 (0.883)	1.714 (1.036)	2.965 (1.483)	4.487 (1.944)
	MRCE	0.947 (0.551)	1.094 (0.613)	1.251 (0.668)	1.434 (0.877)	1.806 (1.128)	2.191 (1.176)
	mSSL	0.877 (0.663)	0.933 (0.678)	1.013 (0.720)	1.104 (0.790)	1.410 (0.943)	1.795 (1.149)
	MR^2	**0.624** (0.523)	**0.693** (0.614)	**0.759** (0.658)	**0.858** (0.722)	**1.125** (0.987)	**1.565** (1.284)
	0.8						
	MV-MR	2.716 (1.263)	3.693 (1.544)	5.272 (2.085)	7.728 (2.944)	13.611 (5.260)	22.417 (8.816)
	MR-BMA	1.617 (0.722)	1.922 (0.858)	2.432 (1.045)	3.065 (1.284)	4.913 (1.875)	7.036 (2.735)
	MRCE	1.891 (0.764)	2.118 (0.858)	2.380 (1.091)	2.667 (1.275)	3.004 (1.203)	3.456 (1.478)
	mSSL	1.538 (0.795)	1.929 (1.204)	2.102 (1.180)	2.325 (1.316)	3.309 (2.217)	3.817 (2.051)
	MR^2	**1.198** (0.594)	**1.391** (0.644)	**1.560** (0.759)	**1.731** (0.815)	**2.251** (1.153)	**2.707** (1.310)

Table S9. Simulation III-Undirected pleiotropy: Sum of squared errors (SSE) of the simulated scenario, averaged over 50 replicates, with standard deviation across 50 replicates in brackets, illustrating the performance of different MR multivariable implementations and multi-response multivariable methods to estimate the true effect size. In simulated Scenario III-Undirected pleiotropy, 30% of all exposure-confounder combinations are generated to have a direct causal effect. Open parameters are the correlation between exposures $r_X = \{0, 0.2, 0.4, 0.6, 0.8\}$ and the pleiotropic effect $\theta^A = \{0.25, 0.5, 0.75, 1, 1.5, 2\}$. Confounding effects on the exposures and outcomes are fixed at $\theta^U_X = 2$ and $\theta^U_Y = 1$, respectively. Best results are highlighted in bold.

			θ^A					
			0.25	0.5	0.75	1	1.5	2
r_X	0	MV-MR	0.679 (0.255)	0.909 (0.332)	1.290 (0.456)	1.829 (0.687)	3.383 (1.331)	5.621 (2.265)
		MR-BMA	0.410 (0.183)	0.510 (0.214)	0.658 (0.274)	0.895 (0.358)	1.659 (0.603)	2.642 (0.885)
		MRCE	0.491 (0.193)	0.534 (0.210)	0.566 (0.219)	0.616 (0.23)	0.756 (0.298)	0.960 (0.422)
		mSSL	0.442 (0.244)	0.491 (0.296)	0.526 (0.271)	0.564 (0.266)	0.681 (0.367)	**0.858** (0.420)
		MR^2	**0.366** (0.165)	**0.412** (0.166)	**0.453** (0.187)	**0.507** (0.205)	**0.588** (0.252)	0.938 (1.021)
	0.2	MV-MR	0.768 (0.363)	1.021 (0.438)	1.465 (0.607)	2.080 (0.857)	3.767 (1.538)	6.349 (2.649)
		MR-BMA	0.430 (0.199)	0.560 (0.236)	0.786 (0.340)	1.088 (0.500)	1.818 (0.799)	2.849 (1.118)
		MRCE	0.540 (0.272)	0.584 (0.278)	0.659 (0.306)	0.722 (0.326)	0.893 (0.374)	1.062 (0.437)
		mSSL	0.484 (0.326)	0.591 (0.485)	0.658 (0.440)	0.696 (0.451)	0.810 (0.415)	**0.989** (0.513)
		MR^2	**0.371** (0.195)	**0.424** (0.221)	**0.496** (0.227)	**0.546** (0.249)	**0.673** (0.359)	1.033 (0.996)
	0.4	MV-MR	0.924 (0.393)	1.245 (0.500)	1.780 (0.695)	2.541 (0.989)	4.782 (1.885)	8.228 (3.153)
		MR-BMA	0.508 (0.244)	0.624 (0.260)	0.819 (0.348)	1.091 (0.495)	1.970 (0.899)	3.301 (1.250)
		MRCE	0.645 (0.296)	0.699 (0.305)	0.760 (0.317)	0.850 (0.359)	1.050 (0.500)	1.260 (0.640)
		mSSL	0.546 (0.279)	0.608 (0.286)	0.678 (0.285)	0.709 (0.296)	0.853 (0.347)	1.167 (0.695)
		MR^2	**0.430** (0.235)	**0.480** (0.243)	**0.526** (0.258)	**0.593** (0.275)	**0.827** (0.765)	**0.994** (0.696)
	0.6	MV-MR	1.294 (0.618)	1.764 (0.755)	2.548 (1.036)	3.613 (1.409)	6.802 (2.678)	11.567 (4.625)
		MR-BMA	0.732 (0.451)	0.876 (0.481)	1.156 (0.570)	1.508 (0.663)	2.581 (1.055)	4.122 (1.611)
		MRCE	0.903 (0.516)	0.975 (0.549)	1.072 (0.589)	1.172 (0.618)	1.406 (0.719)	1.683 (0.702)
		mSSL	0.835 (0.669)	0.882 (0.658)	0.946 (0.676)	1.050 (0.700)	1.261 (0.788)	1.637 (0.883)
		MR^2	**0.593** (0.432)	**0.677** (0.518)	**0.748** (0.605)	**0.821** (0.681)	**1.041** (0.855)	**1.420** (1.064)
	0.8	MV-MR	2.661 (1.249)	3.515 (1.528)	5.054 (2.103)	7.234 (2.824)	13.513 (5.142)	22.485 (8.544)
		MR-BMA	1.606 (0.691)	1.892 (0.819)	2.381 (0.975)	3.102 (1.272)	5.314 (2.453)	7.718 (3.558)
		MRCE	1.791 (0.732)	1.892 (0.770)	2.100 (0.867)	2.257 (0.940)	2.685 (1.216)	3.140 (1.419)
		mSSL	1.393 (0.757)	1.651 (0.982)	1.894 (1.155)	2.103 (1.246)	2.839 (1.726)	3.259 (1.913)
		MR^2	**1.205** (0.588)	**1.360** (0.604)	**1.514** (0.716)	**1.703** (0.766)	**2.156** (1.014)	**2.689** (1.387)

Table S10. Simulation IV-Directed pleiotropy: Sum of squared errors (SSE) of the simulated scenario, averaged over 50 replicates, with standard deviation across 50 replicates in brackets, illustrating the performance of different MR multivariable implementations and multi-response multivariable methods to estimate the true effect size. In simulated Scenario IV-Directed pleiotropy, 30% of all exposure-confounder combinations are generated to have a direct causal effect. Open parameters are the correlation between exposures $r_X = \{0, 0.2, 0.4, 0.6, 0.8\}$ and the pleiotropic effect $\theta^A = \{0.25, 0.5, 0.75, 1, 1.5, 2\}$. Confounding effects on the exposures and outcomes are fixed at $\theta_X^U = 2$ and $\theta_Y^U = 1$, respectively. Best results are highlighted in bold.

r_X			r_Y				
			0	0.2	0.4	0.6	0.8
	0	MV-MR	0.611 (0.246)	0.663 (0.315)	0.658 (0.296)	0.667 (0.305)	0.653 (0.308)
		MR-BMA	0.347 (0.163)	0.363 (0.216)	0.361 (0.207)	0.372 (0.203)	0.357 (0.193)
		MRCE	0.449 (0.196)	0.475 (0.254)	0.462 (0.227)	0.463 (0.235)	0.427 (0.228)
		mSSL	0.577 (0.831)	0.582 (0.858)	0.694 (1.053)	0.664 (1.062)	0.623 (1.036)
		MR^2	**0.315** (0.143)	**0.329** (0.207)	**0.325** (0.216)	**0.305** (0.189)	**0.268** (0.183)
	0.2	MV-MR	0.730 (0.322)	0.744 (0.298)	0.755 (0.279)	0.745 (0.276)	0.737 (0.278)
		MR-BMA	0.417 (0.220)	0.433 (0.219)	0.440 (0.218)	0.422 (0.210)	0.425 (0.217)
		MRCE	0.530 (0.268)	0.541 (0.252)	0.540 (0.234)	0.512 (0.216)	0.483 (0.230)
		mSSL	0.646 (0.966)	0.616 (0.964)	0.638 (0.964)	0.594 (0.969)	0.830 (1.773)
		MR^2	**0.378** (0.199)	**0.378** (0.197)	**0.385** (0.197)	**0.355** (0.202)	**0.321** (0.201)
	0.4	MV-MR	0.863 (0.385)	0.851 (0.348)	0.847 (0.340)	0.867 (0.346)	0.853 (0.343)
		MR-BMA	0.478 (0.235)	0.455 (0.221)	0.462 (0.215)	0.470 (0.224)	0.484 (0.234)
		MRCE	0.633 (0.321)	0.594 (0.264)	0.590 (0.236)	0.604 (0.258)	0.566 (0.227)
		mSSL	0.589 (0.340)	0.574 (0.405)	0.674 (0.868)	0.722 (1.014)	0.810 (1.406)
		MR^2	**0.430** (0.216)	**0.401** (0.201)	**0.388** (0.191)	**0.361** (0.161)	**0.334** (0.152)
	0.6	MV-MR	1.246 (0.597)	1.205 (0.519)	1.212 (0.514)	1.200 (0.464)	1.195 (0.470)
		MR-BMA	0.673 (0.373)	0.651 (0.329)	0.680 (0.369)	0.700 (0.348)	0.693 (0.337)
		MRCE	0.904 (0.516)	0.849 (0.413)	0.867 (0.431)	0.849 (0.423)	0.805 (0.355)
		mSSL	0.881 (1.117)	0.851 (1.095)	0.845 (1.168)	0.891 (1.167)	1.005 (1.762)
		MR^2	**0.567** (0.340)	**0.534** (0.289)	**0.545** (0.329)	**0.521** (0.278)	**0.438** (0.225)
	0.8	MV-MR	2.496 (1.192)	2.391 (0.968)	2.310 (0.942)	2.324 (0.939)	2.287 (0.979)
		MR-BMA	1.441 (0.807)	1.341 (0.587)	1.278 (0.525)	1.346 (0.570)	1.342 (0.635)
		MRCE	1.667 (0.806)	1.598 (0.630)	1.474 (0.545)	1.511 (0.609)	1.430 (0.642)
		mSSL	1.475 (1.113)	1.493 (1.566)	1.415 (1.533)	1.371 (1.517)	1.626 (2.440)
		MR^2	**1.057** (0.629)	**0.977** (0.475)	**0.867** (0.425)	**0.844** (0.450)	**0.754** (0.425)

Table S11. Simulation V-Dependence: Sum of squared errors (SSE) of the simulated scenario, averaged over 50 replicates, with standard deviation across 50 replicates in brackets, illustrating the performance of different MR multivariable implementations and multi-response multivariable methods to estimate the true effect size. In simulated Scenario V-Dependence, 30% of all exposure-confounder combinations are generated to have a direct causal effect. Open parameters are the correlation between exposures $r_X = \{0, 0.2, 0.4, 0.6, 0.8\}$ and the correlation between individual-level responses' errors $r_Y = \{0, 0.2, 0.4, 0.6, 0.8\}$. Confounding effects on the exposures and outcomes are fixed at $\theta_X^U = 2$ and $\theta_Y^U = 1$, respectively. Best results are highlighted in bold.

Simulated exposure effect	Average simulated proportion		Scenario II Confounding	Scenario III Undirected pleiotropy	Scenario IV Directed pleiotropy	Scenario V Dependence
Distinct	0.373	MV-MR	0.742 (0.437)	0.516 (0.500)	0.516 (0.500)	0.680 (0.467)
		MR-BMA	0.779 (0.415)	0.534 (0.499)	0.567 (0.496)	0.697 (0.460)
		mSSL	0.763 (0.426)	0.693 (0.461)	0.690 (0.463)	0.697 (0.460)
		MR^2	**0.783** (0.413)	**0.697** (0.460)	**0.722** (0.448)	**0.743** (0.437)
Shared = 2	0.315	MV-MR	**0.566** (0.496)	0.224 (0.417)	0.269 (0.444)	0.438 (0.496)
		MR-BMA	0.551 (0.498)	0.260 (0.439)	0.309 (0.462)	0.433 (0.496)
		mSSL	0.551 (0.498)	0.422 (0.494)	0.430 (0.495)	0.453 (0.498)
		MR^2	0.556 (0.496)	**0.435** (0.496)	**0.453** (0.498)	**0.463** (0.496)
Shared = 3	0.122	MV-MR	**0.443** (0.497)	0.173 (0.379)	0.173 (0.379)	0.265 (0.442)
		MR-BMA	0.381 (0.486)	0.212 (0.409)	0.240 (0.428)	0.286 (0.452)
		mSSL	0.412 (0.493)	0.298 (0.458)	0.337 (0.473)	**0.306** (0.461)
		MR^2	0.392 (0.489)	**0.308** (0.462)	**0.346** (0.476)	**0.306** (0.461)
Shared = 4	0.023	MV-MR	**0.440** (0.498)	0.133 (0.342)	0.133 (0.342)	0.231 (0.423)
		MR-BMA	0.400 (0.492)	0.200 (0.403)	0.133 (0.342)	0.269 (0.445)
		mSSL	**0.440** (0.498)	**0.267** (0.445)	**0.267** (0.445)	0.346 (0.478)
		MR^2	**0.440** (0.498)	0.233 (0.430)	**0.267** (0.445)	**0.385** (0.488)

Table S12. Power to detect distinct and shared direct causal effects at a common false positives rate in different simulated scenarios when the correlation between exposures is set at $r_X = 0.6$ over 50 replicates with standard deviation across 50 replicates in brackets. Simulated exposure effects are either distinct, where an exposure has only a causal association with one outcome or shared, where the exposure exerts its effect on multiple (2,3,4) responses (Shared = 5 was not simulated). The average simulated proportion indicates the simulated proportion (over all exposure-response combinations) that an exposure is associated with one (distinct) or multiple (shared) responses. In baseline Scenario II-Confounding, only the confounding effects on the exposures and outcomes, $\theta_X^U = 2$ and $\theta_Y^U = 1$, respectively, are used to induce residual correlation between the responses. Residual correlation induced by shared undirected pleiotropy and shared directed pleiotropy with pleiotropic effect $\theta^A = 1$ are presented in Scenario III-Undirected pleiotropy and Scenario IV-Directed pleiotropy, respectively. In Scenario IV-Dependence, outcomes are simulated with the correlation between individual-level responses' errors fixed at $r_Y = 0.6$. Best results are highlighted in bold. MRCE method is not considered since it is not possible to match its type I error with the other methods. For more details on the simulations setting, see Table S2.

S3.3 Supplemental figures

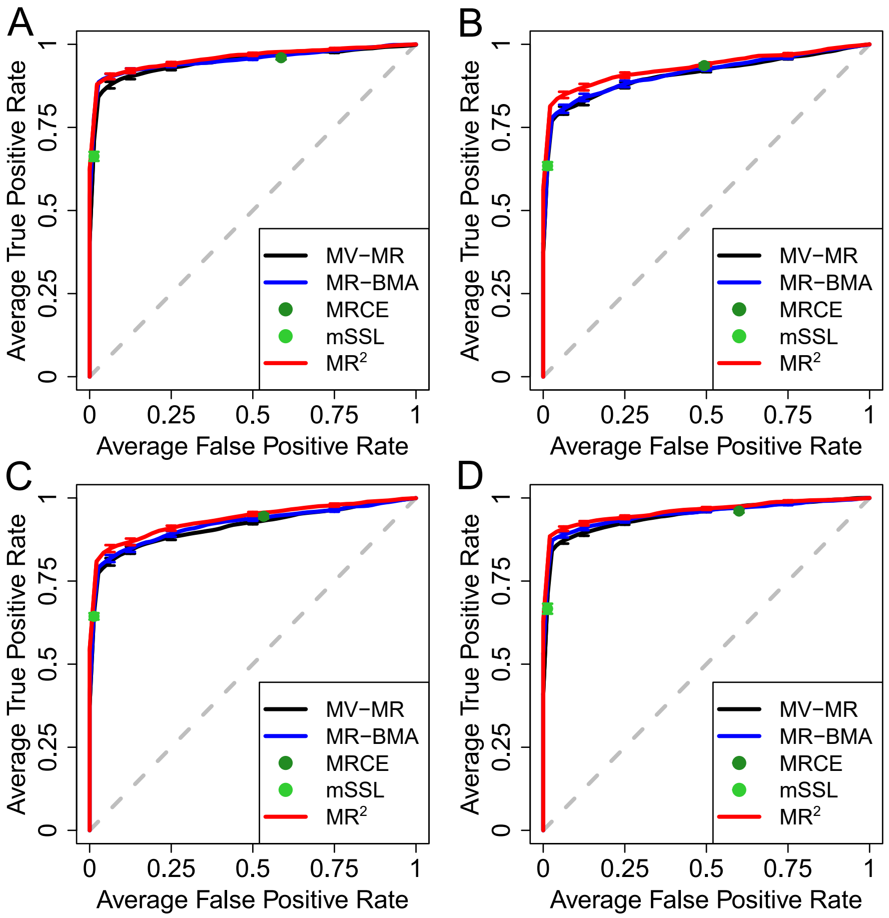

Figure S1. Receiver operating characteristic (ROC) curves in different simulated scenarios when there is no correlation between exposures, averaged over 50 replicates, illustrating the performance of different MR multivariable implementations and multi-response multivariable methods to distinguish between true and false causal exposures for the simulated outcomes by plotting the true (TPR) against the false positive rate (FPR). (**A**) depicts baseline Scenario II-Confounding where only the confounding effects on the exposures and outcomes, $\theta_X^U = 2$ and $\theta_Y^U = 1$, respectively, are used to simulate the data. Residual correlation induced by shared undirected pleiotropy (Scenario III-Undirected pleiotropy) and shared directed pleiotropy (Scenario IV-Directed pleiotropy) with pleiotropic effect set at $\theta^A = 1$ are shown in (**B**) and (**C**), respectively. (**D**) displays the performance of the different methods in Scenario IV-Dependence, where the correlation between individual-level responses' errors is fixed at $r_Y = 0.6$. Vertical bars in each ROC curve, at specific FPR levels, indicate standard error. For more details on the simulations setting, see Table S2.

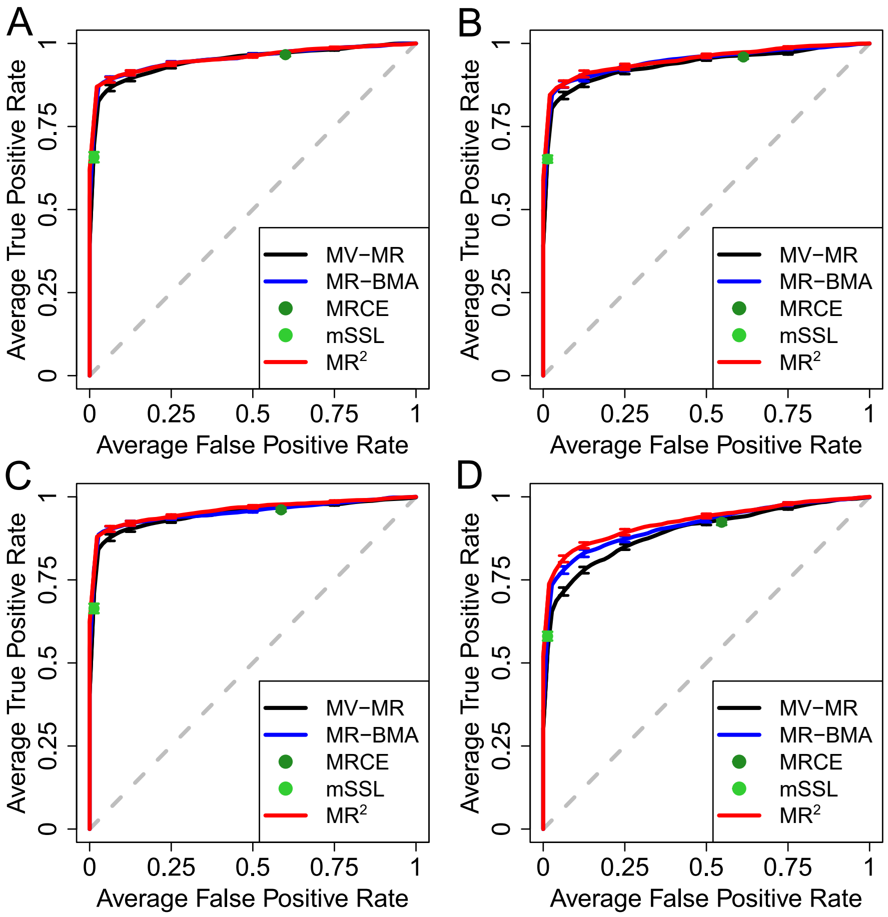

Figure S2. Simulation II-Confounding: Receiver operating characteristic (ROC) for different levels of correlation between the exposures, averaged over 50 replicates, illustrating the performance of different MR multivariable implementations and multi-response multivariable methods to distinguish between true and false causal exposures for the simulated outcomes by plotting the true (TPR) against the false positive rate (FPR). Correlation between the exposures varies from light correlation $r_X = 0.2$ in (**A**) to strong correlation $r_X = 0.8$ in (**D**) with confounding effects on the exposures and outcomes fixed at $\theta_X^U = 2$ and $\theta_Y^U = 1$, respectively. Vertical bars in each ROC curve, at specific FPR levels, indicate standard error.

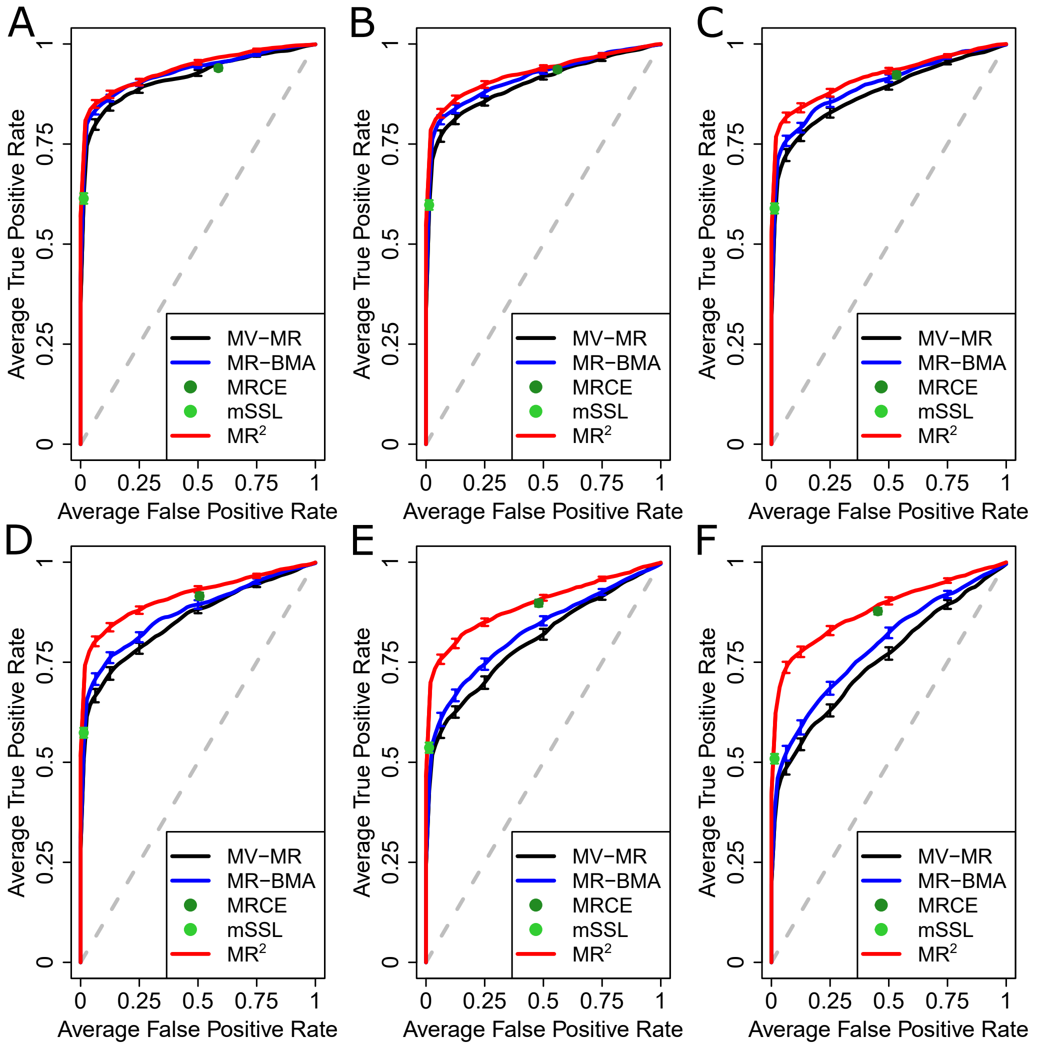

Figure S3. Simulation IV-Directed pleiotropy: Receiver operating characteristic (ROC) curves for different levels of the pleiotropic pathway effect θ^A and when the correlation between exposures is set at $r_X = 0.6$, averaged over 50 replicates, illustrating the performance of different MR implementations and multi-response statistical methods to distinguish between true and false causal exposures for the simulated outcomes by plotting the true (TPR) against the false positive rate (FPR). Pleiotropic pathway effect varies from (**A**) to (**F**) with values $\theta^A = \{0.25, 0.5, 0.75, 1, 1.5, 2\}$. Confounding effects on the exposures and outcomes are fixed at $\theta_X^U = 2$ and $\theta_Y^U = 1$, respectively. Vertical bars in each ROC curve, at specific FPR levels, indicate standard error.

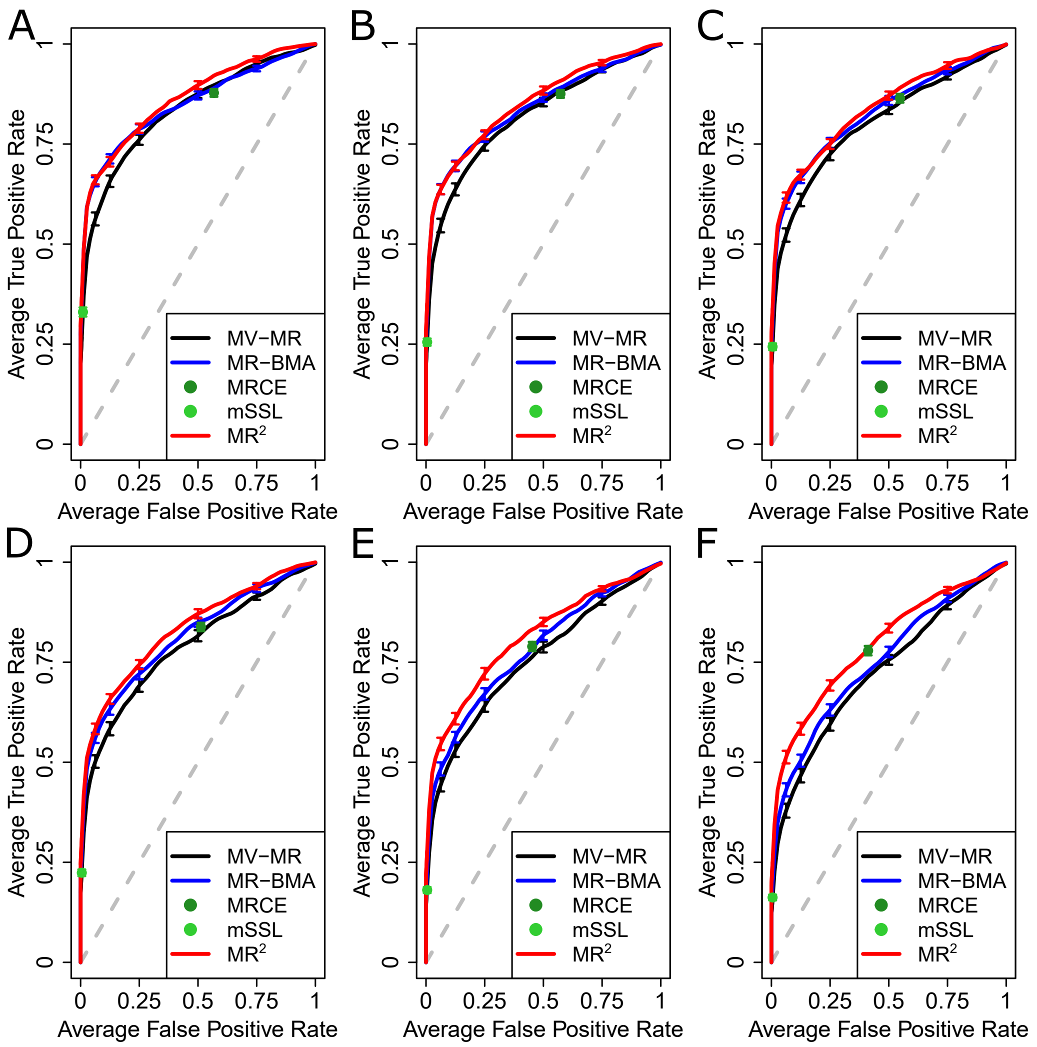

Figure S4. Simulation III-Undirected pleiotropy: Receiver operating characteristic (ROC) curves for different levels of the pleiotropic pathway effect θ^A and when the correlation between exposures is set at $r_X = 0.6$ and the number of individuals at the raw-level at $N = 20,000$ of which half is used to generate the summary-level genetic associations for the exposure and half for the outcomes, averaged over 50 replicates, illustrating the performance of different MR implementations and multi-response statistical methods to distinguish between true and false causal exposures for the simulated outcomes by plotting the true (TPR) against the false positive rate (FPR). Pleiotropic pathway effect varies from (**A**) to (**F**) with values $\theta^A = \{0.25, 0.5, 0.75, 1, 1.5, 2\}$. Confounding effects on the exposures and outcomes are fixed at $\theta_X^U = 2$ and $\theta_Y^U = 1$, respectively. Vertical bars in each ROC curve, at specific FPR levels, indicate standard error.

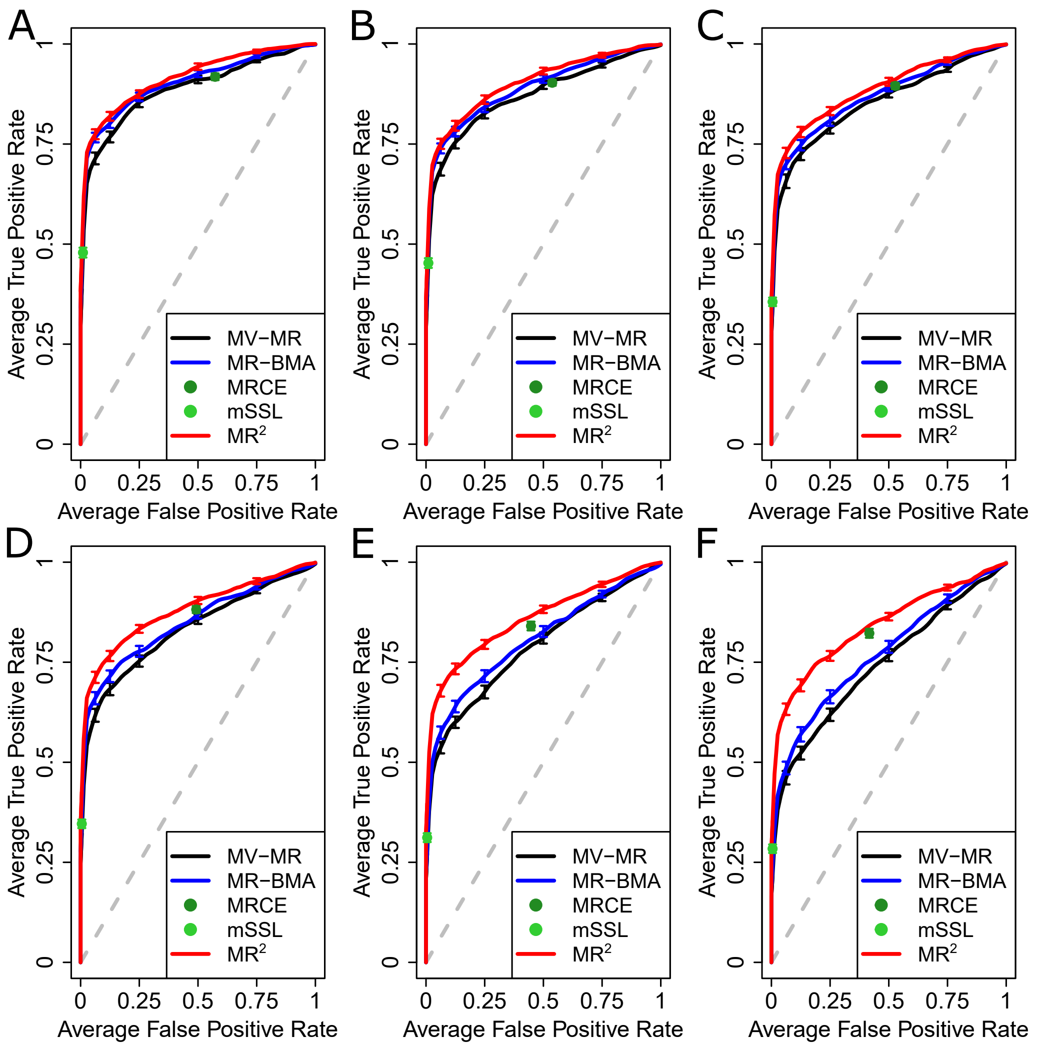

Figure S5. Simulation III-Undirected pleiotropy: Receiver operating characteristic (ROC) curves for different levels of the pleiotropic pathway effect $\boldsymbol{\theta^A}$ and when the correlation between exposures is set at $\boldsymbol{r_X = 0.6}$ and the heritability of the exposures at $\boldsymbol{h_X = 0.05}$, averaged over 50 replicates, illustrating the performance of different MR implementations and multi-response statistical methods to distinguish between true and false causal exposures for the simulated outcomes by plotting the true (TPR) against the false positive rate (FPR). Pleiotropic pathway effect varies from (**A**) to (**F**) with values $\theta^A = \{0.25, 0.5, 0.75, 1, 1.5, 2\}$. Confounding effects on the exposures and outcomes are fixed at $\theta^U_X = 2$ and $\theta^U_Y = 1$, respectively. Vertical bars in each ROC curve, at specific FPR levels, indicate standard error.

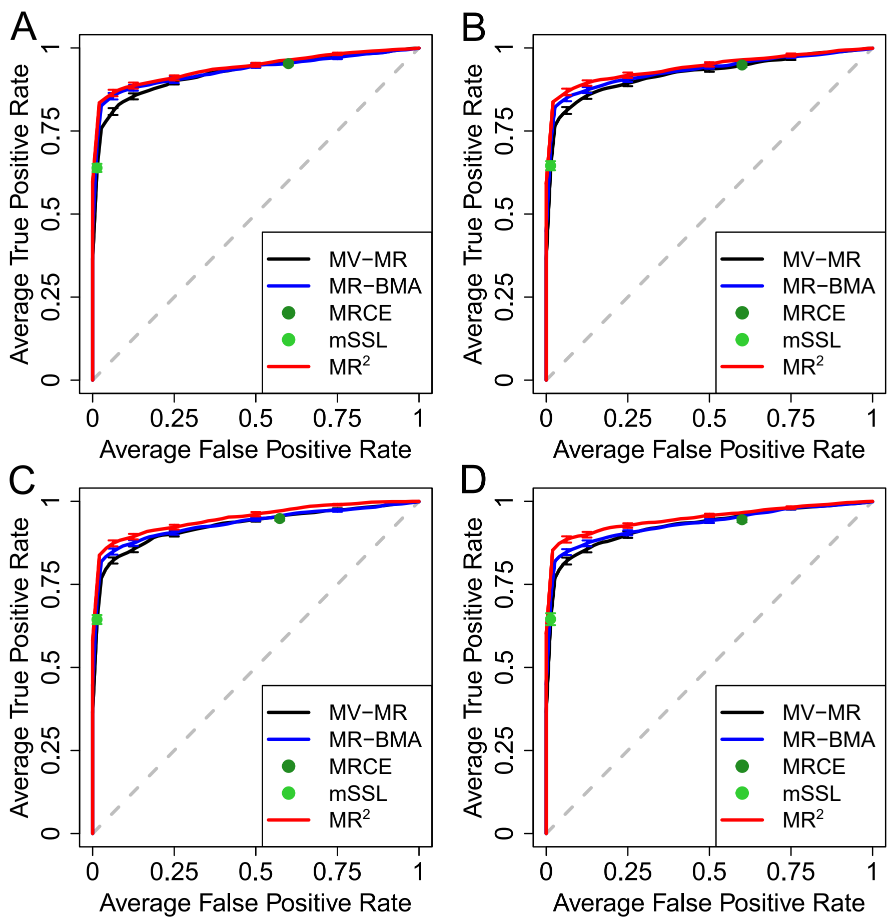

Figure S6. Simulation V-Dependence: Receiver operating characteristic (ROC) curves for different levels of the correlation between individual-level responses' errors, averaged over 50 replicates, illustrating the performance of different MR multivariable implementations and multi-response multivariable methods to distinguish between true and false causal exposures for the simulated outcomes by plotting the true (TPR) against the false positive rate (FPR). Individual-level correlation between the responses' errors varies from (**A**) to (**D**) with values $r_Y = \{0.2, 0.4, 0.6, 0.8\}$. Correlation between exposures is set at $r_X = 0.6$ and confounding effects on the exposures and outcomes are fixed at $\theta_X^U = 2$ and $\theta_Y^U = 1$, respectively. Vertical bars in each ROC curve, at specific FPR levels, indicate standard error.

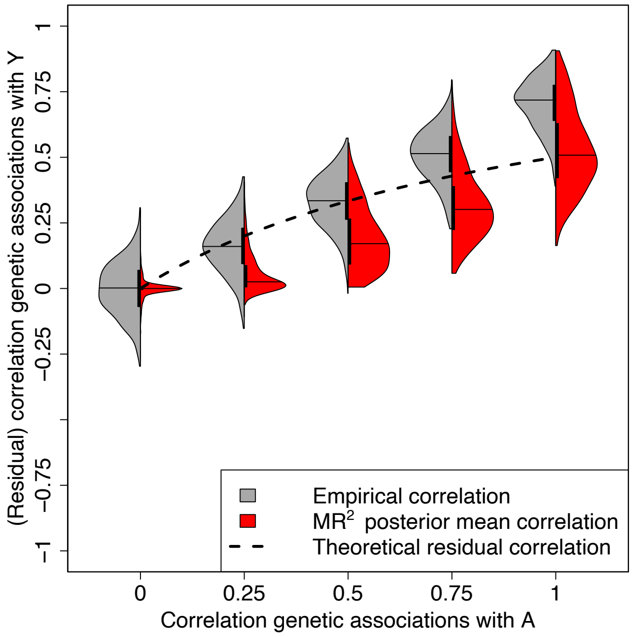

Figure S7. Residual correlation between the summary-level genetic associations with the outcomes induced by various levels of correlation of the genetic associations with the pleiotropic pathways A and with no sample overlap between the summary-level data of the outcomes. Each violin plot depicts the posterior mean of the residual correlations estimated by MR^2 in a simulation scenario similar to Scenario III-Undirected pleiotropy averaged over 50 replicates at different levels of the correlation ρ^A in eq. (S5) between the genetic associations with the pleiotropy pathways A while keeping $\theta^A = 1$ constant. The level of correlation is set at $\rho^A = \{0, 0.25, 0.50, 0.75, 0.99\}$. Confounding effects on the exposures and outcomes are fixed at $\theta_X^U = 2$ and $\theta_Y^U = 1$, respectively, without correlation between exposures and no sample overlap in the summary-level genetic associations with the outcomes. For each side of the violin plot, the vertical black thick line displays the interquartile range, while the black line indicates the median. Black dashed line depicts the theoretical value of residual correlation between summary-level outcomes as a function of ρ^A as shown in eq. (S4). For more details on the simulation setting, see Table S2.

S4 Application examples

S4.1 Data pre-processing

Application examples are based on publicly available summary-level data. An overview of the data sets, with sample sizes and references, is provided in Table S13.

The first application example considers ten common exposures for five cardiovascular disease outcomes (CVDs). The first step of the data-processing merges the summary-level data (beta regression coefficients, their standard errors and associated p-values) of all exposures by their unique "rs" identifier and aligns the effect direction of the genetic associations with each exposure according to the same effect allele. As IVs, we select any genetic variant which is associated with any of the exposures at genome-wide significance (p-value $< 5 \times 10^{-8}$). Next, we merge the genetic variants selected as IVs with the outcome data of the five CVDs by their unique "rs" identifier and aligned the effect direction of the genetic associations with each outcome according to the same effect allele. Finally, we clump the genetic variants to be independent at $r^2 < 0.001$ using a European reference panel [12]. This results in $n = 1,540$ independent genetic variants selected as IVs.

The second application example focuses on ten ApoB-containing lipoprotein subfractions derived from metabolic GWAS using the Nightingale nuclear magnetic resonance spectroscopy platform derived from fasting samples of $24,925$ European descent participants [13]. Instruments selection is based on an external data set. Specifically, given the prior hypothesis of ApoB as the leading exposure for CVDs, we select genetic variants associated with ApoB in UK Biobank at genome-wide significance (p-value $< 5 \times 10^{-8}$). Exposures and outcomes summary-level data are merged by their unique "rs" identifier and effect alleles are aligned. Finally, we clump the genetic variants to be independent at $r^2 < 0.001$ using a European reference panel [14]. This resulted in $n = 148$ IVs.

In both application examples, IVW is performed before the analysis for all outcomes and exposures using weights derived jointly from all responses as described in Appendix. Moreover, the summary-level genetic associations with the exposures are standardised before the analysis. This is usually required in (multi-response) multivariable regression models since the summary-level genetic associations with the exposures should be on the same scale in order to interpret and compare the estimated effect sizes.

S4.2 Analysis plan and comparisons with alternative methods

MR^2 parameters setting

In the first application example, the MR^2 algorithm is run for $75,000$ MCMC iterations of which $25,000$ as burn-in and results saved every 50 iterations, resulting in $1,000$ posterior samples for all the unknowns. In the second application example, we run the algorithm for $150,000$ MCMC iterations of which $50,000$ as burn-in with $1,000$ posterior samples saved. As illustrated in Figures S9 and S14, the burn-in phase and the total length of the MCMC far exceed the number of iterations required to reach convergence to the equilibrium distribution of the Markov chain and explore faithfully the posterior parameters space. No signs of slow convergence or aberrant behaviour of the designed MCMC sampler are also apparent.

In both application examples, the hyper-parameters a_k and b_k in eq. (15) are chosen by specifying $\mathbb{E}(\gamma_k) = 2$ and $\mathbb{V}(\gamma_k) = 2$ which imply *a priori* a range between zero and seven for the number of significant direct causal effects for each outcome. Sensitivity analysis shows that the *a priori* range can also be relaxed ($\mathbb{E}(\gamma_k) = 4$ and $\mathbb{V}(\gamma_k) = 4$) without noticeable differences in the posterior mean of the direct causal effects, see Figures S12 and S17. We also set $v = 1$ in eq. (13) after standardising the summary-level genetic associations with the IVW transformed exposures in order to place appropriate prior mass on reasonable values of the non-zero direct causal effects [8].

We use Congdon [15] definition of outliers or high-leverage and influential observations and report the number of standardised CPOs less than 0.01 to check model adequacy. After excluding genetic variants with standardised CPOs below this threshold, we refit the model without repeating the procedure even if new outliers or high-leverage and influential observations are detected, see Figures S10 and S15.

Finally, the FDR level is set at 5% to select important mPPIs and ePPIs, see Figures S11A and B and S16A and B.

MR alternative methods and parameters setting

MR-BMA is run for each outcome separately with a prior probability of inclusion of $2/10 = 0.2$ in application example 1, using ten common exposures for CVDs, as well as in application example 2, using ten molecular risk factors for the same disease outcomes, in order to match the sparsity prior used in MR^2. An exhaustive search evaluating all combinations of exposures in both applications is conducted and no stochastic search is necessary. Because

summary-level genetic associations with the exposures are standardised prior to the analysis, the prior variance on the direct causal effects is set at 1.

All results presented are generated after removing any influential or outlying genetic variant used as IV. Influential genetic variants are detected using the Cook's distance [12] and a cut-off is chosen according to the median of the F-distribution with d and $n - d$ degrees of freedom, where n is the number of IVs and d is the size of the best visited model in the exhaustive search.

Outlying genetic variants are identified using the q-heterogeneity statistic [12] and threshold using a χ^2 distribution with one degree of freedom and a Bonferroni-adjusted multiple testing correction. Empirical p-values for the marginal inclusion probabilities are calculated using a permutation procedure with $100,000$ permutations [14]. Multiple testing adjustment is performed using Benjamini-Hochberg [16] FDR and Bonferroni correction.

MV-MR [9] is applied on the summary-level data after removing outliers or influential variants and multiple testing adjustment is performed using Benjamini-Hochberg FDR and Bonferroni correction.

S4.3 Supplemental tables

	Response or risk factor	Acronym	Reference	Number cases & controls	Data source
Cardiovascular disease outcomes:	Atrial fibrillation	AF	Nielsen *et al.* 2018 [17]	60,620 cases & 970,216 controls	AFGen Cons., HUNT, DECODE, MGI, DiscovEHR + UK Biobank
	Cardioembolic stroke	CES	Mishra *et al.* 2022 [18]	10,804 cases & 1,234,808 controls	GIGASTROKE consortium + Global Biobanks (UK Biobank and Million Veteran Program)
	Coronary artery disease	CAD	Nelson *et al.* 2017 [19]	113,937 cases & 339,658 controls	CARDIOGRAM Cons. + UK Biobank
	Peripheral artery disease	PAD	Klarin *et al.* 2019 [20]	31,307 cases & 211,753 controls	Million Veteran Program
	Heart failure	HF	Shah *et al.* 2020 [21]	47,309 cases & 930,014 controls	HERMES Cons. + UK Biobank
Exposures in application example 1: Common exposures	Apolipoprotein A	ApoA	Nealelab 2018 [22]		UK Biobank
	Apolipoprotein B	ApoB			UK Biobank
	Body mass index	BMI			UK Biobank
	High-density lipoprotein	HDL			UK Biobank
	Low-density lipoprotein	LDL			UK Biobank
	Systolic blood pressure	SBP			UK Biobank
	Triglycerides	TG			UK Biobank
	Physical activity	PA	Wang *et al.* 2022 [23]	636,924 cases	UK Biobank
	Smoking	Smoking	Wootton *et al.* 2020 [24]	462,690 cases	UK Biobank
	Type 2 diabetes	T2D	Mahajan *et al.* 2018 [25]	74,124 cases & 824,006 controls	UK Biobank
Exposures in application example 2: Molecular risk factors	Medium large-density lipoprotein particles	M.LDL.P	Kettunen *et al.* 2016 [13]	24,925 controls	Meta-analysis of Finnish and European cohorts
	Large large-density lipoprotein particles	L.LDL.P			Meta-analysis of Finnish and European cohorts
	Intermediate-density lipoprotein particles	IDL.P			Meta-analysis of Finnish and European cohorts
	Extra small very-large density lipoprotein particles	XS.VLDL.P			Meta-analysis of Finnish and European cohorts
	Small very-large density lipoprotein particles	S.VLDL.P			Meta-analysis of Finnish and European cohorts
	Medium very-large density lipoprotein particles	M.VLDL.P			Meta-analysis of Finnish and European cohorts
	Large very-large density lipoprotein particles	L.VLDL.P			Meta-analysis of Finnish and European cohorts
	Extra large very-large density lipoprotein particles	XL.VLDL.P			Meta-analysis of Finnish and European cohorts
	Extra-extra large very-large density lipoprotein particles	XXL.VLDL.P			Meta-analysis of Finnish and European cohorts

Table S13. Overview of summary-level data in the two application examples. Common exposures (application example 1) and molecular risk factors (application example 2) for cardiovascular disease outcomes.

Outcome	Ranking	Exposure	Effect estimate	Standard error	p-value	FDR	Bonferroni
CAD	1	**SBP**	0.621	0.041	1.45×10^{-47}	1.45×10^{-46}	1.45×10^{-46}
	2	**T2D**	0.125	0.013	1.69×10^{-20}	8.43×10^{-20}	1.69×10^{-19}
	3	**SMOKING**	0.507	0.093	5.98×10^{-8}	1.99×10^{-7}	5.98×10^{-7}
	4	**BMI**	0.094	0.034	0.005	0.013	0.053
	5	TG	0.083	0.038	0.029	0.058	0.292
	6	LDL	0.251	0.136	0.065	0.105	0.650
	7	ApoA	-0.159	0.089	0.074	0.105	0.737
	8	ApoB	0.178	0.121	0.141	0.176	1.407
	9	HDL	0.084	0.094	0.372	0.414	3.723
	10	PA	0.001	0.050	0.978	0.978	9.779
PAD	1	**T2D**	0.161	0.014	7.91×10^{-30}	7.91×10^{-29}	7.91×10^{-29}
	2	**SMOKING**	0.789	0.098	1.30×10^{-15}	6.48×10^{-15}	1.30×10^{-14}
	3	**SBP**	0.326	0.043	7.81×10^{-14}	2.60×10^{-13}	7.81×10^{-13}
	4	**BMI**	0.155	0.035	1.16×10^{-5}	2.91×10^{-5}	1.16×10^{-4}
	5	TG	0.074	0.040	0.063	0.126	0.632
	6	ApoA	-0.130	0.093	0.163	0.271	1.626
	7	ApoB	0.123	0.127	0.334	0.477	3.340
	8	HDL	0.082	0.099	0.411	0.513	4.106
	9	LDL	0.035	0.143	0.807	0.818	8.067
	10	PA	0.012	0.053	0.818	0.818	8.182
HF	1	**BMI**	0.318	0.030	1.54×10^{-25}	1.54×10^{-24}	1.54×10^{-24}
	2	**SBP**	0.322	0.036	2.80×10^{-18}	1.40×10^{-17}	2.80×10^{-17}
	3	**T2D**	0.047	0.012	5.63×10^{-5}	1.88×10^{-4}	5.63×10^{-4}
	4	**SMOKING**	0.323	0.083	9.77×10^{-5}	2.44×10^{-4}	9.77×10^{-4}
	5	ApoB	0.113	0.108	0.296	0.584	2.958
	6	HDL	-0.072	0.084	0.390	0.584	3.904
	7	PA	0.037	0.045	0.409	0.584	4.088
	8	ApoA	0.048	0.079	0.540	0.674	5.396
	9	LDL	0.015	0.121	0.903	0.983	9.026
	10	TG	-0.001	0.034	0.983	0.983	9.829
AF	1	**SBP**	0.286	0.036	7.71×10^{-15}	7.71×10^{-14}	7.71×10^{-14}
	2	**BMI**	0.225	0.030	5.83×10^{-14}	2.92×10^{-13}	5.83×10^{-13}
	3	**TG**	-0.129	0.034	1.39×10^{-4}	4.62×10^{-4}	1.39×10^{-03}
	4	SMOKING	0.140	0.082	0.090	0.187	0.899
	5	ApoB	0.180	0.107	0.094	0.187	0.938
	6	HDL	-0.133	0.084	0.112	0.187	1.123
	7	ApoA	0.108	0.079	0.172	0.245	1.717
	8	LDL	-0.151	0.120	0.210	0.263	2.103
	9	T2D	-0.009	0.012	0.427	0.439	4.271
	10	PA	0.035	0.045	0.439	0.439	4.392
CES	1	**SBP**	0.266	0.063	2.76×10^{-5}	2.76×10^{-4}	2.76×10^{-4}
	2	**T2D**	0.064	0.020	0.001	0.007	0.015
	3	**SMOKING**	0.345	0.141	0.014	0.048	0.143
	4	TG	-0.117	0.058	0.044	0.111	0.444
	5	PA	0.088	0.077	0.252	0.467	2.517
	6	ApoB	0.199	0.184	0.280	0.467	2.803
	7	LDL	-0.177	0.208	0.395	0.564	3.948
	8	HDL	-0.056	0.144	0.698	0.867	6.976
	9	ApoA	0.038	0.135	0.780	0.867	7.804
	10	BMI	-0.001	0.051	0.979	0.979	9.792

Table S14. Application example 1 on common exposures for cardiovascular disease outcomes using standard multivariable MR (MV-MR) which is run on each outcome separately. Results include the causal effect estimates, the standard error and the corresponding p-value. We adjust for multiple testing using Benjamini-Hochberg False Discovery Rate (FDR) and Bonferroni correction. For each outcome, we sort the exposures by their p-value in increasing order. Exposures selected at 5% FDR highlighted in bold.

Outcome	Ranking	Exposure	Effect estimate	Standard error	p-value	FDR	Bonferroni
CAD		(Intercept)	-0.024	0.029	0.401	0.401	-
	1	**SBP**	0.621	0.041	1.53×10^{-47}	1.53×10^{-46}	1.53×10^{-46}
	2	**T2D**	0.125	0.013	1.46×10^{-20}	7.31×10^{-20}	1.46×10^{-19}
	3	**SMOKING**	0.506	0.093	6.37×10^{-8}	2.12×10^{-7}	6.37×10^{-7}
	4	**BMI**	0.094	0.034	0.005	0.013	0.051
	5	TG	0.083	0.038	0.030	0.059	0.296
	6	LDL	0.253	0.136	0.064	0.105	0.636
	7	ApoA	-0.159	0.089	0.073	0.105	0.732
	8	ApoB	0.177	0.121	0.144	0.180	1.000
	9	HDL	0.085	0.094	0.371	0.412	1.000
	10	PA	-0.001	0.050	0.985	0.985	1.000
PAD		(Intercept)	0.004	0.030	0.883	0.883	-
	1	**T2D**	0.161	0.014	8.79×10^{-30}	8.79×10^{-29}	8.79×10^{-29}
	2	**SMOKING**	0.790	0.098	1.31×10^{-15}	6.55×10^{-15}	1.31×10^{-14}
	3	**SBP**	0.326	0.043	7.92×10^{-14}	2.64×10^{-13}	7.92×10^{-13}
	4	**BMI**	0.155	0.035	1.19×10^{-5}	2.97×10^{-5}	1.19×10^{-4}
	5	TG	0.074	0.040	0.063	0.126	0.631
	6	ApoA	-0.130	0.093	0.163	0.271	1.000
	7	ApoB	0.123	0.127	0.333	0.476	1.000
	8	HDL	0.082	0.099	0.411	0.513	1.000
	9	LDL	0.035	0.143	0.808	0.812	1.000
	10	PA	0.013	0.053	0.812	0.812	1.000
HF		(Intercept)	0.000	0.025	0.990	0.990	-
	1	**BMI**	0.318	0.030	1.63×10^{-25}	1.63×10^{-24}	1.63×10^{-24}
	2	**SBP**	0.322	0.036	2.87×10^{-18}	1.44×10^{-17}	2.87×10^{-17}
	3	**T2D**	0.047	0.012	5.68×10^{-5}	1.89×10^{-4}	5.68×10^{-4}
	4	**SMOKING**	0.323	0.083	9.85×10^{-5}	2.46×10^{-4}	0.001
	5	ApoB	0.113	0.108	0.296	0.586	1.000
	6	HDL	-0.072	0.084	0.391	0.586	1.000
	7	PA	0.037	0.045	0.410	0.586	1.000
	8	ApoA	0.048	0.079	0.540	0.675	1.000
	9	LDL	0.015	0.121	0.903	0.983	1.000
	10	TG	-0.001	0.034	0.983	0.983	1.000
AF		(Intercept)	0.020	0.025	0.436	0.436	-
	1	**SBP**	0.286	0.036	7.62×10^{-15}	7.62×10^{-14}	7.62×10^{-14}
	2	**BMI**	0.225	0.030	6.65×10^{-14}	3.33×10^{-13}	6.65×10^{-13}
	3	**TG**	-0.129	0.034	1.43×10^{-4}	4.76×10^{-4}	0.001
	4	SMOKING	0.141	0.082	0.088	0.184	0.877
	5	ApoB	0.181	0.107	0.092	0.184	0.920
	6	HDL	-0.133	0.084	0.112	0.187	1.000
	7	ApoA	0.108	0.079	0.171	0.245	1.000
	8	LDL	-0.152	0.120	0.207	0.259	1.000
	9	T2D	-0.009	0.012	0.417	0.417	1.000
	10	PA	0.036	0.045	0.417	0.417	1.000
CES		**(Intercept)**	0.169	0.043	9.58×10^{-5}	9.58×10^{-5}	-
	1	**SBP**	0.265	0.063	2.56×10^{-5}	2.56×10^{-4}	2.56×10^{-4}
	2	**T2D**	0.062	0.020	0.002	0.009	0.019
	3	**SMOKING**	0.357	0.140	0.011	0.037	0.110
	4	TG	-0.116	0.058	0.044	0.109	0.438
	5	PA	0.106	0.077	0.168	0.336	1.000
	6	ApoB	0.204	0.183	0.265	0.441	1.000
	7	LDL	-0.181	0.207	0.384	0.548	1.000
	8	HDL	-0.060	0.143	0.675	0.844	1.000
	9	ApoA	0.041	0.134	0.760	0.844	1.000
	10	BMI	-0.004	0.051	0.936	0.936	1.000

Table S15. Application example 1 on common exposures for cardiovascular disease outcomes using multivariable MR-Egger which is run on each outcome separately. Results include the causal effect estimates as well as the intercept, the standard error and the corresponding p-value. We adjust for multiple testing using Benjamini-Hochberg False Discovery Rate (FDR) and Bonferroni correction. For each outcome, we sort the exposures by their p-value in increasing order. Exposures selected at 5% FDR highlighted in bold.

Outcome	Ranking	Exposure	MACE	mPPI	Empirical p-value	FDR	Bonferroni
CAD	1	**SBP**	0.626	1.000	1.00×10^{-5}	3.33×10^{-5}	1.00×10^{-4}
	2	**SMOKING**	0.560	1.000	1.00×10^{-5}	3.33×10^{-5}	1.00×10^{-4}
	3	**T2D**	0.137	1.000	1.00×10^{-5}	3.33×10^{-5}	1.00×10^{-4}
	4	**ApoB**	0.285	0.659	0.001	0.002	0.007
	5	**HDL**	-0.034	0.295	0.017	0.032	0.171
	6	**LDL**	0.161	0.344	0.022	0.032	0.219
	7	**BMI**	0.047	0.409	0.022	0.032	0.221
	8	ApoA	-0.020	0.185	0.062	0.078	0.624
	9	TG	0.002	0.033	0.902	1.000	1.000
	10	PA	0.000	0.002	1.000	1.000	1.000
PAD	1	**SMOKING**	0.787	1.000	1.00×10^{-5}	2.00×10^{-5}	1.00×10^{-4}
	2	**SBP**	0.325	1.000	1.00×10^{-5}	2.00×10^{-5}	1.00×10^{-4}
	3	**ApoB**	0.184	0.975	1.00×10^{-5}	2.00×10^{-5}	1.00×10^{-4}
	4	**BMI**	0.174	0.998	1.00×10^{-5}	2.00×10^{-5}	1.00×10^{-4}
	5	**T2D**	0.171	1.000	1.00×10^{-5}	2.00×10^{-5}	1.00×10^{-4}
	6	LDL	0.005	0.025	0.978	1.000	1.000
	7	TG	0.000	0.000	1.000	1.000	1.000
	8	PA	0.000	0.000	1.000	1.000	1.000
	9	ApoA	0.000	0.001	1.000	1.000	1.000
	10	HDL	0.000	0.001	1.000	1.000	1.000
HF	1	**SBP**	0.324	1.000	1.00×10^{-5}	5.00×10^{-5}	1.00×10^{-4}
	2	**BMI**	0.324	1.000	1.00×10^{-5}	5.00×10^{-5}	1.00×10^{-4}
	3	**ApoB**	0.117	0.897	2.00×10^{-5}	6.67×10^{-5}	2.00×10^{-4}
	4	**T2D**	0.052	0.993	4.00×10^{-5}	1.00×10^{-4}	4.00×10^{-4}
	5	**SMOKING**	0.323	0.991	2.90×10^{-4}	0.001	0.003
	6	LDL	0.014	0.104	0.267	0.445	1.000
	7	TG	0.000	0.001	1.000	1.000	1.000
	8	PA	0.000	0.000	1.000	1.000	1.000
	9	ApoA	0.000	0.000	1.000	1.000	1.000
	10	HDL	0.000	0.001	1.000	1.000	1.000
AF	1	**SBP**	0.281	1.000	1.00×10^{-5}	5.00×10^{-5}	1.00×10^{-4}
	2	**BMI**	0.233	1.000	1.00×10^{-5}	5.00×10^{-5}	1.00×10^{-4}
	3	**TG**	-0.035	0.520	0.009	0.031	0.094
	4	HDL	-0.002	0.048	0.700	0.995	1.000
	5	SMOKING	0.018	0.132	0.870	0.995	1.000
	6	ApoB	0.003	0.040	0.878	0.995	1.000
	7	ApoA	-0.001	0.030	0.923	0.995	1.000
	8	LDL	-0.002	0.027	0.968	0.995	1.000
	9	T2D	0.000	0.008	0.988	0.995	1.000
	10	PA	0.001	0.028	0.995	0.995	1.000
CES	1	**SBP**	0.295	1.000	1.00×10^{-5}	5.00×10^{-5}	1.00×10^{-4}
	2	**BMI**	0.217	1.000	1.00×10^{-5}	5.00×10^{-5}	1.00×10^{-4}
	3	TG	-0.013	0.232	0.048	0.160	0.481
	4	SMOKING	0.041	0.244	0.381	0.953	1.000
	5	HDL	-0.001	0.025	0.947	0.990	1.000
	6	ApoA	-0.001	0.024	0.960	0.990	1.000
	7	ApoB	0.002	0.024	0.970	0.990	1.000
	8	PA	0.002	0.040	0.978	0.990	1.000
	9	T2D	0.000	0.010	0.979	0.990	1.000
	10	LDL	-0.001	0.020	0.990	0.990	1.000

Table S16. Application example 1 on common exposures for cardiovascular disease outcomes using the Mendelian randomisation Bayesian model averaging (MR-BMA) algorithm which is run on each outcome separately. Results include the model-averaged causal effect estimate (MACE), the marginal posterior probability of inclusion (mPPI) and the corresponding empirical p-value [12]. We adjust for multiple testing using Benjamini-Hochberg False Discovery Rate (FDR) and Bonferroni correction. For each outcome, we sort the exposures by their empirical p-value in increasing order and the absolute value of MACE in decreasing order. Exposures selected at 5% FDR highlighted in bold.

Outcome	Ranking	Exposure	Effect estimate	Standard error	p-value	FDR	Bonferroni
CAD	1	M.VLDL.P	0.354	0.289	0.224	0.853	1.000
	2	S.LDL.P	-0.519	0.549	0.347	0.853	1.000
	3	M.LDL.P	1.550	1.727	0.371	0.853	1.000
	4	S.VLDL.P	-0.239	0.340	0.483	0.853	1.000
	5	L.LDL.P	-1.096	2.207	0.620	0.853	1.000
	6	IDL.P	0.541	1.329	0.685	0.853	1.000
	7	L.VLDL.P	-0.120	0.322	0.709	0.853	1.000
	8	XL.VLDL.P	-0.068	0.212	0.749	0.853	1.000
	9	XXL.VLDL.P	-0.048	0.184	0.795	0.853	1.000
	10	XS.VLDL.P	0.105	0.566	0.853	0.853	1.000
PAD	1	XS.VLDL.P	0.727	0.469	0.124	0.632	1.000
	2	S.LDL.P	-0.690	0.533	0.198	0.632	1.000
	3	S.VLDL.P	-0.384	0.305	0.210	0.632	1.000
	4	XL.VLDL.P	-0.240	0.209	0.253	0.632	1.000
	5	XXL.VLDL.P	0.172	0.184	0.352	0.703	1.000
	6	M.LDL.P	1.164	1.666	0.486	0.810	1.000
	7	IDL.P	-0.636	1.156	0.583	0.812	1.000
	8	M.VLDL.P	0.126	0.276	0.649	0.812	1.000
	9	L.VLDL.P	0.078	0.309	0.801	0.890	1.000
	10	L.LDL.P	-0.076	2.065	0.971	0.971	1.000
HF	1	M.LDL.P	3.620	1.478	0.016	0.156	0.156
	2	L.LDL.P	-3.783	1.856	0.044	0.158	0.435
	3	S.VLDL.P	-0.550	0.275	0.047	0.158	0.474
	4	S.LDL.P	-0.867	0.468	0.066	0.166	0.664
	5	M.VLDL.P	0.390	0.249	0.120	0.240	1.000
	6	XXL.VLDL.P	0.218	0.165	0.189	0.287	1.000
	7	IDL.P	1.344	1.046	0.201	0.287	1.000
	8	L.VLDL.P	-0.209	0.279	0.456	0.562	1.000
	9	XL.VLDL.P	-0.125	0.188	0.506	0.562	1.000
	10	XS.VLDL.P	0.238	0.423	0.574	0.574	1.000
AF	1	L.VLDL.P	-0.417	0.250	0.097	0.945	0.968
	2	XXL.VLDL.P	0.157	0.140	0.263	0.945	1.000
	3	M.VLDL.P	0.172	0.220	0.438	0.945	1.000
	4	XL.VLDL.P	-0.093	0.157	0.556	0.945	1.000
	5	XS.VLDL.P	0.209	0.374	0.578	0.945	1.000
	6	IDL.P	-0.448	0.902	0.620	0.945	1.000
	7	S.VLDL.P	0.086	0.247	0.730	0.945	1.000
	8	L.LDL.P	0.401	1.572	0.799	0.945	1.000
	9	M.LDL.P	-0.131	1.262	0.917	0.945	1.000
	10	S.LDL.P	0.029	0.414	0.945	0.945	1.000
CES	1	IDL.P	-3.923	1.654	0.019	0.142	0.191
	2	L.LDL.P	6.416	2.894	0.028	0.142	0.283
	3	M.LDL.P	-4.059	2.311	0.081	0.271	0.813
	4	XS.VLDL.P	0.928	0.673	0.170	0.426	1.000
	5	S.VLDL.P	0.541	0.440	0.220	0.440	1.000
	6	XXL.VLDL.P	0.223	0.249	0.372	0.541	1.000
	7	L.VLDL.P	-0.392	0.443	0.378	0.541	1.000
	8	M.VLDL.P	-0.264	0.391	0.500	0.625	1.000
	9	S.LDL.P	0.406	0.742	0.585	0.650	1.000
	10	XL.VLDL.P	0.046	0.286	0.874	0.874	1.000

Table S17. Application example 2 on molecular risk factors for cardiovascular disease outcomes using standard multivariable MR (MV-MR) which is run on each outcome separately. Results presented include the causal effect estimates, the standard error and the corresponding p-value. We adjusted for multiple testing using Benjamini-Hochberg False Discovery Rate (FDR) and Bonferroni correction. For each outcome, we sort the exposures by their p-value in increasing order. No exposures are selected at 5% FDR.

Outcome	Ranking	Exposure	Effect estimate	Standard error	p-value	FDR	Bonferroni
CAD		(Intercept)	-0.031	0.112	0.781	0.781	-
	1	M.VLDL.P	0.348	0.291	0.235	0.860	1.000
	2	S.LDL.P	-0.511	0.552	0.356	0.860	1.000
	3	M.LDL.P	1.558	1.733	0.371	0.860	1.000
	4	S.VLDL.P	-0.235	0.341	0.493	0.860	1.000
	5	L.LDL.P	-1.121	2.217	0.614	0.860	1.000
	6	IDL.P	0.555	1.335	0.678	0.860	1.000
	7	L.VLDL.P	-0.119	0.324	0.715	0.860	1.000
	8	XL.VLDL.P	-0.071	0.213	0.739	0.860	1.000
	9	XXL.VLDL.P	-0.047	0.185	0.799	0.860	1.000
	10	XS.VLDL.P	0.100	0.568	0.860	0.860	1.000
PAD		(Intercept)	0.085	0.107	0.426	0.426	-
	1	XS.VLDL.P	0.767	0.473	0.107	0.615	1.000
	2	S.LDL.P	-0.724	0.535	0.179	0.615	1.000
	3	S.VLDL.P	-0.409	0.307	0.185	0.615	1.000
	4	XL.VLDL.P	-0.232	0.210	0.270	0.675	1.000
	5	XXL.VLDL.P	0.174	0.185	0.349	0.698	1.000
	6	M.LDL.P	1.162	1.668	0.487	0.769	1.000
	7	IDL.P	-0.717	1.162	0.539	0.769	1.000
	8	M.VLDL.P	0.137	0.277	0.623	0.778	1.000
	9	L.VLDL.P	0.078	0.309	0.801	0.890	1.000
	10	L.LDL.P	0.015	2.071	0.994	0.994	1.000
HF		(Intercept)	-0.126	0.095	0.186	0.186	-
	1	M.LDL.P	3.673	1.474	0.014	0.139	0.139
	2	L.LDL.P	-3.961	1.855	0.035	0.173	0.346
	3	S.VLDL.P	-0.515	0.275	0.064	0.196	0.637
	4	S.LDL.P	-0.830	0.468	0.078	0.196	0.784
	5	M.VLDL.P	0.371	0.249	0.138	0.271	1.000
	6	IDL.P	1.471	1.047	0.163	0.271	1.000
	7	XXL.VLDL.P	0.215	0.165	0.195	0.279	1.000
	8	L.VLDL.P	-0.208	0.279	0.458	0.531	1.000
	9	XL.VLDL.P	-0.133	0.188	0.478	0.531	1.000
	10	XS.VLDL.P	0.179	0.424	0.674	0.674	1.000
AF		(Intercept)	0.070	0.085	0.417	0.417	-
	1	L.VLDL.P	-0.415	0.250	0.099	0.987	0.994
	2	XXL.VLDL.P	0.156	0.140	0.266	0.987	1.000
	3	M.VLDL.P	0.180	0.221	0.418	0.987	1.000
	4	XS.VLDL.P	0.232	0.376	0.538	0.987	1.000
	5	IDL.P	-0.491	0.905	0.588	0.987	1.000
	6	XL.VLDL.P	-0.085	0.157	0.592	0.987	1.000
	7	L.LDL.P	0.442	1.575	0.780	0.989	1.000
	8	S.VLDL.P	0.066	0.249	0.791	0.989	1.000
	9	M.LDL.P	-0.112	1.264	0.929	0.997	1.000
	10	S.LDL.P	-0.002	0.416	0.997	0.997	1.000
CES		(Intercept)	0.286	0.151	0.060	0.060	-
	1	IDL.P	-4.151	1.643	0.013	0.109	0.127
	2	L.LDL.P	6.657	2.870	0.022	0.109	0.219
	3	M.LDL.P	-4.028	2.289	0.081	0.269	0.807
	4	XS.VLDL.P	1.044	0.669	0.121	0.303	1.000
	5	S.VLDL.P	0.462	0.437	0.293	0.554	1.000
	6	XXL.VLDL.P	0.220	0.246	0.373	0.554	1.000
	7	L.VLDL.P	-0.381	0.439	0.388	0.554	1.000
	8	M.VLDL.P	-0.234	0.387	0.547	0.684	1.000
	9	S.LDL.P	0.292	0.737	0.692	0.769	1.000
	10	XL.VLDL.P	0.072	0.284	0.801	0.801	1.000

Table S18. Application example 2 on molecular risk factors for cardiovascular disease outcomes using multivariable MR-Egger which is run on each outcome separately. Results include the causal effect estimates as well as the intercept, the standard error and the corresponding p-value. We adjust for multiple testing using Benjamini-Hochberg False Discovery Rate (FDR) and Bonferroni correction. For each outcome, we sort the exposures by their p-value in increasing order. No molecular exposures are selected at 5% FDR.

Outcome	Ranking	Exposure	MACE	mPPI	Empirical p-value	FDR	Bonferroni
CAD	1	**L.LDL.P**	0.300	0.689	3.00×10^{-5}	3.00×10^{-4}	3.00×10^{-4}
	2	IDL.P	0.094	0.291	0.013	0.065	0.130
	3	M.LDL.P	0.063	0.212	0.055	0.184	0.553
	4	XS.VLDL.P	0.010	0.093	0.491	0.999	1.000
	5	S.LDL.P	-0.011	0.091	0.846	0.999	1.000
	6	M.VLDL.P	0.008	0.055	0.985	0.999	1.000
	7	L.VLDL.P	-0.003	0.040	0.997	0.999	1.000
	8	S.VLDL.P	0.004	0.049	0.997	0.999	1.000
	9	XL.VLDL.P	-0.004	0.041	0.997	0.999	1.000
	10	XXL.VLDL.P	-0.002	0.035	0.999	0.999	1.000
PAD	1	**XS.VLDL.P**	0.192	0.840	9.00×10^{-5}	0.001	0.001
	2	IDL.P	0.026	0.149	0.154	0.772	1.000
	3	XL.VLDL.P	-0.015	0.106	0.888	0.995	1.000
	4	L.LDL.P	0.004	0.063	0.938	0.995	1.000
	5	M.LDL.P	0.004	0.054	0.972	0.995	1.000
	6	S.VLDL.P	0.004	0.065	0.986	0.995	1.000
	7	L.VLDL.P	-0.003	0.056	0.988	0.995	1.000
	8	S.LDL.P	-0.002	0.044	0.992	0.995	1.000
	9	M.VLDL.P	0.003	0.049	0.994	0.995	1.000
	10	XXL.VLDL.P	-0.001	0.049	0.995	0.995	1.000
HF	1	**XS.VLDL.P**	0.190	0.881	1.00×10^{-5}	1.00×10^{-4}	1.00×10^{-4}
	2	IDL.P	0.018	0.118	0.336	0.997	1.000
	3	L.LDL.P	0.001	0.054	0.964	0.997	1.000
	4	M.LDL.P	0.004	0.051	0.977	0.997	1.000
	5	S.LDL.P	0.002	0.044	0.991	0.997	1.000
	6	M.VLDL.P	0.005	0.047	0.993	0.997	1.000
	7	XXL.VLDL.P	0.005	0.049	0.993	0.997	1.000
	8	XL.VLDL.P	-0.005	0.050	0.993	0.997	1.000
	9	L.VLDL.P	-0.003	0.041	0.997	0.997	1.000
	10	S.VLDL.P	0.001	0.050	0.997	0.997	1.000
AF	1	**S.VLDL.P**	0.071	0.433	0.002	0.016	0.023
	2	**L.VLDL.P**	-0.122	0.473	0.003	0.016	0.031
	3	XL.VLDL.P	-0.041	0.274	0.044	0.135	0.442
	4	M.VLDL.P	0.045	0.220	0.054	0.135	0.539
	5	XS.VLDL.P	0.009	0.132	0.147	0.293	1.000
	6	IDL.P	0.005	0.108	0.368	0.614	1.000
	7	L.LDL.P	0.007	0.108	0.476	0.681	1.000
	8	M.LDL.P	0.008	0.103	0.646	0.807	1.000
	9	S.LDL.P	-0.005	0.093	0.785	0.872	1.000
	10	XXL.VLDL.P	0.001	0.089	0.918	0.918	1.000
CES	1	XXL.VLDL.P	-0.010	0.170	0.273	0.639	1.000
	2	L.VLDL.P	-0.011	0.149	0.336	0.639	1.000
	3	S.VLDL.P	0.006	0.109	0.369	0.639	1.000
	4	XS.VLDL.P	-0.003	0.095	0.419	0.639	1.000
	5	IDL.P	-0.005	0.100	0.461	0.639	1.000
	6	M.VLDL.P	0.001	0.124	0.478	0.639	1.000
	7	XL.VLDL.P	-0.003	0.139	0.509	0.639	1.000
	8	M.LDL.P	0.006	0.104	0.615	0.639	1.000
	9	L.LDL.P	0.000	0.099	0.618	0.639	1.000
	10	S.LDL.P	-0.004	0.102	0.639	0.639	1.000

Table S19. Application example 2 on molecular risk factors for cardiovascular disease outcomes using the Mendelian randomisation Bayesian model averaging (MR-BMA) algorithm wich is run on each outcome separately. Results include the model-averaged causal effect estimate (MACE), the marginal posterior probability of inclusion (mPPI) and the corresponding empirical p-value [12]. We adjust for multiple testing using Benjamini-Hochberg False Discovery Rate (FDR) and Bonferroni correction. For each outcome, we sort the exposures by their empirical p-value in increasing order and the absolute value of MACE in decreasing order. Exposures selected at 5% FDR highlighted in bold.

S4.4 Supplemental figures

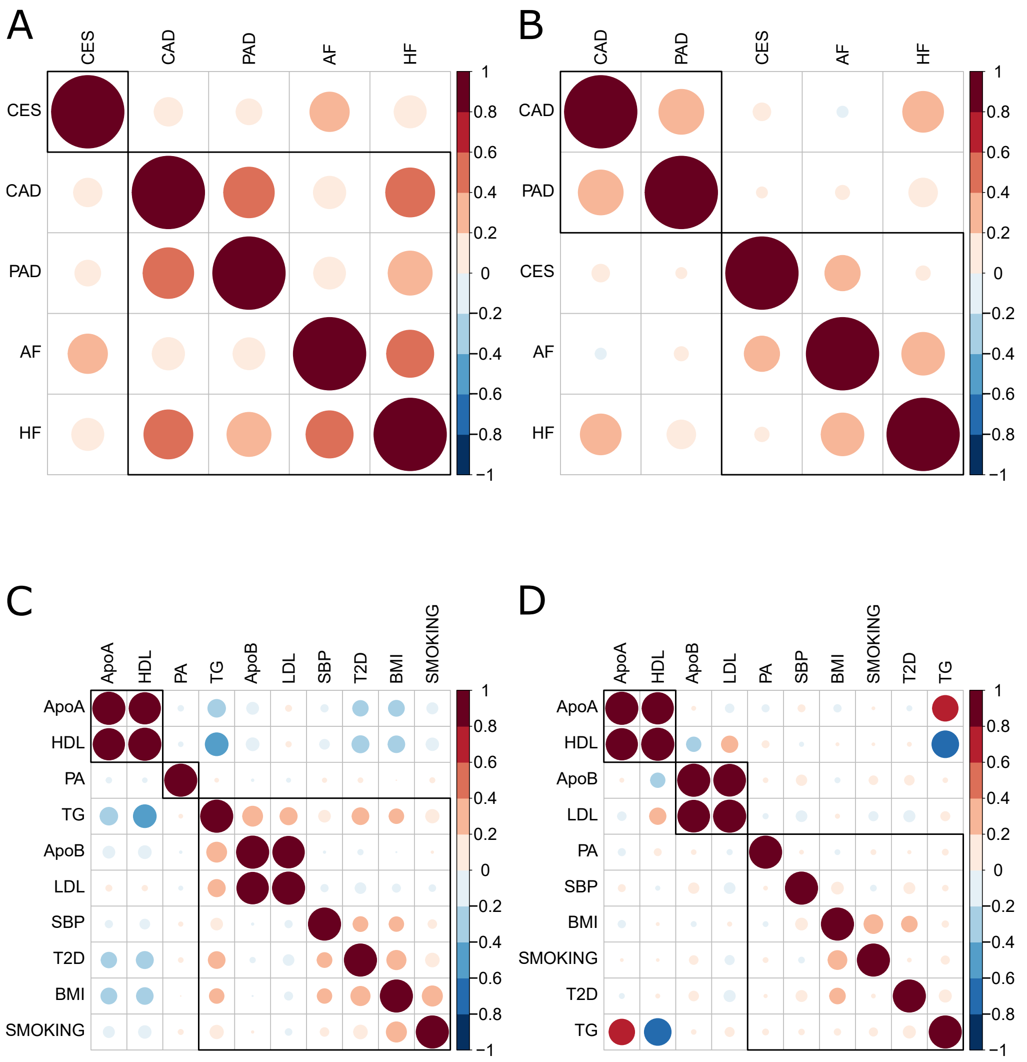

Figure S8. Correlation and partial correlation between summary-level outcomes and between summary-level exposures in application example 1 on common exposures for cardiovascular disease outcomes. Black boxes indicate clusters obtained by using hierarchical clustering with average agglomeration method, where the optimal number of clusters is estimated using `NbClust` R package. (**A**) Correlation between responses based on 1, 533 independent genetic variants associated with any of the ten exposures after removing seven IVs identified as outliers or high-leverage and influential observations (see Figure S10A). (**B**) Partial correlation between responses based on the same IVs. (**C**) Correlation between summary-level exposures based on 1, 533 independent genetic variants associated with any of the ten exposures. (**D**) Partial correlation between summary-level exposures based on the same IVs.

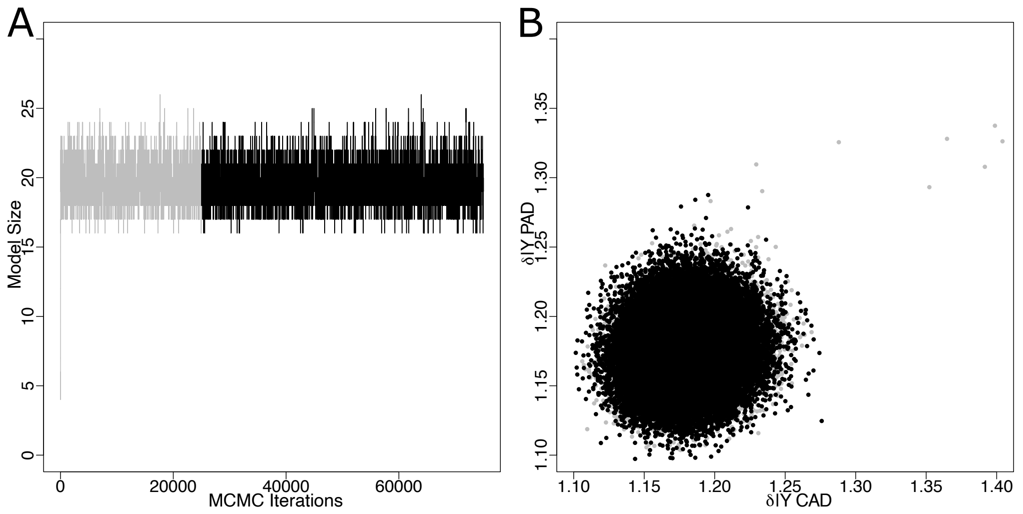

Figure S9. Convergence diagnostics of the multi-response MR^2 model in application example 1 on common exposures for cardiovascular disease outcomes (CVDs)(A) Trace plot of the number of non-zeros direct casual effects estimated during 75K MCMC iterations. Grey colour indicates burn-in phase up to 25K iterations, while black colour shows samples drawn after the burn-in. The designed MCMC algorithm reaches convergence after few iterations. (**B**) Scatterplot of the posterior samples of the square root of the error variance $\delta_k|Y$ for CAD and PAD. Grey colour indicates burn-in phase up to 25K iterations, while black colour shows samples drawn after the burn-in. After a few iterations (top right corner) the algorithm samples from the equilibrium distribution of the Markov chain.

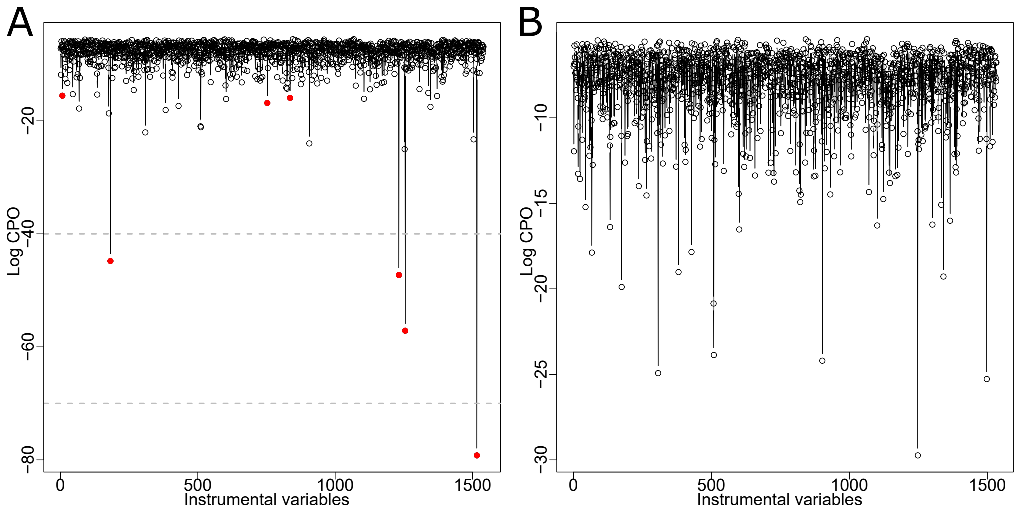

Figure S10. Outliers, high-leverage and influential observations detection using the multi-response MR^2 model in application example 1 on common exposures for cardiovascular disease outcomes (CVDs), plotting the log-conditional predictive ordinate (CPO) (y-axis) against 1.540 instrumental variables (x-axis). Red dots indicate observations with scaled CPOs below 0.01 to be regarded as outliers, high-leverage and influential observations [15]. We also show log-inverse-CPOs larger than 40 (possible outliers) and higher than 70 (extreme values) [26]. (**A**) Log-CPO for each instrumental variable and outliers, high-leverage and influential observations detected in the original summary-level data set of common exposures for CVDs. Seven IVs (0.5%) have scaled CPOs below 0.01, so the MR^2 model is considered to fit adequately. (**B**) Log-CPOs after removing outliers, high-leverage and influential observations from the original data set. No outliers or high-leverage and influential observations are detected.

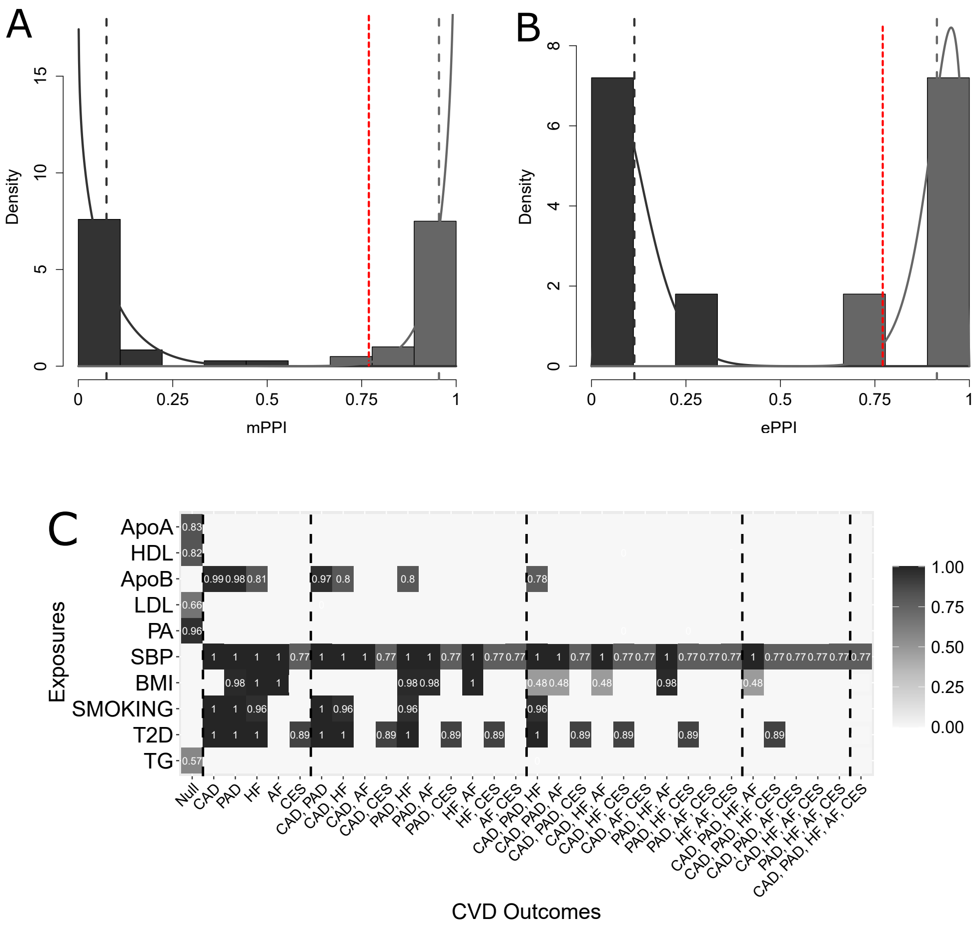

Figure S11. Post-processing results of the multi-response MR^2 model in application example 1 on common exposures for cardiovascular disease outcomes. (**A**) Non-parametric False Discovery Rate (FDR) strategy based on two-component mixture model which clusters low and high levels of the marginal posterior probabilities of inclusion (mPPIs). Dark grey colour code indicates mPPIs and the estimated density of the null (H_0) component, while light grey is used for the alternative (H_1) component. Dark grey and light grey dotted vertical lines shows the means of the beta components with a good separation between them, while red dotted vertical line specifies the threshold on the mPPI scale (mPPI ≥ 0.77) that corresponds to 5% FDR nominal level. (**B**) Non-parametric FDR strategy based on two-component mixture model which clusters low and high levels of the edge posterior probabilities of inclusion (ePPIs). Red dotted vertical line specifies the threshold on the ePPI scale (ePPI ≥ 0.78) that corresponds to 5% FDR nominal level. (**C**) Marginal and joint posterior probability of inclusion (jPPI) of each exposure (y-axis) against a single response or combinations of them (x-axis). Black dotted vertical lines separate combinations of outcomes with the same group size, *i.e.*, singletons, all possible pairs, all possible triplets, etc. For clarity of presentation, mPPIs for non-selected outcome-exposure pairs (mPPI < 0.77 at 5% FDR) are not plotted as well as jPPIs for combination of exposures marginally not selected. The "Null" vertical bar on the left indicates the posterior model probability of the null model.

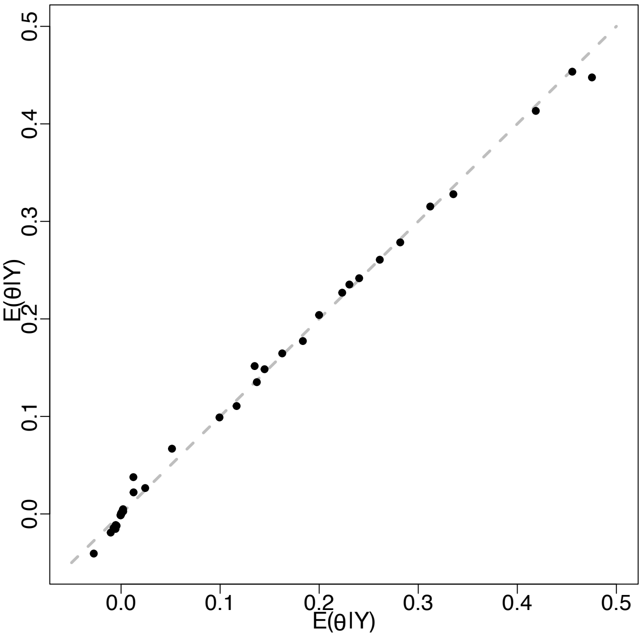

Figure S12. Sensitivity analysis of MR^2 with respected the *a priori* range of important exposures in application example 1 on common exposures for cardiovascular disease outcomes (CVDs). Robustness of the proposed method with respect to the level of sparsity controlled by $\mathbb{E}(\gamma_k)$ and $\mathbb{V}(\gamma_k)$, the *a priori* average number of important exposures associated with each response and its variance. In the original analysis, we fixed these values at $\mathbb{E}(\gamma_k) = 2$ and $\mathbb{V}(\gamma_k) = 2$. This prior choice implies *a priori* a range between zero and seven for the number of significant direct causal effects for each outcome. We changed them to $\mathbb{E}(\gamma_k) = 4$ and $\mathbb{V}(\gamma_k) = 4$ with *a priori* range between zero and 10. Scatterplot of the posterior mean of the direct causal effects in the original prior specification (x-axis) and the new specification (y-axis) shows no appreciable differences.

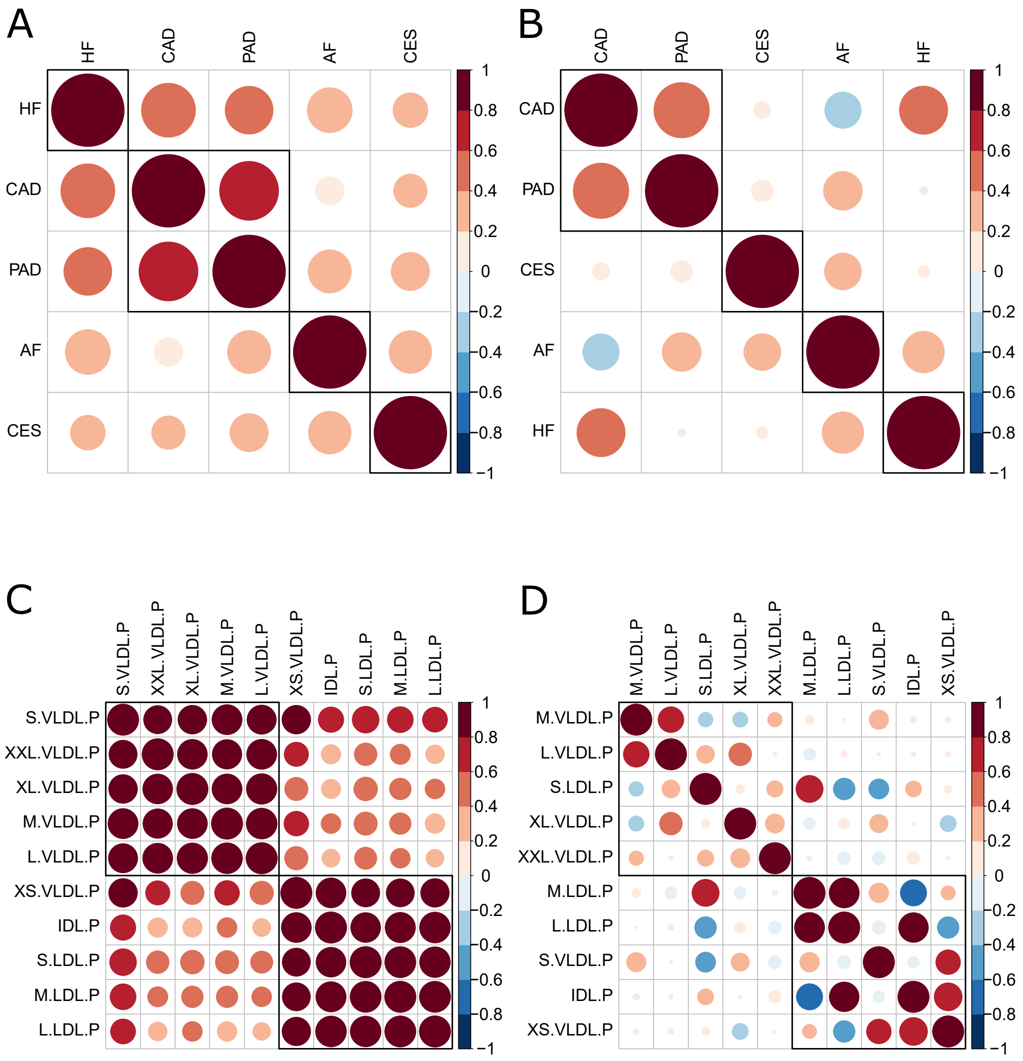

Figure S13. Correlation and partial correlation between summary-level outcomes and between summary-level exposures in application example 2 on molecular exposures for cardiovascular disease outcomes. Black boxes indicate clusters obtained by using hierarchical clustering with average agglomeration method where the optimal number of clusters was estimated using `NbClust` R package. (**A**) Correlation between responses based on 140 independent genetic variants associated with any of the ten molecular exposures after removing eight IVS identified as outliers or high-leverage and influential observations (see Figure S15A). (**B**) Partial correlation between summary-level responses based on the same IVs. (**C**) Correlation between risk factors based on 140 independent genetic variants associated with any of the ten molecular exposures. (**D**) Partial correlation between summary-level risk factors based on the same IVs.

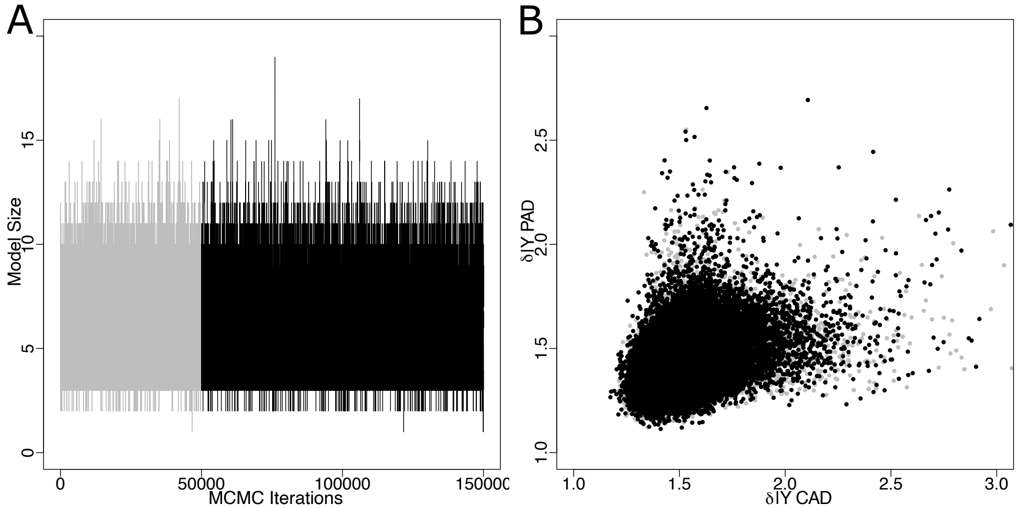

Figure S14. Convergence diagnostics of the multi-response MR^2 model in application example 2 on molecular risk factors for cardiovascular disease outcomes (CVDs)(A) Trace plot of the number of non-zeros direct casual effects estimated during 150K MCMC iterations. Grey colour indicates burn-in phase up to 50K iterations, while black colour shows samples drawn after the burn-in. The designed MCMC algorithm reaches convergence after few iterations. (**B**) Scatterplot of the posterior samples of the square root of the error variance $\delta_k|Y$ for CAD and PAD. Grey colour indicates burn-in phase up to 50K iterations, while black colour shows samples drawn after the burn-in. After a few iterations, the algorithm samples from the equilibrium distribution of the Markov chain. Since fewer exposures are selected, MR^2 samples larger error variances than in application example 1.

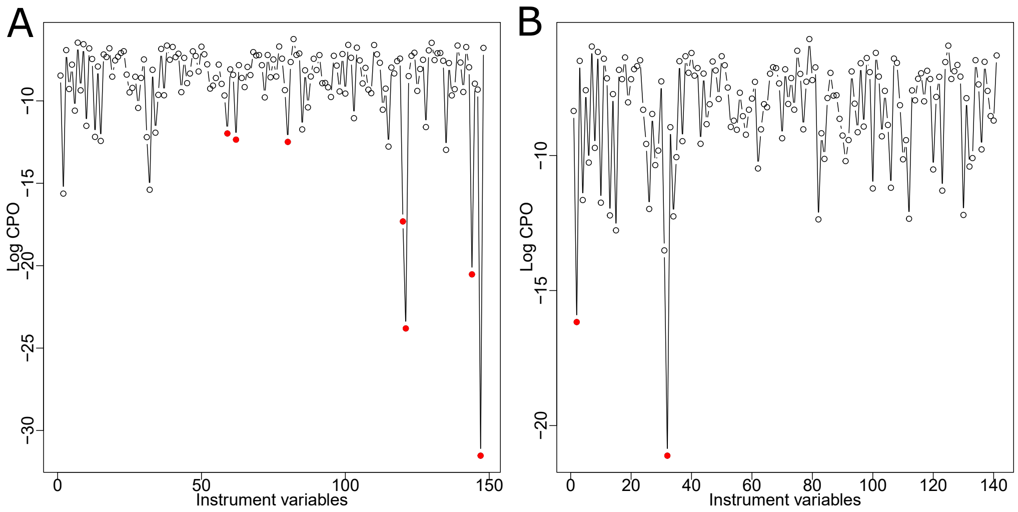

Figure S15. Outliers, high-leverage and influential observations detection using the multi-response MR^2 model in application example 2 on molecular risk factors for cardiovascular disease outcomes (CVDs), plotting the log-conditional predictive ordinate (CPO) (y-axis) against 148 instrumental variables (x-axis). Red dots indicate observations with scaled CPOs below 0.01 to be regarded as outliers, high-leverage and influential observations [15]. (**A**) Log-CPO for each instrumental variable and outliers, high-leverage and influential observations detected in the original summary-level data set of molecular risk factors for CVDs. Seven IVs (5%) have scaled CPOs below 0.01. (**B**) Log-CPOs after removing outliers, high-leverage and influential observations from the original data set. The MR^2 model is now considered to fit adequately since only two outliers or high-leverage and influential observations (2%) are still detected.

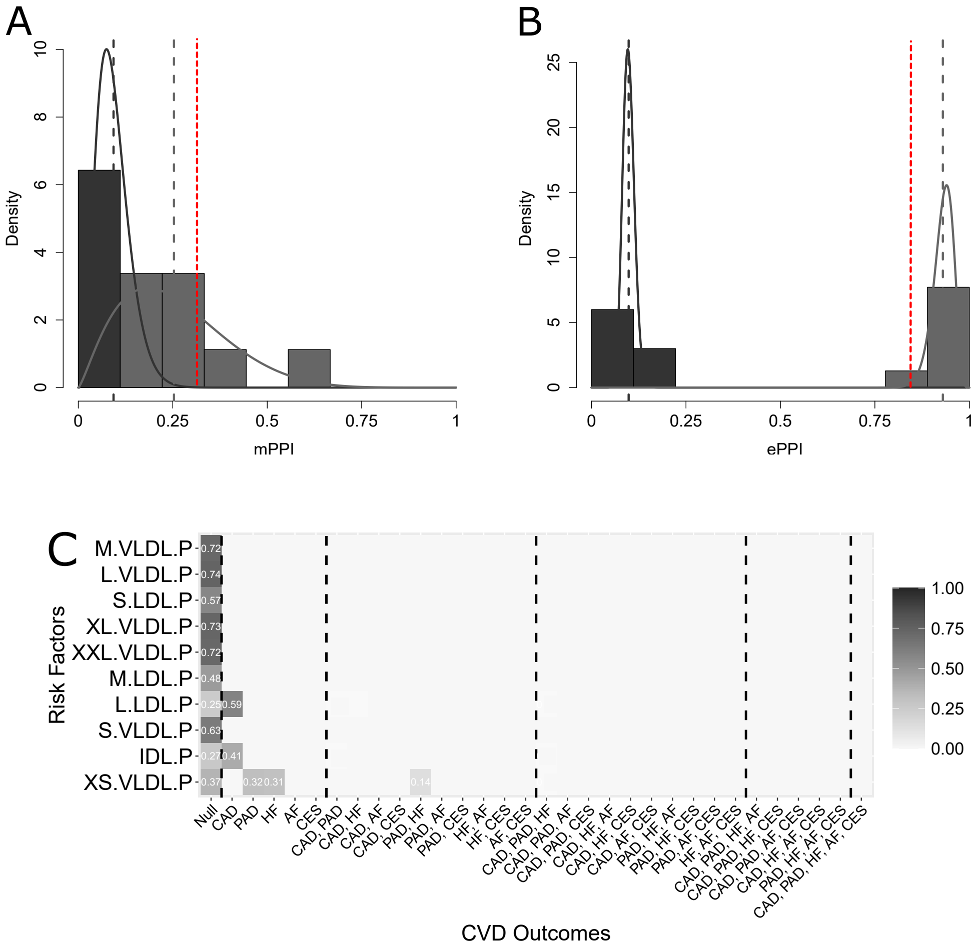

Figure S16. Post-processing results of the multi-response MR^2 model in application example 2 on molecular risk factors for cardiovascular disease outcomes. (**A**) Non-parametric False Discovery Rate (FDR) strategy based on two-component mixture model which clusters low and high levels of the marginal posterior probabilities of inclusion (mPPIs). Dark grey colour code indicates mPPIs and the estimated density of the null (H_0) component, while light grey is used for the alternative (H_1) component. Dark grey and light grey dotted vertical lines shows the means of the beta components, while red dotted vertical line specifies the threshold on the mPPI scale (mPPI ≥ 0.29) that corresponds to 5% FDR nominal level. (**B**) Non-parametric FDR strategy based on two-component mixture model which clusters low and high levels of the edge posterior probabilities of inclusion (ePPIs). Red dotted vertical line specifies the threshold on the ePPI scale (ePPI ≥ 0.85) that corresponds to 5% FDR nominal level.(**C**) Marginal and joint posterior probability of inclusion (jPPI) of each exposure (y-axis) against a single response or combinations of them (x-axis). Black dotted vertical lines separate combinations of outcomes with the same group size, *i.e.*, singletons, all possible pairs, all possible triplets, etc. For clarity of presentation, mPPIs for non-selected outcome-exposure pairs (mPPI < 0.29 at 5% FDR) are not plotted as well as jPPIs for combination of risk factors marginally not selected. The "Null" vertical bar on the left indicates the posterior model probability of the null model.

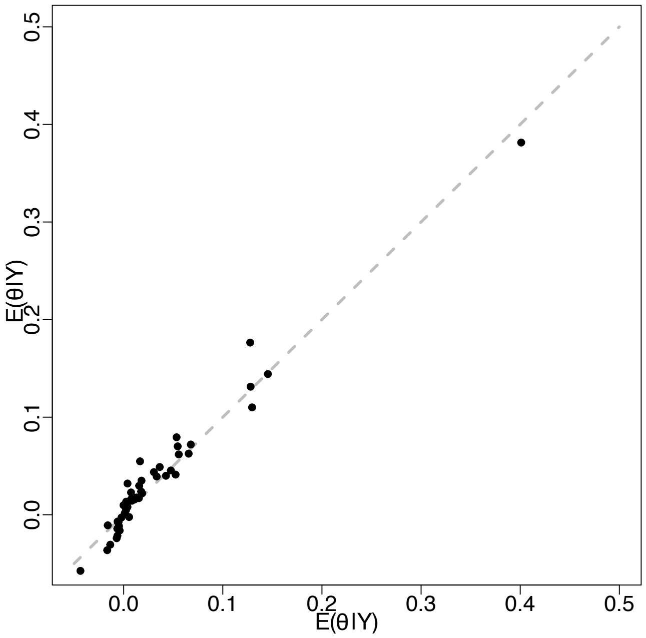

Figure S17. Sensitivity analysis of MR^2 with respected the *a priori* range of important exposures in application example 2 on molecular risk factors for cardiovascular disease outcomes. Robustness of the proposed method with respect to the level of sparsity controlled by $\mathbb{E}(\gamma_k)$ and $\mathbb{V}(\gamma_k)$, the *a priori* average number of important exposures associated with each response and its variance. In the original analysis, we fixed these values at $\mathbb{E}(\gamma_k) = 2$ and $\mathbb{V}(\gamma_k) = 2$. This prior choice implies *a priori* a range between zero and seven for the number of significant direct causal effects for each outcome. We changed them to $\mathbb{E}(\gamma_k) = 4$ and $\mathbb{V}(\gamma_k) = 4$ with *a priori* range between zero and 10. Scatterplot of the posterior mean of the direct causal effects in the original prior specification (x-axis) and the new specification (y-axis) shows no appreciable differences.

References

[1] Alexopoulos, A. & Bottolo, L. Bayesian Variable Selection for Gaussian copula regression models. *Journal of Computational and Graphical Statistics* **30**, 578–593 (2021). URL https://doi.org/10.1080/10618600.2020.1840997.

[2] Holmes, C. C. & Held, L. Bayesian auxiliary variable models for binary and multinomial regression. *Bayesian Analysis* **1**, 145–168 (2006). URL https://doi.org/10.1214/06-BA105.

[3] Rothman, A. J., Levina, E. & Zhu, J. Sparse multivariate regression with covariance estimation. *Journal of Computational and Graphical Statistics* **19**, 947–962 (2010). URL https://doi.org/10.1198/jcgs.2010.09188.

[4] Deshpande, S. K., Ročková, V. & George, E. I. Simultaneous variable and covariance selection with the multivariate spike-and-slab lasso. *Journal of Computational and Graphical Statistics* **28**, 921–931 (2019). URL https://doi.org/10.1080/10618600.2019.1593179.

[5] Wang, H. Sparse seemingly unrelated regression modelling: Applications in finance and econometrics. *Computational Statistics and Data Analysis* **54**, 2866–2877 (2010). URL https://doi.org/10.1016/j.csda.2010.03.028.

[6] George, E. & McCulloch, R. E. Variable selection via Gibbs sampling. *Journal of the American Statistical Association* **88**, 881–889 (1993). URL https://www.jstor.org/stable/2290777.

[7] Lauritzen, S. L. *Graphical Models* (Clarendon Press, Oxford, New York, 2004). Repr. with corrections.

[8] Hans, C., Dobra, A. & West, M. Shotgun stochastic search for "large p" regression. *Journal of the American Statistical Association* **102**, 507–516 (2007). URL https://doi.org/10.1198/016214507000000121.

[9] Burgess, S., Daniel, R. M., Butterworth, A. S. & Thompson, S. G. Network Mendelian randomization: Using genetic variants as instrumental variables to investigate mediation in causal pathways. *International Journal of Epidemiology* **44**, 484–495 (2015). URL https://doi.org/10.1093/ije/dyu176.

[10] Zuber, V., Colijn, J. M., Klaver, C. & Burgess, S. Selecting likely causal risk factors from high-throughput experiments using multivariable Mendelian randomization. *Nature Communications* **11**, 29 (2020). URL `https://doi.org/10.1038/s41467-019-13870-3`.

[11] Rees, J. M. B., Wood, A. M. & Burgess, S. Extending the MR-Egger method for multivariable Mendelian randomization to correct for both measured and unmeasured pleiotropy. *Statistics in Medicine* **36**, 4705–4718 (2017). URL `https://doi.org/10.1002/sim.7492`.

[12] Zuber, V. *et al.* High-throughput multivariable Mendelian randomization analysis prioritizes apolipoprotein B as key lipid risk factor for coronary artery disease. *International Journal of Epidemiology* **50**, 893–901 (2021). URL `https://doi.org/10.1093/ije/dyaa216`.

[13] Kettunen, J. *et al.* Genome-wide study for circulating metabolites identifies 62 loci and reveals novel systemic effects of LPA. *Nature Communications* **7** (2016). URL `https://doi.org/10.1038/ncomms11122`.

[14] Levin, M. G. *et al.* Prioritizing the role of major lipoproteins and subfractions as risk factors for peripheral artery disease. *Circulation* **144**, 353–364 (2021). URL `https://doi.org/10.1161/circulationaha.121.053797`.

[15] Congdon, P. *Bayesian Models for Categorical Data* (John Wiley & Sons, Chichester, 2005).

[16] Benjamini, Y. & Hochberg, Y. Controlling the false discovery rate: a practical and powerful approach to multiple testing. *Journal of the Royal Statistical Society: Series B (Methodological)* **57**, 289–300 (1995). URL `https://doi.org/10.1111/j.2517-6161.1995.tb02031.x`.

[17] Nielsen, J. B. *et al.* Biobank-driven genomic discovery yields new insight into atrial fibrillation biology. *Nature Genetics* **50**, 1234–1239 (2018). URL `https://doi.org/10.1038/s41588-018-0171-3`.

[18] Mishra, A. *et al.* Stroke genetics informs drug discovery and risk prediction across ancestries. *Nature* **611**, 115–123 (2022). URL `https://doi.org/10.1038/s41586-022-05165-3`.

[19] Nelson, C. P. *et al.* Association analyses based on false discovery rate implicate new loci for coronary artery disease. *Nature Genetics* **49**, 1385–1391 (2017). URL `https://doi.org/10.1038/ng.3913`.

[20] Klarin, D. *et al.* Genome-wide association study of peripheral artery disease in the Million Veteran Program. *Nature Medicine* **25**, 1274–1279 (2019). URL `https://doi.org/10.1038/s41591-019-0492-5`.

[21] Shah, S. *et al.* Genome-wide association and Mendelian randomisation analysis provide insights into the pathogenesis of heart failure. *Nature Communications* **11**, 163 (2020). URL `https://doi.org/10.1038/s41467-019-13690-5`.

[22] Liam, A. *et al.* UkBiobank GWAS results (2018). URL `http://www.nealelab.is/uk-biobank`.

[23] Wang, Z. *et al.* Genome-wide association analyses of physical activity and sedentary behavior provide insights into underlying mechanisms and roles in disease prevention. *Nature Genetics* **54**, 1332–1344 (2022). URL `https://doi.org/10.1038/s41588-022-01165-1`.

[24] Wootton, R. E. *et al.* Evidence for causal effects of lifetime smoking on risk for depression and schizophrenia: A Mendelian randomisation study. *Psychological Medicine* **50**, 2435–2443 (2020). URL `https://doi.org/10.1017/S0033291719002678`.

[25] Mahajan, A. *et al.* Fine-mapping type 2 diabetes loci to single-variant resolution using high-density imputation and islet-specific epigenome maps. *Nature Genetics* **50**, 1505–1513 (2018). URL `https://doi.org/10.1038/s41588-018-0241-6`.

[26] Ntzoufras, I. *Bayesian Modeling Using WinBUGS* (Wiley, Hoboken, N.J., 2009). URL `https://doi.org/10.1002/9780470434567`.
